# Major protein alterations in spermatozoa from infertile men with unilateral varicocele

**DOI:** 10.1186/s12958-015-0007-2

**Published:** 2015-02-22

**Authors:** Ashok Agarwal, Rakesh Sharma, Damayanthi Durairajanayagam, Ahmet Ayaz, Zhihong Cui, Belinda Willard, Banu Gopalan, Edmund Sabanegh

**Affiliations:** Center for Reproductive Medicine, Glickman Urological & Kidney Institute, Cleveland Clinic, Mail Code X-11, 10681 Carnegie Avenue, Cleveland, OH 44195 USA; Proteomics Research Core Services, Lerner Research Institute, Cleveland Clinic, Cleveland, OH 44195 USA

**Keywords:** Varicocele, Unilateral varicocele, Male infertility, Spermatozoa, Proteins, Proteomics, Bioinformatics

## Abstract

**Background:**

The etiology of varicocele, a common cause of male factor infertility, remains unclear. Proteomic changes responsible for the underlying pathology of unilateral varicocele have not been evaluated. The objective of this prospective study was to employ proteomic techniques and bioinformatic tools to identify and analyze proteins of interest in infertile men with unilateral varicocele.

**Methods:**

Spermatozoa from infertile men with unilateral varicocele (n = 5) and from fertile men (control; n = 5) were pooled in two groups respectively. Proteins were extracted and separated by 1-D SDS-PAGE. Bands were digested and identified on a LTQ-Orbitrap Elite hybrid mass spectrometer system. Bioinformatic analysis identified the pathways and functions of the differentially expressed proteins (DEP).

**Results:**

Sperm concentration, motility and morphology were lower, and reactive oxygen species levels were higher in unilateral varicocele patients compared to healthy controls. The total number of proteins identified were 1055, 1010 and 1042 in the fertile group, and 795, 713 and 763 proteins in the unilateral varicocele group. Of the 369 DEP between both groups, 120 proteins were unique to the fertile group and 38 proteins were unique to the unilateral varicocele group. Compared to the control group, 114 proteins were overexpressed while 97 proteins were underexpressed in the unilateral varicocele group. We have identified 29 proteins of interest that are involved in spermatogenesis and other fundamental reproductive events such as sperm maturation, acquisition of sperm motility, hyperactivation, capacitation, acrosome reaction and fertilization. The major functional pathways of the 359 DEP related to the unilateral varicocele group involve metabolism, disease, immune system, gene expression, signal transduction and apoptosis. Functional annotations showed that unilateral varicocele mostly affected small molecule biochemistry and post-translational modification proteins. Proteins expressed uniquely in the unilateral varicocele group were cysteine-rich secretory protein 2 precursor (CRISP2) and arginase-2 (ARG2).

**Conclusions:**

The expression of these proteins of interest are altered and possibly functionally compromised in infertile men with unilateral varicocele. If validated, these proteins may lead to potential biomarker(s) and help better understand the mechanism involved in the pathophysiology of unilateral varicocele in infertile men.

**Electronic supplementary material:**

The online version of this article (doi:10.1186/s12958-015-0007-2) contains supplementary material, which is available to authorized users.

## Background

Varicocele, which is the abnormal dilatation of the efferent veins in the pampaniform plexus, is the cause for one-third of all cases of male infertility [1]. Among adult males with varicoceles, 90% are present unilaterally on the left side while 10% are present bilaterally [2]. The incidence of varicocele ranges from 35% to 40% in men with primary infertility, but increases to 80% in men with secondary infertility, suggesting a progressive decline in male fertility [2,3]. However, not all varicoceles are related to male infertility, and men with high grade varicoceles can still father children [4]. The American Society for Reproductive Medicine (ASRM) guidelines for varicocele management recommends to treat a varicocele when it is palpable and present with at least one abnormal semen parameter, in the case of a couple presenting with infertility or when the female partner is normal [5].

The causes of varicocele are multiple and include increased scrotal temperature, stunted testicular growth, semen abnormalities, oxidative stress and Leydig cell dysfunction [2,3,6]. In addition, increased incidence of programmed cell death or apoptosis is consistently reported in varicocele cases [7]. Oxidative-stress induced apoptosis is associated with increased scrotal temperature, but not varicocele grade [8]. Varicoceles are also associated with decreased semen quality such as sperm count, motility, and morphology [9], increased oxidative stress and DNA damage [3,6,10,11]. Although conventional semen analysis is important in the evaluation of the infertile male, it has its flaws and offers low predictive value [12].

Much controversy still surrounds the diagnosis, management and pathophysiology of spermatogenic alterations associated with varicocele. Despite the availability of advanced techniques to measure reactive oxygen species (ROS), sperm DNA integrity and mitochondrial activity [1,3,10,13], the pathogenic and molecular mechanisms responsible for the etiology of varicoceles are not completely understood.

Research in male infertility investigating spermatozoa and seminal plasma proteomes is rapidly emerging in the post-genomic era. Documenting any changes in the protein composition of seminal plasma and spermatozoa might offer better understanding of the functional changes that occur in men suffering from various clinical etiologies associated with male infertility [14-16]. Although several studies have been published on the sperm proteome [17,18] that include comparative proteomics studies [19-21], very few reports have examined the effect of varicocele or varicocelectomy on proteomic profiling [22-26].

While identification of specific proteins to serve as markers of sperm function is critical, there are no reports in literature where the sperm proteome has been examined specifically for either unilateral or bilateral varicocele. The differentially expressed proteins in the varicocele group may be very different to those identified specifically in infertile men with unilateral varicocele disease. Documenting specific changes in the spermatozoa proteins may aid in the better understanding of the functional changes associated with men diagnosed with unilateral varicocele. Furthermore, the candidate proteins of interest may be utilized as potential markers of unilateral varicocele-related infertility.

In this study, we report of the spermatozoa proteins that are differentially expressed in healthy, fertile men and in infertile men with unilateral varicocele, the over- or underexpression of these proteins in the unilateral varicocele group and the crucial reproductive processes that these proteins are involved in. Upon validation, the suggested key spermatozoal proteins may serve as potential biomarkers of unilateral varicocele and may contribute towards the effectiveness of varicocele management.

## Methods

### Patients

Following approval of the study by the Institutional Review Board of Cleveland Clinic, semen samples were collected from 33 infertile patients with unilateral varicocele seeking investigation for fertility and from 10 healthy male volunteers (controls) of proven fertility. All patients and fertile men provided written consent to be enrolled in this prospective study. Following initial semen analysis and evaluation for white blood cells, measurement of ROS, TAC and DNA fragmentation, we pooled 5 samples from the unilateral varicocele group and 5 samples from the fertile group after normalizing for sperm and protein concentration for proteomic analysis.

### Clinical parameters

Varicocele was diagnosed by clinical analysis, including scrotal palpation in a temperature-controlled room (23.8°C) with adequate illumination, and varicocele was graded as described earlier by Dubin and Amelar [27]. 1) Varicocele grade I: dilatation of spermatic cord palpable only with a Valsalva maneuver; 2) Varicocele grade II: dilatation of spermatic cord that is easily palpable, with the patient standing, demonstrating marked venous dilatation during a Valsalva maneuver; and 3) Varicocele grade III: massive dilatation of spermatic cord that is easily visualized with the patient standing and intensified ectasia during a Valsalva maneuver.

### Inclusion criteria

Patients with surgical indication between the ages of 20–40 years, referred to the Urology Department of Cleveland Clinic from March 2012 to April 2014 were included in the study. All the patients had been screened and were non-smokers and had a normal body mass index. None of them had been exposed to environmental stressors, including radiation or chemicals. All female partners of the infertile men had undergone gynecologic evaluation and had normal results on a fertility workup.

### Exclusion criteria

Patients were excluded from the study if they had a recurring fever in the 90-day period prior to semen analysis with evidence of urogenital infection or any other reproductive or urological diseases diagnosed by andrological examination, genetic defects, and/or occupational exposure to spermatogenetic-toxic chemicals. Similarly, men with azoospermia and a sperm concentration <10 million sperm/mL were not included in the present study. Endtz-positive samples were also excluded to avoid the interference from leucocytes in semen samples. Controls were healthy men with normal semen parameters who had fathered at least one healthy child without assisted reproductive measures. All fertile men were ruled out for presence of clinical varicocele. Both infertile men and control subjects did not present with, or have a history of, any systemic illnesses, cryptorchidism, orchitis, epididymitis, urethritis, testicular atrophy, or sexually transmitted diseases, including human immunodeficiency virus.

### Semen analysis

All specimens were collected by masturbation at the Andrology Laboratory after 48–72 hours of sexual abstinence. Samples were allowed to liquefy completely for 15–30 minutes at 37°C before further processing. Following liquefaction, manual semen analysis was performed using a MicroCell counting chamber (Vitrolife, San Diego, CA) to determine sperm concentration and motility. Semen analysis was performed according to the WHO guidelines [28] to evaluate sperm count, motility and presence of round cells. Viability was determined by Eosin-Nigrosin stain. Smears of the raw semen were stained with a Diff-Quik kit (Baxter Healthcare Corporation, Inc., McGaw Park, IL) for assessment of sperm morphology according to Kruger’s’ Strict criteria as described in the WHO, 2010 guideline [28].

### White blood cell measurement

When the round cell concentration in the ejaculate was >1 × 10^6^/mL or >5 round cells per high power field, the sample was tested for leukoctyospermia, i.e. >1 × 10^6^ white blood cells/mL. This was confirmed by the peroxidase or the Endtz test. A 20-μL well-mixed aliquot of the semen sample was mixed with one volume of phosphate buffered saline (PBS) and 2 volumes of working Endtz solution in an amber-colored eppendorf tube [21]. After 5 minutes, a drop of the aliquot was placed on a Makler chamber and examined for the presence of dark brown cells under ×10 bright field objective.

### Measurement of reactive oxygen species, total antioxidant capacity and sperm DNA fragmentation

ROS formation was measured by chemiluminescence assay using luminol (5-amino-2, 3-dihydro-1, 4-phthalazinedione) as the probe. Test samples consisted of luminol (10 μL, 5 mM) and 400 μL of sperm suspension. Chemiluminescence was measured for 15 min using a Berthold luminometer (Autolumat Plus 953, Oakridge, TN). Results were expressed as relative light units (RLU)/sec/ × 10^6^ sperm [29]. Clear seminal plasma was aliquoted and frozen at −55°C until total antioxidant capacity (TAC) was measured using the antioxidant assay kit (Cayman Chemical, Ann Arbor, Mich.). Absorbance was monitored at 750 nm using ELx800 Absorbance Microplate Reader (BioTek Instruments, Inc., Winooski, Vt.). Results were expressed as micromoles of Trolox. Sperm DNA fragmentation was evaluated using a terminal deoxynucleotidyl transferase–mediated fluorescein end labeling (TUNEL) assay with an Apo-Direct kit (Pharmingen, San Diego, CA). All fluorescence signals of labeled spermatozoa were analyzed by the flow cytometer FacScan (Becton Dickinson, San Jose, CA). The percentage of positive cells (TUNEL-positive) was calculated on a 1023-channel scale using the flow cytometer software FlowJoMac version 8.2.4 (FlowJo, LLC, Ashland, OR) [30].

After conducting routine semen analysis, ROS measurement as well as DNA measurement, the remainder of the samples was examined for proteomic analysis. For proteomic study, it is important to normalize the protein concentration so that an equal amount of protein is contributed by an equal number of spermatozoa. This limited the number of samples that had the requisite number of spermatozoa to provide the protein concentration required for proteomic analysis. To balance the fertile and varicocele study groups, we pooled the same number of samples from the fertile group.

### Preparation of samples for proteomic analysis

#### Protein extraction

Two pools each comprising of 5 samples from the unilateral varicocele group and 5 from the fertile group was created. The 5 samples were selected purely on the basis of the sperm concentration necessary to obtain the required number of proteins for proteomic analysis i.e. normalization of the sample to minimize the biological variance. Samples with extremely low sperm concentration were excluded by default. Each pooled set was washed with PBS three times. Once the supernatant was removed, the spermatozoa were solubilized in radio-immunoprecipitation assay (RIPA) lysis buffer (Sigma-Aldrich, St. Louis, MO) containing the proteinase inhibitor cocktail (Roche, Indianapolis, IN). The spermatozoa samples were stored overnight at 4°C to allow for complete lysis of the spermatozoa, which included the cell membranes. After centrifugation at 13,000 g for 20 minutes, the supernatant was aspirated and the protein concentration was determined using a bicinchoninic acid (BCA) kit (Thermo, Rockford, IL). The results from the BCA assay were used to dilute equal amounts of protein in SDS-PAGE sample buffer. These samples were then fractionated using 1D SDS-PAGE gel electrophoresis.

#### Proteomic analysis

Global proteomic analysis was done in triplicate for both the patient and control groups and quantified using the label-free spectral counting method. A 15-μg aliquot of each sample was boiled, and a standard SDS-PAGE was run on a 12.5% Tris–HCl 1D gel with constant voltage of 150 V for 35 min. The gel was run for 1/3 of the total length to prepare for the downstream GelC experiment. The gel was fixed for 30 min in 50% ethanol/10% acetic acid, washed with water thoroughly and stained with Coomassie blue. For the protein digestion, the entire gel lane was cut and divided into 6 smaller pieces. The gel pieces were washed with water and dehydrated in acetonitrile. The bands were then reduced with Dithiothreitol (DTT) and alkylated with iodoacetamide prior to the in-gel digestion. All bands were digested in-gel using trypsin by adding 5 μL 10 ng/μL trypsin in 50 mM ammonium bicarbonate. They were incubated overnight at room temperature to achieve complete digestion and then extracted from the polyacrylamide in two aliquots of 30 μL 50% acetonitrile with 5% formic acid. Gels from the fertile control group and the unilateral varicocele group were run in triplicate to examine the technical reproducibility of the assay.

### Liquid chromotography mass spectrometer analysis (LC-MS)

The extracts were combined and evaporated to <10 μL in Speedvac and then resuspended in 1% acetic acid to make a final volume of ~30 μL for LC-MS analysis. The LC-MS system was a Finnigan LTQ-Orbitrap Elite hybrid mass spectrometer system. The HPLC column was a Dionex 15 cm × 75 μm internal diameter Acclaim Pepmap C18, 2 μm, 100 Å reversed phase capillary chromatography column. Five μL of the extract was injected, and the peptides eluted from the column by an acetonitrile/0.1% formic acid gradient at a flow rate of 0.25 μL/min were introduced into the source of the mass spectrometer on-line. The microelectrospray ion source was operated at 2.0 kV. The digest was analyzed using the data dependent multitask capability of the instrument acquiring full scan mass spectra to determine peptide molecular weights and tandem mass spectra (MS/MS) to determine amino acid sequence in successive instrument scans.

### Data analysis

For semen parameters, analysis of variance (ANOVA) and two-sample T-tests were used to compare the unilateral varicocele patients with the fertile men with respect to the quantitative measurements of protein expression. The t-tests comparing pairs of groups were performed at individual significance levels of <0.05 without adjustment for multiple comparisons.

### Database searching

Tandem mass spectra were extracted by Proteome Discoverer version 1.4.1.288. Charge state deconvolution and de-isotoping were not performed. All MS/MS samples were analyzed using Mascot (Matrix Science, London, UK; version 2.3.02), Sequest (Thermo Fisher Scientific, San Jose, CA, USA; version 1.4.0.288) and X! Tandem (The GPM, thegpm.org; version CYCLONE (2010.12.01.1). Mascot, Sequest and X!Tandem were set up to search the human reference with database (33292 entries) assuming the digestion enzyme trypsin. These searches were performed with a fragment ion mass tolerance of 1.0 Da, and parent ion tolerance of 10 parts per million (PPM). Carbamidomethyl of cysteine was specified as a fixed modification, and oxidation of methionine was specified as variable modifications.

### Criteria for protein identification

To validate MS/MS-based peptide and protein identifications Scaffold (version Scaffold 4.0.6.1, Proteome Software Inc., Portland, OR) was used. Peptide identifications were accepted if they could be established at >95.0% probability by the Peptide Prophet algorithm [31] with Scaffold delta-mass correction. Protein identifications were accepted if they could be established at > 99.0% probability to achieve a false detection rate (FDR) <1.0% and contained at least 2 identified peptides. Protein probabilities were assigned by the Protein Prophet algorithm [32]. Proteins that contained similar peptides and could not be differentiated based on MS/MS analysis alone were grouped to satisfy the principles of parsimony. Proteins were annotated with gene ontology (GO) terms from National Center for Biotechnology Information (NCBI) (downloaded Oct 21, 2013) [33].

### Quantitative proteomics

For proteomic analysis, the relative quantity of the proteins was determined by comparing the number of spectra, termed spectral counts, used to identify each protein. The total number of mass spectra that matched peptides to a particular protein (Spectral counts or SpCs) was used to measure the abundance of proteins in the complex mixture. Normalization of spectral counts using the NSAF (normalized spectral abundance factor) approach [34,35] was applied prior to relative protein quantification. This approach takes into account the sample-to-sample variation that is obtained when performing replicate analyses of a sample and the fact that longer proteins tend to have more peptide identifications than shorter proteins.

Differentially expressed proteins (DEPs) were obtained by applying different constraints for significance tests and/or fold change cutoffs based on the average SpC of the protein from multiple runs, as accurate quantification and determination of real biological change is a function of absolute number of SpCs.

In proteomic analysis, errors are more common when proteins are less abundant. Due to this, the filters used to identify differentially expressed proteins were dependent on the overall abundance of the proteins. It has been reported [36] that accurate quantification and determination of real biological change is dependent on the number of SpCs and hence, different constraints must be applied to SpC levels to circumvent the biases and maintain a constant false positive rates (FPR) for all proteins. Higher SpC have low variance data and as a result, require less stringent fold change cutoffs to achieve accurate quantification. Conversely, lower SpC proteins showed less reproducibility and required higher fold change cut-offs. Based on these findings, the abundance of the proteins were classified as High (H), Medium (M), Low (L), or Very Low (VL) based on their average spectral counts amongst the 3 replicate runs. Different constraints for significance tests (p-value) and/or fold change cutoffs (or NSAF ratio) were applied for these 4 abundance categories, as shown below:

- VL: spectral count range 1.7-7; p ≤ 0.001 and (NSAF ratio ≥ 2.5 for Up, ≤ 0.4 for Down)
- Low: spectral count range 8–19; p ≤ 0.01 and (NSAF ratio ≥2.5 for Up, ≤ 0.4 for Down)
- Medium: spectral count range between 20–79; p ≤ 0.05 AND (NSAF ratio ≥ 2.0 for Up, ≤ 0.5 for Down)
- High: spectral counts >80; p ≤ 0.05 and (NSAF ratio ≥ 1.5 for Up, ≤ 0.67 for Down)

Accurate quantification and determination of real biological change is dependent on the spectral counts abundance and hence different statistical (i.e., P value) and biological (i.e. fold change) constraints must be applied (to the four categories - high, medium, low, very low - identified based on their spectral count levels) to circumvent the biases and maintain a constant FPR for all proteins. These criteria were based on control experiments that analyzed two identical samples using the NSAF spectral counting method.

### Bioinformatics analysis

Functional annotation and enrichment analysis were performed using publicly available bioinformatic annotation tools and databases such as GO Term Finder [37], GO Term Mapper, UniProt, Software for researching annotations of proteins (STRAP) [38] and database for annotation, visualization and integrated discovery (DAVID) (http://david.niaid.nih.gov). Proprietary software packages such as IPA (Ingenuity Pathway Analysis) from Ingenuity® Systems were also used to obtain consensus-based, comprehensive functional context for the large list of proteins derived from proteomic study.

## Results

Thirty-one of the 33 infertile patients (94%) had a left-sided varicocele; of these, 83.6% presented with grade 1 or 2 varicocele (26/31), 16.1% with grade 3 or 4 (5/31), and 6.5% presented with a right sided varicocele (2/33). For the proteomic analysis, 5 samples from the unilateral varicocele group and 5 samples from the control group were pooled. These 5 samples were selected purely on the basis of the sperm concentration necessary to obtain the required number of proteins for proteomic analysis i.e. normalization of the sample to minimize the biological variance. Samples with extremely low sperm concentration were excluded by default.

### Semen analysis

The average age of the fertile men was comparable to that of the patients (40.00 ± 9.8 years vs. 36.3 ± 7.7 years). Sperm concentration was significantly lower in the unilateral varicocele group than in the control group (30.75 ± 28.19 × 10^6^ vs. 69.90 ± 37.65 × 10^6^ sperm/ mL (P = 0.006). As expected, motility was also significantly lower in the patients (41.5 ± 17.3%) than in the control group (57.1 ± 16.0%) (P = 0.021). While none of the fertile men were positive for Endtz, 24% (8 of the 33) were mildly positive, but below 1× 10^6^ white blood cells/ mL among the unilateral varicocele group. Similarly, there was a significant decline in the morphology in the varicocele group compared with the controls (2.6% ± 1.9% vs. 8.4% ± 3.7%) (P < 0.001). Levels of ROS [median (25th, 75th percentile) RLU/sec/ ×10^6^ sperm] were significantly higher in the men with unilateral varicocele [494.8 (114.2, 2547.8)] than in the controls [142.7 (36.2, 337.7)] (P = 0.03) while TAC levels were comparable between the controls and patients (1840.38 ± 403.05 vs. 2029.33 ± 567.46; p = 0.34) micromoles Trolox. Sperm DNA damage in the unilateral varicocele group ranged from 3.3% to 35.3% compared with 8.5% to 18.2% in the controls. For proteomic study, 5 subjects from each group were pooled. Fertile men were negative for leukocytes; all had normal morphology and ROS levels were <200 RLU/s/ × 10^6^sperm.

### Identification of the proteome in varicocele and fertile men by proteomic analysis

For the global proteomic profiling analysis, each pooled sample from the control and unilateral varicocele groups was run in triplicate, and a total of 1191 proteins were identified. For the fertile group, a total of 1055, 1010 and 1042 proteins were identified in the 3 LC-MS runs. Among the proteins identified in the fertile group, some of the most abundant components included semenogelin-2 precursor (SEMG2), semenogelin-1 preprotein (SEMG1), lactotransferrin (LTF), A-kinase anchor protein 4 isoform 2 (AKAP4), tubulin beta-4B chain (TUBB4B) (see Additional file 1: Tables S1, Additional file 2: Table S2, Additional file 3: Table S3). A significantly lower number of proteins were identified (795, 713 and 763 proteins respectively) in the unilateral varicocele group (see Additional file 4: Tables S4, Additional file 5: Table S5, Additional file 6: Table S6).

We categorized proteins according to their abundance into very low, low, medium and high abundance proteins based on the number of spectra identified to determine the experimental variability. The identification of a lower number of proteins in the varicocele group compared to the fertile group may be due to the increased presence of low abundance proteins in the unilateral varicocele group. In proteomic analysis, Low abundance proteins have considerably higher variability due to the complex nature of the samples as well as the run-to-run variability of the LC-MS/MS experiment.

Some of the more abundant proteins in the unilateral varicocele group were lactotransferrin isoform 1 precursor (LTF), fibronectin isoform 3 preprotein (FN1), semenogelin-2 precursor (SEMG2), A-kinase anchor protein 4 isoform 2 (AKAP4), and semenogelin-1 preprotein (SEMG1).

### Identification of differentially expressed proteins among the varicocele infertile and fertile men

**Figure 1:**
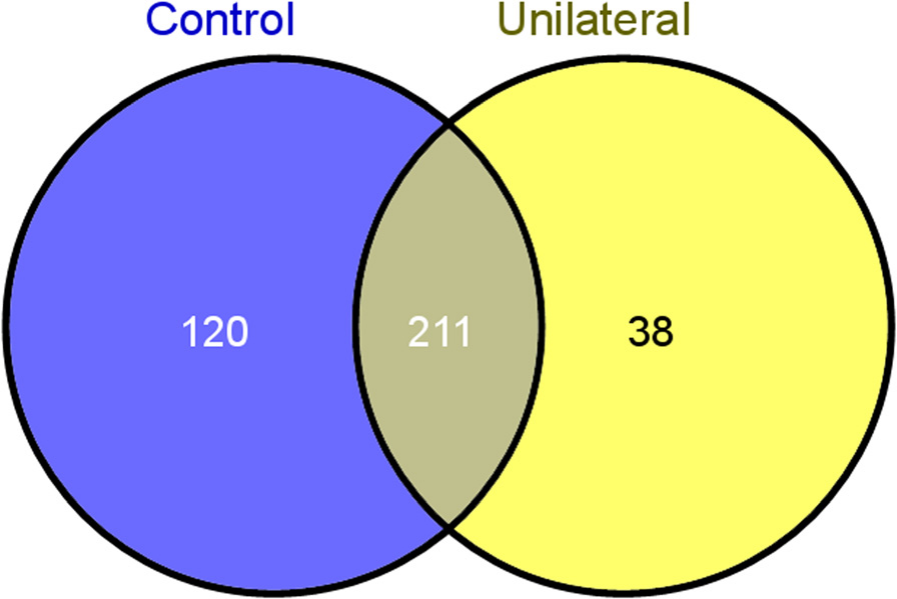
**Venn diagram showing the distribution of 369 differentially expressed proteins in the fertile group and infertile men with unilateral varicocele.** Of the 369 proteins, 120 proteins were unique to the fertile group and 38 to the unilateral varicocele group. 211 proteins were common and were either overexpressed or underexpressed.

**Figure 2:**
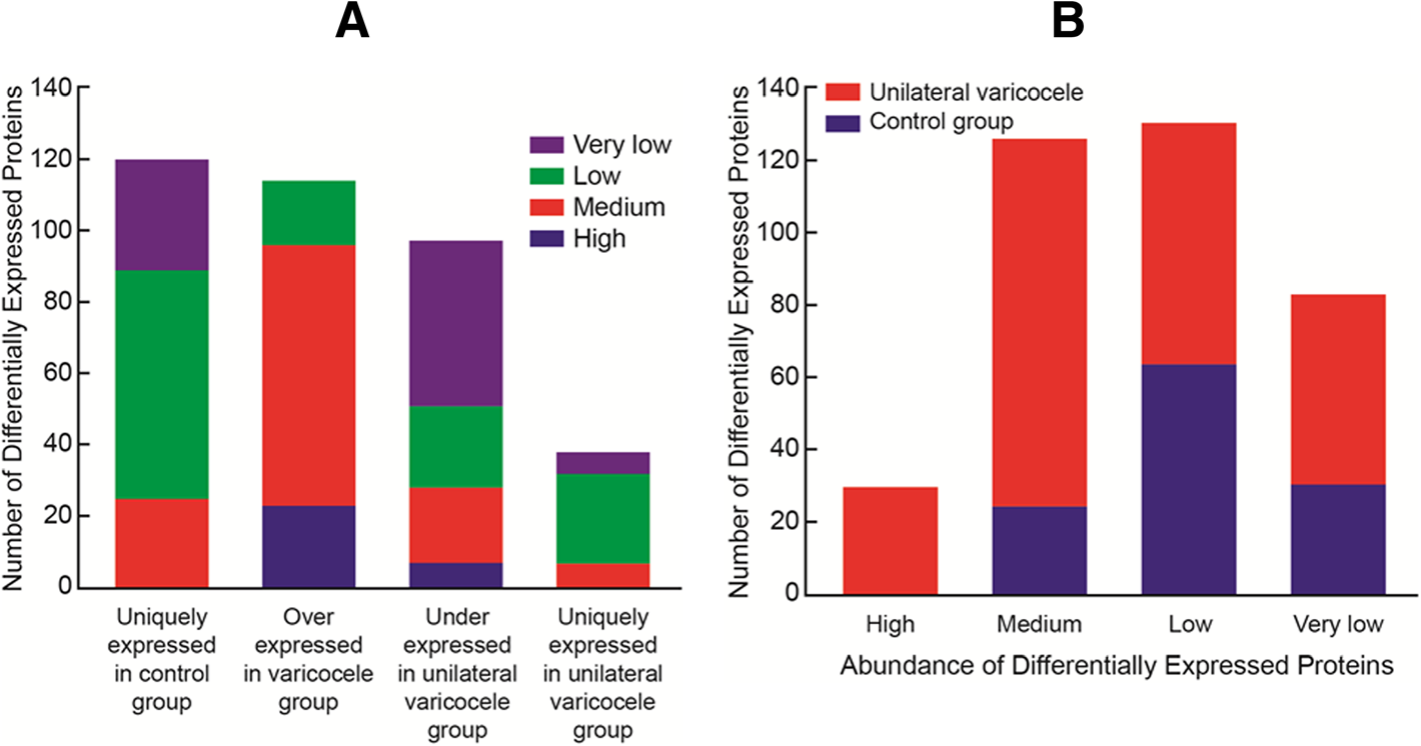
**Expression levels of differentially expressed proteins. A**. The number of DEPs that were uniquely expressed either in the fertile or unilateral varicocele group and their respective levels of expression (up-regulated or down-regulated) in the two groups. **B**. Expression levels of differentially expressed proteins. The abundance of differentially expressed proteins - high, medium, low or very low in the fertile and unilateral varicocele group.

The protein quantitation was performed using the Normalized Spectral Abundance Factor (NSAF), which is based on spectra counts, and therefore all 1191 proteins were quantified in this analysis. Differentially expressed proteins (DEP) were identified based on the filtering criteria given in the procedures section. A total of 369 DEP were identified in the infertile men with varicocele and fertile controls. The Venn diagram shows the distribution of these proteins (Figure 1). 120 of the DEP were unique to the fertile group and 38 were unique to unilateral varicocele group. 211 proteins were common to both groups and were either overexpressed or underexpressed. Among the 120 proteins unique to the fertile control group, 64 were present in the low abundance, 31 in very low abundance and 25 proteins in medium abundance. Thirty eight proteins were unique to the unilateral varicocele group. Of these, 7/38 (18.4%) proteins showed medium expression level, 25/38 (65.8%) in low abundance and 6/38 (15.8%) very low abundance. When compared to the control group, 114 proteins were overexpressed in the varicocele group, of these 23/114 (20.2%) proteins showed high abundance; 73/114 (64%) medium abundance and 18/114 (15.8%) low abundance. Similarly compared to the control group, 97 proteins were underexpressed in the unilateral varicocele group, of which 23/97 (23.7%) proteins showed high abundance, 73/97 (75.3%) exhibited medium expression levels and 18/97 (18.6% low abundance (Figure 2A). The distribution of high, medium, low and very low proteins in the control and the unilateral varicocele groups is shown in Figure 2B.

### Classification of the differentially expressed protein based on their cellular location, molecular function and role in biological processes

**Figure 3:**
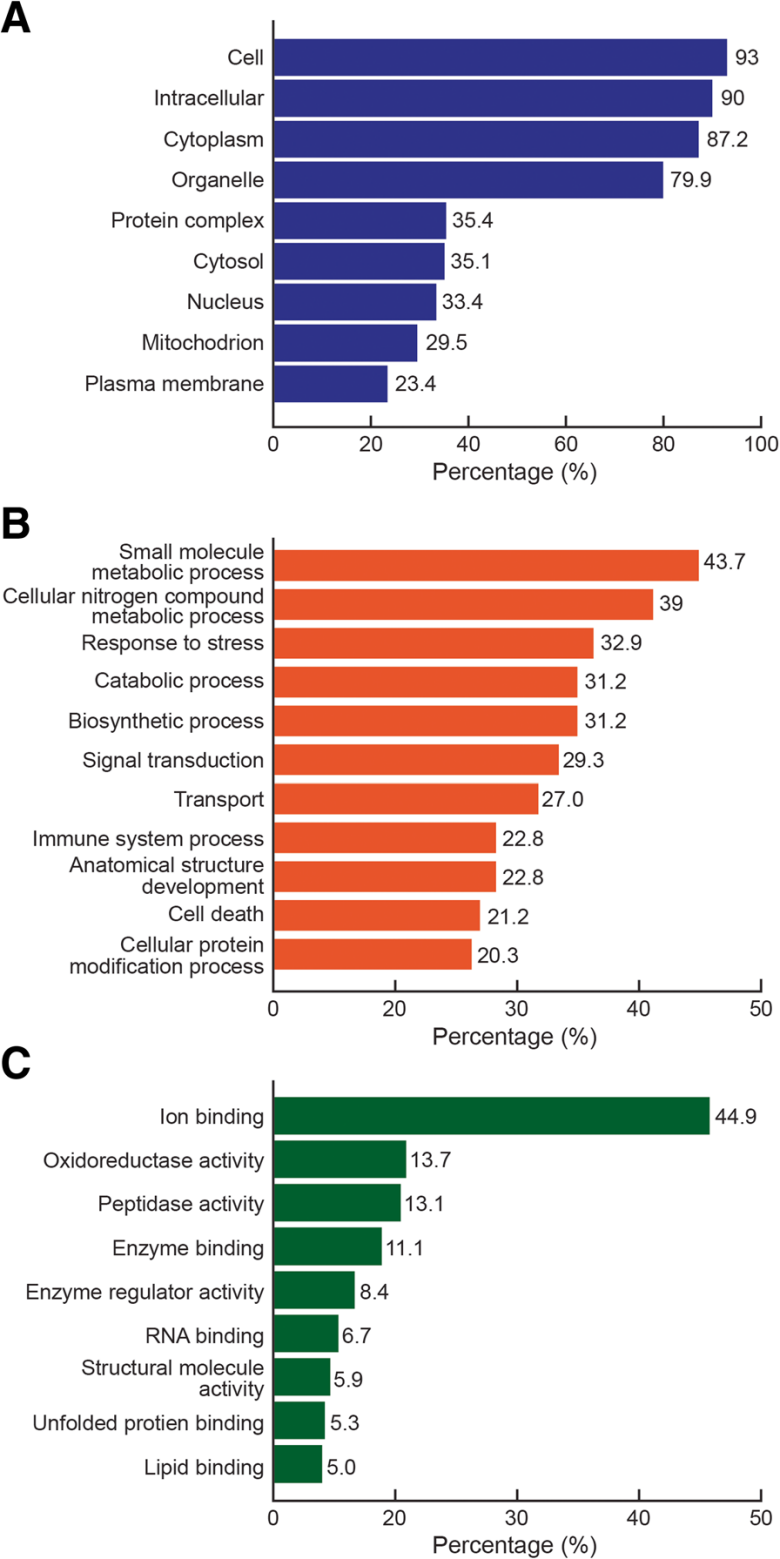
**Gene Ontology annotations for differentially expressed proteins. A**: Cellular distribution **B**: Biological processes and **C**: Molecular functions.

To better understand the functional relevance of the proteins identified by proteomic analyses in the varicocele and fertile groups, GO Term Mapper was used to classify the DEP involved in major cellular and molecular functions and biological processes as well as to analyze their cellular localization (Tables 1, 2 and 3 and Figure 3A-C).

**Table 1:** **Differentially expressed proteins (DEP) participating in various cellular pathways**

**GO biological process terms**	**Percentage of genes**	**GO term usage in gene list**
Cell	93.04	334/359
Intracellular	89.97	323/359
Cytoplasm	87.19	313/359
Organelle	79.94	287/359
Protein complex	35.38	127/359
Cytosol	35.10	126/359
Nucleus	33.43	120/359
Mitochondrion	29.53	106/359
Plasma membrane	23.40	84/359
Extracellular region	19.22	69/359
Cytoplasmic membrane-bounded vesicle	13.93	50/359
Cytoskeleton	13.09	47/359
Extracellular space	10.86	39/359
Endoplasmic reticulum	10.58	38/359
Nucleoplasm	9.75	35/359
Vacuole	5.85	21/359
Lysosome	5.29	19/359
Cilium	5.29	19/359
Golgi apparatus	4.74	17/359
Microtubule organizing center	3.90	14/359
Nucleolus	3.34	12/359
Endosome	3.06	11/359
Nuclear envelope	3.06	11/359
Proteinaceous extracellular matrix	3.06	11/359
Chromosome	2.51	9/359
Ribosome	2.23	8/359
Peroxisome	1.95	7/359
Nuclear chromosome	1.11	4/359
External encapsulating structure	0.28	1/359
Lipid particle	0.28	1/359
Cell wall	0.28	1/359

Note: Of the 369 differentially expressed proteins (DEP), only 359 are involved in the pathways according to the Gene Ontology mapper.

**Table 2:** **Differentially expressed proteins (DEP) participating in various cellular molecular functions**

**GO biological process terms**	**Percentage of genes**	**GO term usage in gene list**
Ion binding	44.85	161/359
Oxidoreductase activity	13.65	49/359
Peptidase activity	13.09	47/359
Enzyme binding	11.14	40/359
Enzyme regulator activity	8.36	30/359
RNA binding	6.69	24 of 359
Structural molecule activity	5.85	21/359
Unfolded protein binding	5.29	19/359
Lipid binding	5.01	18/359
DNA binding	4.46	16/359
ATPase activity	3.90	14/359
Cytoskeletal protein binding	3.62	13/359
Transmembrane transporter activity	3.62	13/359
Ligase activity	2.79	10/359
Kinase activity	2.79	10/359
Isomerase activity	2.79	10/359
Phosphatase activity	2.51	9/359
Transferase activity, transferring acyl groups	2.51	9/359
Lyase activity	2.23	8/359
Hydrolase activity, acting on carbon-nitrogen bonds	2.23	8/359
Structural constituent of ribosome	1.67	6/359
Hydrolase activity, acting on glycosyl bonds	1.67	6/359
Protein binding transcription factor activity	1.67	6/359
Signal transducer activity	1.39	5/359
GTPase activity	1.39	5/359
mRNA binding	1.11	4/359
Nucleic acid binding transcription factor activity	1.11	4/359
Protein transporter activity	1.11	4/359
Transferase activity, transferring glycosyl groups	1.11	4/359
Translation factor activity, nucleic acid binding	0.84	3/359
Nuclease activity	0.84	3/359
Transcription factor binding	0.84	3/359
Small conjugating protein binding	0.56	2/359
Protein binding, bridging	0.56	2/359
Helicase activity	0.56	2/359
Transferase activity, transferring alkyl or aryl groups	0.28	1/359
rRNA binding	0.28	1/359

Note: Of the 369 differentially expressed proteins (DEP), only 359 are involved in the pathways according to the Gene Ontology mapper.

**Table 3:** **Differentially expressed proteins (DEP) participating in various biological processes**

**GO molecular functional terms**	**Percentage of genes**	**GO term usage in gene list**
Small molecule metabolic process	43.73	157/359
Cellular nitrogen compound metabolic process	39.00	140/359
Response to stress	32.87	118/359
Biosynthetic process	31.20	112/359
Catabolic process	31.20	112/359
Signal transduction	29.25	105/359
Transport	27.02	97/359
Anatomical structure development	22.84	82/359
Immune	22.84	82/359
Cell death	21.17	76/359
Cellular protein modification process	20.33	73/359
Cell differentiation	16.16	58/359
Cell cycle	15.04	54/359
Cellular amino acid metabolic process	14.48	52/359
Cellular component assembly	14.21	51/359
Symbiosis, encompassing mutualism through parasitism	13.93	50/359
Generation of precursor metabolites and energy	13.09	47/359
Carbohydrate metabolic process	12.81	46/359
Homeostatic process	11.98	43/359
Macromolecular complex assembly	11.42	41/359
Lipid metabolic process	11.14	40/359
Locomotion	10.58	38/359
Reproduction	10.58	38/359
Protein complex assembly	10.31	37/359
Cell proliferation	9.75	35/359
Membrane organization	9.47	34/359
Cell motility	8.91	32/359
Transmembrane transport	8.36	30/359
Vesicle-mediated transport	8.08	29/359
Nucleobase-containing compound catabolic process	7.80	28/359
Protein folding	6.96	25/359
Growth	6.41	23/359
Protein targeting	5.85	21/359
Cell adhesion	5.85	21/359
Translation	5.85	21/359
Cofactor metabolic process	5.85	21/359
Anatomical structure formation involved in morphogenesis	5.85	21/359
Neurological system process	5.57	20/359
Cell morphogenesis	5.57	20/359
Cell-cell signaling	5.57	20/359
Circulatory system process	4.74	17/359
Mitochondrion organization	4.46	16/359
DNA metabolic process	4.18	15/359
Cytoskeleton organization	4.18	15/359
Embryo development	3.90	14/359
Nucleocytoplasmic transport	3.62	13/359
Sulfur compound metabolic process	3.34	12/359
Protein maturation	2.79	10/359
Aging	2.51	9/359
Cell division	2.51	9/359
Extracellular matrix organization	2.23	8/359
tRNA metabolic process	1.95	7/359
Chromosome organization	1.95	7/359
Cell junction organization	1.67	6/359
Mitosis	1.39	5/359
Ribosome biogenesis	1.39	5/359
Developmental maturation	1.11	4/359
mRNA processing	1.11	4/359
Ribonucleoprotein complex assembly	0.84	3/359
Cytoskeleton-dependent intracellular transport	0.84	3/359
Plasma membrane organization	0.56	2/359
Cell wall organization or biogenesis	0.28	1/359
Nitrogen cycle metabolic process	0.28	1/359
Chromosome segregation	0.28	1/359
Pigmentation	0.28	1/359

Note: Of the 369 differentially expressed proteins (DEP), only 359 are involved in the pathways according to the Gene Ontology mapper.

### Major pathways of DEPs identified by the Reactome database

**Figure 4:**
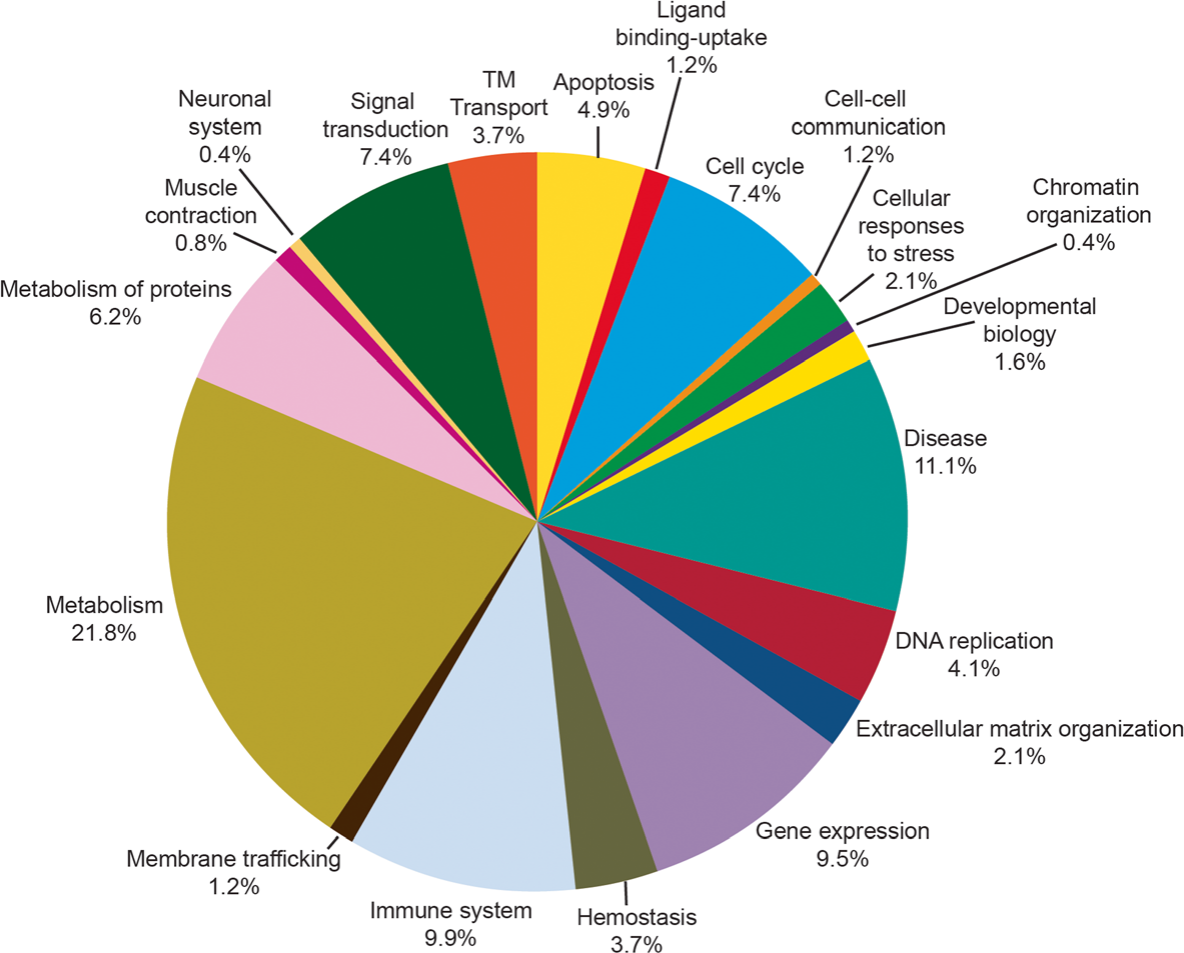
**Major pathways identified by the Reactome database.** The major pathways identified by the Reactome database showing differentially expressed proteins participating in metabolism, disease, immune system, gene expression, signal transduction, apoptosis and other pathways.

Of the 359 DEP, only 219 were assigned to the Reactome database. The major pathways identified included metabolism (21.8%), disease (11.1), immune system (9.9%), gene expression (9.5%;) and signal transduction (7.4%). The percent distribution of DEP in these pathways is also shown in Figure 4.

### Participation of DEPs in the top networks and pathways

**Figure 5:**
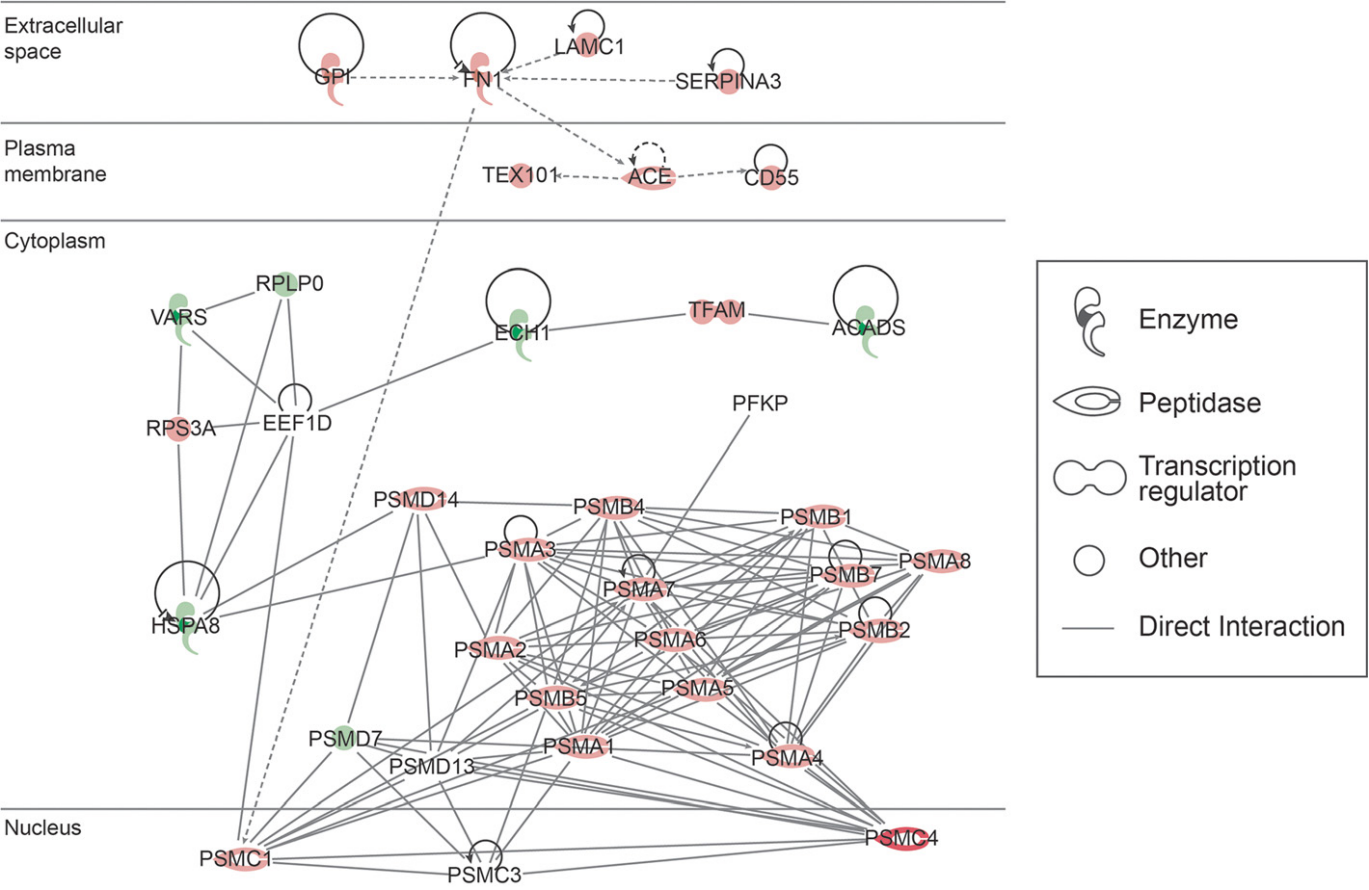
**Top disease and function networks and involvement of differentially expressed proteins in cell death and survival, metabolic disease, inflammatory disease.** Green color shows that these differentially expressed proteins (DEPs) were underexpressed and red shows overexpression of DEPs in unilateral varicocele group compared to the fertile group. Gradation of color reflects their intensity/ abundance of expression (e.g. brighter the red, the larger the protein expression).

**Figure 6:**
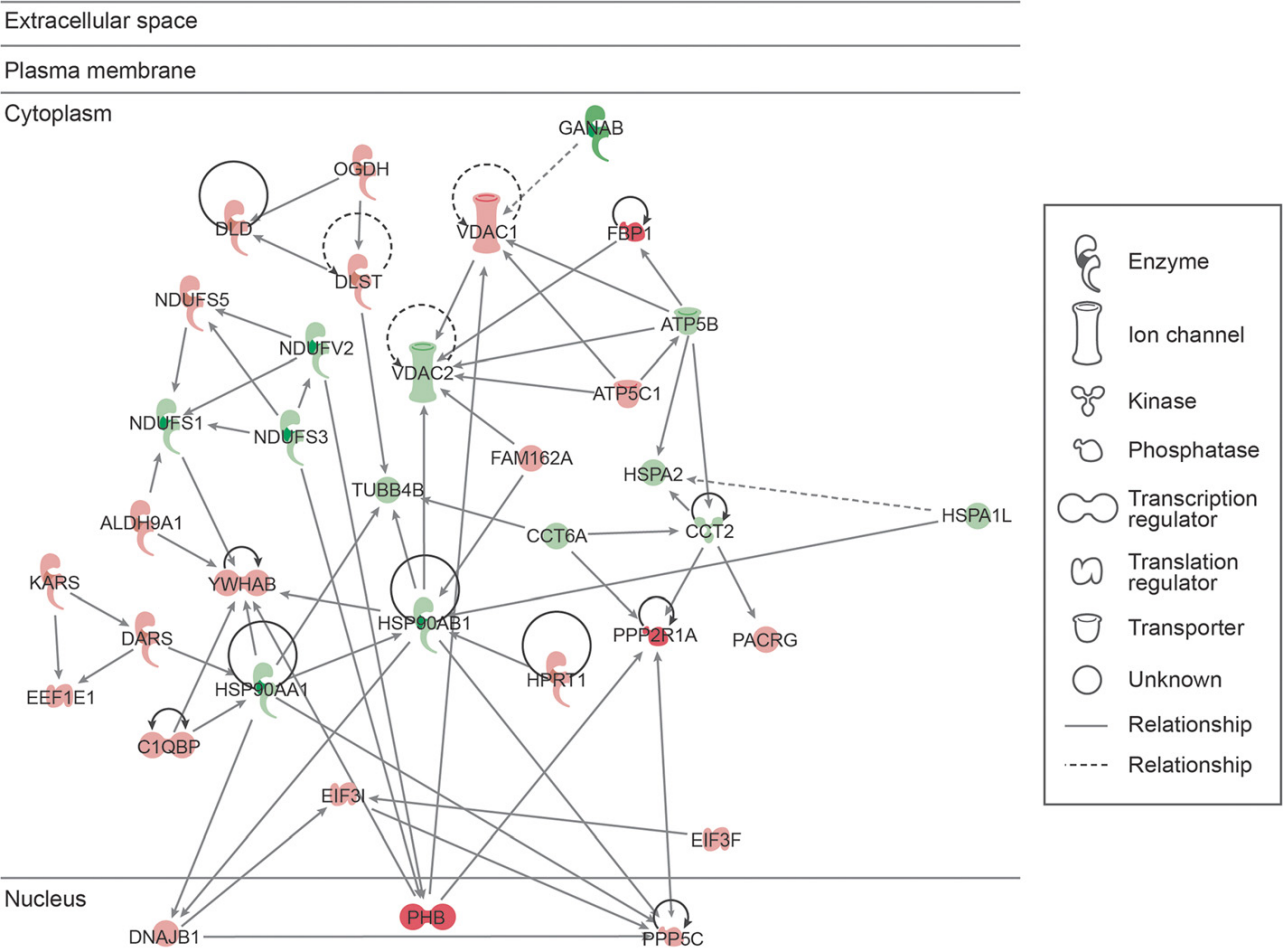
**Top disease and function networks and involvement of differentially expressed proteins in nucleic acid metabolism, small molecule biochemistry, drug metabolism.** Green color shows that these differentially expressed proteins (DEPs) were underexpressed and red shows overexpression of DEPs in unilateral varicocele group compared to the fertile group. Gradation of color reflects their intensity/ abundance of expression (e.g. brighter the red, the larger the protein expression).

**Figure 7:**
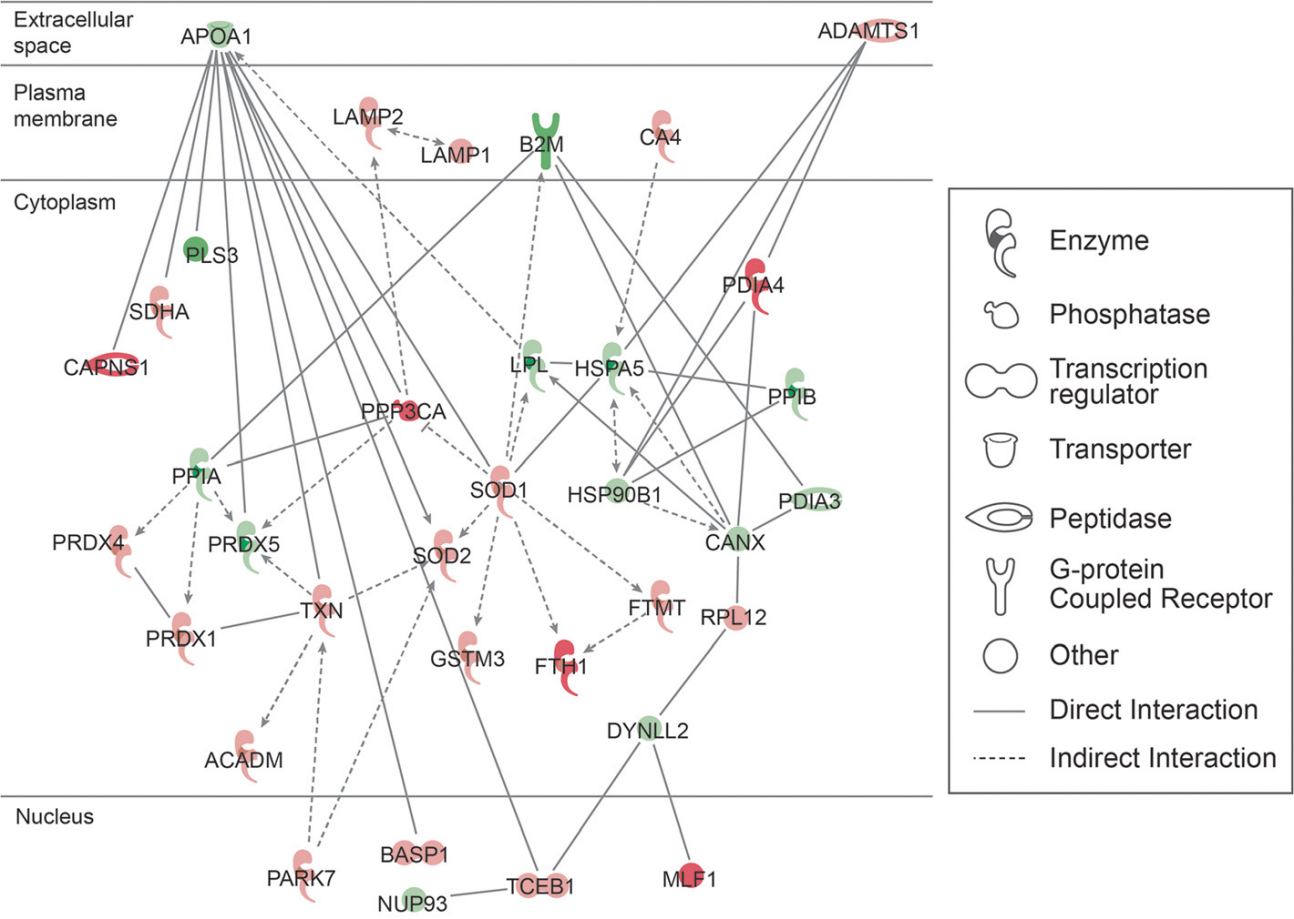
**Top disease and function networks and involvement of differentially expressed proteins in free radical scavenging, neurological disease, skeletal and muscular disorder.** Green color shows that these differentially expressed proteins (DEPs) were underexpressed and red shows overexpression of DEPs in unilateral varicocele group compared to the fertile group. The gradation of color reflects their intensity/ abundance of expression. Gradation of color reflects their intensity/ abundance of expression (e.g. brighter the red, the larger the protein expression).

The top networks involving the DEPs, identified by IPA and DAVID software functional annotations, are shown in Table 4. Networks of interactions of some of the DEP identified in our study and their possible roles in the various functions as well as their subcellular localization are shown in Figures 5, 6 and 7.

**Table 4:** **Ingenuity Pathway Analysis showing top networks, top molecular and cellular functions and top pathways and DAVID’s software annotations**

	**Ingenuity pathway analysis**
Top Networks	Cell death and survival, Metabolic disease, Inflammatory disease (35)
	Nucleic acid metabolism, Small molecule biochemistry, Drug metabolism (35)
	Free radical scavenging, Neurological disease, Skeletal and muscular disorders (35)
	Molecular transport, Protein trafficking, RNA trafficking (20)
Top molecular and cellular functions	Post-translational modification (46), Protein folding (13), Free radical scavenging (34), Nucleic acid metabolism (34), Small molecule biochemistry (82)
Top pathways	Protein ubiquitination pathway (30), Mitochondrial dysfunction (23), TCA cycle (9), Oxidative phosphorylation (13), NRF2-mediated oxidative stress response (14)
	**DAVID’s software functional annotations**
Enriched functional categories	Proteasome-alpha/beta subunit, Threonine protease, Threonine-type endopeptidase activity, regulation of ligase activity, regulation of protein ubiquitination, mitochondrion, aerobic respiration, TCA cycle, glycolysis
Majority of proteins associated with functions	Polymorphism (234), acetylation (170), phosphoprotein (170), cytoplasm (121), mitochondrion (90), cytosol (88), signal (83), hydrolase (78), proteolysis (55), secreted (49), oxidation-reduction (45)
Activated processes/functions^a^	Proteolysis, peptidase activity, Threonine-type endopeptidase, negative regulation of ligase activity
Majority of proteins associated with functions^a^	Acetylation (55), cytoplasm (49), hydrolase (35), peptidase activity (28), secreted (23), mitochondrion (26)
Downregulated processes/functions^b^	Transit peptide, mitochondrion, HSP70, vesicle, stress response, response to unfolded protein
Majority of proteins associated with functions^b^	Acetylation (52), nucleotide binding (36), mitochondrion (29), ATP binding (25), transit peptide (21)
Unique to unilateral varicocele group	
Enriched functional categories	Inflammatory response, defense response, lysosome, ion binding, protease, signal
Unique to fertile group	
Enriched functional categories	Mitochondrial envelope, electron transport, proteasome, regulation of ligase activity, translocation, cell cycle
Functional categories related to reproduction/spermatogenesis	Spermatogenesis (14), spermatid development (5), spermatid differentiation (5), male gamete generation (14), sexual reproduction (18), reproductive process in mitochondrial complex organelle (17)
	Spermatogenesis (6)
	Binding of sperm to zona pellucida (2), Sperm-egg recognition (2), reproductive cellular process (4), sexual reproduction (7)

a = Overexpressed proteins; b = Underexpressed proteins; number in parenthesis are number of identified proteins.

Functional annotations analyzed by David’s software showing different categories and functions.

The top pathways involving the DEPs, as identified by IPA analysis were 1) cell death and survival, metabolic disease, inflammatory disease (Figure 5), 2) nucleic acid metabolism, small molecule biochemistry, drug metabolism (Figure 6), 3) free radical scavenging, neurological disease, skeletal and muscular disorders (Figure 7) and 4) molecular transport, protein trafficking and RNA trafficking. The overexpressed proteins are shown in red and those that are underexpressed are in green. The intensity of the color reflects the level of expression.

35 DEP were identified in the cell death and survival, and metabolic disease (Figure 5). Of these, only 8 were underexpressed, ACAD, ECH1, HSPA8, PFKP, PSMD7, PSMD13, RPLPO and VARS. Twenty seven of the DEP were overexpressed and are shown in pink. Some of the activities that were affected by these DEP were transcription regulation, enzyme activity, and kinase and peptidase activity. Most of the identified DEP were cytoplasmic in distribution. Figure 6 shows 35 DEP in nucleic acid metabolism and small molecule biochemistry. Of these, 14 were underexpressed (shown in green) and 21 were overexpressed (shown in pink). A majority of these DEP were observed to be cytoplasmic in origin. They participate in activities such as enzyme, ion channel, kinase, phosphatase, transcription and translation regulators and transporter function.

Figure 7 shows the involvement of 35 DEP in free radical scavenging, neurological disease, skeletal and muscular disorders in network 3. Of these, 13 were underexpressed and 22 were overexpressed. In this network 3, DEPs were involved in functions related to enzyme activity, phosphatase, transcription regulation, peptidase and the G-protein coupled receptor. Most of DEP were subcellular and cytoplasmic in location.

The top molecular and cellular functions for the DEP included post-translational modification, protein folding, free radical scavenging, nucleic acid metabolism, and small molecule biochemistry. The top pathways included the protein ubiquitination pathway, mitochondrial dysfunction, TCA cycle, oxidative phosphorylation, and NRF2-mediated oxidative stress response. The number of proteins identified with each of these networks, functions and pathways are listed in parenthesis in Table 4.

Similar distributions of DEPs, with respect to functions, networks and locations depicted by DAVID functional annotations are shown in Table 4. This table shows the enriched functional categories and activated processes (proteolysis, peptidase activity, and regulation of ligase activity) relevant to the DEP. Some of the proteins that were overexpressed in the unilateral varicocele group were shown to participate in processes such as proteolysis, peptidase activity, negative regulation of ligases activity or functions such as acetylation, peptidase activity or secretory and mitochondrial activity. While processes such as transit peptides, HSP70, stress response and response to unfolded proteins were underexpressed in DEP in the unilateral varicocele group when compared to the fertile group; the majority of proteins associated with acetylation, nucleotide binding, ATP binding and some transit peptides were overexpressed in the unilateral varicocele group. Some of the enriched functional categories that were unique to the unilateral varicocele group were inflammatory response, defense response, ion binding, protease and signaling. Mitochondrial envelope, electron transport, proteasome, regulation of ligase activity, and translocation were some of the enriched functional categories that were unique to the fertile group.

A key biological function that emerged from the bioinformatics and GO ontology was free radical scavenging. Some of the DEP in unilateral varicocele group including PRDX1, SOD1, SOD2 were shown to be involved in the removal of superoxide. PARK7, PRDX1, SOD1 and SOD2 were related to the quantity of hydrogen peroxide, FTH1, GSRM, PARK7, PRDX1, SOD1 and SOD2 have been demonstrated to be linked to the amount of ROS. Additionally, 10 proteins involved in the production of ROS were identified as: DLST, FBP1, FN1, FTH1, LTF, PARK7, PRDX1, SOD1, SOD2, TXNRD2 while 11 were involved in the synthesis of ROS (DLST, FBP1, FN1, FTH1, IQGAP1, LTF, PARK7, PRDX1, SOD1, SOD2, TXNRD2). Proteins PARK7, PRDX1, SOD1, SOD2, TXNRD2 were involved in metabolism of hydrogen peroxide.

Similarly, 33 proteins were identified to play key roles in cell death. Some of them were ACAA2, ACE, ACLY, ANPEP, BASP1, C1QBP, CA4, DLST, FN1, FTH1, GPI, GSR, HPRT1, LAMP1, LAMP2, LTF, MANF, MME. 19 proteins were seen to be involved in cell survival including ACE, ACLY, FN1, FTH1, GSR, IQGAP1, LTF, PARK7, PPP2CA, PPP2R1A, PPP3CA, PSMA1, PSMA3, PSMA4, PSMA6 and PSMA7. 28 proteins were identified to play a role in necrosis. These were: ACE, ACLY, ANPEP, CA4, DLST, FN1, FTH1, GPI, GSR, HPRT1, LAMP1, LAMP2, LTF, MANF, MME, PARK7, PDCD6IP, PEBP1, PLA2G7, PPP2CA, PPP2R1A, PPP3CA, PRDX1, PSMB1, SDHA, SERPINA3, SOD1 and SOD2.

Furthermore, proteins in the unilateral group that were involved in protein oxidation were FTH1, PA ACADM, CUTC, FBP1, HPRT1, SOD2RK7; SOD1, SOD2 in nitration of proteins; ACADM, CUTC, FBP1, HPRT1, SOD2 in tetramerization of proteins. FN1, LTF, PRDX1, SOD2 were involved in metabolism of hydrogen peroxide; binding of NFKb binding sites; PARK7, PRDX1, SOD1, SOD2, TXNRD2; ACAA2, ACADVL, ACLY, DLD, GOT2, LTF, MDH2, NPC2, PARK7, SDHA, SOD1, SUCLA2 were involved in fatty acid metabolism. These bioinformatics analysis proved useful in the identification of the key functions of those unique proteins present in the unilateral varicocele group. It can be hypothesized that some of these crucial proteins may be responsible for some of the adverse effects of varicocele that is reflected in the function of spermatozoa.

### Identification of differentially expressed proteins relevant to spermatogenesis

Distinct functional categories related to reproduction and/or spermatogenesis was also identified. They included spermatogenesis, spermatid development and differentiation, male gamete generation, binding of sperm to zona pellucida, sperm-egg recognition, reproductive cellular processes and sexual reproduction. Table 5 shows the proteins obtained from UniProt database and STRAP annotation analysis that are likely to play a role in varicocele or male infertility-related function.

**Table 5:** **Proteins associated with fertility related functions obtained from UniProt database and STRAP annotation tool**

**Uniprot No.**	**Gene name**	**Protein name**	**Expression**	**Function**	**References**
O75952	CABYR	Calcium-binding tyrosine phosphorylation-regulated protein isoform c	**↓**	May function as a regulator of both motility- and head-associated functions such as capacitation and the acrosome reaction.	[39]
O75969	AKAP3	A-kinase anchor protein 3	**↓**	May function as a regulator of both motility- and head-associated functions such as capacitation and the acrosome reaction.	[39,40]
P02647	APOA1	Apolipoprotein A-I preproprotein	**↓**	Activates spermatozoa motility.	[41,42]
P04279	SEMG1	Semenogelin-1 preproprotein	**↓**	Constitutes major gel forming proteins in human semen.	[43-47]
P09622	DLD	Dihydrolipoyl dehydrogenase, mitochondrial precursor	**↑**	Lipoamide dehydrogenase is a component of the glycine cleavage system as well as of the alpha-ketoacid dehydrogenase complexes. Involved in the hyperactivation of spermatazoa during capacitation and in the spermatazoa acrosome reaction.	[48-50]
P0C8F1	PATE4	Prostate and testis expressed protein 4	Fertile group only	May modulate the function of nicotinic acetylcholine receptors. May enhance sperm motility.	[51]
P10323	ACR	Acrosin precursor	**↓**	Acrosin is the major protease of mammalian spermatozoa. It is a serine protease of trypsin-like cleavage specificity; proacrosin and stored in the acrosome.	[52,53]
P16562	CRISP2	Cysteine-rich secretory protein 2 precursor	Unilateral varicocele group only	May regulate some ion channels’ activity and thereby regulate calcium fluxes during sperm capacitation.	[54-58]
P21266	GSTM3	Glutathione S-transferase Mu 3	**↑**	Conjugation of reduced glutathione to a wide number of exogenous and endogenous hydrophobic electrophiles. May govern uptake and detoxification of both endogenous compounds and xenobiotics at the testis and brain blood barriers.	[59,60]
P49221	TGM4	Protein-glutamine gamma-glutamyltransferase 4	**↑**	Associated with the mammalian reproductive process. Catalyzes the cross-linking of proteins and the conjugation of polyamines to specific proteins in the seminal tract.	[46]
P56597	NME5	Nucleoside diphosphate kinase homolog 5	Fertile group only	Does not seem to have NDK kinase activity. Confers protection from cell death by Bax and alters the cellular levels of several antioxidant enzymes including Gpx5. May play a role in spermiogenesis by increasing the ability of late-stage spermatids to eliminate reactive oxygen species.	[61]
P61916	NPC2	Epididymal secretory protein E1 precursor	**↑**	Plays important role in exit of cholesterol from endosomal/ lysosomal compartment.	[62,63]
P78540	ARG2	Arginase-2, mitochondrial precursor	Unilateral varicocele group only	Arginase activity negatively associated to sperm concentration and positively with sperm motility.	[64-66]
Q13733	ATP1A4	Sodium/potassium-transporting ATPase subunit alpha-4 isoform 1	**↓**	Essential for germ cell gene expression; sperm with deficiency show bent in the tail, abnormal ion regulation and reduced motility.	[67]
Q15506	SPA17	Sperm surface protein Sp17	**↓**	Sperm surface zona pellucida binding protein. Helps to bind spermatozoa to the zona pellucida with high affinity. Might function in binding zona pellucida and carbohydrates.	[68-71]
Q5BJF6	ODF2	Outer dense fiber protein 2 isoform 3	**↑**	Seems to be a major component of sperm tail outer dense fibers (ODF). ODFs are filamentous structures located on the outside of the axoneme in the midpiece and principal piece of the mammalian sperm tail and may help to maintain the passive elastic structures and elastic recoil of the sperm tail. May have a modulating influence on sperm motility. Functions as a general scaffold protein that is specifically localized at the distal/ sub distal appendages of mother centrioles. Component of the centrosome matrix required for the localization of PLK1 and NIN to the centrosomes. Required for the formation and/or maintenance of normal CETN1 assembly.	[72,73]
Q8IZP9	GPR64	G-protein coupled receptor 64 isoform 2 precursor	**↑**	Could be involved in a signal transduction pathway controlling epididymal function and male fertility.	[74,75]
Q8NCR6	SMRP1	Spermatid-specific manchette-related protein	Fertile group only	May play a role in spermatogenesis. May be involved in nuclear shaping during spermiogenesis and transport of proteins.	[76]
Q8TAA3	PSMA8	Proteasome subunit alpha type-7-like isoform 2	**↑**	Component of the spermatozoa proteasome.	[77-80]
Q8TC29	ENKUR	Enkurin	**↓**	Adapter that functions to localize calcium-sensitive signal transduction machinery in sperm to a calcium-permeable ion channel.	[81]
Q8WWU5	TCP11	T-complex protein 11 homolog isoform 1	Fertile group only	May play an important role in sperm function and fertility.	[82,83]
Q8WYR4	RSPH1	Radial spoke head 1 homolog	**↓**	May play an important role in male meiosis.	[84,85]
Q96A08	HIST1H2BA	Histone H2B type 1-A	**↑**	Variant histone specifically required to direct the transformation of dissociating nucleosomes to protamine in male germ cells. Entirely replaces classical histone H2B prior nucleosome to protamine transition and probably acts as a nucleosome dissociating factor that creates a more dynamic chromatin facilitating the large-scale exchange of histones. Also expressed maternally and is present in the female pronucleus.	[86]
Q96PU9	ODF3	Outer dense fiber protein 3	Fertile group only	Outer dense fibers are filamentous structures located on the outside of the axoneme in the midpiece and principal piece of the mammalian sperm tail. May help to maintain the passive elastic structures and elastic recoil of the sperm tail.	[73,83,87]
Q96QH8	SPACA5	Sperm acrosome-associated protein 5 precursor	Fertile group only	Sperm acrosome-associated protein 5.	[88]
Q99497	PARK7	Protein DJ-1	**↑**	It acts as atypical peroxiredoxin –like peroxidase that scavenges hydrogen peroxide.	[19,89-91]
Q9H1X1	RSPH9	Radial spoke head protein 9 homolog isoform 1	**↓**	Sperm and flagella axonemes. They consist of a thin stalk.	[84]
Q9NQ60	EQTN	Acrosome formation-associated factor isoform 1	Fertile group only	Acrosomal membrane-anchored protein involved in the process of fertilization and in acrosome biogenesis.	[92]
Q9UFH2	DNAH17	Dynein heavy chain 17, axonemal	**↓**	Force generating protein of respiratory cilia. Produces force towards the minus ends of microtubules. Dynein has ATPase activity; the force-producing power stroke is thought to occur on release of ADP. Involved in sperm motility; implicated in sperm flagellar assembly.	[93]

Of the DEP, we identified 29 proteins that were involved in spermatogenesis and other reproductive functions. 11 of these proteins were underexpressed in the unilateral varicocele group. These were calcium-binding tyrosine phosphorylation-regulated protein isoform c (CABYR), A-kinase anchor protein 3 (AKAP3), Apolipoprotein A-I preproprotein (APOPA1), semenogelin-1 preproprotein (SEMG1), acrosin (ACR) precursor, sodium/potassium-transporting ATPase subunit alpha-4 isoform 1 (ATP1A4), sperm surface protein Sp17 (SPA17), Enkurin (ENKUR), radial spoke head 1 homolog (RSPH1), radial spoke head protein 9 homolog isoform 1 (RSPH9) and dynein heavy chain 17 axonemal (DNAH17).

Similarly, the 9 overexpressed proteins in the unilateral varicocele group included dihydrolipoyl dehydrogenase, mitochondrial precursor (DLD), glutathione S-transferase Mu 3 (GSTM3), protein-glutamine gamma-glutamyltransferase 4 (TGM4), epididymal secretory protein E1 precursor (NPC2), outer dense fiber protein 2 isoform 3 (ODF2), G-protein coupled receptor 64 isoform 2 precursor (GPR64), proteasome subunit alpha type-7-like isoform 2 (PSMA8), histone H2B type 1-A (HIST1H2BA) and protein DJ-1 (PARK7) (Table 5). Seven proteins expressed only in the fertile group included prostate and testis expressed protein 4 (PATE4), nucleoside diphosphate kinase homolog 5 (NME5), spermatid-specific manchette-related protein (SMRP1), T-complex protein 11 homolog isoform 1 (TCP11), outer dense fiber protein 3 (ODF3), sperm acrosome associated protein 5 precursor (SPACA5) and acrosome formation associated factor isoform 1 (EQTN). Two proteins that were unique to the varicocele group were cysteine-rich secretory protein 2 precursor (CRISP2) and arginase-2, (ARG2) (Table 5).

## Discussion

To help better delineate possible mechanisms for varicocele and potential biomarkers for evaluating fertility and varicocelectomy candidates, we examined the spermatozoa protein alterations in men with unilateral varicocele and compared them with those of fertile men. In the present study, we observed a significant decline in semen parameters such as concentration, motility, morphology as well as increased production of ROS levels in infertile men with unilateral varicocele. However, semen parameters are poor indicators of the fertilizing ability of spermatozoa.

Mass-spectrometry based proteomic methods coupled with liquid chromatography are widely used. Use of 2-dimensional proteomic maps and liquid chromotography mass spectrometer (LC-MS) analysis, matrix-assisted laser desorption/ionization-time of flight mass spectrometry (Maldi-TOF MS) has greatly helped in the proteomic profiling of mammalian spermatozoa. Several labs prefer the Mudpit method, however, our 1D gel electrophoresis (1-DE) methodology appears to give us better proteome coverage. This may be partly due to the extra clean-up steps the Mudpit analysis strong cation exchange resin (SCX) experiments require over the 1-DE/in gel digestion protocol used by our lab. While these methods certainly have an advantage, these are expensive. The Dionex nano-HPLC coupled to the Orbitrap instrument gives chromatographic reproducibility. Through proteomic profiling, we have provided a comprehensive methodology to help understand the composition and function of the sperm in relation to the unilateral varicocele. We coupled SDS-PAGE for protein fractionation to high resolution LC-MS/MS analysis on an Orbitrap Elite instrument. These techniques consistently deliver high resolution and mass accuracy, reduce the analysis time and increases confidence in the results. While isotopic labeling is a method that allows multiplexing, the number of samples analyzed in the present study were larger than the multiplexing capability of the TMT or iTRAQ reagents.

Since not any one annotation tool or database can provide all the information for all the proteins, functional profiling of proteins was done using a suite of bioinformatics annotation tools and pathway databases and the consensus, consolidated findings have been leveraged to obtain a comprehensive view of the impacted processes/pathways/ functions/networks. Besides showing the perturbed networks, processes and functions based on the protein-protein interactions, the bioinformatics analysis identified leads (that about 29 proteins were actually associated with reproductive functions such as motility, hyperactivation, acrosome reaction, etc.) for experimental validation and help generate hypothesis for future work.

Our genome-wide protein profiling analysis found more than 1000 proteins in the control group and nearly 750 in the unilateral varicocele group. Of these, 369 were differentially expressed (over/underexpressed or uniquely expressed) in either group. We also classified the proteins according to their abundance (high, medium, low or very low) (Figure 2A-B). For the most part, proteins specific to the unilateral varicocele group were expressed at high and medium levels suggestive of a role in the pathology of the disease. Most of the DEP were intracellular proteins (Table 1; Figure 3A). This also suggests their cellular purity because these proteins largely originate from the spermatozoa, illustrating that there was negligible contribution from the seminal plasma proteins.

Most of the DEP participating in various biological processes are shown in (Table 2, Figure 3B). Changes in the expression of these proteins will affect their role in biological processes like small molecule metabolic process, cellular nitrogen compound metabolic processes, response to stress and signal transduction processes. We found that the major molecular functions of the DEP consisted of ion binding, oxidoreductase activity, peptidase activity, enzyme binding and enzyme regulator activity (Table 3, Figure 3C).

Therefore, it is presumed that alterations in the expression level of these proteins could affect enzyme kinetics. It could also be presumed that altered proteins in varicocele patients, responsible for ion binding and oxidoreductase activity, will cause changes in cell defense against oxidative stress or heat stress. Previous studies have reported that oxidative stress caused by high ROS levels resulted in the presence of 17 precursor proteins [21]. Likewise, it could be presumed that changes in the expression level of proteins involved in peptidase activity may affect- catabolic reactions and in turn, affect metabolic functions. Increased peptidase activity may be indicative of increased apoptotic activity. Changes in enzyme regulatory activity could also affect enzyme-substrate affinity in the cell.

The Reactome software analysis categorized 97% (359 of the 369) of the DEP into functional pathways. The major pathways related to the unilateral varicocele group involve metabolism, disease, immune system, gene expression, signal transduction and apoptosis (Figure 4). IPA and DAVID functional annotations showed that small molecule biochemistry and post-translational modification proteins were mostly affected as a result of the unilateral varicocele.

The proteins in molecular interaction networks (Figures 5 and 6) show that majority of DEP were intracellular as shown in Table 1; Figure 3A. Some of the top biological functions in the proteins identified in the unilateral varicocele group were not limited to spermatozoa function but are also present in many other cell types such as in disease, cell viability, necrosis, apoptosis, cytotoxicity, free radical metabolism, protein oxidation, nitration and tetramerization and metabolism of aldehydes, fatty acid, hydrogen peroxide, metabolism of acetyl and acyl-coenzyme A.

### Proteins involved in reproductive functions

Of the 369 DEPs, 29 proteins were involved in reproductive functions such as motility, capacitation, hyperactivation, acrosome reaction and zona-pellucida binding. Some of the proteins along with their reproductive functions are described below:

CABYR along with AKAP3 seem to associate in high molecular weight multi-protein complexes, which regulate the sperm flagella’s energy supply and movements [39,40]. We found this protein to be underexpressed and present in low abundance in the unilateral varicocele patient group when compared with the healthy fertile men. The reduced expression of CABYR in varicocele patients is suggestive of its involvement in poor sperm motility seen in these patients, as low CABYR would entail impaired energy supply to the flagella and consequently spermatozoa movement.

We found that AKAP3 was underexpressed in the unilateral varicocele patients but present in high abundance. As a regulator of sperm motility, the reduced expression of AKAP3 in varicocele patients could contribute to the poor sperm motility seen in these patients [40]. Further, any impairment on the formation of the mitochondrial membrane during spermatogenesis would account for the low sperm count in varicocele patients.

APOA1 activates spermatozoa motility [41,42]. We found that APOA1 preprotein was underexpressed in the unilateral varicocele patient group and showed very low abundance. The low expression of APOA1 is indicative of its possible role in sperm motility, as shown by the poor sperm motility in varicocele patients.

SEMG1 preprotein was underexpressed in the unilateral varicocele patients with a high abundance when compared with the healthy fertile men. Both SEMG1 and SEMG2 constitute the major gel-forming proteins in human semen [43-45]. Inhibition of sperm motility and premature activation of capacitation are the two main functions of SEMG. It addition to tackling oxidative stress it also helps reduce the generation of free radicals by slowing motility to reduce energy consumption as well as by binding to high amounts of zinc that has antioxidant and anti-capacitating effects. In 19 adolescent boys (15–19 years) with Grade II or III varicocele (17 bilateral, 2 unilateral), SEMG 1 and 2 were found only to be expressed in samples taken 3 months post-microsurgical varicocelectomy, but not prior to surgical intervention [46]. As sperm motility and percentage sperm with normal morphology was significantly improved after varicocelectomy, it could be inferred that the lack of SEMG proteins contribute to the poor motility and morphology as seen in varicocele patients. Contrary to our finding, expression of seminal plasma SEMG1 was low in control and varicocele with normal semen quality but high in the adolescent varicocele with abnormal semen quality reflecting a strategy to counteract the deleterious effects of oxidative stress resulting from high levels of ROS and lipid peroxidation. Furthermore, increased levels of SEMG found in the seminal plasma of varicocele with abnormal semen quality may lead to reduced fluidity, premature sperm capacitation and strong inhibition in sperm motility observed in their study [47]. We also reported the presence of SEMG I preprotein overabundance in ROS negative samples in our earlier study [19].

DLD is a mitochondrial precursor that acts as a pro-oxidant when it reduces oxygen to superoxide and catalyzes the production of hydroxyl radicals [48]. It is involved in hyperactivation, capacitation and acrosome reaction. DLD also has antioxidant regenerating properties when it acts as a diaphorase, to scavenge nitric oxide and reduce ubiquinone to ubiquinol [49]. We found that DLD precursor was overexpressed in the unilateral varicocele patients and present in a high abundance. This suggests any increase in the mitochondrial enzyme activity may contribute towards the production of oxidative stress, as seen in varicocele patients. Contrary to our study, Soares et al. reported decreased DLD in experimentally induced varicocele in rats [50].

PATE4 was uniquely expressed in the healthy fertile group and absent in varicocele patients. The absence of PATE4 in varicocele patients emphasizes the role of PATE4 in enhancing sperm motility [51], the absence of which could cause the poor sperm motility in varicocele patients. Acrosin activity is involved in the binding of the sperm to the zona pellucida [52,53]. We found acrosin precursor to be underexpressed in the unilateral varicocele patients and present in very low abundance. The reduced expression of acrosin precursor suggests the impairment of the spermatogenesis process in varicocele patients.

CRISP is localized in the testis in an androgen-independent manner and expressed in the acrosome of the round spermatids and in the sperm tail [54-56]. Following acrosome reaction, CRISP2 remains located in the fusogenic region of the sperm head, which suggests its role in fertilization [56,57]. Testicular CRISP2 is involved in sperm-egg fusion, where it acts in co-operation with CRISP1 [56]. Testicular CRISP2 has a role in germ cell-Sertoli cell adhesions. The human CRISP2 gene is reported to occur at a location associated with translocations and male infertility [58]. We found that CRISP2, although in low abundance, was uniquely expressed in the unilateral varicocele group. It’s presence in varicocele patients only but absence in the fertile controls suggests of its involvement in the infertility seen in some males with varicocele. GSTM3 is involved in detoxification of endogenous compounds and xenobiotics from the testis [59,60]. GSTM3 was overexpressed in the unilateral varicocele group and present in a high abundance. The increase in GSTM3 protein expression suggests its increased role in the detoxification in varicocele patients.

TGM4 catalyzes the cross-linking of proteins and the conjugation of polyamines to specific proteins in the seminal tract. They also cross-link with semenogelin. Their regulation is therefore important to avoid clot formation. Our data shows that TGM4 was overexpressed with high abundance in the unilateral varicocele group. However, in Del Guidice’s study, TGM4 was found only to be expressed in samples after varicocelectomy, but not prior to varicocele removal [46]. It could be inferred that TGM4 may be associated with homeostasis in varicocele patients.

NME 5 was uniquely expressed with medium abundance in the fertile control group. Patients with varicocele are at a disadvantage as the absence of NME 5 signifies the lack of protection from apoptosis and the increase in antioxidants levels [61], which will contribute to oxidative stress and poor sperm parameters in varicocele patients. NPC precursor is an intracellular cholesterol transporter that acts in concert with Niemann-Pick disease, type C (NPC2) and plays an important role in the exit of cholesterol from the endosomal/lysosomal compartment [62,63]. Our data indicates that NPC2 was overexpressed with medium abundance in the unilateral varicocele group suggesting alterations in membrane fluidity, capacitation and acrosome reaction in these patients.

ARG2 was uniquely expressed with low abundance in the unilateral varicocele group. In an earlier study, Elgun et al. found that arginase activity in sperm cells from oligozoospermic infertile men was higher compared to that of healthy fertile fertile men, although arginase activity in seminal plasma did not differ between the two groups studied. Further, arginase activity was negatively correlated to sperm count, but positively correlated to sperm motility. The authors suggested that the increase in arginase activity may upset the arginine-nitric oxide pathway within the spermatozoa of oligospermic infertile men, compromising sperm function and leading to infertility [64,65]. Our data shows the unique expression of ARG2 in varicocele men may be indicative of the poor sperm parameters seen in varicocele patients. High levels of nitric oxide, a free radical, have a negative impact on sperm quality. Also, increased arginase activity leads to lower arginine content in sperm plasma, as seen in men with abnormal semen parameters compared to controls [66].

ATP1A4 is the catalytic component of the active enzyme, which catalyzes the hydrolysis of ATP coupled with the exchange of sodium and potassium ions across the plasma membrane. Rodova et al. showed that ATP1A4 was essential for male germ cell gene expression [67,77]. We found this protein to be underexpressed with very low abundance in unilateral varicocele group; spermatozoa with deficiency of this protein show a characteristic bend in the sperm flagellum which is indicative of abnormal ion regulation, reduced motility and hyperactivation which is essential for capacitation. This finding may indicate poor motility which was also seen in our unilateral varicocele group.

SPA17 is present in the different stages of sperm maturation, such as spermatocytes, spermatids and spermatozoa but not spermatogonia [68,69]. SpA17 is localized throughout the principle piece of the spermatozoa flagellum (the flagellar fibrous sheath). It was also found to be present *in vitro* from ejaculation to oocyte fertilization, indicating its likely involvement in the regulation of sperm maturation, capacitation, acrosomal reaction and sperm-oocyte zona pellucida binding during fertilization [70]. Further, SPA17 may have a regulatory role in a protein kinase A-independent AKAP complex in germinal and somatic cells. Testicular SPA17 expression increases greatly as development progresses, indicating its major contribution to sperm function [71]. We found that sperm surface protein SPA17 was underexpressed with very low abundance in the unilateral varicocele group. This could explain why varicocele patients have poor sperm concentration and motility.

ODF2 is a major component of sperm tail and are located of the axoneme in the mid piece of the sperm tail. It helps maintain the passive elastic structures and the elastic recoil of the sperm tail [72,73]. ODF2 was overexpressed with high abundance in the unilateral varicocele group. GPR64 are involved in the regulation of cAMP and unregulated production of cAMP in capacitated spermatozoa can lead to spontaneous acrosome loss [74,75]. We found GPR64 precursor to be overexpressed with medium abundance in unilateral varicocele group. Overexpression could indicate that some of the spermatozoa in unilateral varicocele group may have already undergone spontaneous acrosome reaction and may no longer be able to fertilize. SMRP1 is involved in nuclear shaping of sperm head during spermiogenesis [76]. Its complete absence in the unilateral varicocele group suggests that some of the sperm may not undergo complete nuclear compaction and may have defective sperm DNA.

PSMA8 is specifically found in the testis and promotes degradation of histones, thereby participating actively in the exchange of histones during spermatogenesis. Du et al. reported that it plays an important role in the regulation of cell proliferation or cell cycle control, transcriptional regulation, immune and stress response, cell differentiation, and apoptosis [77]. Proteasomes are linked with various activities such as motility, acrosome reaction and fertilization [78]. A recent study also implicated proteasomes in capacitation [79]. Sperm proteasome studies in asthenozoospermic samples have shown the differential expression of proteasome in sperm motility. Zhao et al. showed that the 267S protease regulatory subunit 7 was decreased in in asthenozoospermic samples [80]. Our data showed that overexpression of PSMA8 in the unilateral varicocele group indicates the involvement in altered motility and a compromised ability to undergo acrosome reaction.

ENKU is involved in the maintenance of Ca^2+^ ion channels. It is located in the head (acrosome) and the principle piece of the sperm. ENKUR is thought to be a calmodulin-binding protein. It is involved in acrosome reaction. By interacting with TRPC channels (canonical transient receptor potential channels) involved in signal transduction within sperm cells, ENKUR is integral to sperm motility [81]. We reported low expression of ENKUR in the unilateral varicocele group emphasizing the reason for poor motility exhibited in semen samples from infertile men with unilateral varicocele. T-complex protein 11 homolog isoform 1 (TCP11) could play an important role in the regulation of human sperm function [82]. Its presence in the testis indicates its role in spermatogenesis and sperm function. We found low abundance of TCP11 uniquely expressed in the fertile control group only but in low abundance. Lower expression was demonstrated in spermatozoa with poor morphology compared to sperm samples with normal morphology [83]. Higher incidence of sperm coiling is seen in infertile men compared to fertile men and this can compromise the fertilizing capacity in infertile men. RSPH1 may play an important role in meiosis [84,85]. We found RSPH1 underexpressed with low abundance in the unilateral varicocele group.

Infertile men have a higher proportion of spermatozoa with histone variant H2B (HIST1H2BA) than fertile men [86]. Our data showed overexpression of HIST1H2BA in the unilateral varicocele group suggesting defects in histone replacement in spermatozoa from unilateral men that may be responsible for defective chromatin packaging and increased DNA fragmentation in the spermatozoa of these men due to a relative increase in the histone to protamine ratio.

ODF contribute to the distinct morphology and function of the sperm tail. TCP11 interacts with ODF suggesting that TXCP 11 and are responsible for normal sperm tail morphology and motility [73,83,87]. We found ODF3 was expressed only in the fertile control group indicating higher percentage of normal sperm morphology with minimal tail coiling in sperm from fertile men.

Korfanty et al. suggested that SPACA5 is a lysozyme like protein released during acrosome reaction and is involved in fertilization [88]. Low expression of SPACA5 precursor only in the fertile control group suggests that the active form is expressed and responsible for the acrosome reaction and fertilization process.

PARK7 acts as an atypical peroxiredoxin-like peroxidase that scavenges hydrogen peroxide. Following removal of a C-terminal peptide, it displays protease activity and enhanced cytoprotective action against oxidative stress-induced apoptosis. An et al. [89] showed that PARK7 concentration was positively correlated with sperm motility and sperm SOD activity. We found that PARK7 was overexpressed with low abundance in the unilateral varicocele group. Varicocele-associated infertility is mediated by oxidative stress [19,90]. PARK7 has an oxidative stress reducing capacity [91]. Overexpression of PARK7 seen in unilateral varicocele patients may be a mechanism to control and protect against oxidative stress effects.

RSPH9 is a component of the axonemal radial spoke head [84]. In our study, we found that RSPH9 was underexpressed in the unilateral varicocele group. This could explain abnormalities of sperm tails observed in the sperm with abnormal morphology in varicocele patients, and their subsequent reduction in fertility. Afaf 1 or EQTN is highly expressed in the testis. It is involved in calcium-triggered acrosome reaction and participates in fertilization [92]. The unique expression of EQTN in the fertile control group suggests its active role in acrosome reaction. DNAH17is involved in sperm motility [93]. We found that this protein was underexpressed in the unilateral varicocele group. The low expression may be responsible for poor motility seen in infertile men with varicocele and especially in those with unilateral varicocele. The majority of the DEP involved in motility and abnormal morphology, acrosome reaction, capacitation and chromatin packaging may explain the poor semen parameters such as reduced motility and abnormal morphology observed in the semen of the unilateral varicocele patients.

We compared the proteins reported by Hosseinifar’s group who characterized the protein profile of 20 normospermic men without varicocele and 20 sperm samples from oligozoospermic men with grade 3 varicocele using 2-dimensional gel electrophoresis (2DE) [22]. These authors identified 15 distinct proteins in the varicocele group compared with the control group. Of these 10 were also identified in our study although not all were differentially expressed. ACPP was overexpressed in the varicocele study whereas in our study it was underexpressed but not differentially expressed as it did not meet the fold change requirement that we set for identifying the High abundance protein. Similarly ATP5D was underexpressed in their study but was overexpressed in our study but was not differentially expressed as it did not meet the p value requirement for high abundant protein. CLU and KLK was underexpressed but not differentially expressed in our study as it did not meet the fold requirement for the High abundance protein. HSPA5 was underexpressed and differentially expressed in both studies. KLK3, PIP, and SEMG2 precursor were underexpressed in both studies but not differentially expressed in our study as they failed to meet the fold change requirement set in our study. PARK 7 and SOD1 were differentially expressed in both studies however both were overexpressed in our study. The reverse trend in the expression of DEP in our study may be related to the fact that we examined only the unilateral varicocele and also the techniques used as well as the cutoff for protein abundance was different and this could explain the difference in expression of these proteins in our study.

138 of the differentially expressed proteins were unique to the fertile group and only 38 were unique to the unilateral group. This is an important finding which indicates that there are major protein alterations that are responsible for spermatogenesis, acquisition of fertilizing capacity such as motility/ flagellar movement, hyperactivation, capacitation and acrosome reaction that are significantly altered in these men. These alterations may be responsible for the underlying pathology of infertility seen in these men.

### Identification of candidate proteins as potential biomarkers based on peptide coverage

Based on the number of peptides and peptide coverage number in the triple run, we narrowed down the choice of potential candidate proteins that could be identified as our first choice of potential biomarkers in identifying unilateral varicoceles associated with male infertility (Table 5). Nine candidate proteins were identified based on greater than 60% peptide coverage in either group. CABYR, SEMG-1 preproprotein, RSPH1and SPA17 were underexpressed in the unilateral varicocele group. Four proteins that were overexpressed in the unilateral group were GSTM3, DLD, PSMA8 and PARK7. NME5 was expressed only in the fertile group.

The second choice candidates based on peptide coverage between 50% – 60% were AKAP3 and RSPH1 which were underexpressed in the unilateral group, and HIS1H2BA which was overexpressed in the unilateral group. ODF3 was present only in the fertile group. The other potential candidates are listed in Table 5. We feel that many of these proteins, especially those identified as moderately abundant in one of the samples, may indeed be a DEP. All proteins identified as DEP that are of interest for any down-stream applications, i.e. biomarker analysis, need to be validated with either western, ELISA, or stable isotope dilution LC-MS. We did not examine the effect of grade on the downstream markers due to the sample size limitation. However, examination of the proteome profiles of men with different varicocele grades are among our planned future studies. Fertile group had approximately 30% more proteins, corresponding to an average of 279 than the varicocele group and may be due to the increased presence of very low abundance proteins in the unilateral varicocele group. Another plausible explanation for the lower number of proteins identified in the unilateral group with varicocele may be an indication of the potential defects in the transcriptome. The onset and progression of varicocele disease may not only affect the process of spermatogenesis in terms of the number and quality of spermatozoa, but may also affect the changes occurring in the molecular mechanism underlying protein alterations and may be indicative of the possible defects in the sperm transcriptome.

## Conclusions

In summary, the present study identified proteins that are exclusively expressed in men with unilateral varicocele. CABYR, AKAP, APOPA1, SEMG1, ACR, SPA17, RSPH1, RSPH9 and DNAH17 underexpressed in the unilateral varicocele group are associated with the reproductive and fertilizing ability of spermatozoa. DLD, GSTM3, TGM4, NPC23, ODF2GPR64, PSM8, HIST1H2BA and PARK7 were overexpressed in the unilateral varicocele group suggesting that sperm quality and functional capacity was abnormally affected.

In this study, we have examined the protein profile and shortlisted some proteins that may play a key role in the progression of the disease. This is by no means an answer to the identification of select biomarkers of varicocele and a more elaborate study with larger sample size and defined parameters such as the grade of varicocele etc. are necessary. Furthermore, the shortlisted protein markers need to be validated. However, this is a novel study as it examines the effect of unilateral varicocele on the onset of infertility and attempts to identify the underlying alteration in the protein profile in addition to oxidative stress being identified as an etiology of varicocele. By implementing proteomics and functional bioinformatics analysis we have provided a list of putative targets that further needs to be experimentally validated before classifying them as potential biomarker(s) in diagnosing the disease as well as using them to develop an algorithm to determine which men are more likely to benefit from varicocelectomy.

## Additional files

Additional file 1: Table S1.
**Global proteomic profiling of fertile control group in triplicate - first run - gel 1.**
Additional file 2: Table S2.
**Global proteomic profiling of fertile control group in triplicate - second run - gel 2.**
Additional file 3: Table S3.
**Global proteomic profiling of fertile control group in triplicate - third run - gel 3.**
Additiona file 4: Table S4.
**Global proteomic profiling of unilateral varicocele group in triplicate - first run - gel 1.**
Additional file 5: Table S5.
**Global proteomic profiling of unilateral varicocele group in triplicate - second run - gel 2.**
Additional file 6: Table S6.
**Global proteomic profiling of unilateral varicocele group in triplicate - third run - gel 3.0.**

## Abbreviations

1-D SDS-PAGE: One dimensional sodium dodecyl polyacrylamide gel electrophoresis
2DE: 2-dimensional gel electrophoresis
ANOVA: Analysis of variance
ART: Assisted reproduction technology
ASRM: American Society for Reproductive Medicine
BCA: Bicinchoninic acid
DAVID: Database for annotation, visualization and integrated discovery
DEP: Differentially expressed proteins
DNA: Deoxyribonucleic acid
DTT: Dithiothreitol
GSH: Glutathione
GPx: Glutathione peroxidase
GR: Glutathione reductase
GST: Glutathione-S transferase
GSSG: Gluthathione disulphide
IVF: *In vitro* fertilization
IVF-ET: *In vitro* fertilization-embryo transfer
ICSI: Intracytoplasmic sperm injection
FITC-dUTP: Fluorescein isothiocynate–tagged deoxyuridine triphosphate nucleotides
FPR: False positive rate
GO: Gene Ontology
H_2_O_2_: Hydrogen peroxide
HPLC: High performance liquid chromatography
IPA: Ingenuity Pathway Analysis
iTRAQ: Isobaric tags for relative and absolute quantitation
LC-MS/MS: Liquid chromatography tandem mass spectroscopy
LC-MS: Liquid chromotography mass spectrometer analysis
Maldi-TOF MS: Matrix-assisted laser desorption/ionization-time of flight mass spectrometry
MS/MS: Tandem mass spectra
NCBI: National Center for Biotechnology Information
NSAF: Normalized spectral abundance factor
OAT: Oligoasthenoteratozoospermia
PCOS: Polycystic ovarian syndrome
PBS: Phosphate buffer solution
PPM: Parts per million
RCTs: Randomized controlled trials
RIPA: Radio-immunoprecipitation assay
RLU: Relative light units
ROS: Reactive oxygen species
SCX: Strong cation exchange resin
SOD: Superoxide dismutase
SpCs: Spectral counts
STRAP: Annotation analysis
TAC: Total antioxidant capacity
TMT: Tandem Mass Tag
TUNEL: Terminal deoxynucleotidyl transferase–mediated fluorescein end labelling terminal deoxytransferase (TdT) enzyme
WHO: World Health Organization
