# Supplementary material for: Major protein alterations in spermatozoa from infertile men with unilateral varicocele
Source: Reprod Biol Endocrinol. 2015 Feb 22;13:8. doi: 10.1186/s12958-015-0007-2 (PMC4383193; doi:10.1186/s12958-015-0007-2)
Supplement: Additional file 1: Table S1. — Global proteomic profiling of fertile control group in triplicate - first run - gel 1. [file 12958_2015_7_MOESM1_ESM.docx]

**Supplemental Table 1. Global proteomic profiling of fertile control group in triplicate - first run – gel 1.**

| **No.** | **Gel 1** | | | | | |
| --- | --- | --- | --- | --- | --- | --- |
|  | **Protein** | **Accession No.** | **Mass** | **Peptides** | **Sequence** | **Spectral** |
|  |  |  | **kDa** | **No.** | **Cov (%)** | **Counts** |
| 1 | semenogelin-2 precursor | 4506885 | 65 | 62 | 62% | 1803 |
| 2 | semenogelin-1 preproprotein | 4506883 | 52 | 72 | 71% | 1588 |
| 3 | lactotransferrin isoform 1 precursor | 54607120 | 78 | 74 | 76% | 1308 |
| 4 | A-kinase anchor protein 4 isoform 2 | 21493039 | 93 | 71 | 72% | 1069 |
| 5 | tubulin beta-4B chain | 5174735 | 50 | 39 | 74% | 922 |
| 6 | fibronectin isoform 3 preproprotein | 16933542 | 259 | 112 | 48% | 875 |
| 7 | heat shock-related 70 protein 2 | 13676857 | 70 | 48 | 72% | 727 |
| 8 | tubulin alpha-3C/D chain | 17921993 | 50 | 34 | 75% | 638 |
| 9 | heat shock protein HSP 90-alpha isoform 1 | 153792590 | 98 | 59 | 53% | 612 |
| 10 | A-kinase anchor protein 3 | 21493041 | 95 | 58 | 56% | 567 |
| 11 | actin, cytoplasmic 1 | 4501885 | 42 | 34 | 85% | 564 |
| 12 | 78 glucose-regulated protein precursor | 16507237 | 72 | 38 | 55% | 544 |
| 13 | prolactin-inducible protein precursor | 4505821 | 17 | 17 | 77% | 540 |
| 14 | clusterin preproprotein | 355594753 | 52 | 28 | 43% | 515 |
| 15 | keratin, type II cytoskeletal 1 | 119395750 | 66 | 44 | 53% | 494 |
| 16 | endoplasmin precursor | 4507677 | 92 | 55 | 55% | 473 |
| 17 | heat shock cognate 71 protein isoform 1 | 5729877 | 71 | 18 | 43% | 447 |
| 18 | keratin, type I cytoskeletal 9 | 55956899 | 62 | 41 | 62% | 446 |
| 19 | pyruvate kinase isozymes M1/M2 isoform c | 332164775 | 66 | 42 | 64% | 412 |
| 20 | ATP synthase subunit beta, mitochondrial precursor | 32189394 | 57 | 30 | 74% | 389 |
| 21 | heat shock protein HSP 90-beta | 20149594 | 83 | 23 | 47% | 385 |
| 22 | prostatic acid phosphatase isoform TM-PAP precursor | 197116348 | 48 | 22 | 44% | 366 |
| 23 | heat shock 70 protein 1-like | 124256496 | 70 | 28 | 59% | 350 |
| 24 | protein disulfide-isomerase A3 precursor | 21361657 | 57 | 36 | 65% | 340 |
| 25 | tubulin alpha-1A chain | 17986283 | 50 | 5 | 64% | 340 |
| 26 | alpha-enolase isoform 1 | 4503571 | 47 | 35 | 77% | 337 |
| 27 | heat shock 70 protein 1A/1B | 194248072 | 70 | 17 | 49% | 326 |
| 28 | tubulin beta-4A chain | 21361322 | 50 | 2 | 61% | 322 |
| 29 | keratin, type I cytoskeletal 10 | 195972866 | 59 | 33 | 44% | 301 |
| 30 | keratin, type II cytoskeletal 2 epidermal | 47132620 | 65 | 30 | 57% | 276 |
| 31 | fructose-bisphosphate aldolase A isoform 2 | 342187211 | 45 | 34 | 76% | 263 |
| 32 | hypoxia up-regulated protein 1 precursor | 5453832 | 111 | 48 | 45% | 258 |
| 33 | outer dense fiber protein 2 isoform 3 | 310750406 | 81 | 30 | 48% | 255 |
| 34 | pyruvate kinase isozymes M1/M2 isoform a | 33286418 | 58 | 2 | 76% | 249 |
| 35 | prostate-specific antigen isoform 1 preproprotein | 4502173 | 29 | 17 | 84% | 241 |
| 36 | calcium-binding tyrosine phosphorylation-regulated protein isoform c | 24797112 | 41 | 23 | 59% | 238 |
| 37 | glyceraldehyde-3-phosphate dehydrogenase, testis-specific | 7657116 | 45 | 20 | 75% | 236 |
| 38 | elongation factor 1-alpha 1 | 4503471 | 50 | 26 | 54% | 231 |
| 39 | calcium-binding tyrosine phosphorylation-regulated protein isoform a | 24797108 | 53 | 6 | 43% | 229 |
| 40 | tubulin beta chain | 29788785 | 50 | 2 | 61% | 229 |
| 41 | tubulin beta-6 chain | 14210536 | 50 | 3 | 19% | 228 |
| 42 | aminopeptidase N precursor | 157266300 | 110 | 50 | 41% | 226 |
| 43 | keratin, type I cytoskeletal 13 isoform a | 131412225 | 50 | 28 | 59% | 226 |
| 44 | ropporin-1A | 21359920 | 24 | 13 | 69% | 220 |
| 45 | plastin-2 | 167614506 | 70 | 41 | 78% | 220 |
| 46 | myosin-9 | 12667788 | 227 | 68 | 38% | 216 |
| 47 | triosephosphate isomerase isoform 2 | 226529917 | 31 | 24 | 94% | 214 |
| 48 | ruvB-like 2 | 5730023 | 51 | 35 | 76% | 212 |
| 49 | tubulin beta-3 chain isoform 1 | 50592996 | 50 | 2 | 32% | 204 |
| 50 | leucine-rich repeat-containing protein 37B precursor | 53829385 | 106 | 28 | 29% | 203 |
| 51 | ATP synthase subunit alpha, mitochondrial precursor | 4757810 | 60 | 31 | 58% | 202 |
| 52 | tubulin alpha-1C chain | 14389309 | 50 | 2 | 61% | 200 |
| 53 | 60 heat shock protein, mitochondrial | 31542947 | 61 | 37 | 76% | 190 |
| 54 | transitional endoplasmic reticulum ATPase | 6005942 | 89 | 37 | 51% | 189 |
| 55 | fatty acid synthase | 41872631 | 273 | 42 | 16% | 188 |
| 56 | acetyl-CoA acetyltransferase, mitochondrial precursor | 4557237 | 45 | 25 | 53% | 188 |
| 57 | malate dehydrogenase, mitochondrial precursor | 21735621 | 36 | 21 | 65% | 187 |
| 58 | T-complex protein 1 subunit beta isoform 1 | 5453603 | 57 | 33 | 67% | 183 |
| 59 | creatine kinase B-type | 21536286 | 43 | 18 | 65% | 172 |
| 60 | angiotensin-converting enzyme isoform 1 precursor | 4503273 | 150 | 27 | 23% | 171 |
| 61 | keratin, type II cytoskeletal 5 | 119395754 | 62 | 19 | 32% | 171 |
| 62 | keratin, type II cytoskeletal 6A | 5031839 | 60 | 13 | 45% | 171 |
| 63 | phosphoglycerate kinase 2 | 31543397 | 45 | 26 | 75% | 163 |
| 64 | annexin A5 | 4502107 | 36 | 24 | 79% | 163 |
| 65 | glutathione S-transferase Mu 3 | 23065552 | 27 | 21 | 77% | 161 |
| 66 | heat shock protein beta-1 | 4504517 | 23 | 18 | 87% | 160 |
| 67 | fumarate hydratase, mitochondrial | 19743875 | 55 | 22 | 61% | 159 |
| 68 | ruvB-like 1 | 4506753 | 50 | 27 | 70% | 159 |
| 69 | acrosin-binding protein precursor | 17999524 | 61 | 23 | 43% | 158 |
| 70 | hexokinase-1 isoform HKI-ta/tb | 15991831 | 103 | 35 | 37% | 155 |
| 71 | annexin A1 | 4502101 | 39 | 23 | 64% | 154 |
| 72 | zona pellucida-binding protein 1 isoform 1 precursor | 229577313 | 40 | 18 | 60% | 154 |
| 73 | annexin A2 isoform 1 | 50845388 | 40 | 25 | 66% | 153 |
| 74 | clathrin heavy chain 1 | 4758012 | 192 | 40 | 25% | 153 |
| 75 | nuclear pore membrane glycoprotein 210-like isoform 1 precursor | 117414168 | 211 | 50 | 32% | 149 |
| 76 | uncharacterized protein C1orf56 precursor | 20149646 | 37 | 14 | 53% | 147 |
| 77 | T-complex protein 1 subunit gamma isoform a | 63162572 | 61 | 28 | 69% | 145 |
| 78 | glyceraldehyde-3-phosphate dehydrogenase | 7669492 | 36 | 16 | 66% | 143 |
| 79 | leucine-rich repeat-containing protein 37A precursor | 289547512 | 188 | 23 | 14% | 143 |
| 80 | neutral alpha-glucosidase AB isoform 2 precursor | 38202257 | 107 | 32 | 30% | 141 |
| 81 | ropporin-1B | 59891409 | 24 | 5 | 85% | 138 |
| 82 | aconitate hydratase, mitochondrial precursor | 4501867 | 85 | 25 | 39% | 137 |
| 83 | cathelicidin antimicrobial peptide preproprotein | 348041314 | 20 | 13 | 51% | 132 |
| 84 | serum albumin preproprotein | 4502027 | 69 | 30 | 51% | 131 |
| 85 | sperm acrosome membrane-associated protein 1 precursor | 13569934 | 32 | 8 | 33% | 131 |
| 86 | ras-related protein Rab-2A isoform a | 4506365 | 24 | 16 | 73% | 131 |
| 87 | sorbitol dehydrogenase | 156627571 | 38 | 17 | 50% | 125 |
| 88 | cytochrome b-c1 complex subunit 2, mitochondrial precursor | 50592988 | 48 | 21 | 54% | 124 |
| 89 | radial spoke head protein 6 homolog A | 13540559 | 81 | 16 | 32% | 123 |
| 90 | dipeptidyl peptidase 4 | 18765694 | 88 | 30 | 36% | 121 |
| 91 | keratin, type II cytoskeletal 4 | 331999954 | 56 | 24 | 51% | 120 |
| 92 | calreticulin precursor | 4757900 | 48 | 21 | 69% | 119 |
| 93 | 14-3-3 protein epsilon | 5803225 | 29 | 20 | 62% | 118 |
| 94 | long-chain-fatty-acid--CoA ligase 1 | 40807491 | 78 | 24 | 37% | 118 |
| 95 | succinate dehydrogenase [ubiquinone] flavoprotein subunit, mitochondrial | 156416003 | 73 | 26 | 57% | 117 |
| 96 | peroxiredoxin-6 | 4758638 | 25 | 15 | 72% | 115 |
| 97 | trifunctional enzyme subunit beta, mitochondrial precursor | 4504327 | 51 | 26 | 54% | 112 |
| 98 | L-lactate dehydrogenase C chain | 4504973 | 36 | 24 | 75% | 111 |
| 99 | T-complex protein 1 subunit delta | 38455427 | 58 | 22 | 51% | 111 |
| 100 | elongation factor 2 | 4503483 | 95 | 31 | 29% | 110 |
| 101 | elongation factor 1-gamma | 4503481 | 50 | 20 | 51% | 109 |
| 102 | pyruvate dehydrogenase E1 component subunit beta, mitochondrial isoform 1 precursor | 156564403 | 39 | 14 | 42% | 109 |
| 103 | dolichyl-diphosphooligosaccharide--protein glycosyltransferase subunit 1 precursor | 4506675 | 69 | 24 | 49% | 109 |
| 104 | phospholipid hydroperoxide glutathione peroxidase, mitochondrial isoform A precursor | 75709200 | 22 | 14 | 59% | 107 |
| 105 | sperm protein associated with the nucleus on the X chromosome C | 13435137 | 11 | 10 | 82% | 107 |
| 106 | T-complex protein 1 subunit theta | 48762932 | 60 | 24 | 52% | 105 |
| 107 | T-complex protein 1 subunit eta isoform a | 5453607 | 59 | 23 | 53% | 105 |
| 108 | cytochrome c oxidase subunit 4 isoform 1, mitochondrial precursor | 4502981 | 20 | 17 | 56% | 104 |
| 109 | cysteine-rich secretory protein 1 isoform 1 precursor | 327315372 | 28 | 14 | 71% | 103 |
| 110 | lipoprotein lipase precursor | 4557727 | 53 | 16 | 32% | 103 |
| 111 | izumo sperm-egg fusion protein 4 isoform 1 precursor | 89903025 | 24 | 9 | 50% | 101 |
| 112 | isocitrate dehydrogenase [NADP] cytoplasmic | 28178825 | 47 | 18 | 42% | 101 |
| 113 | saccharopine dehydrogenase-like oxidoreductase | 55770836 | 47 | 16 | 59% | 99 |
| 114 | T-complex protein 1 subunit alpha isoform a | 57863257 | 60 | 25 | 63% | 97 |
| 115 | sperm protein associated with the nucleus on the X chromosome B/F | 22027492 | 12 | 7 | 81% | 97 |
| 116 | calnexin precursor | 10716563 | 68 | 21 | 34% | 94 |
| 117 | trifunctional enzyme subunit alpha, mitochondrial precursor | 20127408 | 83 | 24 | 36% | 94 |
| 118 | sperm equatorial segment protein 1 precursor | 21717832 | 39 | 13 | 33% | 94 |
| 119 | NADH-ubiquinone oxidoreductase 75 subunit, mitochondrial isoform 1 | 33519475 | 79 | 26 | 32% | 94 |
| 120 | tektin-2 | 16507950 | 50 | 24 | 62% | 93 |
| 121 | agrin precursor | 54873613 | 215 | 37 | 26% | 93 |
| 122 | annexin A6 isoform 1 | 71773329 | 76 | 24 | 39% | 91 |
| 123 | mitochondrial inner membrane protein isoform 3 | 154354966 | 83 | 26 | 41% | 91 |
| 124 | protein disulfide-isomerase precursor | 20070125 | 57 | 24 | 44% | 90 |
| 125 | cytochrome c oxidase subunit 5A, mitochondrial precursor | 190885499 | 17 | 11 | 71% | 90 |
| 126 | T-complex protein 1 subunit epsilon | 24307939 | 60 | 28 | 58% | 87 |
| 127 | tektin-3 | 13994250 | 57 | 19 | 56% | 86 |
| 128 | peroxiredoxin-1 | 320461711 | 22 | 11 | 58% | 86 |
| 129 | cAMP-dependent protein kinase type I-alpha regulatory subunit | 4506063 | 43 | 13 | 45% | 85 |
| 130 | keratin, type I cytoskeletal 14 | 15431310 | 52 | 9 | 36% | 84 |
| 131 | protein FAM166A | 48717426 | 36 | 15 | 61% | 84 |
| 132 | L-asparaginase | 145275200 | 32 | 13 | 52% | 83 |
| 133 | citrate synthase, mitochondrial precursor | 38327625 | 52 | 21 | 58% | 83 |
| 134 | dynein light chain 2, cytoplasmic | 18087855 | 10 | 6 | 70% | 83 |
| 135 | dynein light chain 1, cytoplasmic | 4505813 | 10 | 8 | 65% | 81 |
| 136 | phosphoglycerate kinase 1 | 4505763 | 45 | 12 | 52% | 80 |
| 137 | valyl-tRNA synthetase | 5454158 | 140 | 26 | 24% | 79 |
| 138 | 6-phosphofructokinase type C isoform 1 | 11321601 | 86 | 25 | 34% | 79 |
| 139 | protein disulfide-isomerase A4 precursor | 4758304 | 73 | 23 | 38% | 79 |
| 140 | histone H2B type 1-A | 24586679 | 14 | 7 | 37% | 78 |
| 141 | cytosol aminopeptidase | 41393561 | 56 | 22 | 61% | 77 |
| 142 | dihydrolipoyllysine-residue acetyltransferase component of pyruvate dehydrogenase complex, mitochondrial precursor | 31711992 | 69 | 16 | 30% | 77 |
| 143 | histone H4 | 4504303 | 11 | 12 | 65% | 77 |
| 144 | radial spoke head 1 homolog | 18254456 | 35 | 14 | 55% | 77 |
| 145 | erlin-2 isoform 1 | 6005721 | 38 | 19 | 58% | 76 |
| 146 | dihydrolipoyl dehydrogenase, mitochondrial precursor | 91199540 | 54 | 21 | 56% | 75 |
| 147 | cytosolic non-specific dipeptidase isoform 1 | 271398239 | 53 | 20 | 49% | 75 |
| 148 | laminin subunit beta-2 precursor | 119703755 | 196 | 24 | 17% | 74 |
| 149 | 14-3-3 protein zeta/delta | 4507953 | 28 | 13 | 59% | 73 |
| 150 | ubiquitin-40S ribosomal protein S27a precursor | 208022622 | 18 | 4 | 30% | 72 |
| 151 | vesicular integral-membrane protein VIP36 precursor | 5803023 | 40 | 15 | 54% | 72 |
| 152 | 2,4-dienoyl-CoA reductase, mitochondrial precursor | 4503301 | 36 | 15 | 59% | 72 |
| 153 | sperm surface protein Sp17 | 8394343 | 17 | 11 | 79% | 72 |
| 154 | cAMP-dependent protein kinase type II-alpha regulatory subunit | 4758958 | 46 | 16 | 52% | 72 |
| 155 | ezrin | 21614499 | 69 | 18 | 29% | 71 |
| 156 | carnitine O-palmitoyltransferase 2, mitochondrial precursor | 4503023 | 74 | 28 | 52% | 71 |
| 157 | importin subunit beta-1 | 19923142 | 97 | 19 | 21% | 70 |
| 158 | serum amyloid P-component precursor | 4502133 | 25 | 9 | 34% | 69 |
| 159 | gastricsin isoform 1 preproprotein | 4505757 | 42 | 5 | 12% | 69 |
| 160 | annexin A3 | 4826643 | 36 | 13 | 28% | 67 |
| 161 | radial spoke head protein 9 homolog isoform 1 | 32964825 | 31 | 19 | 79% | 67 |
| 162 | peptidyl-prolyl cis-trans isomerase A | 10863927 | 18 | 12 | 66% | 64 |
| 163 | peroxiredoxin-5, mitochondrial isoform a precursor | 6912238 | 22 | 14 | 57% | 63 |
| 164 | histone H2A type 1-A | 25092737 | 14 | 7 | 35% | 63 |
| 165 | laminin subunit alpha-5 precursor | 21264602 | 400 | 30 | 11% | 62 |
| 166 | plasma serine protease inhibitor precursor | 194018472 | 46 | 14 | 49% | 62 |
| 167 | acrosin precursor | 148613878 | 46 | 15 | 36% | 62 |
| 168 | dipeptidase 3 isoform a precursor | 193211608 | 56 | 14 | 27% | 61 |
| 169 | D-3-phosphoglycerate dehydrogenase | 23308577 | 57 | 10 | 20% | 60 |
| 170 | peroxiredoxin-2 isoform a | 32189392 | 22 | 10 | 54% | 60 |
| 171 | voltage-dependent anion-selective channel protein 2 isoform 2 | 296317339 | 32 | 11 | 47% | 60 |
| 172 | T-complex protein 1 subunit zeta-2 isoform 1 | 58331173 | 58 | 11 | 37% | 60 |
| 173 | sperm protein associated with the nucleus on the X chromosome E | 22027496 | 11 | 2 | 80% | 60 |
| 174 | protein disulfide-isomerase A6 precursor | 5031973 | 48 | 14 | 40% | 59 |
| 175 | peroxiredoxin-4 precursor | 5453549 | 31 | 12 | 56% | 58 |
| 176 | adipocyte plasma membrane-associated protein | 24308201 | 46 | 19 | 54% | 57 |
| 177 | alpha-actinin-4 | 12025678 | 105 | 22 | 34% | 57 |
| 178 | cofilin-1 | 5031635 | 19 | 10 | 62% | 57 |
| 179 | adenylate kinase isoenzyme 1 | 4502011 | 22 | 10 | 55% | 57 |
| 180 | alpha-centractin | 5031569 | 43 | 13 | 59% | 56 |
| 181 | myosin light polypeptide 6 isoform 2 | 88999583 | 17 | 7 | 58% | 56 |
| 182 | laminin subunit gamma-1 precursor | 145309326 | 178 | 20 | 18% | 55 |
| 183 | peptidyl-prolyl cis-trans isomerase B precursor | 4758950 | 24 | 11 | 52% | 55 |
| 184 | cytochrome c oxidase subunit II | 251831110 | 26 | 7 | 48% | 55 |
| 185 | succinyl-CoA:3-ketoacid-coenzyme A transferase 1, mitochondrial precursor | 4557817 | 56 | 17 | 42% | 55 |
| 186 | tripeptidyl-peptidase 2 | 186972143 | 138 | 24 | 22% | 55 |
| 187 | single-stranded DNA-binding protein, mitochondrial precursor | 4507231 | 17 | 10 | 70% | 54 |
| 188 | epididymal sperm-binding protein 1 precursor | 301601648 | 26 | 9 | 44% | 54 |
| 189 | cytochrome b-c1 complex subunit 1, mitochondrial precursor | 46593007 | 53 | 19 | 47% | 54 |
| 190 | elongation factor 1-delta isoform 1 | 304555581 | 71 | 8 | 17% | 54 |
| 191 | heat shock 70 protein 4L | 31541941 | 95 | 26 | 36% | 54 |
| 192 | keratin, type I cytoskeletal 16 | 24430192 | 51 | 7 | 32% | 54 |
| 193 | acrosomal protein SP-10 isoform a precursor | 4501879 | 28 | 6 | 22% | 53 |
| 194 | carboxypeptidase Z isoform 2 precursor | 62388875 | 73 | 15 | 27% | 53 |
| 195 | mitochondrial carrier homolog 2 | 7657347 | 33 | 10 | 48% | 53 |
| 196 | protein-glutamine gamma-glutamyltransferase 4 | 156627577 | 77 | 14 | 22% | 51 |
| 197 | cytochrome c oxidase subunit 5B, mitochondrial precursor | 17017988 | 14 | 14 | 67% | 51 |
| 198 | 60S acidic ribosomal protein P0 | 16933546 | 34 | 11 | 47% | 51 |
| 199 | uncharacterized protein C9orf9 | 33285006 | 19 | 12 | 81% | 51 |
| 200 | NADH dehydrogenase [ubiquinone] iron-sulfur protein 3, mitochondrial precursor | 4758788 | 30 | 14 | 53% | 51 |
| 201 | plastin-3 isoform 1 | 209862851 | 71 | 10 | 23% | 51 |
| 202 | olfactomedin-4 precursor | 32313593 | 57 | 13 | 32% | 50 |
| 203 | ferritin, mitochondrial precursor | 29126241 | 28 | 11 | 50% | 50 |
| 204 | T-complex protein 1 subunit zeta isoform a | 4502643 | 58 | 17 | 39% | 50 |
| 205 | beta-2-microglobulin precursor | 4757826 | 14 | 4 | 38% | 50 |
| 206 | nuclear pore complex protein Nup155 isoform 1 | 24430149 | 155 | 20 | 18% | 50 |
| 207 | 14-3-3 protein sigma | 5454052 | 28 | 8 | 48% | 50 |
| 208 | matrix-remodeling-associated protein 5 precursor | 139948432 | 312 | 22 | 7.50% | 49 |
| 209 | voltage-dependent anion-selective channel protein 3 isoform 1 | 25188179 | 31 | 12 | 48% | 49 |
| 210 | carbonic anhydrase 4 precursor | 4502519 | 35 | 11 | 34% | 49 |
| 211 | actin-related protein T2 | 29893808 | 42 | 14 | 55% | 49 |
| 212 | fructose-bisphosphate aldolase C | 4885063 | 39 | 3 | 21% | 49 |
| 213 | glutamine synthetase | 19923206 | 42 | 12 | 39% | 48 |
| 214 | myeloid leukemia factor 1 isoform 1 | 11967975 | 31 | 10 | 34% | 48 |
| 215 | 26S protease regulatory subunit 6A | 21361144 | 49 | 14 | 38% | 48 |
| 216 | dolichyl-diphosphooligosaccharide--protein glycosyltransferase 48 subunit precursor | 20070197 | 51 | 11 | 36% | 47 |
| 217 | very long-chain specific acyl-CoA dehydrogenase, mitochondrial isoform 2 precursor | 76496475 | 68 | 19 | 45% | 46 |
| 218 | isocitrate dehydrogenase [NAD] subunit alpha, mitochondrial precursor | 5031777 | 40 | 10 | 34% | 46 |
| 219 | tektin-4 | 21389613 | 51 | 14 | 43% | 45 |
| 220 | uncharacterized protein KIAA1683 isoform a | 224451032 | 147 | 19 | 17% | 45 |
| 221 | protein phosphatase 1 regulatory subunit 7 | 4506013 | 42 | 18 | 63% | 45 |
| 222 | proteasome subunit beta type-5 isoform 1 | 4506201 | 28 | 10 | 39% | 44 |
| 223 | sperm acrosome membrane-associated protein 3 | 27777653 | 23 | 8 | 33% | 44 |
| 224 | 26S proteasome non-ATPase regulatory subunit 2 | 25777602 | 100 | 18 | 25% | 44 |
| 225 | electron transfer flavoprotein subunit alpha, mitochondrial isoform a | 4503607 | 35 | 14 | 64% | 43 |
| 226 | hydroxyacyl-coenzyme A dehydrogenase, mitochondrial isoform 1 precursor | 296179427 | 36 | 9 | 51% | 43 |
| 227 | coiled-coil domain-containing protein 147 | 56961680 | 103 | 19 | 22% | 43 |
| 228 | nucleoporin p54 | 26051237 | 55 | 13 | 34% | 43 |
| 229 | mucin-6 precursor | 151301154 | 257 | 19 | 10% | 42 |
| 230 | nucleoside diphosphate kinase homolog 5 | 4505413 | 24 | 9 | 64% | 42 |
| 231 | 26S protease regulatory subunit 8 isoform 1 | 24497435 | 46 | 10 | 35% | 41 |
| 232 | profilin-1 | 4826898 | 15 | 7 | 51% | 41 |
| 233 | ATP synthase subunit O, mitochondrial precursor | 4502303 | 23 | 9 | 54% | 41 |
| 234 | ATP synthase subunit d, mitochondrial isoform a | 5453559 | 18 | 12 | 80% | 41 |
| 235 | 26S proteasome non-ATPase regulatory subunit 13 isoform 1 | 157502193 | 43 | 11 | 40% | 41 |
| 236 | sodium/potassium-transporting ATPase subunit alpha-4 isoform 1 | 153946397 | 114 | 17 | 19% | 41 |
| 237 | 26S protease regulatory subunit 4 | 24430151 | 49 | 14 | 21% | 41 |
| 238 | glypican-4 precursor | 21614525 | 62 | 12 | 22% | 41 |
| 239 | ras-related protein Rab-11B | 190358517 | 24 | 11 | 49% | 40 |
| 240 | 3-hydroxyisobutyrate dehydrogenase, mitochondrial precursor | 23308751 | 35 | 9 | 30% | 40 |
| 241 | serpin B6 | 41152086 | 43 | 14 | 48% | 39 |
| 242 | cytochrome c1, heme protein, mitochondrial | 21359867 | 35 | 10 | 43% | 39 |
| 243 | 26S proteasome non-ATPase regulatory subunit 7 | 25777615 | 37 | 10 | 40% | 39 |
| 244 | importin subunit alpha-2 | 4504897 | 58 | 14 | 50% | 39 |
| 245 | alanyl-tRNA editing protein Aarsd1 isoform 1 | 217416402 | 66 | 11 | 28% | 39 |
| 246 | L-lactate dehydrogenase A chain isoform 3 | 260099723 | 40 | 11 | 40% | 38 |
| 247 | carboxypeptidase E preproprotein | 4503009 | 53 | 9 | 19% | 38 |
| 248 | proteasome subunit alpha type-5 isoform 1 | 23110942 | 26 | 9 | 52% | 38 |
| 249 | dolichyl-diphosphooligosaccharide--protein glycosyltransferase subunit 2 isoform 2 precursor | 209413738 | 68 | 14 | 33% | 38 |
| 250 | synaptophysin-like protein 1 isoform a | 5803185 | 29 | 5 | 30% | 38 |
| 251 | 26S protease regulatory subunit 6B isoform 1 | 5729991 | 47 | 12 | 38% | 38 |
| 252 | nuclear pore complex protein Nup93 isoform 1 | 208609990 | 93 | 16 | 24% | 38 |
| 253 | SYNJ2BP-COX16 protein isoform 1 | 321400118 | 21 | 7 | 33% | 38 |
| 254 | outer dense fiber protein 3 | 19526475 | 28 | 13 | 57% | 38 |
| 255 | heme oxygenase 2 | 8051608 | 36 | 12 | 46% | 38 |
| 256 | FUN14 domain-containing protein 2 | 24371248 | 21 | 11 | 55% | 38 |
| 257 | erlin-1 | 154800487 | 39 | 8 | 27% | 38 |
| 258 | tetratricopeptide repeat protein 25 | 13899233 | 77 | 14 | 13% | 38 |
| 259 | ubiquitin-like modifier-activating enzyme 1 | 23510340 | 118 | 9 | 8.20% | 37 |
| 260 | nucleobindin-2 precursor | 4826870 | 50 | 13 | 44% | 37 |
| 261 | stress-70 protein, mitochondrial precursor | 24234688 | 74 | 13 | 29% | 37 |
| 262 | isocitrate dehydrogenase [NAD] subunit beta, mitochondrial isoform a precursor | 28178821 | 42 | 13 | 44% | 37 |
| 263 | importin-5 | 24797086 | 126 | 13 | 17% | 36 |
| 264 | protein FAM71B | 222418633 | 65 | 8 | 12% | 36 |
| 265 | hyaluronidase PH-20 isoform 2 | 291290981 | 58 | 9 | 23% | 36 |
| 266 | electron transfer flavoprotein subunit beta isoform 1 | 4503609 | 28 | 12 | 49% | 36 |
| 267 | thioredoxin domain-containing protein 3 | 148839372 | 67 | 15 | 31% | 36 |
| 268 | protein NDRG1 | 37655183 | 43 | 5 | 22% | 35 |
| 269 | proteasome subunit alpha type-2 | 4506181 | 26 | 11 | 57% | 35 |
| 270 | hsc70-interacting protein | 19923193 | 41 | 6 | 20% | 35 |
| 271 | collagen alpha-1(XVIII) chain isoform 1 precursor | 110611235 | 154 | 10 | 9.00% | 35 |
| 272 | vesicle-associated membrane protein-associated protein A isoform 2 | 94721252 | 28 | 7 | 38% | 35 |
| 273 | 26S proteasome non-ATPase regulatory subunit 11 | 28872725 | 47 | 11 | 31% | 35 |
| 274 | mitochondria-eating protein | 21687119 | 61 | 9 | 18% | 35 |
| 275 | epididymal secretory protein E3-beta precursor | 11641279 | 18 | 7 | 52% | 35 |
| 276 | delta(3,5)-Delta(2,4)-dienoyl-CoA isomerase, mitochondrial precursor | 70995211 | 36 | 13 | 55% | 35 |
| 277 | protein S100-A8 | 21614544 | 11 | 7 | 53% | 34 |
| 278 | transmembrane emp24 domain-containing protein 10 precursor | 98986464 | 25 | 9 | 40% | 34 |
| 279 | succinate dehydrogenase [ubiquinone] iron-sulfur subunit, mitochondrial precursor | 115387094 | 32 | 12 | 38% | 34 |
| 280 | uncharacterized protein C20orf107 precursor | 71043642 | 19 | 12 | 55% | 34 |
| 281 | barrier-to-autointegration factor | 4502389 | 10 | 9 | 71% | 34 |
| 282 | L-lactate dehydrogenase A-like 6B | 15082234 | 42 | 11 | 42% | 34 |
| 283 | pyruvate dehydrogenase E1 component subunit alpha, testis-specific form, mitochondrial precursor | 4885543 | 43 | 12 | 39% | 34 |
| 284 | thioredoxin-related transmembrane protein 4 precursor | 40254947 | 39 | 5 | 17% | 34 |
| 285 | signal peptidase complex subunit 2 | 162417971 | 25 | 10 | 44% | 34 |
| 286 | lysosomal alpha-glucosidase preproprotein | 119393891 | 105 | 9 | 11% | 34 |
| 287 | maltase-glucoamylase, intestinal | 221316699 | 210 | 14 | 10% | 33 |
| 288 | glucose-6-phosphate isomerase isoform 2 | 18201905 | 63 | 12 | 35% | 33 |
| 289 | neprilysin | 116256329 | 86 | 12 | 19% | 33 |
| 290 | glycerol kinase 2 | 41393575 | 61 | 10 | 24% | 33 |
| 291 | axonemal dynein light intermediate polypeptide 1 | 37595560 | 32 | 11 | 43% | 33 |
| 292 | nuclear pore membrane glycoprotein 210 precursor | 27477134 | 205 | 14 | 9.40% | 33 |
| 293 | anterior gradient protein 2 homolog precursor | 5453541 | 20 | 6 | 47% | 33 |
| 294 | enoyl-CoA hydratase, mitochondrial | 194097323 | 31 | 12 | 48% | 33 |
| 295 | prohibitin | 4505773 | 30 | 11 | 58% | 33 |
| 296 | cytochrome b-c1 complex subunit 7 isoform 1 | 5454152 | 14 | 8 | 59% | 33 |
| 297 | DPY30 domain-containing protein 1 | 20270377 | 21 | 7 | 42% | 33 |
| 298 | calmodulin | 58218968 | 17 | 9 | 81% | 32 |
| 299 | histone H3.1 | 4504281 | 15 | 5 | 43% | 32 |
| 300 | medium-chain specific acyl-CoA dehydrogenase, mitochondrial isoform b precursor | 187960098 | 47 | 12 | 35% | 32 |
| 301 | chloride intracellular channel protein 1 | 14251209 | 27 | 6 | 36% | 32 |
| 302 | protein DJ-1 | 31543380 | 20 | 13 | 87% | 32 |
| 303 | synaptic vesicle membrane protein VAT-1 homolog | 18379349 | 42 | 12 | 47% | 32 |
| 304 | apolipoprotein A-I preproprotein | 4557321 | 31 | 10 | 39% | 32 |
| 305 | glucosidase 2 subunit beta isoform 1 precursor | 48255889 | 59 | 9 | 18% | 31 |
| 306 | 14-3-3 protein theta | 5803227 | 28 | 8 | 43% | 31 |
| 307 | ADP/ATP translocase 4 | 13775208 | 35 | 9 | 31% | 31 |
| 308 | myosin-10 | 41406064 | 229 | 7 | 9.30% | 31 |
| 309 | eukaryotic translation initiation factor 5A-1 isoform B | 219555712 | 17 | 8 | 51% | 31 |
| 310 | reticulocalbin-2 precursor | 4506457 | 37 | 10 | 44% | 31 |
| 311 | ras-related protein Rab-14 | 19923483 | 24 | 10 | 56% | 31 |
| 312 | short-chain specific acyl-CoA dehydrogenase, mitochondrial precursor | 4557233 | 44 | 10 | 34% | 31 |
| 313 | lysyl-tRNA synthetase isoform 1 | 194272210 | 71 | 8 | 16% | 31 |
| 314 | histone H2A type 2-A | 106775678 | 14 | 2 | 35% | 31 |
| 315 | L-xylulose reductase isoform 1 | 7705925 | 26 | 8 | 37% | 30 |
| 316 | alpha-crystallin B chain | 4503057 | 20 | 11 | 55% | 30 |
| 317 | ATP synthase subunit b, mitochondrial precursor | 21361565 | 29 | 8 | 44% | 30 |
| 318 | parkin coregulated gene protein isoform 2 | 122939202 | 29 | 9 | 46% | 30 |
| 319 | radial spoke head protein 3 homolog | 31543559 | 64 | 8 | 20% | 30 |
| 320 | 2-oxoglutarate dehydrogenase, mitochondrial isoform 3 precursor | 259013553 | 116 | 14 | 18% | 30 |
| 321 | proteasome subunit alpha type-7 | 4506189 | 28 | 5 | 52% | 30 |
| 322 | keratin, type I cytoskeletal 19 | 24234699 | 44 | 5 | 34% | 30 |
| 323 | aspartate aminotransferase, mitochondrial precursor | 73486658 | 48 | 10 | 35% | 29 |
| 324 | 4-trimethylaminobutyraldehyde dehydrogenase | 115387104 | 56 | 9 | 22% | 29 |
| 325 | alcohol dehydrogenase [NADP+] | 24497577 | 37 | 9 | 30% | 29 |
| 326 | nitric oxide synthase, endothelial isoform 1 | 40254422 | 133 | 7 | 7.70% | 29 |
| 327 | vesicle-fusing ATPase | 156564401 | 83 | 12 | 18% | 29 |
| 328 | protein dpy-19 homolog 2 | 93277105 | 87 | 11 | 16% | 29 |
| 329 | peptidyl-prolyl cis-trans isomerase FKBP4 | 4503729 | 52 | 12 | 33% | 29 |
| 330 | enkurin | 21450721 | 29 | 8 | 34% | 29 |
| 331 | enoyl-CoA delta isomerase 1, mitochondrial isoform 2 precursor | 295842266 | 31 | 7 | 25% | 29 |
| 332 | ES1 protein homolog, mitochondrial isoform Ia precursor | 296531406 | 28 | 8 | 41% | 29 |
| 333 | endophilin-B1 isoform 2 | 331284170 | 44 | 8 | 20% | 29 |
| 334 | von Willebrand factor A domain-containing protein 1 isoform 1 precursor | 40068485 | 47 | 12 | 44% | 29 |
| 335 | large proline-rich protein BAG6 isoform a | 149158692 | 119 | 10 | 14% | 29 |
| 336 | ras-related protein Rab-2B isoform 1 | 21361884 | 24 | 3 | 56% | 29 |
| 337 | glutamate carboxypeptidase 2 isoform 1 | 4758398 | 84 | 12 | 22% | 28 |
| 338 | adenylate kinase 7 | 148727333 | 83 | 9 | 11% | 28 |
| 339 | uncharacterized protein C20orf106 precursor | 71043622 | 20 | 5 | 54% | 28 |
| 340 | stomatin-like protein 2 | 7305503 | 39 | 9 | 47% | 28 |
| 341 | coiled-coil domain-containing protein 63 | 22749217 | 66 | 11 | 26% | 28 |
| 342 | alpha-actinin-1 isoform c | 194097352 | 103 | 6 | 16% | 28 |
| 343 | glutathione S-transferase P | 4504183 | 23 | 6 | 52% | 27 |
| 344 | annexin A4 | 4502105 | 36 | 12 | 42% | 27 |
| 345 | proteasome subunit beta type-7 proprotein | 4506203 | 30 | 11 | 42% | 27 |
| 346 | 26S protease regulatory subunit 10B | 195539395 | 46 | 9 | 23% | 27 |
| 347 | flavin reductase (NADPH) | 4502419 | 22 | 4 | 24% | 27 |
| 348 | ADP-ribosylation factor 1 | 4502201 | 21 | 8 | 45% | 27 |
| 349 | ras-related protein Rab-1B | 13569962 | 22 | 4 | 25% | 27 |
| 350 | isochorismatase domain-containing protein 2, mitochondrial isoform 2 | 13376007 | 24 | 6 | 60% | 27 |
| 351 | 60S acidic ribosomal protein P2 | 4506671 | 12 | 6 | 74% | 27 |
| 352 | 26S proteasome non-ATPase regulatory subunit 14 | 5031981 | 35 | 9 | 47% | 27 |
| 353 | kallikrein-2 isoform 1 precursor | 5031829 | 29 | 6 | 27% | 27 |
| 354 | calmin | 19923599 | 112 | 8 | 13% | 27 |
| 355 | filamin-B isoform 2 | 105990514 | 278 | 15 | 7.50% | 26 |
| 356 | proteasome subunit alpha type-1 isoform 1 | 23110935 | 30 | 9 | 39% | 26 |
| 357 | G-protein coupled receptor 64 isoform 2 precursor | 119943116 | 110 | 7 | 9.40% | 26 |
| 358 | 10 heat shock protein, mitochondrial | 4504523 | 11 | 8 | 60% | 26 |
| 359 | transmembrane emp24 domain-containing protein 9 precursor | 39725636 | 27 | 9 | 20% | 26 |
| 360 | protein FAM154A | 301129242 | 55 | 9 | 23% | 26 |
| 361 | epididymal secretory protein E3-alpha precursor | 11386189 | 18 | 5 | 31% | 26 |
| 362 | alpha-mannosidase 2C1 | 46852164 | 116 | 13 | 14% | 26 |
| 363 | cullin-associated NEDD8-dissociated protein 1 | 21361794 | 136 | 12 | 13% | 25 |
| 364 | phosphatidylethanolamine-binding protein 1 preproprotein | 4505621 | 21 | 6 | 57% | 25 |
| 365 | sperm-associated antigen 6 isoform 1 | 6912678 | 55 | 9 | 34% | 25 |
| 366 | carbonyl reductase [NADPH] 1 | 4502599 | 30 | 6 | 28% | 25 |
| 367 | tektin-1 | 16753231 | 48 | 8 | 29% | 25 |
| 368 | testis-expressed protein 101 isoform 1 | 194018544 | 29 | 5 | 23% | 25 |
| 369 | malectin precursor | 7661948 | 32 | 9 | 41% | 25 |
| 370 | protein S100-A11 | 5032057 | 12 | 7 | 56% | 25 |
| 371 | cytochrome b-c1 complex subunit Rieske, mitochondrial | 163644321 | 30 | 8 | 30% | 25 |
| 372 | calmegin precursor | 4758004 | 70 | 9 | 15% | 25 |
| 373 | 26S proteasome non-ATPase regulatory subunit 3 | 25777612 | 61 | 12 | 29% | 25 |
| 374 | ubiquitin carboxyl-terminal hydrolase isozyme L3 | 5174741 | 26 | 7 | 35% | 25 |
| 375 | pyruvate dehydrogenase protein X component, mitochondrial isoform 2 | 203098816 | 51 | 10 | 25% | 25 |
| 376 | GTP-binding nuclear protein Ran | 5453555 | 24 | 7 | 23% | 25 |
| 377 | 26S proteasome non-ATPase regulatory subunit 1 isoform 1 | 25777600 | 106 | 10 | 9.70% | 25 |
| 378 | cAMP-dependent protein kinase catalytic subunit alpha isoform 2 | 46909584 | 40 | 7 | 26% | 25 |
| 379 | keratin, type I cytoskeletal 18 | 4557888 | 48 | 9 | 33% | 25 |
| 380 | sodium/potassium-transporting ATPase subunit alpha-2 proprotein | 4502271 | 112 | 2 | 11% | 25 |
| 381 | sodium/potassium-transporting ATPase subunit alpha-3 | 22748667 | 112 | 2 | 12% | 25 |
| 382 | proteasome subunit alpha type-6 | 23110944 | 27 | 9 | 43% | 24 |
| 383 | proteasome subunit alpha type-3 isoform 2 | 23110939 | 28 | 7 | 30% | 24 |
| 384 | CDGSH iron-sulfur domain-containing protein 1 | 8923930 | 12 | 6 | 56% | 24 |
| 385 | adenylyl cyclase-associated protein 1 | 5453595 | 52 | 8 | 17% | 24 |
| 386 | hydroxyacylglutathione hydrolase, mitochondrial isoform 1 precursor | 94538322 | 34 | 9 | 36% | 24 |
| 387 | prenylcysteine oxidase 1 precursor | 166795301 | 57 | 10 | 25% | 24 |
| 388 | dnaJ homolog subfamily B member 11 precursor | 7706495 | 41 | 10 | 27% | 24 |
| 389 | rab GDP dissociation inhibitor beta isoform 1 | 6598323 | 51 | 10 | 27% | 24 |
| 390 | beta-lactamase-like protein 2 | 7705793 | 33 | 9 | 43% | 24 |
| 391 | dynactin subunit 1 isoform 1 | 13259510 | 142 | 13 | 8.50% | 24 |
| 392 | dihydrolipoyllysine-residue succinyltransferase component of 2-oxoglutarate dehydrogenase complex, mitochondrial isoform 1 precursor | 19923748 | 49 | 11 | 36% | 23 |
| 393 | proteasome subunit alpha type-7-like isoform 2 | 68303563 | 28 | 8 | 42% | 23 |
| 394 | proteasome subunit beta type-3 | 22538465 | 23 | 7 | 47% | 23 |
| 395 | NADH-cytochrome b5 reductase 2 | 47778923 | 31 | 9 | 47% | 23 |
| 396 | endoplasmic reticulum resident protein 44 precursor | 52487191 | 47 | 8 | 27% | 23 |
| 397 | succinyl-CoA ligase [ADP-forming] subunit beta, mitochondrial precursor | 11321583 | 50 | 7 | 12% | 23 |
| 398 | ras-related protein Rab-3B | 19923750 | 25 | 5 | 22% | 23 |
| 399 | vimentin | 62414289 | 54 | 8 | 24% | 23 |
| 400 | acrosome formation-associated factor isoform 1 | 239582757 | 33 | 6 | 30% | 23 |
| 401 | abhydrolase domain-containing protein 10, mitochondrial precursor | 8923001 | 34 | 7 | 33% | 23 |
| 402 | adenylate kinase 8 | 22749187 | 55 | 11 | 28% | 23 |
| 403 | histone H2A.V isoform 1 | 6912616 | 14 | 3 | 31% | 23 |
| 404 | uncharacterized protein C1orf194 . | 171916088 | 18 | 6 | 55% | 23 |
| 405 | beta-centractin | 11342680 | 42 | 2 | 36% | 23 |
| 406 | glutathione reductase, mitochondrial isoform 1 precursor | 50301238 | 56 | 10 | 32% | 22 |
| 407 | ras GTPase-activating-like protein IQGAP1 | 4506787 | 189 | 10 | 7.80% | 22 |
| 408 | transmembrane protease serine 2 isoform 2 | 205360943 | 54 | 5 | 10% | 22 |
| 409 | proteasome subunit beta type-6 | 23110925 | 25 | 4 | 29% | 22 |
| 410 | proteasome activator complex subunit 1 isoform 1 | 5453990 | 29 | 9 | 41% | 22 |
| 411 | rho GDP-dissociation inhibitor 1 isoform a | 4757768 | 23 | 4 | 26% | 22 |
| 412 | cathepsin F precursor | 6042196 | 53 | 7 | 13% | 22 |
| 413 | NADH dehydrogenase [ubiquinone] flavoprotein 1, mitochondrial isoform 1 precursor | 20149568 | 51 | 8 | 29% | 22 |
| 414 | F-actin-capping protein subunit alpha-1 | 5453597 | 33 | 5 | 34% | 22 |
| 415 | transthyretin precursor | 4507725 | 16 | 8 | 54% | 22 |
| 416 | sodium/potassium-transporting ATPase subunit alpha-1 isoform a | 21361181 | 113 | 3 | 13% | 22 |
| 417 | superoxide dismutase [Mn], mitochondrial isoform A precursor | 67782305 | 25 | 7 | 38% | 21 |
| 418 | galectin-3-binding protein precursor | 5031863 | 65 | 6 | 11% | 21 |
| 419 | proteasome subunit beta type-1 | 4506193 | 26 | 7 | 40% | 21 |
| 420 | uromodulin precursor | 59850812 | 70 | 7 | 12% | 21 |
| 421 | protein-L-isoaspartate(D-aspartate) O-methyltransferase isoform 2 | 354983493 | 30 | 7 | 45% | 21 |
| 422 | endoplasmic reticulum resident protein 29 isoform 1 precursor | 5803013 | 29 | 8 | 35% | 21 |
| 423 | coiled-coil domain-containing protein 19, mitochondrial precursor | 81295816 | 66 | 7 | 15% | 21 |
| 424 | membrane-associated progesterone receptor component 2 | 291621647 | 26 | 4 | 17% | 21 |
| 425 | eukaryotic translation initiation factor 3 subunit F | 4503519 | 38 | 7 | 23% | 21 |
| 426 | lysozyme-like protein 4 precursor | 21389465 | 16 | 5 | 42% | 21 |
| 427 | dnaJ homolog subfamily B member 1 | 5453690 | 38 | 6 | 16% | 21 |
| 428 | brain acid soluble protein 1 | 30795231 | 23 | 6 | 40% | 20 |
| 429 | WD repeat-containing protein 16 isoform b | 124028512 | 68 | 9 | 13% | 20 |
| 430 | 6-phosphogluconate dehydrogenase, decarboxylating | 40068518 | 53 | 7 | 23% | 20 |
| 431 | proteasome subunit alpha type-4 isoform 1 | 4506185 | 29 | 8 | 46% | 20 |
| 432 | complement component 1 Q subcomponent-binding protein, mitochondrial precursor | 4502491 | 31 | 6 | 32% | 20 |
| 433 | hypoxanthine-guanine phosphoribosyltransferase | 4504483 | 25 | 6 | 32% | 20 |
| 434 | myosin regulatory light chain 12B | 15809016 | 20 | 7 | 51% | 20 |
| 435 | transcription factor A, mitochondrial precursor | 4507401 | 29 | 9 | 41% | 20 |
| 436 | sperm acrosome-associated protein 5 precursor | 120952755 | 18 | 5 | 31% | 20 |
| 437 | uncharacterized protein C2orf57 | 284413778 | 42 | 4 | 15% | 20 |
| 438 | long-chain-fatty-acid--CoA ligase 6 isoform e | 327412327 | 79 | 9 | 17% | 20 |
| 439 | histone H1t . | 20544168 | 22 | 7 | 30% | 20 |
| 440 | dnaJ homolog subfamily A member 2 | 5031741 | 46 | 7 | 27% | 20 |
| 441 | melanoma inhibitory activity protein 3 precursor | 122891870 | 214 | 9 | 6.20% | 20 |
| 442 | 14-3-3 protein beta/alpha | 21328448 | 28 | 3 | 35% | 20 |
| 443 | normal mucosa of esophagus-specific gene 1 protein | 37694067 | 10 | 5 | 52% | 20 |
| 444 | solute carrier family 2, facilitated glucose transporter member 14 | 23592238 | 56 | 6 | 13% | 19 |
| 445 | protein NipSnap homolog 3A | 22267436 | 28 | 6 | 31% | 19 |
| 446 | CD59 glycoprotein preproprotein | 187828910 | 14 | 5 | 26% | 19 |
| 447 | NME1-NME2 protein | 66392203 | 30 | 8 | 35% | 19 |
| 448 | translin | 4759270 | 26 | 6 | 38% | 19 |
| 449 | transmembrane protein 190 precursor | 21040263 | 19 | 6 | 39% | 19 |
| 450 | nicastrin precursor | 24638433 | 78 | 8 | 13% | 19 |
| 451 | general vesicular transport factor p115 | 4505541 | 108 | 9 | 8.90% | 19 |
| 452 | kunitz-type protease inhibitor 3 precursor | 189571689 | 10 | 3 | 29% | 19 |
| 453 | coiled-coil-helix-coiled-coil-helix domain-containing protein 3, mitochondrial precursor | 8923390 | 26 | 6 | 19% | 19 |
| 454 | sperm acrosome membrane-associated protein 4 precursor | 19424138 | 13 | 2 | 22% | 19 |
| 455 | deoxyguanosine kinase, mitochondrial isoform a precursor | 18426967 | 32 | 7 | 35% | 19 |
| 456 | coiled-coil domain-containing protein 105 | 226492892 | 57 | 7 | 16% | 19 |
| 457 | cytochrome c | 11128019 | 12 | 5 | 46% | 19 |
| 458 | NADH dehydrogenase [ubiquinone] iron-sulfur protein 6, mitochondrial precursor | 4758792 | 14 | 6 | 61% | 19 |
| 459 | L-lactate dehydrogenase B chain | 4557032 | 37 | 6 | 31% | 19 |
| 460 | ADP-ribosylation factor 4 | 4502205 | 21 | 4 | 52% | 19 |
| 461 | lipid phosphate phosphohydrolase 1 isoform 1 | 29171736 | 32 | 3 | 16% | 18 |
| 462 | sodium/potassium-transporting ATPase subunit beta-3 | 4502281 | 32 | 8 | 33% | 18 |
| 463 | lactoylglutathione lyase | 118402586 | 21 | 5 | 22% | 18 |
| 464 | glypican-1 precursor | 167001141 | 62 | 7 | 10% | 18 |
| 465 | thioredoxin reductase 2, mitochondrial precursor | 22035672 | 57 | 7 | 27% | 18 |
| 466 | transmembrane and coiled-coil domain-containing protein 2 | 56847610 | 20 | 5 | 33% | 18 |
| 467 | extracellular matrix protein 1 isoform 3 precursor | 322302700 | 64 | 7 | 12% | 18 |
| 468 | translationally-controlled tumor protein | 4507669 | 20 | 4 | 24% | 18 |
| 469 | uncharacterized protein C9orf24 isoform 1 | 21362074 | 30 | 7 | 29% | 18 |
| 470 | EGF-like repeat and discoidin I-like domain-containing protein 3 precursor | 31317224 | 54 | 8 | 24% | 18 |
| 471 | NADH dehydrogenase [ubiquinone] 1 beta subcomplex subunit 10 | 4758774 | 21 | 6 | 37% | 18 |
| 472 | prostate and testis expressed protein 1 precursor | 19923082 | 14 | 6 | 55% | 18 |
| 473 | EF-hand domain-containing family member C2 | 31542743 | 87 | 6 | 7.30% | 18 |
| 474 | 26S proteasome non-ATPase regulatory subunit 8 | 156631005 | 40 | 7 | 26% | 18 |
| 475 | EF-hand domain-containing protein 1 isoform 1 | 156616292 | 74 | 8 | 14% | 18 |
| 476 | nucleotide exchange factor SIL1 precursor | 11968009 | 52 | 7 | 16% | 18 |
| 477 | adenylate kinase 2, mitochondrial isoform a | 4502013 | 26 | 6 | 36% | 18 |
| 478 | nuclear pore glycoprotein p62 | 24497605 | 53 | 5 | 12% | 18 |
| 479 | aspartyl aminopeptidase | 156416028 | 53 | 8 | 30% | 17 |
| 480 | lysosome-associated membrane glycoprotein 1 precursor | 112380628 | 45 | 5 | 12% | 17 |
| 481 | proteasome subunit beta type-4 | 22538467 | 29 | 7 | 39% | 17 |
| 482 | dynein heavy chain 8, axonemal | 332688227 | 539 | 10 | 2.40% | 17 |
| 483 | calpain small subunit 1 | 51599151 | 28 | 5 | 20% | 17 |
| 484 | dynein heavy chain 17, axonemal | 256542310 | 509 | 8 | 2.90% | 17 |
| 485 | hornerin | 57864582 | 282 | 7 | 5.20% | 17 |
| 486 | 26S protease regulatory subunit 7 isoform 1 | 4506209 | 49 | 7 | 22% | 17 |
| 487 | eukaryotic initiation factor 4A-I isoform 1 | 4503529 | 46 | 6 | 21% | 17 |
| 488 | carboxypeptidase D isoform 1 precursor | 22202611 | 153 | 9 | 9.50% | 17 |
| 489 | lactadherin isoform a preproprotein | 167830475 | 43 | 5 | 18% | 17 |
| 490 | bifunctional aminoacyl-tRNA synthetase | 62241042 | 171 | 8 | 5.70% | 17 |
| 491 | receptor expression-enhancing protein 6 | 19923919 | 21 | 5 | 22% | 17 |
| 492 | serine/threonine-protein phosphatase PP1-beta catalytic subunit isoform 1 | 4506005 | 37 | 5 | 21% | 17 |
| 493 | ribonuclease-like protein 13 precursor | 59276062 | 18 | 5 | 33% | 17 |
| 494 | keratin, type II cytoskeletal 8 | 4504919 | 54 | 4 | 14% | 17 |
| 495 | exportin-7 | 154448892 | 124 | 8 | 9.20% | 17 |
| 496 | leucine zipper transcription factor-like protein 1 | 9966793 | 35 | 7 | 23% | 17 |
| 497 | 40S ribosomal protein SA | 59859885 | 33 | 6 | 35% | 17 |
| 498 | acyl-CoA-binding protein isoform 5 | 295842514 | 14 | 5 | 44% | 17 |
| 499 | voltage-dependent anion-selective channel protein 1 | 4507879 | 31 | 6 | 23% | 17 |
| 500 | L-amino-acid oxidase isoform 2 precursor | 27477089 | 65 | 7 | 19% | 17 |
| 501 | uncharacterized protein C15orf26 | 148747373 | 34 | 6 | 25% | 17 |
| 502 | cytochrome c oxidase subunit 6A1, mitochondrial precursor | 17999528 | 12 | 2 | 43% | 17 |
| 503 | adenosylhomocysteinase isoform 1 | 9951915 | 48 | 9 | 24% | 16 |
| 504 | protein S100-A9 | 4506773 | 13 | 6 | 53% | 16 |
| 505 | tektin-5 | 21389569 | 56 | 7 | 18% | 16 |
| 506 | copper homeostasis protein cutC homolog | 148596990 | 29 | 7 | 41% | 16 |
| 507 | membrane-associated progesterone receptor component 1 | 5729875 | 22 | 6 | 24% | 16 |
| 508 | T-complex protein 11 homolog isoform 1 | 148226214 | 57 | 5 | 19% | 16 |
| 509 | testis-specific H1 histone | 32401437 | 28 | 4 | 13% | 16 |
| 510 | glycodelin precursor | 65507501 | 21 | 3 | 24% | 16 |
| 511 | phosphoglycerate mutase 2 | 50593010 | 29 | 8 | 46% | 16 |
| 512 | adenine phosphoribosyltransferase isoform a | 4502171 | 20 | 6 | 43% | 16 |
| 513 | alpha-soluble NSF attachment protein | 47933379 | 33 | 8 | 39% | 16 |
| 514 | cytochrome c oxidase subunit 6B1 | 4502985 | 10 | 5 | 57% | 16 |
| 515 | serine/threonine-protein phosphatase with EF-hands 1 isoform 1b | 23312374 | 73 | 6 | 11% | 16 |
| 516 | ATP synthase subunit gamma, mitochondrial isoform L (liver) precursor | 50345988 | 33 | 6 | 22% | 16 |
| 517 | LETM1 and EF-hand domain-containing protein 1, mitochondrial precursor | 6912482 | 83 | 7 | 12% | 16 |
| 518 | heme-binding protein 2 | 7657603 | 23 | 4 | 20% | 16 |
| 519 | transcription elongation factor B polypeptide 1 isoform a | 325652033 | 12 | 5 | 51% | 16 |
| 520 | transmembrane protein 89 precursor | 56847630 | 18 | 4 | 28% | 16 |
| 521 | cadherin-1 preproprotein | 4757960 | 97 | 5 | 5.80% | 16 |
| 522 | programmed cell death protein 6 | 7019485 | 22 | 7 | 61% | 16 |
| 523 | histone H2A-Bbd type 2/3 | 63029935 | 13 | 5 | 67% | 16 |
| 524 | eukaryotic translation initiation factor 3 subunit I | 4503513 | 37 | 6 | 31% | 16 |
| 525 | 14-3-3 protein gamma | 21464101 | 28 | 2 | 20% | 16 |
| 526 | NADH dehydrogenase [ubiquinone] 1 alpha subcomplex subunit 5 | 4826848 | 13 | 6 | 66% | 16 |
| 527 | 3-hydroxyacyl-CoA dehydrogenase type-2 isoform 1 . | 4758504 | 27 | 6 | 33% | 16 |
| 528 | myeloperoxidase precursor | 4557759 | 84 | 9 | 16% | 15 |
| 529 | zinc-alpha-2-glycoprotein precursor | 4502337 | 34 | 6 | 27% | 15 |
| 530 | ras-related protein Rab-27B | 5729997 | 25 | 6 | 26% | 15 |
| 531 | prostasin preproprotein | 4506153 | 36 | 5 | 22% | 15 |
| 532 | plasma membrane calcium-transporting ATPase 4 isoform 4b | 48255957 | 134 | 7 | 6.90% | 15 |
| 533 | zymogen granule protein 16 homolog B precursor | 94536866 | 23 | 4 | 27% | 15 |
| 534 | dynactin subunit 2 | 5453629 | 45 | 5 | 18% | 15 |
| 535 | 40S ribosomal protein S3a | 4506723 | 30 | 6 | 28% | 15 |
| 536 | dnaJ homolog subfamily B member 8 | 23503241 | 26 | 6 | 36% | 15 |
| 537 | kinectin isoform a | 33620775 | 156 | 8 | 6.60% | 15 |
| 538 | transmembrane emp24 domain-containing protein 4 precursor | 33457308 | 26 | 4 | 25% | 15 |
| 539 | EF-hand calcium-binding domain-containing protein 6 isoform a | 38570107 | 173 | 7 | 6.70% | 15 |
| 540 | histidine triad nucleotide-binding protein 1 | 4885413 | 14 | 6 | 72% | 15 |
| 541 | NADH dehydrogenase [ubiquinone] iron-sulfur protein 8, mitochondrial precursor | 4505371 | 24 | 4 | 20% | 15 |
| 542 | fatty acid-binding protein, epidermal | 4557581 | 15 | 6 | 38% | 15 |
| 543 | ADP/ATP translocase 2 | 156071459 | 33 | 5 | 24% | 15 |
| 544 | serine/threonine-protein phosphatase PGAM5, mitochondrial isoform 1 | 281604136 | 32 | 4 | 12% | 15 |
| 545 | cullin-3 | 4503165 | 89 | 10 | 17% | 15 |
| 546 | phosphoglycerate mutase 1 | 4505753 | 29 | 5 | 46% | 15 |
| 547 | RPS10-NUDT3 protein | 321117084 | 33 | 3 | 14% | 15 |
| 548 | cancer/testis antigen 47A | 121949786 | 30 | 4 | 25% | 15 |
| 549 | eukaryotic initiation factor 4A-II | 83700235 | 46 | 2 | 21% | 15 |
| 550 | 14-3-3 protein eta | 4507951 | 28 | 2 | 20% | 15 |
| 551 | cofilin-2 isoform 1 | 33946278 | 19 | 2 | 39% | 15 |
| 552 | aspartate aminotransferase, cytoplasmic | 4504067 | 46 | 7 | 28% | 14 |
| 553 | epididymal secretory protein E1 precursor | 5453678 | 17 | 5 | 39% | 14 |
| 554 | gamma-glutamyltranspeptidase 1 precursor | 73915090 | 61 | 4 | 7.20% | 14 |
| 555 | casein kinase II subunit alpha isoform a | 29570791 | 45 | 5 | 23% | 14 |
| 556 | protein ERGIC-53 precursor | 5031873 | 58 | 5 | 9.40% | 14 |
| 557 | beta-defensin 129 precursor | 18250304 | 20 | 5 | 30% | 14 |
| 558 | receptor expression-enhancing protein 5 | 115430112 | 21 | 3 | 11% | 14 |
| 559 | endoplasmic reticulum-Golgi intermediate compartment protein 3 isoform a | 38327615 | 44 | 6 | 15% | 14 |
| 560 | vesicle-associated membrane protein 3 | 4759300 | 11 | 5 | 41% | 14 |
| 561 | BPI fold-containing family A member 3 isoform 1 precursor | 109627654 | 28 | 4 | 28% | 14 |
| 562 | metalloproteinase inhibitor 1 precursor | 4507509 | 23 | 5 | 37% | 14 |
| 563 | 40S ribosomal protein S2 | 15055539 | 31 | 6 | 21% | 14 |
| 564 | ropporin-1-like protein | 17572807 | 26 | 6 | 29% | 14 |
| 565 | basigin isoform 2 precursor | 38372925 | 29 | 5 | 26% | 14 |
| 566 | ATPase inhibitor, mitochondrial isoform 1 precursor | 7705927 | 12 | 2 | 7.50% | 14 |
| 567 | speriolin isoform 1 | 197276668 | 62 | 4 | 6.90% | 14 |
| 568 | calcium-binding and spermatid-specific protein 1 | 90652863 | 43 | 3 | 10% | 14 |
| 569 | retinoid-inducible serine carboxypeptidase precursor | 11055992 | 51 | 7 | 18% | 14 |
| 570 | translocon-associated protein subunit alpha precursor | 169404009 | 32 | 3 | 12% | 14 |
| 571 | histidine triad nucleotide-binding protein 2, mitochondrial precursor | 14211923 | 17 | 4 | 37% | 14 |
| 572 | NADH dehydrogenase [ubiquinone] flavoprotein 2, mitochondrial precursor | 222080062 | 27 | 5 | 24% | 14 |
| 573 | serine protease 58 precursor | 48255915 | 27 | 5 | 26% | 14 |
| 574 | 40S ribosomal protein S18 | 11968182 | 18 | 5 | 31% | 14 |
| 575 | 40S ribosomal protein S16 | 4506691 | 16 | 3 | 19% | 14 |
| 576 | ATP synthase subunit g, mitochondrial | 51479156 | 11 | 4 | 47% | 14 |
| 577 | tryptophanyl-tRNA synthetase, cytoplasmic isoform b | 47419918 | 49 | 6 | 23% | 14 |
| 578 | mitochondrial import receptor subunit TOM22 homolog | 9910382 | 16 | 4 | 54% | 14 |
| 579 | heterogeneous nuclear ribonucleoprotein M isoform a | 14141152 | 78 | 7 | 9.90% | 14 |
| 580 | dnaJ homolog subfamily B member 13 | 39204547 | 36 | 5 | 20% | 14 |
| 581 | nidogen-1 precursor | 115298674 | 136 | 7 | 8.30% | 14 |
| 582 | sortilin isoform 1 preproprotein | 17149834 | 92 | 5 | 7.20% | 14 |
| 583 | serine/threonine-protein phosphatase 2A activator isoform b | 29725611 | 37 | 5 | 27% | 14 |
| 584 | ATP synthase subunit e, mitochondrial | 6005717 | 8 | 4 | 46% | 14 |
| 585 | cytochrome c oxidase subunit 6C proprotein | 4758040 | 9 | 4 | 37% | 14 |
| 586 | chitinase domain-containing protein 1 isoform a | 218083142 | 45 | 6 | 21% | 14 |
| 587 | UBX domain-containing protein 11 isoform 1 | 116734681 | 54 | 5 | 16% | 14 |
| 588 | coiled-coil domain-containing protein 40 isoform 1 | 148664197 | 130 | 7 | 6.70% | 14 |
| 589 | ras-related protein Rab-1A isoform 1 | 4758988 | 23 | 3 | 32% | 14 |
| 590 | CD177 antigen precursor | 110735433 | 46 | 5 | 17% | 13 |
| 591 | glucosamine--fructose-6-phosphate aminotransferase [isomerizing] 1 isoform 1 | 347659028 | 79 | 5 | 8.70% | 13 |
| 592 | neutrophil defensin 3 preproprotein | 4885179 | 10 | 4 | 20% | 13 |
| 593 | mesencephalic astrocyte-derived neurotrophic factor precursor | 299523086 | 21 | 6 | 32% | 13 |
| 594 | transmembrane emp24 domain-containing protein 7 precursor | 32996709 | 25 | 4 | 21% | 13 |
| 595 | uncharacterized protein C7orf61 | 51972226 | 24 | 4 | 24% | 13 |
| 596 | stress-induced-phosphoprotein 1 | 5803181 | 63 | 4 | 9.80% | 13 |
| 597 | kelch-like protein 10 | 148664209 | 69 | 7 | 17% | 13 |
| 598 | COP9 signalosome complex subunit 4 | 38373690 | 46 | 4 | 7.90% | 13 |
| 599 | ras-related protein Ral-A precursor | 33946329 | 24 | 3 | 13% | 13 |
| 600 | disintegrin and metalloproteinase domain-containing protein 7 preproprotein | 114326453 | 86 | 6 | 9.20% | 13 |
| 601 | lysozyme-like protein 6 precursor | 9966905 | 17 | 3 | 42% | 13 |
| 602 | izumo sperm-egg fusion protein 2 precursor | 63999117 | 25 | 4 | 14% | 13 |
| 603 | C-Myc-binding protein | 57242777 | 12 | 6 | 65% | 13 |
| 604 | glycerol kinase isoform a | 42794763 | 58 | 2 | 11% | 13 |
| 605 | NAD-dependent malic enzyme, mitochondrial isoform 1 precursor [Homo | 4505145 | 65 | 6 | 19% | 13 |
| 606 | importin subunit alpha-4 . | 34485722 | 58 | 6 | 14% | 13 |
| 607 | cytoplasmic dynein 1 heavy chain 1 | 33350932 | 532 | 8 | 2.20% | 12 |
| 608 | alpha-1-antitrypsin precursor | 50363217 | 47 | 4 | 14% | 12 |
| 609 | ribonuclease inhibitor | 42822872 | 50 | 6 | 14% | 12 |
| 610 | acylamino-acid-releasing enzyme | 23510451 | 81 | 5 | 11% | 12 |
| 611 | annexin A11 | 22165431 | 54 | 4 | 10% | 12 |
| 612 | platelet-activating factor acetylhydrolase precursor | 270133071 | 50 | 4 | 15% | 12 |
| 613 | serine/threonine-protein phosphatase 2A 65 regulatory subunit A alpha isoform | 21361399 | 65 | 6 | 14% | 12 |
| 614 | leukocyte surface antigen CD47 isoform 1 precursor | 4502673 | 35 | 2 | 5.90% | 12 |
| 615 | interferon-inducible GTPase 5 | 10257429 | 50 | 6 | 25% | 12 |
| 616 | 26S proteasome non-ATPase regulatory subunit 12 isoform 1 | 4506221 | 53 | 4 | 13% | 12 |
| 617 | slit homolog 2 protein precursor | 4759146 | 170 | 8 | 7.50% | 12 |
| 618 | tomoregulin-2 precursor | 12383051 | 41 | 5 | 13% | 12 |
| 619 | fragile X mental retardation 1 neighbor protein | 22749199 | 29 | 4 | 20% | 12 |
| 620 | thioredoxin isoform 1 | 50592994 | 12 | 4 | 43% | 12 |
| 621 | malate dehydrogenase, cytoplasmic isoform 1 | 312283701 | 39 | 6 | 27% | 12 |
| 622 | mRNA export factor | 62739173 | 41 | 4 | 16% | 12 |
| 623 | 45 calcium-binding protein isoform 2 precursor | 18699732 | 42 | 5 | 23% | 12 |
| 624 | uncharacterized protein C6orf81 | 31542280 | 41 | 4 | 19% | 12 |
| 625 | 40S ribosomal protein S8 | 4506743 | 24 | 4 | 19% | 12 |
| 626 | histone H2B type 1-M | 4504263 | 14 | 2 | 32% | 12 |
| 627 | cytochrome b-c1 complex subunit 6, mitochondrial | 83627705 | 11 | 4 | 58% | 12 |
| 628 | UPF0733 protein C2orf88 | 110349742 | 11 | 6 | 68% | 12 |
| 629 | translin-associated protein X | 5174731 | 33 | 6 | 33% | 12 |
| 630 | signal peptidase complex catalytic subunit SEC11A | 7657609 | 21 | 4 | 31% | 12 |
| 631 | A disintegrin and metalloproteinase with thrombospondin motifs 1 preproprotein | 50845384 | 105 | 4 | 5.50% | 12 |
| 632 | sulfatase-modifying factor 2 isoform e precursor | 194248090 | 39 | 4 | 14% | 12 |
| 633 | heat shock 70 protein 13 precursor | 48928056 | 52 | 5 | 15% | 12 |
| 634 | acyl-coenzyme A thioesterase 13 isoform 2 | 231567183 | 12 | 4 | 35% | 12 |
| 635 | dynein light chain roadblock-type 2 | 18702323 | 11 | 3 | 34% | 12 |
| 636 | nucleoporin p58/p45 isoform a | 30102928 | 61 | 6 | 13% | 12 |
| 637 | clathrin light chain A isoform a . | 4502899 | 24 | 4 | 22% | 12 |
| 638 | 40S ribosomal protein S19 | 4506695 | 16 | 4 | 30% | 12 |
| 639 | ras-related protein Rab-5C isoform a | 41393614 | 23 | 4 | 24% | 11 |
| 640 | acid ceramidase isoform b | 189011546 | 47 | 5 | 11% | 11 |
| 641 | proteasome subunit beta type-2 isoform 1 | 4506195 | 23 | 6 | 50% | 11 |
| 642 | poly(rC)-binding protein 1 | 222352151 | 37 | 4 | 12% | 11 |
| 643 | ecto-ADP-ribosyltransferase 3 isoform a precursor | 194097380 | 44 | 4 | 12% | 11 |
| 644 | ribose-phosphate pyrophosphokinase 2 isoform 2 | 4506129 | 35 | 5 | 23% | 11 |
| 645 | 3-ketoacyl-CoA thiolase, mitochondrial | 167614485 | 42 | 6 | 25% | 11 |
| 646 | V-type proton ATPase catalytic subunit A | 19913424 | 68 | 6 | 19% | 11 |
| 647 | GLIPR1-like protein 1 precursor | 22749527 | 26 | 3 | 16% | 11 |
| 648 | 40S ribosomal protein S9 | 14141193 | 23 | 3 | 12% | 11 |
| 649 | desmoplakin isoform I | 58530840 | 332 | 7 | 3.00% | 11 |
| 650 | actin-related protein M1 | 221139714 | 41 | 4 | 18% | 11 |
| 651 | dnaJ homolog subfamily A member 4 isoform 2 | 194328760 | 45 | 6 | 18% | 11 |
| 652 | semaphorin-3C precursor | 5454048 | 85 | 3 | 6.10% | 11 |
| 653 | apolipoprotein A-IV precursor | 71773110 | 45 | 5 | 15% | 11 |
| 654 | testis, prostate and placenta-expressed protein isoform 2 precursor | 154759245 | 31 | 5 | 26% | 11 |
| 655 | enoyl-CoA delta isomerase 2, mitochondrial isoform 2 | 260274832 | 44 | 7 | 22% | 11 |
| 656 | stromal cell-derived factor 2-like protein 1 precursor | 56243533 | 24 | 5 | 44% | 11 |
| 657 | actin-like protein 7A | 5729720 | 49 | 4 | 18% | 11 |
| 658 | methionyl-tRNA synthetase, cytoplasmic | 14043022 | 101 | 6 | 8.90% | 11 |
| 659 | apoptosis-inducing factor 1, mitochondrial isoform 2 precursor | 22202629 | 66 | 5 | 11% | 11 |
| 660 | profilin-3 | 71274140 | 15 | 5 | 51% | 11 |
| 661 | transmembrane protein 126A isoform 1 . | 14150017 | 22 | 4 | 34% | 11 |
| 662 | aldose reductase | 4502049 | 36 | 5 | 26% | 10 |
| 663 | glycogen phosphorylase, brain form | 21361370 | 97 | 4 | 4.70% | 10 |
| 664 | superoxide dismutase [Cu-Zn] | 4507149 | 16 | 4 | 60% | 10 |
| 665 | purine nucleoside phosphorylase | 157168362 | 32 | 5 | 24% | 10 |
| 666 | ras-related protein Rab-7a | 34147513 | 23 | 5 | 28% | 10 |
| 667 | carnitine O-acetyltransferase precursor | 21618331 | 71 | 5 | 7.70% | 10 |
| 668 | acyl carrier protein, mitochondrial precursor | 4826852 | 17 | 3 | 12% | 10 |
| 669 | atlastin-3 | 45827806 | 61 | 4 | 14% | 10 |
| 670 | serine/threonine-protein phosphatase 2A catalytic subunit alpha isoform | 4506017 | 36 | 4 | 21% | 10 |
| 671 | ubiquitin thioesterase OTUB1 | 109148508 | 31 | 5 | 24% | 10 |
| 672 | erythrocyte band 7 integral membrane protein isoform a | 38016911 | 32 | 4 | 20% | 10 |
| 673 | serine protease inhibitor Kazal-type 2 precursor | 10863911 | 9 | 2 | 36% | 10 |
| 674 | regenerating islet-derived protein 3-gamma precursor | 38348213 | 19 | 3 | 27% | 10 |
| 675 | casein kinase II subunit beta | 23503295 | 25 | 5 | 25% | 10 |
| 676 | aspartyl-tRNA synthetase, cytoplasmic | 45439306 | 57 | 5 | 9.60% | 10 |
| 677 | septin-7 isoform 2 | 148352329 | 51 | 5 | 19% | 10 |
| 678 | nitrilase homolog 1 isoform 3 | 297632348 | 34 | 4 | 14% | 10 |
| 679 | signal peptidase complex subunit 3 | 11345462 | 20 | 4 | 22% | 10 |
| 680 | transcription elongation factor B polypeptide 2 isoform b | 46276893 | 18 | 5 | 34% | 10 |
| 681 | 40S ribosomal protein S12 | 14277700 | 15 | 5 | 51% | 10 |
| 682 | 26S proteasome non-ATPase regulatory subunit 4 | 5292161 | 41 | 4 | 11% | 10 |
| 683 | mitochondrial import receptor subunit TOM34 | 21361356 | 35 | 4 | 13% | 10 |
| 684 | heat shock 70 protein 4 | 38327039 | 94 | 3 | 4.50% | 10 |
| 685 | acetyl-CoA acetyltransferase, cytosolic | 148539872 | 41 | 4 | 17% | 10 |
| 686 | nuclear migration protein nudC | 5729953 | 38 | 4 | 15% | 10 |
| 687 | eukaryotic translation elongation factor 1 epsilon-1 isoform 1 | 4758862 | 20 | 4 | 34% | 10 |
| 688 | dynein light chain Tctex-type 1 | 5730085 | 12 | 4 | 30% | 10 |
| 689 | metalloproteinase inhibitor 3 precursor | 4507513 | 24 | 5 | 25% | 10 |
| 690 | carbonic anhydrase 2 | 4557395 | 29 | 4 | 20% | 10 |
| 691 | NADH dehydrogenase [ubiquinone] 1 beta subcomplex subunit 7 | 10764847 | 16 | 4 | 26% | 10 |
| 692 | 40S ribosomal protein S17 | 4506693 | 16 | 3 | 43% | 10 |
| 693 | dynein heavy chain 7, axonemal | 151301127 | 461 | 4 | 1.20% | 10 |
| 694 | nucleoporin NUP53 | 31982904 | 35 | 4 | 18% | 10 |
| 695 | COP9 signalosome complex subunit 8 isoform 1 | 5729779 | 23 | 3 | 30% | 10 |
| 696 | diablo homolog, mitochondrial isoform 1 precursor | 9845297 | 27 | 5 | 25% | 10 |
| 697 | ubiquitin thioesterase OTUB2 | 12962939 | 27 | 4 | 18% | 10 |
| 698 | NADH dehydrogenase [ubiquinone] 1 alpha subcomplex subunit 2 isoform 1 | 4505355 | 11 | 4 | 41% | 10 |
| 699 | ADP-ribosylation factor-like protein 3 | 4757774 | 20 | 5 | 45% | 10 |
| 700 | brain protein 44 | 219521872 | 14 | 4 | 39% | 10 |
| 701 | midkine precursor | 4505135 | 16 | 4 | 23% | 9 |
| 702 | F-actin-capping protein subunit beta isoform 1 | 4826659 | 31 | 4 | 13% | 9 |
| 703 | 40S ribosomal protein S3 | 15718687 | 27 | 6 | 35% | 9 |
| 704 | annexin A7 isoform 1 | 4502111 | 50 | 4 | 9.20% | 9 |
| 705 | ras-related C3 botulinum toxin substrate 1 isoform Rac1b | 9845509 | 23 | 5 | 21% | 9 |
| 706 | adenylate kinase domain-containing protein 1 isoform 1 | 237858799 | 221 | 7 | 4.30% | 9 |
| 707 | cystatin-B | 4503117 | 11 | 2 | 34% | 9 |
| 708 | spondin-2 precursor | 6912682 | 36 | 3 | 7.60% | 9 |
| 709 | tropomyosin alpha-3 chain isoform 2 | 24119203 | 29 | 4 | 20% | 9 |
| 710 | ran-specific GTPase-activating protein | 4506407 | 23 | 2 | 10.00% | 9 |
| 711 | ATP synthase subunit delta, mitochondrial precursor | 50345991 | 17 | 4 | 39% | 9 |
| 712 | vitronectin precursor | 88853069 | 54 | 3 | 8.60% | 9 |
| 713 | 40S ribosomal protein S15a | 71772415 | 15 | 4 | 32% | 9 |
| 714 | bifunctional ATP-dependent dihydroxyacetone kinase/FAD-AMP lyase (cyclizing) | 20149621 | 59 | 4 | 11% | 9 |
| 715 | pro-cathepsin H preproprotein | 23110955 | 37 | 2 | 8.40% | 9 |
| 716 | destrin isoform a | 5802966 | 19 | 2 | 17% | 9 |
| 717 | uncharacterized protein C9orf171 | 46409466 | 36 | 5 | 16% | 9 |
| 718 | protein TSC21 | 22749357 | 21 | 2 | 13% | 9 |
| 719 | aflatoxin B1 aldehyde reductase member 2 | 41327764 | 40 | 4 | 16% | 9 |
| 720 | ubiquitin carboxyl-terminal hydrolase 7 | 150378533 | 128 | 6 | 6.70% | 9 |
| 721 | peptidyl-prolyl cis-trans isomerase F, mitochondrial precursor | 5031987 | 22 | 4 | 35% | 9 |
| 722 | 3-mercaptopyruvate sulfurtransferase isoform 2 | 61835204 | 33 | 3 | 19% | 9 |
| 723 | WD repeat-containing protein 65 isoform a | 345199335 | 149 | 8 | 7.10% | 9 |
| 724 | 60S ribosomal protein L12 | 4506597 | 18 | 3 | 25% | 9 |
| 725 | prenylated Rab acceptor protein 1 | 222144309 | 21 | 3 | 18% | 9 |
| 726 | translocon-associated protein subunit delta isoform 3 precursor | 325301078 | 20 | 3 | 24% | 9 |
| 727 | dnaJ homolog subfamily B member 6 isoform b | 4885495 | 27 | 2 | 12% | 9 |
| 728 | beta-defensin 126 preproprotein . | 13624333 | 12 | 2 | 8.10% | 9 |
| 729 | leucine-rich repeat-containing protein 23 isoform a | 42542396 | 40 | 4 | 19% | 9 |
| 730 | brain protein 44-like protein 2 | 306922396 | 15 | 3 | 34% | 9 |
| 731 | apolipoprotein O precursor | 13129148 | 22 | 3 | 19% | 9 |
| 732 | tenascin precursor | 153946395 | 241 | 6 | 3.70% | 9 |
| 733 | kita-kyushu lung cancer antigen 1 . | 63025190 | 13 | 2 | 19% | 9 |
| 734 | peptidyl-prolyl cis-trans isomerase FKBP2 precursor . | 17149842 | 16 | 5 | 32% | 9 |
| 735 | ubiquitin carboxyl-terminal hydrolase 14 isoform b | 82880645 | 52 | 4 | 7.00% | 8 |
| 736 | ferritin heavy chain | 56682959 | 21 | 3 | 23% | 8 |
| 737 | alcohol dehydrogenase class-3 | 71565154 | 40 | 4 | 11% | 8 |
| 738 | inositol monophosphatase 1 isoform 2 | 221625487 | 37 | 3 | 13% | 8 |
| 739 | calpain-1 catalytic subunit | 311893363 | 82 | 5 | 8.00% | 8 |
| 740 | lysosome membrane protein 2 isoform 1 precursor | 5031631 | 54 | 4 | 14% | 8 |
| 741 | apolipoprotein D precursor | 4502163 | 21 | 4 | 21% | 8 |
| 742 | 26S proteasome non-ATPase regulatory subunit 6 | 7661914 | 46 | 4 | 13% | 8 |
| 743 | glycerophosphodiester phosphodiesterase 1 | 7706617 | 38 | 4 | 19% | 8 |
| 744 | NADH dehydrogenase [ubiquinone] 1 alpha subcomplex subunit 8 | 7657369 | 20 | 3 | 23% | 8 |
| 745 | ADP-ribosyl cyclase 1 | 38454326 | 34 | 2 | 8.70% | 8 |
| 746 | importin-4 | 62460637 | 119 | 5 | 5.80% | 8 |
| 747 | 60S ribosomal protein L7a | 4506661 | 30 | 3 | 15% | 8 |
| 748 | UTP--glucose-1-phosphate uridylyltransferase isoform b | 48255968 | 56 | 4 | 10% | 8 |
| 749 | dnaJ homolog subfamily C member 3 precursor | 5453980 | 58 | 5 | 18% | 8 |
| 750 | puromycin-sensitive aminopeptidase | 158937236 | 103 | 4 | 2.70% | 8 |
| 751 | 60S ribosomal protein L18 | 4506607 | 22 | 3 | 13% | 8 |
| 752 | cytochrome c oxidase subunit 7A2, mitochondrial precursor | 262118227 | 13 | 3 | 22% | 8 |
| 753 | nuclear pore complex protein Nup205 | 57634534 | 228 | 6 | 3.60% | 8 |
| 754 | cytochrome b5 type B | 83921614 | 17 | 3 | 17% | 8 |
| 755 | protein FAM71A | 282721094 | 63 | 4 | 8.40% | 8 |
| 756 | dynein light chain 1, axonemal isoform 1 | 164607156 | 22 | 4 | 19% | 8 |
| 757 | lipase member I | 39752679 | 55 | 3 | 6.70% | 8 |
| 758 | left-right determination factor 1 preproprotein | 10337603 | 41 | 3 | 11% | 8 |
| 759 | 3-oxoacyl-[acyl-carrier-protein] synthase, mitochondrial isoform 1 | 8923559 | 49 | 3 | 15% | 8 |
| 760 | glutathione S-transferase omega-2 isoform 1 | 38016131 | 28 | 5 | 22% | 8 |
| 761 | myotrophin | 21956645 | 13 | 3 | 43% | 8 |
| 762 | thioredoxin domain-containing protein 17 | 14249348 | 14 | 3 | 27% | 8 |
| 763 | ras-related protein Rab-6A isoform b | 38679888 | 24 | 3 | 16% | 8 |
| 764 | nucleosome assembly protein 1-like 1 | 4758756 | 45 | 3 | 13% | 8 |
| 765 | actin-like protein 9 | 194097462 | 46 | 4 | 13% | 8 |
| 766 | protein arginine N-methyltransferase 5 isoform a | 20070220 | 73 | 5 | 9.70% | 8 |
| 767 | deoxyuridine 5'-triphosphate nucleotidohydrolase, mitochondrial isoform 1 precursor | 70906441 | 27 | 4 | 21% | 8 |
| 768 | Sjoegren syndrome nuclear autoantigen 1 | 189571687 | 14 | 4 | 27% | 8 |
| 769 | NADH dehydrogenase [ubiquinone] 1 beta subcomplex subunit 8, mitochondrial precursor | 4826854 | 22 | 3 | 21% | 8 |
| 770 | voltage-dependent calcium channel subunit alpha-2/delta-2 isoform c | 291290994 | 130 | 6 | 6.80% | 8 |
| 771 | V-type proton ATPase subunit B, brain isoform | 19913428 | 57 | 4 | 14% | 8 |
| 772 | transcriptional activator protein Pur-alpha | 5032007 | 35 | 3 | 15% | 8 |
| 773 | peptidase inhibitor 15 preproprotein | 7705676 | 29 | 4 | 19% | 8 |
| 774 | integral membrane protein 2B | 11527402 | 30 | 3 | 21% | 8 |
| 775 | bovine seminal plasma protein homolog 1 precursor | 190358548 | 16 | 3 | 25% | 8 |
| 776 | succinyl-CoA ligase [ADP/GDP-forming] subunit alpha, mitochondrial | 109452591 | 36 | 2 | 8.70% | 8 |
| 777 | isovaleryl-CoA dehydrogenase, mitochondrial isoform 1 precursor | 226958412 | 47 | 3 | 16% | 8 |
| 778 | splicing factor 3A subunit 3 . | 5803167 | 59 | 2 | 4.40% | 8 |
| 779 | outer dense fiber protein 3B . | 116292180 | 27 | 3 | 12% | 8 |
| 780 | ATP-citrate synthase isoform 1 | 38569421 | 121 | 6 | 6.40% | 7 |
| 781 | UPF0577 protein KIAA1324 precursor | 38569482 | 111 | 4 | 3.80% | 7 |
| 782 | homogentisate 1,2-dioxygenase | 115527117 | 50 | 4 | 16% | 7 |
| 783 | proactivator polypeptide isoform a preproprotein | 11386147 | 58 | 3 | 5.30% | 7 |
| 784 | nucleophosmin isoform 1 | 10835063 | 33 | 3 | 24% | 7 |
| 785 | gamma-glutamyl hydrolase precursor | 4503987 | 36 | 3 | 17% | 7 |
| 786 | transmembrane emp24 domain-containing protein 2 precursor | 5803149 | 23 | 2 | 18% | 7 |
| 787 | fumarylacetoacetate hydrolase domain-containing protein 2B | 40786394 | 35 | 3 | 21% | 7 |
| 788 | ferritin light chain | 20149498 | 20 | 3 | 23% | 7 |
| 789 | 60S ribosomal protein L14 | 78000181 | 23 | 3 | 16% | 7 |
| 790 | proteasome activator complex subunit 4 | 163644283 | 211 | 6 | 3.50% | 7 |
| 791 | NADH-cytochrome b5 reductase 3 isoform 2 | 193794826 | 32 | 3 | 17% | 7 |
| 792 | nucleosome assembly protein 1-like 4 | 5174613 | 43 | 3 | 9.60% | 7 |
| 793 | transketolase-like protein 1 isoform b | 225637461 | 65 | 3 | 6.40% | 7 |
| 794 | UPF0740 protein C1orf192 | 63029930 | 19 | 4 | 27% | 7 |
| 795 | ATP synthase subunit epsilon, mitochondrial | 5901896 | 6 | 3 | 45% | 7 |
| 796 | sperm-associated antigen 16 protein isoform 1 | 70909324 | 71 | 5 | 10% | 7 |
| 797 | dipeptidase 1 precursor | 4758190 | 46 | 3 | 13% | 7 |
| 798 | EF-hand calcium-binding domain-containing protein 1 isoform a | 13375787 | 24 | 3 | 23% | 7 |
| 799 | BAG family molecular chaperone regulator 5 isoform b | 6631077 | 51 | 4 | 16% | 7 |
| 800 | HD domain-containing protein 2 | 116875826 | 23 | 3 | 24% | 7 |
| 801 | dihydropteridine reductase | 208973246 | 26 | 3 | 23% | 7 |
| 802 | heterogeneous nuclear ribonucleoprotein K isoform b | 14165435 | 51 | 4 | 13% | 7 |
| 803 | SPARC-related modular calcium-binding protein 2 isoform 1 precursor | 24308277 | 51 | 3 | 12% | 7 |
| 804 | synaptogyrin-2 | 4759202 | 25 | 3 | 13% | 7 |
| 805 | glycyl-tRNA synthetase precursor | 116805340 | 83 | 4 | 8.40% | 7 |
| 806 | BAG family molecular chaperone regulator 2 | 4757834 | 24 | 3 | 20% | 7 |
| 807 | protein FAM162A | 49355721 | 17 | 2 | 20% | 7 |
| 808 | PREDICTED: hypothetical protein LOC728597 | 341914388 | 72 | 4 | 6.70% | 7 |
| 809 | aminoacyl tRNA synthase complex-interacting multifunctional protein 1 isoform b precursor | 215490011 | 37 | 3 | 17% | 7 |
| 810 | protein DPCD | 39930355 | 23 | 3 | 15% | 7 |
| 811 | transmembrane protein 205 | 63055043 | 21 | 2 | 16% | 7 |
| 812 | thiosulfate sulfurtransferase/rhodanese-like domain-containing protein 1 isoform 1 | 163965377 | 13 | 3 | 21% | 7 |
| 813 | ethanolamine-phosphate cytidylyltransferase isoform 1 | 296841136 | 46 | 3 | 14% | 7 |
| 814 | ubiquitin-conjugating enzyme E2 L3 | 4507789 | 18 | 3 | 30% | 7 |
| 815 | desmoglein-1 preproprotein | 119703744 | 114 | 6 | 9.70% | 7 |
| 816 | mannosyl-oligosaccharide glucosidase isoform 1 | 149999606 | 92 | 3 | 4.70% | 7 |
| 817 | uncharacterized protein C9orf135 | 58219541 | 26 | 3 | 21% | 7 |
| 818 | high mobility group protein B2 . | 194688133 | 24 | 3 | 19% | 7 |
| 819 | cysteine desulfurase, mitochondrial isoform a | 32307132 | 50 | 4 | 14% | 7 |
| 820 | ATP synthase-coupling factor 6, mitochondrial isoform a precursor | 18644883 | 13 | 3 | 46% | 7 |
| 821 | nucleobindin-1 precursor | 20070228 | 54 | 3 | 12% | 7 |
| 822 | mitochondrial fission 1 protein . | 151108473 | 17 | 3 | 26% | 7 |
| 823 | azurocidin preproprotein | 11342670 | 27 | 2 | 9.60% | 6 |
| 824 | calicin | 169636428 | 67 | 3 | 6.10% | 6 |
| 825 | exportin-2 | 29029559 | 110 | 3 | 4.60% | 6 |
| 826 | 60S acidic ribosomal protein P1 isoform 1 | 4506669 | 12 | 2 | 57% | 6 |
| 827 | cytochrome b5 isoform 1 | 41281768 | 15 | 3 | 36% | 6 |
| 828 | phosphate carrier protein, mitochondrial isoform b precursor | 47132595 | 40 | 2 | 5.50% | 6 |
| 829 | 60S ribosomal protein L11 isoform 1 | 15431290 | 20 | 2 | 13% | 6 |
| 830 | beta-hexosaminidase subunit beta preproprotein | 4504373 | 63 | 4 | 9.50% | 6 |
| 831 | platelet-activating factor acetylhydrolase IB subunit beta isoform a | 4505585 | 26 | 2 | 12% | 6 |
| 832 | thioredoxin domain-containing protein 2 isoform 2 | 148727319 | 60 | 3 | 4.90% | 6 |
| 833 | 60S ribosomal protein L7 | 15431301 | 29 | 4 | 18% | 6 |
| 834 | matrilin-2 isoform a precursor | 62548860 | 107 | 3 | 4.40% | 6 |
| 835 | glyoxylate reductase/hydroxypyruvate reductase | 6912396 | 36 | 3 | 15% | 6 |
| 836 | 72 type IV collagenase isoform a preproprotein | 11342666 | 74 | 2 | 5.00% | 6 |
| 837 | antileukoproteinase precursor | 4507065 | 14 | 2 | 15% | 6 |
| 838 | trafficking protein particle complex subunit 3 | 7656926 | 20 | 2 | 13% | 6 |
| 839 | tripeptidyl-peptidase 1 preproprotein | 5729770 | 61 | 4 | 14% | 6 |
| 840 | transketolase-like protein 2 . | 133778974 | 68 | 2 | 5.60% | 6 |
| 841 | dynein heavy chain 12, axonemal isoform 1 | 194440727 | 357 | 2 | 1.00% | 6 |
| 842 | mitochondrial dicarboxylate carrier | 20149598 | 31 | 4 | 19% | 6 |
| 843 | biglycan preproprotein | 4502403 | 42 | 3 | 7.60% | 6 |
| 844 | prostate and testis expressed protein 2 precursor | 47086459 | 13 | 2 | 27% | 6 |
| 845 | chloride intracellular channel protein 4 | 7330335 | 29 | 3 | 13% | 6 |
| 846 | protein CutA isoform 1 | 62198241 | 21 | 2 | 21% | 6 |
| 847 | prohibitin-2 isoform 2 | 6005854 | 33 | 3 | 13% | 6 |
| 848 | glutathione S-transferase Mu 1 isoform 1 | 23065544 | 26 | 2 | 14% | 6 |
| 849 | tubulin polymerization-promoting protein family member 2 | 226491350 | 19 | 3 | 32% | 6 |
| 850 | probable inactive serine protease 37 isoform 1 precursor | 285394164 | 26 | 2 | 8.90% | 6 |
| 851 | sperm-associated antigen 11B isoform A preproprotein | 7706551 | 11 | 2 | 19% | 6 |
| 852 | putative peptidyl-tRNA hydrolase PTRHD1 | 61966781 | 16 | 3 | 25% | 6 |
| 853 | ras-related protein Rab-5B isoform 1 | 354725902 | 24 | 2 | 18% | 6 |
| 854 | lysosomal Pro-X carboxypeptidase isoform 1 preproprotein | 4826940 | 56 | 2 | 7.30% | 6 |
| 855 | NADH dehydrogenase [ubiquinone] iron-sulfur protein 4, mitochondrial precursor | 4505369 | 20 | 2 | 14% | 6 |
| 856 | UPF0587 protein C1orf123 | 8923541 | 18 | 2 | 25% | 6 |
| 857 | syntaxin-12 | 28933465 | 32 | 3 | 14% | 6 |
| 858 | 6-phosphogluconolactonase | 6912586 | 28 | 3 | 21% | 6 |
| 859 | agouti-related protein precursor | 4501995 | 14 | 2 | 14% | 6 |
| 860 | major prion protein preproprotein . | 122056623 | 28 | 2 | 9.50% | 6 |
| 861 | dolichyl-diphosphooligosaccharide--protein glycosyltransferase subunit DAD1 | 4503253 | 12 | 2 | 19% | 6 |
| 862 | non-specific lipid-transfer protein isoform 1 proprotein | 19923233 | 59 | 2 | 3.50% | 6 |
| 863 | V-type proton ATPase subunit d 1 | 19913432 | 40 | 2 | 6.80% | 6 |
| 864 | transmembrane emp24 domain-containing protein 1 precursor | 5803040 | 25 | 2 | 11% | 6 |
| 865 | ADP-ribosylation factor 6 | 4502211 | 20 | 2 | 12% | 6 |
| 866 | endoplasmic reticulum lectin 1 isoform 2 precursor . | 188528696 | 52 | 4 | 19% | 6 |
| 867 | alpha-galactosidase A precursor | 4504009 | 49 | 2 | 7.00% | 6 |
| 868 | V-type proton ATPase subunit E 1 isoform a | 4502317 | 26 | 2 | 12% | 6 |
| 869 | uncharacterized protein C19orf18 precursor | 22748999 | 24 | 2 | 9.80% | 6 |
| 870 | ADP-ribosylation factor-like protein 8B | 8922601 | 22 | 2 | 18% | 6 |
| 871 | secretoglobin family 1D member 2 precursor | 5729909 | 10 | 2 | 22% | 6 |
| 872 | uncharacterized protein C22orf43 | 56118955 | 25 | 2 | 9.60% | 6 |
| 873 | 60S ribosomal protein L31 isoform 3 | 153252132 | 14 | 2 | 19% | 6 |
| 874 | iron-sulfur cluster assembly enzyme ISCU, mitochondrial isoform ISCU2 precursor | 56699456 | 18 | 2 | 10% | 6 |
| 875 | 40S ribosomal protein S24 isoform a | 14916501 | 15 | 2 | 21% | 6 |
| 876 | retinoic acid receptor responder protein 1 isoform 2 precursor | 46255041 | 26 | 2 | 9.60% | 6 |
| 877 | phenylalanyl-tRNA synthetase alpha chain | 4758340 | 58 | 3 | 12% | 6 |
| 878 | fibrinogen-like protein 1 precursor . | 42544189 | 36 | 2 | 7.40% | 6 |
| 879 | titin isoform N2-A | 291045225 | 3713 | 3 | 0.06% | 5 |
| 880 | glycerol-3-phosphate dehydrogenase 1-like protein | 24307999 | 38 | 2 | 6.00% | 5 |
| 881 | complement decay-accelerating factor isoform 2 precursor | 168693643 | 49 | 3 | 9.10% | 5 |
| 882 | beta-microseminoprotein isoform a precursor | 4557036 | 13 | 3 | 18% | 5 |
| 883 | cathepsin D preproprotein | 4503143 | 45 | 3 | 12% | 5 |
| 884 | CD63 antigen isoform A | 4502679 | 26 | 2 | 5.00% | 5 |
| 885 | cystatin-S precursor | 4503109 | 16 | 3 | 28% | 5 |
| 886 | lysosome-associated membrane glycoprotein 2 isoform C precursor | 169790833 | 45 | 2 | 4.10% | 5 |
| 887 | spectrin alpha chain, brain isoform 2 | 154759259 | 285 | 3 | 1.90% | 5 |
| 888 | phospholipase A1 member A isoform 2 precursor | 332688256 | 48 | 2 | 7.30% | 5 |
| 889 | UPF0468 protein C16orf80 | 8392875 | 23 | 3 | 19% | 5 |
| 890 | sperm-associated antigen 11B isoform D preproprotein | 126131097 | 15 | 2 | 17% | 5 |
| 891 | acyl-protein thioesterase 1 | 5453722 | 25 | 3 | 21% | 5 |
| 892 | S-adenosylmethionine synthase isoform type-2 | 5174529 | 44 | 2 | 6.60% | 5 |
| 893 | filaggrin-2 | 62122917 | 248 | 3 | 2.80% | 5 |
| 894 | spermatogenesis-associated protein 19, mitochondrial precursor | 28376652 | 19 | 3 | 26% | 5 |
| 895 | thioredoxin-dependent peroxide reductase, mitochondrial isoform b | 32483377 | 26 | 3 | 16% | 5 |
| 896 | zona pellucida-binding protein 2 isoform 1 precursor | 84875535 | 36 | 2 | 7.90% | 5 |
| 897 | cytosolic 5'-nucleotidase 1B isoform 4 | 312283642 | 71 | 2 | 3.00% | 5 |
| 898 | renin receptor precursor | 15011918 | 39 | 3 | 13% | 5 |
| 899 | sialidase-1 precursor | 4557791 | 45 | 2 | 12% | 5 |
| 900 | 60S ribosomal protein L13a | 6912634 | 24 | 2 | 10% | 5 |
| 901 | 15 selenoprotein isoform 1 precursor | 42741648 | 18 | 3 | 17% | 5 |
| 902 | proteasome activator complex subunit 2 . | 30410792 | 27 | 2 | 13% | 5 |
| 903 | guanine nucleotide-binding protein subunit beta-2-like 1 | 5174447 | 35 | 2 | 8.50% | 5 |
| 904 | vesicle-trafficking protein SEC22b precursor | 94429050 | 25 | 2 | 11% | 5 |
| 905 | coiled-coil-helix-coiled-coil-helix domain-containing protein 6 | 14150134 | 26 | 3 | 23% | 5 |
| 906 | S-phase kinase-associated protein 1 isoform b | 25777713 | 19 | 3 | 18% | 5 |
| 907 | metaxin-2 | 5729937 | 30 | 2 | 8.00% | 5 |
| 908 | prostate and testis expressed protein 4 | 221554530 | 11 | 3 | 30% | 5 |
| 909 | secreted frizzled-related protein 1 precursor . | 56117838 | 35 | 3 | 12% | 5 |
| 910 | protein EAN57 isoform 1 | 255759947 | 31 | 2 | 6.80% | 5 |
| 911 | minor histocompatibility antigen H13 isoform 1 | 23308607 | 41 | 2 | 9.50% | 5 |
| 912 | ATP synthase subunit s, mitochondrial isoform a precursor [Homo | 51558774 | 25 | 3 | 13% | 5 |
| 913 | coiled-coil domain-containing protein 151 | 117553613 | 69 | 5 | 11% | 5 |
| 914 | glypican-3 isoform 1 precursor | 257471006 | 68 | 3 | 5.30% | 5 |
| 915 | NADH dehydrogenase [ubiquinone] iron-sulfur protein 5 | 4758790 | 13 | 2 | 18% | 5 |
| 916 | thioredoxin domain-containing protein 12 precursor | 7705696 | 19 | 2 | 14% | 5 |
| 917 | mitochondrial 2-oxoglutarate/malate carrier protein isoform 1 | 21361114 | 34 | 3 | 13% | 5 |
| 918 | protein FAM3B isoform a precursor | 46255030 | 26 | 2 | 8.10% | 5 |
| 919 | astrocytic phosphoprotein PEA-15 | 4505705 | 15 | 2 | 17% | 5 |
| 920 | coiled-coil domain-containing protein 42A isoform 1 | 226494053 | 38 | 2 | 11% | 5 |
| 921 | uncharacterized protein C7orf72 | 332634960 | 50 | 2 | 9.80% | 5 |
| 922 | cornulin | 7706635 | 54 | 2 | 5.10% | 5 |
| 923 | glutaredoxin-3 | 95113651 | 37 | 3 | 19% | 5 |
| 924 | arrestin domain-containing protein 5 . | 122937478 | 38 | 3 | 11% | 5 |
| 925 | mimitin, mitochondrial | 29789409 | 20 | 2 | 24% | 5 |
| 926 | protein-tyrosine phosphatase mitochondrial 1 isoform 1 | 148224884 | 23 | 3 | 21% | 5 |
| 927 | DAZ-associated protein 1 isoform b . | 25470886 | 43 | 3 | 14% | 5 |
| 928 | 60S ribosomal protein L26 | 4506621 | 17 | 3 | 21% | 5 |
| 929 | coiled-coil domain-containing protein 90B, mitochondrial precursor | 20149663 | 30 | 2 | 15% | 5 |
| 930 | maleylacetoacetate isomerase isoform 1 | 22202624 | 24 | 2 | 10% | 5 |
| 931 | uncharacterized protein C2orf61 isoform 1 . | 254675143 | 28 | 2 | 13% | 5 |
| 932 | sphingomyelin phosphodiesterase 4 isoform 2 | 102467481 | 98 | 3 | 4.30% | 5 |
| 933 | gelsolin isoform a precursor | 4504165 | 86 | 2 | 3.60% | 4 |
| 934 | UPF0556 protein C19orf10 precursor | 33457348 | 19 | 2 | 12% | 4 |
| 935 | profilin-2 isoform a | 16753215 | 15 | 2 | 21% | 4 |
| 936 | elongation factor 1-beta | 4503477 | 25 | 2 | 24% | 4 |
| 937 | ras-related protein Rap-1b isoform 1 precursor | 7661678 | 21 | 2 | 13% | 4 |
| 938 | sarcoplasmic/endoplasmic reticulum calcium ATPase 2 isoform b | 24638454 | 115 | 3 | 3.90% | 4 |
| 939 | dynactin subunit 3 isoform 1 | 6005745 | 21 | 2 | 9.10% | 4 |
| 940 | carnitine O-palmitoyltransferase 1, muscle isoform isoform a | 223468678 | 88 | 4 | 3.60% | 4 |
| 941 | acid sphingomyelinase-like phosphodiesterase 3b isoform 1 precursor | 57242798 | 51 | 2 | 5.70% | 4 |
| 942 | izumo sperm-egg fusion protein 1 precursor | 194097475 | 39 | 2 | 9.10% | 4 |
| 943 | 14 phosphohistidine phosphatase isoform 3 | 24475861 | 14 | 2 | 26% | 4 |
| 944 | fatty-acid amide hydrolase 1 | 166795287 | 63 | 2 | 7.80% | 4 |
| 945 | aquaporin-5 | 4502183 | 28 | 2 | 9.80% | 4 |
| 946 | protein NDRG3 isoform a | 14165266 | 41 | 3 | 15% | 4 |
| 947 | low molecular weight phosphotyrosine protein phosphatase isoform c | 4757714 | 18 | 2 | 17% | 4 |
| 948 | nucleoside diphosphate kinase 7 isoform a | 7019465 | 42 | 2 | 6.60% | 4 |
| 949 | protein OS-9 isoform 1 precursor | 5803109 | 76 | 2 | 4.90% | 4 |
| 950 | protamine-2 | 68989267 | 13 | 2 | 27% | 4 |
| 951 | calcium-binding mitochondrial carrier protein Aralar1 | 21361103 | 75 | 2 | 3.70% | 4 |
| 952 | transducin beta-like protein 2 precursor | 7549793 | 50 | 2 | 6.90% | 4 |
| 953 | dickkopf-like protein 1 isoform 2 precursor | 308818218 | 24 | 3 | 15% | 4 |
| 954 | thioredoxin-like protein 1 | 4759274 | 32 | 2 | 10% | 4 |
| 955 | growth hormone-inducible transmembrane protein | 118200356 | 37 | 2 | 6.40% | 4 |
| 956 | thiamine-triphosphatase | 13236577 | 26 | 2 | 12% | 4 |
| 957 | beta-defensin 105 precursor | 103485141 | 9 | 2 | 27% | 4 |
| 958 | ubiquitin-conjugating enzyme E2 N | 4507793 | 17 | 2 | 17% | 4 |
| 959 | septin-4 isoform 3 | 17986247 | 53 | 2 | 5.00% | 4 |
| 960 | eukaryotic translation initiation factor 3 subunit M | 23397429 | 43 | 2 | 13% | 4 |
| 961 | actin-related protein 2/3 complex subunit 4 isoform c | 311771647 | 22 | 2 | 18% | 4 |
| 962 | electron transfer flavoprotein-ubiquinone oxidoreductase, mitochondrial precursor | 119703746 | 68 | 2 | 5.20% | 4 |
| 963 | calcium-regulated heat stable protein 1 | 109715858 | 16 | 2 | 18% | 4 |
| 964 | glutaminyl-peptide cyclotransferase precursor | 6912618 | 41 | 2 | 13% | 4 |
| 965 | SPRY domain-containing protein 7 isoform 1 | 20531765 | 22 | 2 | 13% | 4 |
| 966 | coiled-coil domain-containing protein 164 | 217416374 | 87 | 4 | 6.20% | 4 |
| 967 | uncharacterized protein C19orf71 | 207442677 | 24 | 2 | 14% | 4 |
| 968 | UPF0573 protein C2orf70 | 157502167 | 23 | 2 | 17% | 4 |
| 969 | tissue alpha-L-fucosidase precursor . | 119360348 | 54 | 2 | 6.00% | 4 |
| 970 | 4F2 cell-surface antigen heavy chain isoform c | 65506891 | 68 | 2 | 4.10% | 4 |
| 971 | protein transport protein Sec61 subunit beta | 5803165 | 10 | 2 | 26% | 4 |
| 972 | rhabdoid tumor deletion region protein 1 | 7657530 | 39 | 3 | 16% | 4 |
| 973 | serine/threonine-protein phosphatase 6 catalytic subunit isoform a | 183603929 | 39 | 2 | 8.20% | 4 |
| 974 | procollagen-lysine,2-oxoglutarate 5-dioxygenase 1 precursor | 32307144 | 84 | 2 | 3.20% | 4 |
| 975 | ubiquitin-conjugating enzyme E2 variant 1 isoform d | 73765546 | 16 | 2 | 14% | 4 |
| 976 | intraflagellar transport protein 27 homolog isoform 2 | 6857824 | 20 | 2 | 13% | 4 |
| 977 | prefoldin subunit 2 | 12408675 | 17 | 2 | 17% | 4 |
| 978 | COP9 signalosome complex subunit 7b | 12232385 | 30 | 2 | 9.10% | 4 |
| 979 | eukaryotic translation initiation factor 3 subunit B | 33239445 | 92 | 3 | 4.40% | 4 |
| 980 | carboxylesterase 5A isoform 1 precursor . | 219521907 | 64 | 2 | 5.40% | 4 |
| 981 | protein lin-7 homolog A | 4759306 | 26 | 4 | 20% | 4 |
| 982 | heat shock protein beta-11 | 118343647 | 16 | 2 | 28% | 4 |
| 983 | abhydrolase domain-containing protein 16A isoform a . | 15100151 | 63 | 2 | 5.40% | 4 |
| 984 | tumor protein p53-inducible protein 11 | 33695117 | 21 | 2 | 13% | 4 |
| 985 | synaptosomal-associated protein 29 | 4759154 | 29 | 2 | 8.50% | 4 |
| 986 | cob(I)yrinic acid a,c-diamide adenosyltransferase, mitochondrial precursor | 16418349 | 27 | 2 | 10% | 4 |
| 987 | selenoprotein P isoform 2 . | 148277022 | 46 | 2 | 7.80% | 4 |
| 988 | inactive serine protease 54 precursor | 122937420 | 44 | 2 | 9.40% | 4 |
| 989 | 60S ribosomal protein L13 isoform 1 | 15431295 | 24 | 2 | 9.50% | 3 |
| 990 | ubiquitin carboxyl-terminal hydrolase 5 isoform 2 | 148727247 | 93 | 2 | 4.60% | 3 |
| 991 | cysteine-rich secretory protein 2 precursor | 215490018 | 27 | 2 | 4.90% | 3 |
| 992 | 40S ribosomal protein S11 | 4506681 | 18 | 2 | 9.50% | 3 |
| 993 | eukaryotic translation initiation factor 3 subunit A | 4503509 | 167 | 3 | 2.30% | 3 |
| 994 | endoplasmic reticulum-Golgi intermediate compartment protein 1 | 72534712 | 33 | 3 | 17% | 3 |
| 995 | retinol dehydrogenase 11 isoform 1 precursor | 166795268 | 35 | 2 | 7.90% | 3 |
| 996 | 60S ribosomal protein L30 | 4506631 | 13 | 2 | 24% | 3 |
| 997 | sperm flagellar protein 2 isoform 1 | 145699133 | 210 | 2 | 1.20% | 3 |
| 998 | nicotinamide phosphoribosyltransferase precursor | 5031977 | 56 | 2 | 10% | 3 |
| 999 | retinal dehydrogenase 1 | 21361176 | 55 | 2 | 4.40% | 3 |
| 1000 | sulfhydryl oxidase 1 isoform a precursor | 13325075 | 83 | 3 | 4.70% | 3 |
| 1001 | 60S ribosomal protein L23 | 4506605 | 15 | 2 | 16% | 3 |
| 1002 | dynein intermediate chain 1, axonemal | 6912338 | 79 | 2 | 5.90% | 3 |
| 1003 | S-formylglutathione hydrolase | 33413400 | 31 | 2 | 16% | 3 |
| 1004 | eukaryotic translation initiation factor 6 isoform a | 4504771 | 27 | 2 | 13% | 3 |
| 1005 | bifunctional purine biosynthesis protein PURH | 20127454 | 65 | 2 | 7.10% | 3 |
| 1006 | protein BRICK1 | 27544939 | 9 | 2 | 28% | 3 |
| 1007 | 60S ribosomal protein L22 proprotein | 4506613 | 15 | 2 | 19% | 3 |
| 1008 | dynein intermediate chain 2, axonemal isoform 1 . | 217416452 | 69 | 2 | 3.80% | 3 |
| 1009 | prostaglandin E synthase 3 | 23308579 | 19 | 2 | 20% | 3 |
| 1010 | spermatid-associated protein | 22749425 | 52 | 2 | 6.90% | 3 |
| 1011 | cell cycle control protein 50A isoform 1 | 8922720 | 41 | 2 | 5.80% | 3 |
| 1012 | neuropilin-1 isoform a precursor | 182508169 | 103 | 3 | 5.60% | 3 |
| 1013 | DNA damage-binding protein 1 | 148529014 | 127 | 2 | 2.50% | 3 |
| 1014 | glutaminyl-tRNA synthetase | 4826960 | 88 | 2 | 3.10% | 3 |
| 1015 | solute carrier family 2, facilitated glucose transporter member 5 isoform 1 | 4507013 | 55 | 2 | 6.40% | 3 |
| 1016 | 26S proteasome non-ATPase regulatory subunit 5 | 4826952 | 56 | 2 | 5.60% | 3 |
| 1017 | nodal modulator 3 precursor . | 51944969 | 134 | 2 | 2.00% | 3 |
| 1018 | methylosome protein 50 . | 13129110 | 37 | 3 | 11% | 3 |
| 1019 | uncharacterized protein C2orf77 | 146260271 | 66 | 2 | 3.80% | 3 |
| 1020 | cytochrome b5 domain-containing protein 1 | 40255060 | 27 | 3 | 21% | 3 |
| 1021 | uncharacterized protein C2orf74 isoform 1 . | 221139892 | 22 | 2 | 11% | 3 |
| 1022 | COP9 signalosome complex subunit 7a | 7705330 | 30 | 2 | 8.70% | 3 |
| 1023 | centrin-1 . | 4757974 | 20 | 3 | 19% | 3 |
| 1024 | grpE protein homolog 1, mitochondrial precursor . | 24308295 | 24 | 2 | 10% | 3 |
| 1025 | cysteine--tRNA ligase, cytoplasmic isoform c . | 62240992 | 95 | 2 | 3.20% | 3 |
| 1026 | RIB43A-like with coiled-coils protein 2 | 241666448 | 45 | 3 | 11% | 3 |
| 1027 | uncharacterized protein LOC145788 | 311771669 | 14 | 2 | 27% | 3 |
| 1028 | mitochondrial import receptor subunit TOM40 homolog . | 5174723 | 38 | 2 | 16% | 3 |
| 1029 | polypeptide N-acetylgalactosaminyltransferase 3 . | 153266878 | 73 | 2 | 4.40% | 3 |
| 1030 | protein disulfide-isomerase-like protein of the testis precursor | 28372543 | 67 | 3 | 6.30% | 3 |
| 1031 | tetraspanin-16 isoform 1 . | 6912726 | 26 | 2 | 8.20% | 3 |
| 1032 | peptidyl-prolyl cis-trans isomerase-like 6 isoform 1 [Homo | 27735045 | 35 | 2 | 15% | 3 |
| 1033 | GTP:AMP phosphotransferase AK3, mitochondrial isoform a [Homo | 19923437 | 26 | 2 | 8.40% | 3 |
| 1034 | ADP-ribosylation factor-like protein 1 | 4502227 | 20 | 2 | 15% | 3 |
| 1035 | mitochondrial-processing peptidase subunit alpha isoform 1 | 24308013 | 58 | 2 | 7.20% | 3 |
| 1036 | ectonucleotide pyrophosphatase/phosphodiesterase family member 3 | 111160296 | 100 | 2 | 4.10% | 2 |
| 1037 | cytoskeleton-associated protein 4 | 19920317 | 66 | 2 | 4.20% | 2 |
| 1038 | 60S ribosomal protein L5 | 14591909 | 34 | 2 | 11% | 2 |
| 1039 | membrane cofactor protein isoform 4 precursor | 24432108 | 43 | 2 | 7.30% | 2 |
| 1040 | PITH domain-containing protein 1 | 21361837 | 24 | 2 | 13% | 2 |
| 1041 | UMP-CMP kinase isoform a | 7706497 | 26 | 2 | 9.20% | 2 |
| 1042 | NADPH--cytochrome P450 reductase | 127139033 | 77 | 2 | 4.90% | 2 |
| 1043 | arylsulfatase A isoform a precursor . | 313569791 | 54 | 2 | 6.10% | 2 |
| 1044 | dehydrogenase/reductase SDR family member 7 precursor | 7706318 | 38 | 2 | 9.70% | 2 |
| 1045 | disintegrin and metalloproteinase domain-containing protein 29 preproprotein | 73765552 | 93 | 2 | 2.90% | 2 |
| 1046 | aminoacyl tRNA synthase complex-interacting multifunctional protein 2 | 11125770 | 35 | 2 | 11% | 2 |
| 1047 | growth arrest-specific protein 8 | 4503917 | 56 | 2 | 3.80% | 2 |
| 1048 | uncharacterized protein LOC100130705 | 304376316 | 20 | 2 | 12% | 2 |
| 1049 | 40S ribosomal protein S13 | 4506685 | 17 | 2 | 19% | 2 |
| 1050 | lon protease homolog, mitochondrial | 21396489 | 106 | 2 | 2.60% | 2 |
| 1051 | UBX domain-containing protein 6 isoform 1 | 13376854 | 50 | 2 | 11% | 2 |
| 1052 | translocation protein SEC63 homolog . | 6005872 | 88 | 2 | 2.60% | 2 |
| 1053 | beta-mannosidase precursor . | 84798622 | 101 | 2 | 2.50% | 2 |
| 1054 | ubiquitin-like-conjugating enzyme ATG3 | 19526773 | 36 | 2 | 8.90% | 2 |
| 1055 | transmembrane protein 209 | 66348165 | 63 | 2 | 7.70% | 2 |
