# Supplementary material for: Major protein alterations in spermatozoa from infertile men with unilateral varicocele
Source: Reprod Biol Endocrinol. 2015 Feb 22;13:8. doi: 10.1186/s12958-015-0007-2 (PMC4383193; doi:10.1186/s12958-015-0007-2)
Supplement: Additional file 3: Table S3. — Global proteomic profiling of fertile control group in triplicate - third run - gel 3. [file 12958_2015_7_MOESM3_ESM.docx]

**Supplemental Table 3. Global proteomic profiling of fertile control group in triplicate - third run – gel 3.**

| **No.** | **Gel 3** | | | | | |
| --- | --- | --- | --- | --- | --- | --- |
|  | **Protein** | **Accession No.** | **Mass** | **Peptides** | **Sequence** | **Spectral** |
|  |  |  | **kDa** | **No.** | **Cov (%)** | **Counts** |
| 1 | semenogelin-2 precursor | 4506885 | 65 | 51 | 73% | 2028 |
| 2 | semenogelin-1 preproprotein | 4506883 | 52 | 63 | 76% | 1488 |
| 3 | A-kinase anchor protein 4 isoform 2 | 21493039 | 93 | 74 | 74% | 1149 |
| 4 | lactotransferrin isoform 1 precursor | 54607120 | 78 | 77 | 80% | 1128 |
| 5 | tubulin beta-4B chain | 5174735 | 50 | 37 | 74% | 922 |
| 6 | heat shock-related 70 protein 2 | 13676857 | 70 | 51 | 69% | 805 |
| 7 | actin, cytoplasmic 1 | 4501885 | 42 | 37 | 91% | 788 |
| 8 | fibronectin isoform 3 preproprotein | 16933542 | 259 | 107 | 54% | 750 |
| 9 | tubulin alpha-3C/D chain | 17921993 | 50 | 38 | 76% | 696 |
| 10 | clusterin preproprotein | 355594753 | 52 | 32 | 53% | 614 |
| 11 | 78 glucose-regulated protein precursor | 16507237 | 72 | 37 | 57% | 572 |
| 12 | heat shock protein HSP 90-alpha isoform 1 | 153792590 | 98 | 48 | 47% | 525 |
| 13 | A-kinase anchor protein 3 | 21493041 | 95 | 49 | 50% | 514 |
| 14 | heat shock cognate 71 protein isoform 1 | 5729877 | 71 | 17 | 47% | 452 |
| 15 | ATP synthase subunit beta, mitochondrial precursor | 32189394 | 57 | 32 | 76% | 431 |
| 16 | endoplasmin precursor | 4507677 | 92 | 53 | 55% | 420 |
| 17 | keratin, type II cytoskeletal 1 | 119395750 | 66 | 35 | 52% | 409 |
| 18 | tubulin alpha-1A chain | 17986283 | 50 | 3 | 60% | 396 |
| 19 | prostatic acid phosphatase isoform TM-PAP precursor | 197116348 | 48 | 21 | 44% | 394 |
| 20 | heat shock 70 protein 1A/1B | 194248072 | 70 | 20 | 52% | 392 |
| 21 | pyruvate kinase isozymes M1/M2 isoform c | 332164775 | 66 | 42 | 65% | 390 |
| 22 | heat shock protein HSP 90-beta | 20149594 | 83 | 20 | 40% | 378 |
| 23 | heat shock 70 protein 1-like | 124256496 | 70 | 27 | 53% | 371 |
| 24 | protein disulfide-isomerase A3 precursor | 21361657 | 57 | 39 | 68% | 366 |
| 25 | alpha-enolase isoform 1 | 4503571 | 47 | 35 | 78% | 360 |
| 26 | prolactin-inducible protein precursor | 4505821 | 17 | 19 | 77% | 354 |
| 27 | keratin, type I cytoskeletal 9 | 55956899 | 62 | 33 | 54% | 297 |
| 28 | keratin, type I cytoskeletal 10 | 195972866 | 59 | 29 | 41% | 294 |
| 29 | prostate-specific antigen isoform 1 preproprotein | 4502173 | 29 | 15 | 69% | 285 |
| 30 | fructose-bisphosphate aldolase A isoform 2 | 342187211 | 45 | 35 | 88% | 279 |
| 31 | glyceraldehyde-3-phosphate dehydrogenase, testis-specific | 7657116 | 45 | 20 | 77% | 271 |
| 32 | triosephosphate isomerase isoform 2 | 226529917 | 31 | 28 | 94% | 247 |
| 33 | pyruvate kinase isozymes M1/M2 isoform a | 33286418 | 58 | 2 | 69% | 245 |
| 34 | tubulin beta chain | 29788785 | 50 | 2 | 62% | 243 |
| 35 | keratin, type II cytoskeletal 2 epidermal | 47132620 | 65 | 28 | 47% | 242 |
| 36 | outer dense fiber protein 2 isoform 3 | 310750406 | 81 | 28 | 37% | 242 |
| 37 | calcium-binding tyrosine phosphorylation-regulated protein isoform c | 24797112 | 41 | 21 | 61% | 239 |
| 38 | calcium-binding tyrosine phosphorylation-regulated protein isoform a | 24797108 | 53 | 7 | 46% | 235 |
| 39 | hypoxia up-regulated protein 1 precursor | 5453832 | 111 | 38 | 36% | 230 |
| 40 | keratin, type I cytoskeletal 13 isoform a | 131412225 | 50 | 25 | 56% | 225 |
| 41 | elongation factor 1-alpha 1 | 4503471 | 50 | 21 | 53% | 219 |
| 42 | tubulin alpha-1C chain | 14389309 | 50 | 2 | 57% | 214 |
| 43 | ropporin-1A | 21359920 | 24 | 17 | 81% | 206 |
| 44 | leucine-rich repeat-containing protein 37B precursor | 53829385 | 106 | 26 | 30% | 206 |
| 45 | acetyl-CoA acetyltransferase, mitochondrial precursor | 4557237 | 45 | 26 | 61% | 201 |
| 46 | ruvB-like 2 | 5730023 | 51 | 30 | 70% | 199 |
| 47 | malate dehydrogenase, mitochondrial precursor | 21735621 | 36 | 19 | 68% | 199 |
| 48 | 60 heat shock protein, mitochondrial | 31542947 | 61 | 37 | 79% | 192 |
| 49 | ATP synthase subunit alpha, mitochondrial precursor | 4757810 | 60 | 28 | 52% | 188 |
| 50 | keratin, type II cytoskeletal 6A | 5031839 | 60 | 14 | 41% | 181 |
| 51 | aminopeptidase N precursor | 157266300 | 110 | 42 | 43% | 181 |
| 52 | plastin-2 | 167614506 | 70 | 32 | 63% | 179 |
| 53 | fumarate hydratase, mitochondrial | 19743875 | 55 | 25 | 63% | 172 |
| 54 | ruvB-like 1 | 4506753 | 50 | 27 | 79% | 167 |
| 55 | glutathione S-transferase Mu 3 | 23065552 | 27 | 19 | 75% | 166 |
| 56 | T-complex protein 1 subunit beta isoform 1 | 5453603 | 57 | 28 | 62% | 163 |
| 57 | uncharacterized protein C1orf56 precursor | 20149646 | 37 | 16 | 49% | 162 |
| 58 | annexin A1 | 4502101 | 39 | 22 | 65% | 161 |
| 59 | heat shock protein beta-1 | 4504517 | 23 | 18 | 87% | 161 |
| 60 | cathelicidin antimicrobial peptide preproprotein | 348041314 | 20 | 13 | 51% | 160 |
| 61 | annexin A5 | 4502107 | 36 | 25 | 76% | 159 |
| 62 | myosin-9 | 12667788 | 227 | 52 | 29% | 158 |
| 63 | angiotensin-converting enzyme isoform 1 precursor | 4503273 | 150 | 27 | 23% | 158 |
| 64 | annexin A2 isoform 1 | 50845388 | 40 | 23 | 50% | 152 |
| 65 | acrosin-binding protein precursor | 17999524 | 61 | 24 | 46% | 150 |
| 66 | fatty acid synthase | 41872631 | 273 | 40 | 12% | 148 |
| 67 | nuclear pore membrane glycoprotein 210-like isoform 1 precursor | 117414168 | 211 | 50 | 32% | 145 |
| 68 | phosphoglycerate kinase 2 | 31543397 | 45 | 23 | 70% | 144 |
| 69 | leucine-rich repeat-containing protein 37A precursor | 289547512 | 188 | 19 | 12% | 144 |
| 70 | zona pellucida-binding protein 1 isoform 1 precursor | 229577313 | 40 | 18 | 52% | 139 |
| 71 | tubulin beta-4A chain | 21361322 | 50 | 2 | 61% | 139 |
| 72 | dipeptidyl peptidase 4 | 18765694 | 88 | 32 | 43% | 135 |
| 73 | transitional endoplasmic reticulum ATPase | 6005942 | 89 | 32 | 43% | 134 |
| 74 | ropporin-1B | 59891409 | 24 | 5 | 80% | 134 |
| 75 | keratin, type II cytoskeletal 4 | 331999954 | 56 | 25 | 54% | 131 |
| 76 | ras-related protein Rab-2A isoform a | 4506365 | 24 | 15 | 70% | 130 |
| 77 | cytochrome b-c1 complex subunit 2, mitochondrial precursor | 50592988 | 48 | 19 | 50% | 129 |
| 78 | hexokinase-1 isoform HKI-ta/tb | 15991831 | 103 | 33 | 36% | 128 |
| 79 | aconitate hydratase, mitochondrial precursor | 4501867 | 85 | 25 | 35% | 128 |
| 80 | glyceraldehyde-3-phosphate dehydrogenase | 7669492 | 36 | 18 | 59% | 127 |
| 81 | creatine kinase B-type | 21536286 | 43 | 16 | 49% | 126 |
| 82 | phospholipid hydroperoxide glutathione peroxidase, mitochondrial isoform A precursor | 75709200 | 22 | 16 | 63% | 123 |
| 83 | serum albumin preproprotein | 4502027 | 69 | 27 | 47% | 122 |
| 84 | sperm acrosome membrane-associated protein 1 precursor | 13569934 | 32 | 9 | 39% | 120 |
| 85 | izumo sperm-egg fusion protein 4 isoform 1 precursor | 89903025 | 24 | 9 | 50% | 120 |
| 86 | keratin, type II cytoskeletal 5 | 119395754 | 62 | 10 | 18% | 119 |
| 87 | sorbitol dehydrogenase | 156627571 | 38 | 15 | 41% | 119 |
| 88 | trifunctional enzyme subunit alpha, mitochondrial precursor | 20127408 | 83 | 24 | 38% | 115 |
| 89 | saccharopine dehydrogenase-like oxidoreductase | 55770836 | 47 | 16 | 51% | 114 |
| 90 | T-complex protein 1 subunit theta | 48762932 | 60 | 26 | 42% | 111 |
| 91 | tektin-3 | 13994250 | 57 | 20 | 52% | 110 |
| 92 | tubulin beta-6 chain | 14210536 | 50 | 4 | 20% | 109 |
| 93 | 14-3-3 protein epsilon | 5803225 | 29 | 20 | 73% | 109 |
| 94 | peroxiredoxin-1 | 320461711 | 22 | 13 | 58% | 109 |
| 95 | calreticulin precursor | 4757900 | 48 | 19 | 61% | 108 |
| 96 | pyruvate dehydrogenase E1 component subunit beta, mitochondrial isoform 1 precursor | 156564403 | 39 | 15 | 45% | 108 |
| 97 | sperm protein associated with the nucleus on the X chromosome C | 13435137 | 11 | 8 | 89% | 108 |
| 98 | sperm protein associated with the nucleus on the X chromosome B/F | 22027492 | 12 | 6 | 79% | 107 |
| 99 | cysteine-rich secretory protein 1 isoform 1 precursor | 327315372 | 28 | 11 | 55% | 106 |
| 100 | trifunctional enzyme subunit beta, mitochondrial precursor | 4504327 | 51 | 23 | 57% | 104 |
| 101 | beta-actin-like protein 2 | 63055057 | 42 | 2 | 35% | 104 |
| 102 | T-complex protein 1 subunit delta | 38455427 | 58 | 19 | 41% | 102 |
| 103 | cytochrome c oxidase subunit 4 isoform 1, mitochondrial precursor | 4502981 | 20 | 15 | 55% | 101 |
| 104 | 14-3-3 protein zeta/delta | 4507953 | 28 | 11 | 62% | 100 |
| 105 | protein disulfide-isomerase precursor | 20070125 | 57 | 24 | 51% | 100 |
| 106 | elongation factor 1-gamma | 4503481 | 50 | 16 | 52% | 99 |
| 107 | L-lactate dehydrogenase C chain | 4504973 | 36 | 17 | 49% | 97 |
| 108 | dynein light chain 2, cytoplasmic | 18087855 | 10 | 6 | 70% | 95 |
| 109 | T-complex protein 1 subunit gamma isoform a | 63162572 | 61 | 21 | 49% | 92 |
| 110 | cAMP-dependent protein kinase type I-alpha regulatory subunit | 4506063 | 43 | 13 | 40% | 92 |
| 111 | succinate dehydrogenase [ubiquinone] flavoprotein subunit, mitochondrial | 156416003 | 73 | 23 | 44% | 91 |
| 112 | lipoprotein lipase precursor | 4557727 | 53 | 15 | 36% | 91 |
| 113 | dynein light chain 1, cytoplasmic | 4505813 | 10 | 8 | 65% | 90 |
| 114 | ubiquitin-40S ribosomal protein S27a precursor | 208022622 | 18 | 4 | 30% | 90 |
| 115 | long-chain-fatty-acid--CoA ligase 1 | 40807491 | 78 | 22 | 27% | 89 |
| 116 | T-complex protein 1 subunit epsilon | 24307939 | 60 | 22 | 56% | 88 |
| 117 | citrate synthase, mitochondrial precursor | 38327625 | 52 | 18 | 58% | 85 |
| 118 | peroxiredoxin-6 | 4758638 | 25 | 17 | 69% | 83 |
| 119 | clathrin heavy chain 1 | 4758012 | 192 | 28 | 17% | 82 |
| 120 | T-complex protein 1 subunit eta isoform a | 5453607 | 59 | 19 | 50% | 82 |
| 121 | dolichyl-diphosphooligosaccharide--protein glycosyltransferase subunit 1 precursor | 4506675 | 69 | 23 | 51% | 81 |
| 122 | serum amyloid P-component precursor | 4502133 | 25 | 9 | 34% | 81 |
| 123 | cAMP-dependent protein kinase type II-alpha regulatory subunit | 4758958 | 46 | 18 | 42% | 80 |
| 124 | tektin-2 | 16507950 | 50 | 19 | 54% | 79 |
| 125 | erlin-2 isoform 1 | 6005721 | 38 | 14 | 52% | 79 |
| 126 | valyl-tRNA synthetase | 5454158 | 140 | 24 | 22% | 78 |
| 127 | protein FAM166A | 48717426 | 36 | 14 | 63% | 77 |
| 128 | 2,4-dienoyl-CoA reductase, mitochondrial precursor | 4503301 | 36 | 12 | 50% | 77 |
| 129 | isocitrate dehydrogenase [NADP] cytoplasmic | 28178825 | 47 | 15 | 42% | 77 |
| 130 | peroxiredoxin-5, mitochondrial isoform a precursor | 6912238 | 22 | 13 | 56% | 77 |
| 131 | neutral alpha-glucosidase AB isoform 2 precursor | 38202257 | 107 | 19 | 18% | 76 |
| 132 | cytosol aminopeptidase | 41393561 | 56 | 22 | 55% | 76 |
| 133 | dihydrolipoyllysine-residue acetyltransferase component of pyruvate dehydrogenase complex, mitochondrial precursor | 31711992 | 69 | 13 | 28% | 76 |
| 134 | cytosolic non-specific dipeptidase isoform 1 | 271398239 | 53 | 18 | 41% | 76 |
| 135 | radial spoke head protein 9 homolog isoform 1 | 32964825 | 31 | 22 | 89% | 76 |
| 136 | T-complex protein 1 subunit alpha isoform a | 57863257 | 60 | 21 | 59% | 75 |
| 137 | elongation factor 2 | 4503483 | 95 | 25 | 20% | 73 |
| 138 | laminin subunit beta-2 precursor | 119703755 | 196 | 24 | 17% | 73 |
| 139 | protein disulfide-isomerase A4 precursor | 4758304 | 73 | 17 | 30% | 73 |
| 140 | sperm protein associated with the nucleus on the X chromosome E | 22027496 | 11 | 2 | 89% | 73 |
| 141 | tubulin beta-3 chain isoform 1 | 50592996 | 50 | 2 | 28% | 72 |
| 142 | sperm equatorial segment protein 1 precursor | 21717832 | 39 | 11 | 29% | 72 |
| 143 | NADH-ubiquinone oxidoreductase 75 subunit, mitochondrial isoform 1 | 33519475 | 79 | 13 | 21% | 72 |
| 144 | radial spoke head protein 6 homolog A | 13540559 | 81 | 13 | 27% | 68 |
| 145 | adipocyte plasma membrane-associated protein | 24308201 | 46 | 18 | 51% | 68 |
| 146 | L-asparaginase | 145275200 | 32 | 13 | 56% | 68 |
| 147 | gastricsin isoform 1 preproprotein | 4505757 | 42 | 4 | 9.30% | 67 |
| 148 | tektin-4 | 21389613 | 51 | 17 | 46% | 67 |
| 149 | mitochondrial inner membrane protein isoform 3 | 154354966 | 83 | 21 | 33% | 66 |
| 150 | calnexin precursor | 10716563 | 68 | 17 | 28% | 66 |
| 151 | vesicular integral-membrane protein VIP36 precursor | 5803023 | 40 | 11 | 47% | 65 |
| 152 | 14-3-3 protein sigma | 5454052 | 28 | 11 | 58% | 65 |
| 153 | peroxiredoxin-2 isoform a | 32189392 | 22 | 11 | 55% | 65 |
| 154 | adenylate kinase isoenzyme 1 | 4502011 | 22 | 11 | 55% | 65 |
| 155 | 6-phosphofructokinase type C isoform 1 | 11321601 | 86 | 22 | 31% | 64 |
| 156 | peptidyl-prolyl cis-trans isomerase A | 10863927 | 18 | 12 | 67% | 64 |
| 157 | cytochrome c oxidase subunit II | 251831110 | 26 | 8 | 48% | 64 |
| 158 | laminin subunit alpha-5 precursor | 21264602 | 400 | 27 | 9.70% | 64 |
| 159 | radial spoke head 1 homolog | 18254456 | 35 | 12 | 52% | 64 |
| 160 | annexin A3 | 4826643 | 36 | 16 | 27% | 64 |
| 161 | dihydrolipoyl dehydrogenase, mitochondrial precursor | 91199540 | 54 | 15 | 41% | 63 |
| 162 | agrin precursor | 54873613 | 215 | 25 | 19% | 63 |
| 163 | ezrin | 21614499 | 69 | 15 | 21% | 62 |
| 164 | plasma serine protease inhibitor precursor | 194018472 | 46 | 15 | 48% | 61 |
| 165 | carnitine O-palmitoyltransferase 2, mitochondrial precursor | 4503023 | 74 | 18 | 39% | 60 |
| 166 | 14-3-3 protein theta | 5803227 | 28 | 7 | 47% | 60 |
| 167 | sperm surface protein Sp17 | 8394343 | 17 | 11 | 74% | 59 |
| 168 | alpha-centractin | 5031569 | 43 | 13 | 60% | 59 |
| 169 | D-3-phosphoglycerate dehydrogenase | 23308577 | 57 | 9 | 18% | 59 |
| 170 | keratin, type I cytoskeletal 14 | 15431310 | 52 | 7 | 28% | 58 |
| 171 | histone H2B type 1-A | 24586679 | 14 | 6 | 41% | 58 |
| 172 | protein disulfide-isomerase A6 precursor | 5031973 | 48 | 13 | 32% | 58 |
| 173 | acrosomal protein SP-10 isoform a precursor | 4501879 | 28 | 7 | 24% | 56 |
| 174 | dipeptidase 3 isoform a precursor | 193211608 | 56 | 10 | 21% | 56 |
| 175 | elongation factor 1-delta isoform 1 | 304555581 | 71 | 8 | 15% | 55 |
| 176 | voltage-dependent anion-selective channel protein 2 isoform 2 | 296317339 | 32 | 12 | 52% | 54 |
| 177 | erlin-1 | 154800487 | 39 | 7 | 35% | 54 |
| 178 | myosin light polypeptide 6 isoform 2 | 88999583 | 17 | 8 | 53% | 54 |
| 179 | cytochrome c oxidase subunit 5B, mitochondrial precursor | 17017988 | 14 | 12 | 43% | 54 |
| 180 | T-complex protein 1 subunit zeta-2 isoform 1 | 58331173 | 58 | 8 | 30% | 52 |
| 181 | heat shock 70 protein 4L | 31541941 | 95 | 17 | 26% | 52 |
| 182 | epididymal sperm-binding protein 1 precursor | 301601648 | 26 | 8 | 44% | 52 |
| 183 | ras-related protein Rab-11B | 190358517 | 24 | 11 | 52% | 52 |
| 184 | histone H4 | 4504303 | 11 | 9 | 59% | 51 |
| 185 | voltage-dependent anion-selective channel protein 3 isoform 1 | 25188179 | 31 | 13 | 48% | 51 |
| 186 | stress-70 protein, mitochondrial precursor | 24234688 | 74 | 13 | 26% | 50 |
| 187 | alpha-actinin-4 | 12025678 | 105 | 16 | 23% | 50 |
| 188 | protein phosphatase 1 regulatory subunit 7 | 4506013 | 42 | 13 | 47% | 50 |
| 189 | electron transfer flavoprotein subunit alpha, mitochondrial isoform a | 4503607 | 35 | 15 | 66% | 50 |
| 190 | hydroxyacyl-coenzyme A dehydrogenase, mitochondrial isoform 1 precursor | 296179427 | 36 | 10 | 54% | 50 |
| 191 | cytochrome c oxidase subunit 5A, mitochondrial precursor | 190885499 | 17 | 7 | 38% | 48 |
| 192 | succinyl-CoA:3-ketoacid-coenzyme A transferase 1, mitochondrial precursor | 4557817 | 56 | 14 | 41% | 48 |
| 193 | peroxiredoxin-4 precursor | 5453549 | 31 | 12 | 49% | 48 |
| 194 | acrosin precursor | 148613878 | 46 | 14 | 39% | 48 |
| 195 | glutathione S-transferase P | 4504183 | 23 | 10 | 60% | 48 |
| 196 | enoyl-CoA hydratase, mitochondrial | 194097323 | 31 | 13 | 53% | 48 |
| 197 | beta-2-microglobulin precursor | 4757826 | 14 | 5 | 38% | 47 |
| 198 | ATP synthase subunit b, mitochondrial precursor | 21361565 | 29 | 12 | 52% | 47 |
| 199 | hyaluronidase PH-20 isoform 2 | 291290981 | 58 | 8 | 23% | 46 |
| 200 | cytochrome b-c1 complex subunit 1, mitochondrial precursor | 46593007 | 53 | 17 | 46% | 46 |
| 201 | ATP synthase subunit d, mitochondrial isoform a | 5453559 | 18 | 12 | 80% | 46 |
| 202 | olfactomedin-4 precursor | 32313593 | 57 | 10 | 28% | 46 |
| 203 | uncharacterized protein KIAA1683 isoform a | 224451032 | 147 | 15 | 14% | 46 |
| 204 | keratin, type II cytoskeletal 6B | 119703753 | 60 | 2 | 22% | 46 |
| 205 | ferritin, mitochondrial precursor | 29126241 | 28 | 9 | 47% | 46 |
| 206 | uncharacterized protein C9orf9 | 33285006 | 19 | 10 | 79% | 46 |
| 207 | 26S protease regulatory subunit 4 | 24430151 | 49 | 10 | 20% | 45 |
| 208 | 3-hydroxyisobutyrate dehydrogenase, mitochondrial precursor | 23308751 | 35 | 8 | 30% | 45 |
| 209 | peptidyl-prolyl cis-trans isomerase B precursor | 4758950 | 24 | 10 | 55% | 44 |
| 210 | nucleobindin-2 precursor | 4826870 | 50 | 13 | 38% | 44 |
| 211 | protein FAM71B | 222418633 | 65 | 10 | 11% | 44 |
| 212 | uncharacterized protein C20orf106 precursor | 71043622 | 20 | 8 | 60% | 44 |
| 213 | synaptophysin-like protein 1 isoform a | 5803185 | 29 | 5 | 30% | 44 |
| 214 | plastin-3 isoform 1 | 209862851 | 71 | 11 | 27% | 43 |
| 215 | importin subunit beta-1 | 19923142 | 97 | 13 | 12% | 43 |
| 216 | 60S acidic ribosomal protein P0 | 16933546 | 34 | 9 | 46% | 43 |
| 217 | nucleoside diphosphate kinase homolog 5 | 4505413 | 24 | 8 | 52% | 43 |
| 218 | annexin A6 isoform 1 | 71773329 | 76 | 15 | 25% | 42 |
| 219 | protein-glutamine gamma-glutamyltransferase 4 | 156627577 | 77 | 15 | 20% | 42 |
| 220 | myeloid leukemia factor 1 isoform 1 | 11967975 | 31 | 11 | 40% | 42 |
| 221 | T-complex protein 1 subunit zeta isoform a | 4502643 | 58 | 12 | 26% | 41 |
| 222 | phosphoglycerate kinase 1 | 4505763 | 45 | 7 | 31% | 41 |
| 223 | dolichyl-diphosphooligosaccharide--protein glycosyltransferase subunit 2 isoform 2 precursor | 209413738 | 68 | 15 | 43% | 41 |
| 224 | vesicle-associated membrane protein-associated protein A isoform 2 | 94721252 | 28 | 9 | 49% | 41 |
| 225 | fructose-bisphosphate aldolase C | 4885063 | 39 | 2 | 21% | 41 |
| 226 | 26S protease regulatory subunit 6A | 21361144 | 49 | 11 | 36% | 41 |
| 227 | isocitrate dehydrogenase [NAD] subunit alpha, mitochondrial precursor | 5031777 | 40 | 9 | 31% | 41 |
| 228 | axonemal dynein light intermediate polypeptide 1 | 37595560 | 32 | 13 | 52% | 41 |
| 229 | 26S protease regulatory subunit 8 isoform 1 | 24497435 | 46 | 9 | 27% | 41 |
| 230 | hsc70-interacting protein | 19923193 | 41 | 6 | 18% | 41 |
| 231 | carbonic anhydrase 4 precursor | 4502519 | 35 | 10 | 34% | 41 |
| 232 | protein DJ-1 | 31543380 | 20 | 12 | 82% | 41 |
| 233 | keratin, type I cytoskeletal 16 | 24430192 | 51 | 5 | 30% | 40 |
| 234 | glutamine synthetase | 19923206 | 42 | 7 | 22% | 40 |
| 235 | L-lactate dehydrogenase A chain isoform 3 | 260099723 | 40 | 10 | 37% | 40 |
| 236 | matrix-remodeling-associated protein 5 precursor | 139948432 | 312 | 14 | 5.80% | 39 |
| 237 | profilin-1 | 4826898 | 15 | 9 | 65% | 39 |
| 238 | delta(3,5)-Delta(2,4)-dienoyl-CoA isomerase, mitochondrial precursor | 70995211 | 36 | 13 | 57% | 39 |
| 239 | calmodulin | 58218968 | 17 | 11 | 81% | 38 |
| 240 | mucin-6 precursor | 151301154 | 257 | 14 | 6.70% | 38 |
| 241 | mitochondrial carrier homolog 2 | 7657347 | 33 | 9 | 46% | 38 |
| 242 | dolichyl-diphosphooligosaccharide--protein glycosyltransferase 48 subunit precursor | 20070197 | 51 | 10 | 32% | 38 |
| 243 | ras-related protein Rab-2B isoform 1 | 21361884 | 24 | 3 | 53% | 38 |
| 244 | coiled-coil domain-containing protein 147 | 56961680 | 103 | 15 | 19% | 38 |
| 245 | apolipoprotein A-I preproprotein | 4557321 | 31 | 10 | 39% | 38 |
| 246 | ATP synthase subunit O, mitochondrial precursor | 4502303 | 23 | 12 | 67% | 37 |
| 247 | nuclear pore complex protein Nup155 isoform 1 | 24430149 | 155 | 15 | 15% | 37 |
| 248 | enkurin | 21450721 | 29 | 11 | 47% | 37 |
| 249 | NADH dehydrogenase [ubiquinone] iron-sulfur protein 3, mitochondrial precursor | 4758788 | 30 | 12 | 43% | 37 |
| 250 | dihydrolipoyllysine-residue succinyltransferase component of 2-oxoglutarate dehydrogenase complex, mitochondrial isoform 1 precursor | 19923748 | 49 | 10 | 34% | 36 |
| 251 | ADP/ATP translocase 4 | 13775208 | 35 | 11 | 37% | 36 |
| 252 | mitochondria-eating protein | 21687119 | 61 | 9 | 18% | 36 |
| 253 | laminin subunit gamma-1 precursor | 145309326 | 178 | 14 | 12% | 35 |
| 254 | sperm acrosome membrane-associated protein 3 | 27777653 | 23 | 6 | 28% | 35 |
| 255 | proteasome subunit alpha type-1 isoform 1 | 23110935 | 30 | 12 | 56% | 35 |
| 256 | ras-related protein Rab-14 | 19923483 | 24 | 11 | 64% | 35 |
| 257 | actin-related protein T2 | 29893808 | 42 | 10 | 39% | 34 |
| 258 | neprilysin | 116256329 | 86 | 15 | 20% | 34 |
| 259 | outer dense fiber protein 3 | 19526475 | 28 | 9 | 43% | 34 |
| 260 | prohibitin | 4505773 | 30 | 9 | 42% | 34 |
| 261 | 26S protease regulatory subunit 6B isoform 1 | 5729991 | 47 | 12 | 35% | 34 |
| 262 | isochorismatase domain-containing protein 2, mitochondrial isoform 2 | 13376007 | 24 | 6 | 60% | 34 |
| 263 | endoplasmic reticulum resident protein 29 isoform 1 precursor | 5803013 | 29 | 10 | 44% | 34 |
| 264 | alpha-crystallin B chain | 4503057 | 20 | 11 | 62% | 34 |
| 265 | cofilin-1 | 5031635 | 19 | 10 | 56% | 33 |
| 266 | glucose-6-phosphate isomerase isoform 2 | 18201905 | 63 | 11 | 28% | 33 |
| 267 | transmembrane emp24 domain-containing protein 10 precursor | 98986464 | 25 | 9 | 42% | 33 |
| 268 | reticulocalbin-2 precursor | 4506457 | 37 | 12 | 46% | 33 |
| 269 | histone H3.1 | 4504281 | 15 | 4 | 20% | 33 |
| 270 | nucleoporin p54 | 26051237 | 55 | 10 | 24% | 33 |
| 271 | proteasome subunit alpha type-6 | 23110944 | 27 | 9 | 39% | 33 |
| 272 | protein dpy-19 homolog 2 | 93277105 | 87 | 10 | 15% | 32 |
| 273 | protein-L-isoaspartate(D-aspartate) O-methyltransferase isoform 2 | 354983493 | 30 | 11 | 52% | 32 |
| 274 | ubiquitin-like modifier-activating enzyme 1 | 23510340 | 118 | 10 | 8.60% | 32 |
| 275 | 14-3-3 protein beta/alpha | 21328448 | 28 | 3 | 35% | 32 |
| 276 | adenylate kinase 7 | 148727333 | 83 | 9 | 18% | 32 |
| 277 | short-chain specific acyl-CoA dehydrogenase, mitochondrial precursor | 4557233 | 44 | 7 | 25% | 32 |
| 278 | thioredoxin-related transmembrane protein 4 precursor | 40254947 | 39 | 6 | 17% | 32 |
| 279 | importin subunit alpha-2 | 4504897 | 58 | 9 | 23% | 31 |
| 280 | nuclear pore complex protein Nup93 isoform 1 | 208609990 | 93 | 11 | 12% | 31 |
| 281 | medium-chain specific acyl-CoA dehydrogenase, mitochondrial isoform b precursor | 187960098 | 47 | 12 | 35% | 31 |
| 282 | heme oxygenase 2 | 8051608 | 36 | 9 | 46% | 31 |
| 283 | transmembrane emp24 domain-containing protein 9 precursor | 39725636 | 27 | 8 | 24% | 31 |
| 284 | ADP-ribosylation factor 1 | 4502201 | 21 | 9 | 52% | 31 |
| 285 | epididymal secretory protein E3-beta precursor | 11641279 | 18 | 6 | 50% | 31 |
| 286 | stomatin-like protein 2 | 7305503 | 39 | 10 | 51% | 31 |
| 287 | cytochrome b-c1 complex subunit Rieske, mitochondrial | 163644321 | 30 | 8 | 39% | 31 |
| 288 | uncharacterized protein C20orf107 precursor | 71043642 | 19 | 10 | 56% | 30 |
| 289 | synaptic vesicle membrane protein VAT-1 homolog | 18379349 | 42 | 11 | 39% | 30 |
| 290 | brain acid soluble protein 1 | 30795231 | 23 | 6 | 47% | 30 |
| 291 | 26S proteasome non-ATPase regulatory subunit 13 isoform 1 | 157502193 | 43 | 10 | 27% | 29 |
| 292 | proteasome subunit alpha type-5 isoform 1 | 23110942 | 26 | 10 | 53% | 29 |
| 293 | annexin A4 | 4502105 | 36 | 11 | 41% | 29 |
| 294 | abhydrolase domain-containing protein 10, mitochondrial precursor | 8923001 | 34 | 6 | 26% | 29 |
| 295 | sodium/potassium-transporting ATPase subunit alpha-4 isoform 1 | 153946397 | 114 | 12 | 13% | 29 |
| 296 | protein NDRG1 | 37655183 | 43 | 5 | 22% | 29 |
| 297 | carbonyl reductase [NADPH] 1 | 4502599 | 30 | 10 | 40% | 29 |
| 298 | isocitrate dehydrogenase [NAD] subunit beta, mitochondrial isoform a precursor | 28178821 | 42 | 12 | 35% | 29 |
| 299 | rab GDP dissociation inhibitor beta isoform 1 | 6598323 | 51 | 7 | 24% | 29 |
| 300 | NADH dehydrogenase [ubiquinone] 1 beta subcomplex subunit 10 | 4758774 | 21 | 6 | 37% | 29 |
| 301 | DPY30 domain-containing protein 1 | 20270377 | 21 | 11 | 76% | 29 |
| 302 | serpin B6 | 41152086 | 43 | 10 | 39% | 28 |
| 303 | 26S proteasome non-ATPase regulatory subunit 7 | 25777615 | 37 | 7 | 31% | 28 |
| 304 | cytochrome c1, heme protein, mitochondrial | 21359867 | 35 | 10 | 52% | 28 |
| 305 | hydroxyacylglutathione hydrolase, mitochondrial isoform 1 precursor | 94538322 | 34 | 11 | 37% | 28 |
| 306 | SYNJ2BP-COX16 protein isoform 1 | 321400118 | 21 | 7 | 33% | 28 |
| 307 | proteasome subunit beta type-5 isoform 1 | 4506201 | 28 | 7 | 32% | 28 |
| 308 | glycerol kinase 2 | 41393575 | 61 | 7 | 17% | 28 |
| 309 | uromodulin precursor | 59850812 | 70 | 10 | 17% | 28 |
| 310 | solute carrier family 2, facilitated glucose transporter member 14 | 23592238 | 56 | 8 | 14% | 28 |
| 311 | signal peptidase complex subunit 2 | 162417971 | 25 | 11 | 39% | 28 |
| 312 | tetratricopeptide repeat protein 25 | 13899233 | 77 | 11 | 14% | 28 |
| 313 | ribonuclease inhibitor | 42822872 | 50 | 7 | 15% | 28 |
| 314 | proteasome subunit beta type-6 | 23110925 | 25 | 7 | 47% | 28 |
| 315 | single-stranded DNA-binding protein, mitochondrial precursor | 4507231 | 17 | 6 | 42% | 27 |
| 316 | electron transfer flavoprotein subunit beta isoform 1 | 4503609 | 28 | 11 | 45% | 27 |
| 317 | maltase-glucoamylase, intestinal | 221316699 | 210 | 10 | 7.10% | 27 |
| 318 | endophilin-B1 isoform 2 | 331284170 | 44 | 8 | 21% | 27 |
| 319 | 14-3-3 protein gamma | 21464101 | 28 | 3 | 34% | 27 |
| 320 | solute carrier family 2, facilitated glucose transporter member 3 | 5902090 | 54 | 3 | 14% | 27 |
| 321 | L-xylulose reductase isoform 1 | 7705925 | 26 | 10 | 36% | 27 |
| 322 | collagen alpha-1(XVIII) chain isoform 1 precursor | 110611235 | 154 | 10 | 7.70% | 27 |
| 323 | proteasome subunit beta type-7 proprotein | 4506203 | 30 | 9 | 49% | 27 |
| 324 | sperm-associated antigen 6 isoform 1 | 6912678 | 55 | 10 | 33% | 26 |
| 325 | succinate dehydrogenase [ubiquinone] iron-sulfur subunit, mitochondrial precursor | 115387094 | 32 | 8 | 33% | 26 |
| 326 | radial spoke head protein 3 homolog | 31543559 | 64 | 7 | 17% | 26 |
| 327 | transmembrane protease serine 2 isoform 2 | 205360943 | 54 | 5 | 8.70% | 26 |
| 328 | proteasome subunit alpha type-7-like isoform 2 | 68303563 | 28 | 9 | 50% | 26 |
| 329 | beta-defensin 129 precursor | 18250304 | 20 | 4 | 24% | 26 |
| 330 | receptor expression-enhancing protein 6 | 19923919 | 21 | 7 | 33% | 26 |
| 331 | ropporin-1-like protein | 17572807 | 26 | 10 | 47% | 26 |
| 332 | adenylyl cyclase-associated protein 1 | 5453595 | 52 | 9 | 21% | 26 |
| 333 | alpha-soluble NSF attachment protein | 47933379 | 33 | 9 | 42% | 26 |
| 334 | malectin precursor | 7661948 | 32 | 9 | 41% | 25 |
| 335 | uncharacterized protein C7orf61 | 51972226 | 24 | 5 | 34% | 25 |
| 336 | proteasome subunit alpha type-2 | 4506181 | 26 | 8 | 48% | 25 |
| 337 | phosphatidylethanolamine-binding protein 1 preproprotein | 4505621 | 21 | 6 | 53% | 25 |
| 338 | ubiquitin carboxyl-terminal hydrolase isozyme L3 | 5174741 | 26 | 6 | 33% | 25 |
| 339 | flavin reductase (NADPH) | 4502419 | 22 | 6 | 35% | 25 |
| 340 | eukaryotic translation initiation factor 5A-1 isoform B | 219555712 | 17 | 9 | 51% | 25 |
| 341 | ras-related protein Rab-27B | 5729997 | 25 | 9 | 35% | 25 |
| 342 | NADH dehydrogenase [ubiquinone] iron-sulfur protein 8, mitochondrial precursor | 4505371 | 24 | 5 | 24% | 25 |
| 343 | carboxypeptidase Z isoform 2 precursor | 62388875 | 73 | 7 | 9.50% | 24 |
| 344 | carboxypeptidase E preproprotein | 4503009 | 53 | 7 | 19% | 24 |
| 345 | nuclear pore membrane glycoprotein 210 precursor | 27477134 | 205 | 12 | 9.20% | 24 |
| 346 | 26S protease regulatory subunit 10B | 195539395 | 46 | 8 | 25% | 24 |
| 347 | 26S proteasome non-ATPase regulatory subunit 14 | 5031981 | 35 | 6 | 40% | 24 |
| 348 | plasma membrane calcium-transporting ATPase 4 isoform 4b | 48255957 | 134 | 10 | 10% | 24 |
| 349 | alanyl-tRNA editing protein Aarsd1 isoform 1 | 217416402 | 66 | 8 | 21% | 24 |
| 350 | coiled-coil-helix-coiled-coil-helix domain-containing protein 3, mitochondrial precursor | 8923390 | 26 | 7 | 24% | 24 |
| 351 | 26S proteasome non-ATPase regulatory subunit 11 | 28872725 | 47 | 7 | 21% | 24 |
| 352 | transmembrane protein 190 precursor | 21040263 | 19 | 5 | 31% | 24 |
| 353 | L-lactate dehydrogenase A-like 6B | 15082234 | 42 | 6 | 28% | 24 |
| 354 | proteasome subunit beta type-4 | 22538467 | 29 | 7 | 45% | 24 |
| 355 | membrane-associated progesterone receptor component 1 | 5729875 | 22 | 8 | 40% | 24 |
| 356 | proteasome subunit beta type-3 | 22538465 | 23 | 5 | 32% | 24 |
| 357 | very long-chain specific acyl-CoA dehydrogenase, mitochondrial isoform 2 precursor | 76496475 | 68 | 10 | 25% | 23 |
| 358 | filamin-B isoform 2 | 105990514 | 278 | 13 | 5.90% | 23 |
| 359 | lipid phosphate phosphohydrolase 1 isoform 1 | 29171736 | 32 | 4 | 21% | 23 |
| 360 | lysosome-associated membrane glycoprotein 1 precursor | 112380628 | 45 | 4 | 9.80% | 23 |
| 361 | glypican-4 precursor | 21614525 | 62 | 7 | 17% | 23 |
| 362 | membrane-associated progesterone receptor component 2 | 291621647 | 26 | 4 | 17% | 23 |
| 363 | endoplasmic reticulum resident protein 44 precursor | 52487191 | 47 | 6 | 25% | 23 |
| 364 | epididymal secretory protein E3-alpha precursor | 11386189 | 18 | 5 | 41% | 23 |
| 365 | proteasome subunit alpha type-7 | 4506189 | 28 | 3 | 42% | 23 |
| 366 | pyruvate dehydrogenase E1 component subunit alpha, testis-specific form, mitochondrial precursor | 4885543 | 43 | 8 | 18% | 22 |
| 367 | G-protein coupled receptor 64 isoform 2 precursor | 119943116 | 110 | 6 | 8.50% | 22 |
| 368 | vesicle-fusing ATPase | 156564401 | 83 | 8 | 13% | 22 |
| 369 | ES1 protein homolog, mitochondrial isoform Ia precursor | 296531406 | 28 | 7 | 38% | 22 |
| 370 | tektin-1 | 16753231 | 48 | 7 | 23% | 22 |
| 371 | glutathione reductase, mitochondrial isoform 1 precursor | 50301238 | 56 | 10 | 39% | 22 |
| 372 | lactadherin isoform a preproprotein | 167830475 | 43 | 7 | 24% | 22 |
| 373 | uncharacterized protein C9orf24 isoform 1 | 21362074 | 30 | 7 | 28% | 22 |
| 374 | enoyl-CoA delta isomerase 1, mitochondrial isoform 2 precursor | 295842266 | 31 | 5 | 20% | 22 |
| 375 | translin | 4759270 | 26 | 9 | 46% | 22 |
| 376 | ADP-ribosylation factor 4 | 4502205 | 21 | 3 | 44% | 22 |
| 377 | NADH dehydrogenase [ubiquinone] flavoprotein 2, mitochondrial precursor | 222080062 | 27 | 8 | 32% | 22 |
| 378 | protein S100-A8 | 21614544 | 11 | 5 | 47% | 22 |
| 379 | WD repeat-containing protein 16 isoform b | 124028512 | 68 | 9 | 21% | 21 |
| 380 | tripeptidyl-peptidase 2 | 186972143 | 138 | 9 | 10% | 21 |
| 381 | keratin, type I cytoskeletal 19 | 24234699 | 44 | 3 | 19% | 21 |
| 382 | aspartate aminotransferase, mitochondrial precursor | 73486658 | 48 | 9 | 32% | 21 |
| 383 | calcium-binding and spermatid-specific protein 1 | 90652863 | 43 | 7 | 32% | 21 |
| 384 | 26S proteasome non-ATPase regulatory subunit 2 | 25777602 | 100 | 11 | 15% | 21 |
| 385 | parkin coregulated gene protein isoform 2 | 122939202 | 29 | 6 | 32% | 21 |
| 386 | CDGSH iron-sulfur domain-containing protein 1 | 8923930 | 12 | 6 | 56% | 21 |
| 387 | transmembrane and coiled-coil domain-containing protein 2 | 56847610 | 20 | 5 | 28% | 21 |
| 388 | FUN14 domain-containing protein 2 | 24371248 | 21 | 10 | 42% | 21 |
| 389 | vesicle-associated membrane protein 3 | 4759300 | 11 | 6 | 53% | 21 |
| 390 | acrosome formation-associated factor isoform 1 | 239582757 | 33 | 4 | 21% | 21 |
| 391 | NME1-NME2 protein | 66392203 | 30 | 8 | 39% | 21 |
| 392 | ribonuclease-like protein 13 precursor | 59276062 | 18 | 6 | 35% | 21 |
| 393 | COP9 signalosome complex subunit 4 | 38373690 | 46 | 8 | 21% | 21 |
| 394 | zymogen granule protein 16 homolog B precursor | 94536866 | 23 | 5 | 34% | 21 |
| 395 | keratin, type II cytoskeletal 8 | 4504919 | 54 | 4 | 14% | 20 |
| 396 | 2-oxoglutarate dehydrogenase, mitochondrial isoform 3 precursor | 259013553 | 116 | 9 | 9.80% | 20 |
| 397 | alcohol dehydrogenase [NADP+] | 24497577 | 37 | 6 | 13% | 20 |
| 398 | eukaryotic initiation factor 4A-I isoform 1 | 4503529 | 46 | 6 | 17% | 20 |
| 399 | alpha-actinin-1 isoform c | 194097352 | 103 | 2 | 11% | 20 |
| 400 | cullin-associated NEDD8-dissociated protein 1 | 21361794 | 136 | 8 | 8.80% | 20 |
| 401 | adenosylhomocysteinase isoform 1 | 9951915 | 48 | 8 | 21% | 20 |
| 402 | proteasome subunit beta type-1 | 4506193 | 26 | 6 | 28% | 20 |
| 403 | 26S protease regulatory subunit 7 isoform 1 | 4506209 | 49 | 8 | 25% | 20 |
| 404 | protein FAM154A | 301129242 | 55 | 5 | 16% | 20 |
| 405 | sperm acrosome-associated protein 5 precursor | 120952755 | 18 | 5 | 35% | 20 |
| 406 | barrier-to-autointegration factor | 4502389 | 10 | 4 | 45% | 20 |
| 407 | glutamate carboxypeptidase 2 isoform 1 | 4758398 | 84 | 9 | 18% | 20 |
| 408 | GTP-binding nuclear protein Ran | 5453555 | 24 | 6 | 35% | 20 |
| 409 | glypican-1 precursor | 167001141 | 62 | 7 | 15% | 20 |
| 410 | sodium/potassium-transporting ATPase subunit alpha-2 proprotein | 4502271 | 112 | 2 | 9.10% | 20 |
| 411 | lysozyme-like protein 4 precursor | 21389465 | 16 | 5 | 42% | 20 |
| 412 | thioredoxin domain-containing protein 3 | 148839372 | 67 | 8 | 19% | 19 |
| 413 | importin-5 | 24797086 | 126 | 8 | 10% | 19 |
| 414 | glucosidase 2 subunit beta isoform 1 precursor | 48255889 | 59 | 7 | 13% | 19 |
| 415 | lysosomal alpha-glucosidase preproprotein | 119393891 | 105 | 6 | 8.00% | 19 |
| 416 | sodium/potassium-transporting ATPase subunit beta-3 | 4502281 | 32 | 5 | 33% | 19 |
| 417 | CD59 glycoprotein preproprotein | 187828910 | 14 | 3 | 25% | 19 |
| 418 | calmin | 19923599 | 112 | 10 | 15% | 19 |
| 419 | adenylate kinase 8 | 22749187 | 55 | 7 | 19% | 19 |
| 420 | extracellular matrix protein 1 isoform 3 precursor | 322302700 | 64 | 7 | 11% | 19 |
| 421 | transcription factor A, mitochondrial precursor | 4507401 | 29 | 7 | 32% | 19 |
| 422 | kallikrein-2 isoform 1 precursor | 5031829 | 29 | 5 | 18% | 19 |
| 423 | 40S ribosomal protein S2 | 15055539 | 31 | 7 | 21% | 19 |
| 424 | kelch-like protein 10 | 148664209 | 69 | 9 | 24% | 19 |
| 425 | 40S ribosomal protein S3 | 15718687 | 27 | 8 | 32% | 19 |
| 426 | receptor expression-enhancing protein 5 | 115430112 | 21 | 6 | 25% | 19 |
| 427 | sodium/potassium-transporting ATPase subunit alpha-1 isoform a | 21361181 | 113 | 2 | 10% | 19 |
| 428 | 4-trimethylaminobutyraldehyde dehydrogenase | 115387104 | 56 | 7 | 15% | 18 |
| 429 | NADH-cytochrome b5 reductase 2 | 47778923 | 31 | 9 | 46% | 18 |
| 430 | kunitz-type protease inhibitor 3 precursor | 189571689 | 10 | 3 | 29% | 18 |
| 431 | dynactin subunit 2 | 5453629 | 45 | 6 | 22% | 18 |
| 432 | calpain small subunit 1 | 51599151 | 28 | 5 | 44% | 18 |
| 433 | ATP synthase subunit gamma, mitochondrial isoform L (liver) precursor | 50345988 | 33 | 6 | 22% | 18 |
| 434 | protein S100-A11 | 5032057 | 12 | 6 | 52% | 18 |
| 435 | galectin-3-binding protein precursor | 5031863 | 65 | 6 | 9.90% | 18 |
| 436 | BPI fold-containing family A member 3 isoform 1 precursor | 109627654 | 28 | 5 | 31% | 18 |
| 437 | mitochondrial import receptor subunit TOM22 homolog | 9910382 | 16 | 6 | 77% | 18 |
| 438 | epididymal secretory protein E1 precursor | 5453678 | 17 | 6 | 45% | 18 |
| 439 | cathepsin F precursor | 6042196 | 53 | 7 | 12% | 18 |
| 440 | translationally-controlled tumor protein | 4507669 | 20 | 5 | 55% | 18 |
| 441 | EF-hand calcium-binding domain-containing protein 6 isoform a | 38570107 | 173 | 7 | 4.20% | 18 |
| 442 | transmembrane emp24 domain-containing protein 7 precursor | 32996709 | 25 | 7 | 39% | 18 |
| 443 | ras-related protein Rab-1B | 13569962 | 22 | 5 | 36% | 18 |
| 444 | casein kinase II subunit alpha isoform a | 29570791 | 45 | 5 | 22% | 18 |
| 445 | 40S ribosomal protein SA | 59859885 | 33 | 6 | 32% | 18 |
| 446 | deoxyuridine 5'-triphosphate nucleotidohydrolase, mitochondrial isoform 1 precursor | 70906441 | 27 | 6 | 33% | 18 |
| 447 | adenine phosphoribosyltransferase isoform a | 4502171 | 20 | 7 | 36% | 18 |
| 448 | chloride intracellular channel protein 1 | 14251209 | 27 | 7 | 30% | 17 |
| 449 | cytochrome b-c1 complex subunit 7 isoform 1 | 5454152 | 14 | 7 | 55% | 17 |
| 450 | rho GDP-dissociation inhibitor 1 isoform a | 4757768 | 23 | 3 | 23% | 17 |
| 451 | transmembrane emp24 domain-containing protein 4 precursor | 33457308 | 26 | 3 | 20% | 17 |
| 452 | lysyl-tRNA synthetase isoform 1 | 194272210 | 71 | 6 | 11% | 17 |
| 453 | dnaJ homolog subfamily B member 11 precursor | 7706495 | 41 | 7 | 25% | 17 |
| 454 | carboxypeptidase D isoform 1 precursor | 22202611 | 153 | 10 | 11% | 17 |
| 455 | phosphoglycerate mutase 2 | 50593010 | 29 | 5 | 27% | 17 |
| 456 | ras GTPase-activating-like protein IQGAP1 | 4506787 | 189 | 9 | 6.30% | 17 |
| 457 | 40S ribosomal protein S3a | 4506723 | 30 | 7 | 26% | 17 |
| 458 | eukaryotic translation initiation factor 3 subunit F | 4503519 | 38 | 5 | 16% | 17 |
| 459 | nicastrin precursor | 24638433 | 78 | 6 | 9.90% | 17 |
| 460 | signal peptidase complex subunit 3 | 11345462 | 20 | 5 | 26% | 17 |
| 461 | beta-lactamase-like protein 2 | 7705793 | 33 | 7 | 32% | 17 |
| 462 | stress-induced-phosphoprotein 1 | 5803181 | 63 | 6 | 13% | 17 |
| 463 | von Willebrand factor A domain-containing protein 1 isoform 1 precursor | 40068485 | 47 | 7 | 28% | 17 |
| 464 | prenylcysteine oxidase 1 precursor | 166795301 | 57 | 6 | 18% | 17 |
| 465 | ras-related protein Rab-3D | 4759000 | 24 | 3 | 32% | 17 |
| 466 | ATPase inhibitor, mitochondrial isoform 1 precursor | 7705927 | 12 | 3 | 15% | 17 |
| 467 | F-actin-capping protein subunit alpha-1 | 5453597 | 33 | 5 | 31% | 17 |
| 468 | lactoylglutathione lyase | 118402586 | 21 | 6 | 29% | 17 |
| 469 | proteasome subunit beta type-2 isoform 1 | 4506195 | 23 | 6 | 38% | 17 |
| 470 | histone H2A type 1-A | 25092737 | 14 | 3 | 27% | 16 |
| 471 | proteasome subunit alpha type-4 isoform 1 | 4506185 | 29 | 6 | 25% | 16 |
| 472 | protein S100-A9 | 4506773 | 13 | 4 | 46% | 16 |
| 473 | pyruvate dehydrogenase protein X component, mitochondrial isoform 2 | 203098816 | 51 | 7 | 24% | 16 |
| 474 | 6-phosphogluconate dehydrogenase, decarboxylating | 40068518 | 53 | 7 | 21% | 16 |
| 475 | complement component 1 Q subcomponent-binding protein, mitochondrial precursor | 4502491 | 31 | 5 | 32% | 16 |
| 476 | long-chain-fatty-acid--CoA ligase 6 isoform e | 327412327 | 79 | 6 | 13% | 16 |
| 477 | proteasome activator complex subunit 1 isoform 1 | 5453990 | 29 | 5 | 30% | 16 |
| 478 | copper homeostasis protein cutC homolog | 148596990 | 29 | 5 | 31% | 16 |
| 479 | myosin regulatory light chain 12B | 15809016 | 20 | 6 | 45% | 16 |
| 480 | UPF0468 protein C16orf80 | 8392875 | 23 | 3 | 16% | 16 |
| 481 | mesencephalic astrocyte-derived neurotrophic factor precursor | 299523086 | 21 | 6 | 19% | 16 |
| 482 | cAMP-dependent protein kinase catalytic subunit alpha isoform 2 | 46909584 | 40 | 3 | 14% | 16 |
| 483 | ecto-ADP-ribosyltransferase 3 isoform a precursor | 194097380 | 44 | 4 | 11% | 16 |
| 484 | ATP-citrate synthase isoform 1 | 38569421 | 121 | 6 | 3.70% | 16 |
| 485 | ras-related protein Rab-3B | 19923750 | 25 | 5 | 36% | 16 |
| 486 | adenylate kinase 2, mitochondrial isoform a | 4502013 | 26 | 6 | 41% | 16 |
| 487 | glycodelin precursor | 65507501 | 21 | 3 | 24% | 16 |
| 488 | ATP synthase subunit g, mitochondrial | 51479156 | 11 | 5 | 43% | 16 |
| 489 | transmembrane emp24 domain-containing protein 2 precursor | 5803149 | 23 | 4 | 31% | 16 |
| 490 | heme-binding protein 2 | 7657603 | 23 | 4 | 24% | 16 |
| 491 | cadherin-1 preproprotein | 4757960 | 97 | 3 | 3.20% | 16 |
| 492 | transthyretin precursor | 4507725 | 16 | 5 | 51% | 16 |
| 493 | eukaryotic initiation factor 4A-II | 83700235 | 46 | 2 | 21% | 16 |
| 494 | myosin-10 | 41406064 | 229 | 2 | 4.10% | 15 |
| 495 | dynein heavy chain 17, axonemal | 256542310 | 509 | 9 | 2.90% | 15 |
| 496 | dynein heavy chain 8, axonemal | 332688227 | 539 | 10 | 2.50% | 15 |
| 497 | leucine zipper transcription factor-like protein 1 | 9966793 | 35 | 5 | 23% | 15 |
| 498 | melanoma inhibitory activity protein 3 precursor | 122891870 | 214 | 7 | 4.80% | 15 |
| 499 | ADP/ATP translocase 2 | 156071459 | 33 | 3 | 19% | 15 |
| 500 | mRNA export factor | 62739173 | 41 | 6 | 16% | 15 |
| 501 | testis-expressed protein 101 isoform 1 | 194018544 | 29 | 3 | 15% | 15 |
| 502 | voltage-dependent anion-selective channel protein 1 | 4507879 | 31 | 3 | 16% | 15 |
| 503 | proteasome subunit alpha type-3 isoform 2 | 23110939 | 28 | 7 | 34% | 15 |
| 504 | nuclear pore glycoprotein p62 | 24497605 | 53 | 5 | 14% | 15 |
| 505 | zinc-alpha-2-glycoprotein precursor | 4502337 | 34 | 7 | 27% | 15 |
| 506 | hypoxanthine-guanine phosphoribosyltransferase | 4504483 | 25 | 6 | 43% | 15 |
| 507 | L-lactate dehydrogenase B chain | 4557032 | 37 | 3 | 16% | 15 |
| 508 | 26S proteasome non-ATPase regulatory subunit 3 | 25777612 | 61 | 4 | 9.90% | 15 |
| 509 | transmembrane protein 89 precursor | 56847630 | 18 | 4 | 28% | 15 |
| 510 | calcium-binding mitochondrial carrier protein Aralar1 | 21361103 | 75 | 6 | 13% | 15 |
| 511 | izumo sperm-egg fusion protein 2 precursor | 63999117 | 25 | 4 | 14% | 15 |
| 512 | UPF0733 protein C2orf88 | 110349742 | 11 | 6 | 68% | 14 |
| 513 | prostate and testis expressed protein 1 precursor | 19923082 | 14 | 5 | 45% | 14 |
| 514 | thioredoxin isoform 1 | 50592994 | 12 | 3 | 32% | 14 |
| 515 | coiled-coil domain-containing protein 63 | 22749217 | 66 | 6 | 14% | 14 |
| 516 | translin-associated protein X | 5174731 | 33 | 6 | 26% | 14 |
| 517 | dnaJ homolog subfamily A member 2 | 5031741 | 46 | 6 | 24% | 14 |
| 518 | prostasin preproprotein | 4506153 | 36 | 3 | 18% | 14 |
| 519 | eukaryotic translation elongation factor 1 epsilon-1 isoform 1 | 4758862 | 20 | 5 | 41% | 14 |
| 520 | fragile X mental retardation 1 neighbor protein | 22749199 | 29 | 4 | 15% | 14 |
| 521 | diablo homolog, mitochondrial isoform 1 precursor | 9845297 | 27 | 5 | 26% | 14 |
| 522 | cathepsin D preproprotein | 4503143 | 45 | 5 | 18% | 14 |
| 523 | superoxide dismutase [Cu-Zn] | 4507149 | 16 | 5 | 60% | 14 |
| 524 | dnaJ homolog subfamily B member 13 | 39204547 | 36 | 5 | 22% | 14 |
| 525 | uncharacterized protein C15orf26 | 148747373 | 34 | 4 | 18% | 14 |
| 526 | fatty-acid amide hydrolase 1 | 166795287 | 63 | 6 | 12% | 14 |
| 527 | ras-related protein Rab-1A isoform 1 | 4758988 | 23 | 3 | 42% | 14 |
| 528 | enoyl-CoA delta isomerase 2, mitochondrial isoform 2 | 260274832 | 44 | 7 | 23% | 14 |
| 529 | EF-hand domain-containing protein 1 isoform 1 | 156616292 | 74 | 9 | 17% | 13 |
| 530 | exportin-7 | 154448892 | 124 | 6 | 5.80% | 13 |
| 531 | tektin-5 | 21389569 | 56 | 7 | 21% | 13 |
| 532 | regenerating islet-derived protein 3-gamma precursor | 38348213 | 19 | 4 | 34% | 13 |
| 533 | UTP--glucose-1-phosphate uridylyltransferase isoform b | 48255968 | 56 | 5 | 13% | 13 |
| 534 | T-complex protein 11 homolog isoform 1 | 148226214 | 57 | 6 | 23% | 13 |
| 535 | annexin A11 | 22165431 | 54 | 5 | 15% | 13 |
| 536 | superoxide dismutase [Mn], mitochondrial isoform A precursor | 67782305 | 25 | 4 | 32% | 13 |
| 537 | poly(rC)-binding protein 1 | 222352151 | 37 | 3 | 9.30% | 13 |
| 538 | glycogen phosphorylase, brain form | 21361370 | 97 | 5 | 6.20% | 13 |
| 539 | F-actin-capping protein subunit beta isoform 1 | 4826659 | 31 | 5 | 19% | 13 |
| 540 | cystatin-B | 4503117 | 11 | 4 | 55% | 13 |
| 541 | 3-ketoacyl-CoA thiolase, mitochondrial | 167614485 | 42 | 5 | 26% | 13 |
| 542 | UPF0740 protein C1orf192 | 63029930 | 19 | 3 | 22% | 13 |
| 543 | 45 calcium-binding protein isoform 2 precursor | 18699732 | 42 | 6 | 23% | 13 |
| 544 | ras-related protein Rab-5C isoform a | 41393614 | 23 | 5 | 30% | 13 |
| 545 | casein kinase II subunit beta | 23503295 | 25 | 6 | 46% | 13 |
| 546 | dynein light chain 1, axonemal isoform 1 | 164607156 | 22 | 6 | 28% | 13 |
| 547 | HD domain-containing protein 2 | 116875826 | 23 | 5 | 36% | 13 |
| 548 | translocon-associated protein subunit alpha precursor | 169404009 | 32 | 3 | 12% | 13 |
| 549 | acyl carrier protein, mitochondrial precursor | 4826852 | 17 | 3 | 21% | 13 |
| 550 | ferritin heavy chain | 56682959 | 21 | 6 | 37% | 13 |
| 551 | thioredoxin-dependent peroxide reductase, mitochondrial isoform b | 32483377 | 26 | 3 | 16% | 13 |
| 552 | COP9 signalosome complex subunit 8 isoform 1 | 5729779 | 23 | 3 | 29% | 13 |
| 553 | apoptosis-inducing factor 1, mitochondrial isoform 2 precursor | 22202629 | 66 | 7 | 16% | 13 |
| 554 | carbonic anhydrase 2 | 4557395 | 29 | 5 | 24% | 13 |
| 555 | LETM1 and EF-hand domain-containing protein 1, mitochondrial precursor | 6912482 | 83 | 5 | 7.60% | 12 |
| 556 | cytoplasmic dynein 1 heavy chain 1 | 33350932 | 532 | 7 | 1.20% | 12 |
| 557 | bifunctional aminoacyl-tRNA synthetase | 62241042 | 171 | 6 | 4.70% | 12 |
| 558 | sperm acrosome membrane-associated protein 4 precursor | 19424138 | 13 | 2 | 22% | 12 |
| 559 | aspartyl aminopeptidase | 156416028 | 53 | 4 | 15% | 12 |
| 560 | gamma-glutamyltranspeptidase 1 precursor | 73915090 | 61 | 4 | 6.30% | 12 |
| 561 | dnaJ homolog subfamily B member 1 | 5453690 | 38 | 4 | 14% | 12 |
| 562 | coiled-coil domain-containing protein 105 | 226492892 | 57 | 7 | 16% | 12 |
| 563 | malate dehydrogenase, cytoplasmic isoform 1 | 312283701 | 39 | 4 | 18% | 12 |
| 564 | erythrocyte band 7 integral membrane protein isoform a | 38016911 | 32 | 4 | 20% | 12 |
| 565 | 60S acidic ribosomal protein P2 | 4506671 | 12 | 4 | 59% | 12 |
| 566 | protein NipSnap homolog 3A | 22267436 | 28 | 5 | 28% | 12 |
| 567 | programmed cell death protein 6 | 7019485 | 22 | 6 | 45% | 12 |
| 568 | cytochrome c oxidase subunit 6B1 | 4502985 | 10 | 4 | 34% | 12 |
| 569 | tryptophanyl-tRNA synthetase, cytoplasmic isoform b | 47419918 | 49 | 4 | 17% | 12 |
| 570 | nucleoporin p58/p45 isoform a | 30102928 | 61 | 5 | 15% | 12 |
| 571 | stromal cell-derived factor 2-like protein 1 precursor | 56243533 | 24 | 3 | 20% | 12 |
| 572 | speriolin isoform 1 | 197276668 | 62 | 4 | 9.80% | 12 |
| 573 | A disintegrin and metalloproteinase with thrombospondin motifs 1 preproprotein | 50845384 | 105 | 4 | 6.80% | 12 |
| 574 | general vesicular transport factor p115 | 4505541 | 108 | 5 | 5.50% | 12 |
| 575 | prenylated Rab acceptor protein 1 | 222144309 | 21 | 3 | 18% | 12 |
| 576 | nucleotide exchange factor SIL1 precursor | 11968009 | 52 | 5 | 13% | 12 |
| 577 | 26S proteasome non-ATPase regulatory subunit 8 | 156631005 | 40 | 6 | 23% | 12 |
| 578 | 3-hydroxyacyl-CoA dehydrogenase type-2 isoform 1 . | 4758504 | 27 | 4 | 15% | 12 |
| 579 | acylamino-acid-releasing enzyme | 23510451 | 81 | 6 | 11% | 12 |
| 580 | ubiquitin thioesterase OTUB1 | 109148508 | 31 | 6 | 25% | 12 |
| 581 | serine protease inhibitor Kazal-type 2 precursor | 10863911 | 9 | 2 | 36% | 12 |
| 582 | dnaJ homolog subfamily B member 8 | 23503241 | 26 | 4 | 22% | 11 |
| 583 | EGF-like repeat and discoidin I-like domain-containing protein 3 precursor | 31317224 | 54 | 5 | 14% | 11 |
| 584 | eukaryotic translation initiation factor 3 subunit I | 4503513 | 37 | 4 | 22% | 11 |
| 585 | CD177 antigen precursor | 110735433 | 46 | 4 | 15% | 11 |
| 586 | kinectin isoform a | 33620775 | 156 | 9 | 7.70% | 11 |
| 587 | serine protease 58 precursor | 48255915 | 27 | 4 | 18% | 11 |
| 588 | septin-7 isoform 2 | 148352329 | 51 | 4 | 16% | 11 |
| 589 | nucleoporin NUP53 | 31982904 | 35 | 4 | 19% | 11 |
| 590 | ubiquitin carboxyl-terminal hydrolase 14 isoform b | 82880645 | 52 | 5 | 16% | 11 |
| 591 | signal peptidase complex catalytic subunit SEC11A | 7657609 | 21 | 4 | 31% | 11 |
| 592 | mitochondrial dicarboxylate carrier | 20149598 | 31 | 4 | 18% | 11 |
| 593 | serine/threonine-protein phosphatase PGAM5, mitochondrial isoform 1 | 281604136 | 32 | 2 | 7.60% | 11 |
| 594 | aspartate aminotransferase, cytoplasmic | 4504067 | 46 | 5 | 24% | 11 |
| 595 | actin-related protein M1 | 221139714 | 41 | 5 | 18% | 11 |
| 596 | syntaxin-12 | 28933465 | 32 | 4 | 27% | 11 |
| 597 | leucine-rich repeat-containing protein 23 isoform a | 42542396 | 40 | 4 | 21% | 11 |
| 598 | gamma-glutamyl hydrolase precursor | 4503987 | 36 | 3 | 12% | 11 |
| 599 | elongation factor 1-beta | 4503477 | 25 | 3 | 30% | 11 |
| 600 | serine/threonine-protein phosphatase 2A 65 regulatory subunit A alpha isoform | 21361399 | 65 | 5 | 9.80% | 11 |
| 601 | purine nucleoside phosphorylase | 157168362 | 32 | 4 | 16% | 11 |
| 602 | voltage-dependent calcium channel subunit alpha-2/delta-2 isoform c | 291290994 | 130 | 5 | 5.20% | 11 |
| 603 | UMP-CMP kinase isoform a | 7706497 | 26 | 4 | 24% | 11 |
| 604 | carnitine O-acetyltransferase precursor | 21618331 | 71 | 4 | 9.30% | 11 |
| 605 | phospholipase A1 member A isoform 2 precursor | 332688256 | 48 | 3 | 10% | 11 |
| 606 | actin-like protein 9 | 194097462 | 46 | 5 | 21% | 11 |
| 607 | chloride intracellular channel protein 4 | 7330335 | 29 | 4 | 22% | 11 |
| 608 | GTP-binding protein SAR1b | 7705827 | 22 | 2 | 35% | 11 |
| 609 | alpha-mannosidase 2C1 | 46852164 | 116 | 4 | 5.40% | 10 |
| 610 | vimentin | 62414289 | 54 | 5 | 14% | 10 |
| 611 | alpha-1-antitrypsin precursor | 50363217 | 47 | 4 | 12% | 10 |
| 612 | aldose reductase | 4502049 | 36 | 4 | 20% | 10 |
| 613 | NADH dehydrogenase [ubiquinone] 1 alpha subcomplex subunit 8 | 7657369 | 20 | 3 | 22% | 10 |
| 614 | slit homolog 2 protein precursor | 4759146 | 170 | 6 | 6.40% | 10 |
| 615 | testis, prostate and placenta-expressed protein isoform 2 precursor | 154759245 | 31 | 5 | 29% | 10 |
| 616 | transcription elongation factor B polypeptide 2 isoform b | 46276893 | 18 | 4 | 34% | 10 |
| 617 | dynactin subunit 1 isoform 1 | 13259510 | 142 | 5 | 3.80% | 10 |
| 618 | heterogeneous nuclear ribonucleoprotein M isoform a | 14141152 | 78 | 6 | 9.70% | 10 |
| 619 | methionyl-tRNA synthetase, cytoplasmic | 14043022 | 101 | 5 | 8.40% | 10 |
| 620 | tubulin polymerization-promoting protein family member 2 | 226491350 | 19 | 5 | 48% | 10 |
| 621 | anterior gradient protein 2 homolog precursor | 5453541 | 20 | 4 | 33% | 10 |
| 622 | proteasome activator complex subunit 4 | 163644283 | 211 | 5 | 3.60% | 10 |
| 623 | transcription elongation factor B polypeptide 1 isoform a | 325652033 | 12 | 5 | 51% | 10 |
| 624 | tomoregulin-2 precursor | 12383051 | 41 | 4 | 11% | 10 |
| 625 | serine/threonine-protein phosphatase 2A catalytic subunit alpha isoform | 4506017 | 36 | 4 | 21% | 10 |
| 626 | uncharacterized protein C9orf171 | 46409466 | 36 | 4 | 14% | 10 |
| 627 | apolipoprotein D precursor | 4502163 | 21 | 4 | 22% | 10 |
| 628 | EF-hand calcium-binding domain-containing protein 1 isoform a | 13375787 | 24 | 4 | 27% | 10 |
| 629 | tropomyosin alpha-3 chain isoform 2 | 24119203 | 29 | 3 | 17% | 10 |
| 630 | ran-specific GTPase-activating protein | 4506407 | 23 | 2 | 10.00% | 10 |
| 631 | nucleophosmin isoform 1 | 10835063 | 33 | 2 | 17% | 10 |
| 632 | protein DPCD | 39930355 | 23 | 5 | 34% | 10 |
| 633 | NADH dehydrogenase [ubiquinone] 1 beta subcomplex subunit 7 | 10764847 | 16 | 4 | 27% | 10 |
| 634 | midkine precursor | 4505135 | 16 | 2 | 18% | 10 |
| 635 | puromycin-sensitive aminopeptidase | 158937236 | 103 | 5 | 2.70% | 10 |
| 636 | ATP synthase subunit epsilon, mitochondrial | 5901896 | 6 | 3 | 45% | 10 |
| 637 | GLIPR1-like protein 1 precursor | 22749527 | 26 | 4 | 16% | 10 |
| 638 | acyl-protein thioesterase 1 | 5453722 | 25 | 4 | 32% | 10 |
| 639 | NADH dehydrogenase [ubiquinone] flavoprotein 1, mitochondrial isoform 1 precursor | 20149568 | 51 | 4 | 13% | 9 |
| 640 | calmegin precursor | 4758004 | 70 | 4 | 9.00% | 9 |
| 641 | EF-hand domain-containing family member C2 | 31542743 | 87 | 4 | 3.10% | 9 |
| 642 | testis-specific H1 histone | 32401437 | 28 | 2 | 9.40% | 9 |
| 643 | leukocyte surface antigen CD47 isoform 1 precursor | 4502673 | 35 | 2 | 5.90% | 9 |
| 644 | uncharacterized protein C2orf57 | 284413778 | 42 | 4 | 15% | 9 |
| 645 | histone H1t . | 20544168 | 22 | 3 | 17% | 9 |
| 646 | V-type proton ATPase catalytic subunit A | 19913424 | 68 | 3 | 8.60% | 9 |
| 647 | dnaJ homolog subfamily A member 4 isoform 2 | 194328760 | 45 | 5 | 17% | 9 |
| 648 | phosphate carrier protein, mitochondrial isoform b precursor | 47132595 | 40 | 3 | 9.70% | 9 |
| 649 | gelsolin isoform a precursor | 4504165 | 86 | 3 | 4.00% | 9 |
| 650 | disintegrin and metalloproteinase domain-containing protein 7 preproprotein | 114326453 | 86 | 4 | 7.30% | 9 |
| 651 | 26S proteasome non-ATPase regulatory subunit 4 | 5292161 | 41 | 5 | 18% | 9 |
| 652 | pro-cathepsin H preproprotein | 23110955 | 37 | 2 | 8.40% | 9 |
| 653 | ras-related protein Ral-A precursor | 33946329 | 24 | 3 | 18% | 9 |
| 654 | dnaJ homolog subfamily B member 6 isoform b | 4885495 | 27 | 2 | 14% | 9 |
| 655 | probable inactive serine protease 37 isoform 1 precursor | 285394164 | 26 | 3 | 16% | 9 |
| 656 | metalloproteinase inhibitor 1 precursor | 4507509 | 23 | 3 | 27% | 9 |
| 657 | UBX domain-containing protein 11 isoform 1 | 116734681 | 54 | 3 | 11% | 9 |
| 658 | glutathione S-transferase Mu 1 isoform 1 | 23065544 | 26 | 2 | 14% | 9 |
| 659 | retinoid-inducible serine carboxypeptidase precursor | 11055992 | 51 | 3 | 5.50% | 9 |
| 660 | sulfatase-modifying factor 2 isoform e precursor | 194248090 | 39 | 3 | 11% | 9 |
| 661 | 60S ribosomal protein L7a | 4506661 | 30 | 3 | 15% | 9 |
| 662 | cytochrome c oxidase subunit 7A2, mitochondrial precursor | 262118227 | 13 | 3 | 22% | 9 |
| 663 | eukaryotic translation initiation factor 6 isoform a | 4504771 | 27 | 3 | 20% | 9 |
| 664 | peptidyl-prolyl cis-trans isomerase F, mitochondrial precursor | 5031987 | 22 | 3 | 21% | 9 |
| 665 | ras-related protein Rab-6A isoform b | 38679888 | 24 | 3 | 23% | 9 |
| 666 | actin-like protein 7A | 5729720 | 49 | 4 | 15% | 9 |
| 667 | fumarylacetoacetate hydrolase domain-containing protein 2B | 40786394 | 35 | 3 | 17% | 9 |
| 668 | retinal dehydrogenase 1 | 21361176 | 55 | 5 | 17% | 9 |
| 669 | izumo sperm-egg fusion protein 1 precursor | 194097475 | 39 | 4 | 22% | 9 |
| 670 | normal mucosa of esophagus-specific gene 1 protein | 37694067 | 10 | 4 | 29% | 9 |
| 671 | UPF0587 protein C1orf123 | 8923541 | 18 | 2 | 25% | 9 |
| 672 | cell division control protein 42 homolog isoform 1 | 4757952 | 21 | 3 | 26% | 9 |
| 673 | F-actin-capping protein subunit alpha-3 | 15277417 | 35 | 3 | 12% | 9 |
| 674 | bifunctional purine biosynthesis protein PURH | 20127454 | 65 | 4 | 13% | 9 |
| 675 | cancer/testis antigen 47A | 121949786 | 30 | 3 | 15% | 9 |
| 676 | nitrilase homolog 1 isoform 3 | 297632348 | 34 | 3 | 9.60% | 9 |
| 677 | ras-related C3 botulinum toxin substrate 1 isoform Rac1b | 9845509 | 23 | 4 | 13% | 9 |
| 678 | NADH-cytochrome b5 reductase 3 isoform 2 | 193794826 | 32 | 3 | 23% | 9 |
| 679 | sialidase-1 precursor | 4557791 | 45 | 3 | 12% | 9 |
| 680 | coiled-coil domain-containing protein 19, mitochondrial precursor | 81295816 | 66 | 4 | 10.00% | 8 |
| 681 | 26S proteasome non-ATPase regulatory subunit 1 isoform 1 | 25777600 | 106 | 3 | 5.00% | 8 |
| 682 | cytochrome c | 11128019 | 12 | 3 | 31% | 8 |
| 683 | succinyl-CoA ligase [ADP-forming] subunit beta, mitochondrial precursor | 11321583 | 50 | 3 | 7.80% | 8 |
| 684 | large proline-rich protein BAG6 isoform a | 149158692 | 119 | 4 | 4.50% | 8 |
| 685 | 10 heat shock protein, mitochondrial | 4504523 | 11 | 4 | 33% | 8 |
| 686 | ras-related protein Rab-7a | 34147513 | 23 | 4 | 13% | 8 |
| 687 | cullin-3 | 4503165 | 89 | 5 | 7.80% | 8 |
| 688 | protein FAM71A | 282721094 | 63 | 3 | 5.40% | 8 |
| 689 | bifunctional ATP-dependent dihydroxyacetone kinase/FAD-AMP lyase (cyclizing) | 20149621 | 59 | 3 | 8.30% | 8 |
| 690 | 60S ribosomal protein L12 | 4506597 | 18 | 4 | 40% | 8 |
| 691 | dihydropteridine reductase | 208973246 | 26 | 3 | 21% | 8 |
| 692 | keratin, type I cytoskeletal 18 | 4557888 | 48 | 2 | 13% | 8 |
| 693 | mitochondrial import receptor subunit TOM34 | 21361356 | 35 | 4 | 19% | 8 |
| 694 | lipase member I | 39752679 | 55 | 3 | 6.70% | 8 |
| 695 | spermatogenesis-associated protein 19, mitochondrial precursor | 28376652 | 19 | 3 | 26% | 8 |
| 696 | proteasome activator complex subunit 2 . | 30410792 | 27 | 4 | 18% | 8 |
| 697 | S-phase kinase-associated protein 1 isoform b | 25777713 | 19 | 4 | 36% | 8 |
| 698 | nucleoside diphosphate kinase 7 isoform a | 7019465 | 42 | 4 | 15% | 8 |
| 699 | atlastin-3 | 45827806 | 61 | 3 | 11% | 8 |
| 700 | uncharacterized protein C6orf81 | 31542280 | 41 | 4 | 19% | 8 |
| 701 | aflatoxin B1 aldehyde reductase member 2 | 41327764 | 40 | 4 | 16% | 8 |
| 702 | four and a half LIM domains protein 1 isoform 1 | 228480211 | 36 | 3 | 11% | 8 |
| 703 | WD repeat-containing protein 65 isoform a | 345199335 | 149 | 4 | 3.50% | 8 |
| 704 | serine protease HTRA1 precursor | 4506141 | 51 | 4 | 14% | 8 |
| 705 | transcriptional activator protein Pur-alpha | 5032007 | 35 | 3 | 18% | 8 |
| 706 | macrophage-capping protein | 63252913 | 38 | 3 | 16% | 8 |
| 707 | cytochrome b5 type B | 83921614 | 17 | 4 | 52% | 8 |
| 708 | integral membrane protein 2B | 11527402 | 30 | 3 | 16% | 8 |
| 709 | dynactin subunit 3 isoform 1 | 6005745 | 21 | 4 | 19% | 8 |
| 710 | intraflagellar transport protein 27 homolog isoform 2 | 6857824 | 20 | 3 | 24% | 8 |
| 711 | neutrophil defensin 3 preproprotein | 4885179 | 10 | 3 | 20% | 7 |
| 712 | protein ERGIC-53 precursor | 5031873 | 58 | 4 | 13% | 7 |
| 713 | nuclear migration protein nudC | 5729953 | 38 | 3 | 11% | 7 |
| 714 | lysosome membrane protein 2 isoform 1 precursor | 5031631 | 54 | 3 | 7.30% | 7 |
| 715 | glyoxylate reductase/hydroxypyruvate reductase | 6912396 | 36 | 3 | 16% | 7 |
| 716 | arfaptin-1 isoform 1 | 71040094 | 42 | 2 | 5.10% | 7 |
| 717 | interferon-inducible GTPase 5 | 10257429 | 50 | 3 | 12% | 7 |
| 718 | serine/threonine-protein phosphatase PP1-beta catalytic subunit isoform 1 | 4506005 | 37 | 2 | 7.60% | 7 |
| 719 | sortilin isoform 1 preproprotein | 17149834 | 92 | 3 | 4.60% | 7 |
| 720 | 40S ribosomal protein S8 | 4506743 | 24 | 3 | 13% | 7 |
| 721 | histidine triad nucleotide-binding protein 2, mitochondrial precursor | 14211923 | 17 | 3 | 31% | 7 |
| 722 | inositol monophosphatase 1 isoform 2 | 221625487 | 37 | 3 | 11% | 7 |
| 723 | mammaglobin-B precursor | 4505171 | 11 | 3 | 40% | 7 |
| 724 | transmembrane protein 205 | 63055043 | 21 | 2 | 16% | 7 |
| 725 | ras-related protein Rab-5B isoform 1 | 354725902 | 24 | 3 | 22% | 7 |
| 726 | carnitine O-palmitoyltransferase 1, muscle isoform isoform a | 223468678 | 88 | 4 | 6.30% | 7 |
| 727 | thiamine-triphosphatase | 13236577 | 26 | 3 | 18% | 7 |
| 728 | myosin light chain 6B | 313851001 | 23 | 2 | 18% | 7 |
| 729 | acid ceramidase isoform b | 189011546 | 47 | 5 | 11% | 7 |
| 730 | 3-mercaptopyruvate sulfurtransferase isoform 2 | 61835204 | 33 | 2 | 14% | 7 |
| 731 | ubiquitin-conjugating enzyme E2 L3 | 4507789 | 18 | 3 | 30% | 7 |
| 732 | endoplasmic reticulum lectin 1 isoform 2 precursor . | 188528696 | 52 | 3 | 11% | 7 |
| 733 | acyl-CoA-binding protein isoform 5 | 295842514 | 14 | 3 | 36% | 7 |
| 734 | 40S ribosomal protein S18 | 11968182 | 18 | 3 | 18% | 7 |
| 735 | ubiquitin thioesterase OTUB2 | 12962939 | 27 | 3 | 16% | 7 |
| 736 | spondin-2 precursor | 6912682 | 36 | 3 | 8.50% | 7 |
| 737 | agouti-related protein precursor | 4501995 | 14 | 2 | 15% | 7 |
| 738 | V-type proton ATPase subunit E 1 isoform a | 4502317 | 26 | 2 | 12% | 7 |
| 739 | sperm-associated antigen 11B isoform D preproprotein | 126131097 | 15 | 2 | 17% | 7 |
| 740 | dynein intermediate chain 1, axonemal | 6912338 | 79 | 4 | 6.20% | 7 |
| 741 | transmembrane protein 126A isoform 1 . | 14150017 | 22 | 2 | 24% | 7 |
| 742 | retinol dehydrogenase 11 isoform 1 precursor | 166795268 | 35 | 3 | 15% | 7 |
| 743 | heat shock 70 protein 4 | 38327039 | 94 | 3 | 4.30% | 7 |
| 744 | 40S ribosomal protein S17 | 4506693 | 16 | 3 | 43% | 7 |
| 745 | platelet-activating factor acetylhydrolase IB subunit beta isoform a | 4505585 | 26 | 3 | 17% | 7 |
| 746 | protein EAN57 isoform 1 | 255759947 | 31 | 3 | 10% | 7 |
| 747 | low molecular weight phosphotyrosine protein phosphatase isoform c | 4757714 | 18 | 3 | 20% | 7 |
| 748 | ankyrin repeat domain-containing protein 45 | 38348298 | 30 | 3 | 14% | 7 |
| 749 | 40S ribosomal protein S4, X isoform X isoform | 4506725 | 30 | 3 | 11% | 7 |
| 750 | cat eye syndrome critical region protein 5 isoform 2 precursor | 14861834 | 46 | 4 | 18% | 7 |
| 751 | profilin-3 | 71274140 | 15 | 2 | 18% | 7 |
| 752 | ADP-ribosylation factor-like protein 3 | 4757774 | 20 | 3 | 28% | 7 |
| 753 | azurocidin preproprotein | 11342670 | 27 | 4 | 32% | 7 |
| 754 | prohibitin-2 isoform 2 | 6005854 | 33 | 3 | 14% | 7 |
| 755 | coiled-coil domain-containing protein 90B, mitochondrial precursor | 20149663 | 30 | 3 | 15% | 7 |
| 756 | endoplasmic reticulum-Golgi intermediate compartment protein 1 | 72534712 | 33 | 3 | 16% | 7 |
| 757 | centrin-1 . | 4757974 | 20 | 3 | 24% | 7 |
| 758 | fructose-1,6-bisphosphatase 1 | 189083692 | 37 | 3 | 14% | 7 |
| 759 | ras-related protein Rab-27A | 34485709 | 25 | 2 | 15% | 7 |
| 760 | myeloid-associated differentiation marker | 19923903 | 35 | 2 | 10% | 7 |
| 761 | NADH dehydrogenase [ubiquinone] iron-sulfur protein 6, mitochondrial precursor | 4758792 | 14 | 3 | 20% | 6 |
| 762 | apolipoprotein A-IV precursor | 71773110 | 45 | 4 | 13% | 6 |
| 763 | calicin | 169636428 | 67 | 4 | 9.90% | 6 |
| 764 | nidogen-1 precursor | 115298674 | 136 | 4 | 4.20% | 6 |
| 765 | V-type proton ATPase subunit B, brain isoform | 19913428 | 57 | 3 | 12% | 6 |
| 766 | nucleosome assembly protein 1-like 4 | 5174613 | 43 | 2 | 8.50% | 6 |
| 767 | UPF0556 protein C19orf10 precursor | 33457348 | 19 | 3 | 22% | 6 |
| 768 | myotrophin | 21956645 | 13 | 2 | 25% | 6 |
| 769 | 72 type IV collagenase isoform a preproprotein | 11342666 | 74 | 2 | 4.70% | 6 |
| 770 | cystatin-C precursor | 4503107 | 16 | 2 | 18% | 6 |
| 771 | PREDICTED: hypothetical protein LOC728597 | 341914388 | 72 | 3 | 4.90% | 6 |
| 772 | trafficking protein particle complex subunit 3 | 7656926 | 20 | 3 | 19% | 6 |
| 773 | complement decay-accelerating factor isoform 2 precursor | 168693643 | 49 | 3 | 10% | 6 |
| 774 | serine/threonine-protein phosphatase 2A activator isoform b | 29725611 | 37 | 2 | 8.00% | 6 |
| 775 | aspartyl-tRNA synthetase, cytoplasmic | 45439306 | 57 | 3 | 9.20% | 6 |
| 776 | uncharacterized protein C9orf135 | 58219541 | 26 | 3 | 21% | 6 |
| 777 | dolichyl-diphosphooligosaccharide--protein glycosyltransferase subunit STT3A | 22749415 | 81 | 3 | 4.30% | 6 |
| 778 | myeloperoxidase precursor | 4557759 | 84 | 3 | 5.50% | 6 |
| 779 | Sjoegren syndrome nuclear autoantigen 1 | 189571687 | 14 | 2 | 20% | 6 |
| 780 | dipeptidase 1 precursor | 4758190 | 46 | 2 | 8.30% | 6 |
| 781 | cytochrome c oxidase subunit 6C proprotein | 4758040 | 9 | 2 | 28% | 6 |
| 782 | dynein light chain roadblock-type 2 | 18702323 | 11 | 2 | 29% | 6 |
| 783 | transketolase-like protein 1 isoform b | 225637461 | 65 | 2 | 5.10% | 6 |
| 784 | transketolase-like protein 2 . | 133778974 | 68 | 3 | 7.80% | 6 |
| 785 | glycerol-3-phosphate dehydrogenase 1-like protein | 24307999 | 38 | 2 | 4.80% | 6 |
| 786 | ectonucleotide pyrophosphatase/phosphodiesterase family member 3 | 111160296 | 100 | 3 | 5.60% | 6 |
| 787 | alcohol dehydrogenase class-3 | 71565154 | 40 | 2 | 4.80% | 6 |
| 788 | 60S ribosomal protein L18 | 4506607 | 22 | 2 | 14% | 6 |
| 789 | cysteine desulfurase, mitochondrial isoform a | 32307132 | 50 | 3 | 10% | 6 |
| 790 | ras-related protein Rap-1b isoform 1 precursor | 7661678 | 21 | 3 | 19% | 6 |
| 791 | arginyl-tRNA synthetase, cytoplasmic | 15149476 | 75 | 3 | 5.00% | 6 |
| 792 | late cornified envelope-like proline-rich protein 1 | 58082087 | 11 | 2 | 42% | 6 |
| 793 | NAD-dependent malic enzyme, mitochondrial isoform 1 precursor [Homo | 4505145 | 65 | 3 | 11% | 6 |
| 794 | protein TSC21 | 22749357 | 21 | 4 | 27% | 6 |
| 795 | nuclear pore complex protein Nup205 | 57634534 | 228 | 4 | 2.30% | 6 |
| 796 | 3-oxoacyl-[acyl-carrier-protein] synthase, mitochondrial isoform 1 | 8923559 | 49 | 3 | 15% | 6 |
| 797 | carboxypeptidase M precursor | 6631081 | 51 | 2 | 3.80% | 6 |
| 798 | heat shock 70 protein 13 precursor | 48928056 | 52 | 2 | 5.50% | 6 |
| 799 | tripeptidyl-peptidase 1 preproprotein | 5729770 | 61 | 2 | 5.70% | 6 |
| 800 | elongation factor Ts, mitochondrial isoform 2 precursor [Homo | 171846268 | 35 | 2 | 14% | 6 |
| 801 | epoxide hydrolase 2 | 27597073 | 63 | 2 | 2.70% | 6 |
| 802 | WAP four-disulfide core domain protein 8 precursor | 153946389 | 28 | 3 | 17% | 6 |
| 803 | ATP synthase subunit e, mitochondrial | 6005717 | 8 | 2 | 30% | 6 |
| 804 | destrin isoform a | 5802966 | 19 | 2 | 17% | 6 |
| 805 | tenascin precursor | 153946395 | 241 | 4 | 2.40% | 6 |
| 806 | peptidase inhibitor 15 preproprotein | 7705676 | 29 | 2 | 10% | 6 |
| 807 | ferritin light chain | 20149498 | 20 | 2 | 18% | 6 |
| 808 | cytochrome b5 isoform 1 | 41281768 | 15 | 2 | 20% | 6 |
| 809 | secretoglobin family 1D member 2 precursor | 5729909 | 10 | 2 | 22% | 6 |
| 810 | vesicle-trafficking protein SEC22b precursor | 94429050 | 25 | 2 | 15% | 6 |
| 811 | ATP synthase subunit s, mitochondrial isoform a precursor [Homo | 51558774 | 25 | 2 | 9.30% | 6 |
| 812 | cornulin | 7706635 | 54 | 2 | 9.70% | 6 |
| 813 | protamine-2 | 68989267 | 13 | 2 | 27% | 6 |
| 814 | uncharacterized protein C19orf71 | 207442677 | 24 | 2 | 14% | 6 |
| 815 | S-formylglutathione hydrolase | 33413400 | 31 | 2 | 16% | 6 |
| 816 | cell cycle control protein 50A isoform 1 | 8922720 | 41 | 3 | 12% | 6 |
| 817 | cytochrome b5 domain-containing protein 1 | 40255060 | 27 | 2 | 11% | 6 |
| 818 | uncharacterized protein LOC100130705 | 304376316 | 20 | 3 | 19% | 6 |
| 819 | neutrophil gelatinase-associated lipocalin precursor | 38455402 | 23 | 4 | 27% | 6 |
| 820 | protein ALEX XLas | 117938759 | 111 | 2 | 2.60% | 6 |
| 821 | transforming protein RhoA precursor | 10835049 | 22 | 3 | 23% | 6 |
| 822 | 40S ribosomal protein S25 | 4506707 | 14 | 2 | 16% | 6 |
| 823 | ATP5J2-PTCD1 fusion protein | 311893396 | 84 | 2 | 3.20% | 6 |
| 824 | beta-hexosaminidase subunit alpha preproprotein | 189181666 | 61 | 2 | 4.20% | 6 |
| 825 | 60S ribosomal protein L24 | 4506619 | 18 | 2 | 13% | 6 |
| 826 | out at first protein homolog precursor | 30425438 | 31 | 2 | 10% | 6 |
| 827 | NSFL1 cofactor p47 isoform d | 332078466 | 41 | 2 | 7.00% | 6 |
| 828 | 26S proteasome non-ATPase regulatory subunit 12 isoform 1 | 4506221 | 53 | 2 | 7.50% | 5 |
| 829 | hornerin | 57864582 | 282 | 3 | 2.30% | 5 |
| 830 | sarcoplasmic/endoplasmic reticulum calcium ATPase 2 isoform b | 24638454 | 115 | 3 | 4.00% | 5 |
| 831 | peptidyl-prolyl cis-trans isomerase FKBP4 | 4503729 | 52 | 2 | 6.10% | 5 |
| 832 | 26S proteasome non-ATPase regulatory subunit 6 | 7661914 | 46 | 3 | 11% | 5 |
| 833 | histone H2A-Bbd type 2/3 | 63029935 | 13 | 2 | 30% | 5 |
| 834 | calpain-1 catalytic subunit | 311893363 | 82 | 2 | 3.10% | 5 |
| 835 | cytochrome b-c1 complex subunit 6, mitochondrial | 83627705 | 11 | 2 | 27% | 5 |
| 836 | cystatin-S precursor | 4503109 | 16 | 2 | 18% | 5 |
| 837 | glycyl-tRNA synthetase precursor | 116805340 | 83 | 3 | 4.60% | 5 |
| 838 | 60S ribosomal protein L7 | 15431301 | 29 | 3 | 18% | 5 |
| 839 | GTP-binding protein SAR1a | 217416369 | 22 | 3 | 15% | 5 |
| 840 | importin-4 | 62460637 | 119 | 3 | 3.50% | 5 |
| 841 | CMT1A duplicated region transcript 15 protein | 56090618 | 21 | 2 | 21% | 5 |
| 842 | translocon-associated protein subunit delta isoform 3 precursor | 325301078 | 20 | 2 | 13% | 5 |
| 843 | apolipoprotein O precursor | 13129148 | 22 | 2 | 11% | 5 |
| 844 | thioredoxin domain-containing protein 17 | 14249348 | 14 | 2 | 15% | 5 |
| 845 | UPF0577 protein KIAA1324 precursor | 38569482 | 111 | 3 | 3.60% | 5 |
| 846 | homogentisate 1,2-dioxygenase | 115527117 | 50 | 2 | 8.10% | 5 |
| 847 | 60S ribosomal protein L11 isoform 1 | 15431290 | 20 | 2 | 13% | 5 |
| 848 | NADH dehydrogenase [ubiquinone] iron-sulfur protein 4, mitochondrial precursor | 4505369 | 20 | 3 | 19% | 5 |
| 849 | ADP-ribosylation factor-like protein 8B | 8922601 | 22 | 3 | 22% | 5 |
| 850 | uncharacterized protein C22orf43 | 56118955 | 25 | 2 | 9.60% | 5 |
| 851 | guanine nucleotide-binding protein subunit beta-2-like 1 | 5174447 | 35 | 2 | 8.50% | 5 |
| 852 | SPRY domain-containing protein 7 isoform 1 | 20531765 | 22 | 3 | 29% | 5 |
| 853 | B-cell receptor-associated protein 31 isoform a | 213511508 | 35 | 2 | 6.40% | 5 |
| 854 | endoplasmic reticulum-Golgi intermediate compartment protein 2 | 50959176 | 43 | 2 | 6.90% | 5 |
| 855 | ubiquitin carboxyl-terminal hydrolase isozyme L1 | 21361091 | 25 | 2 | 14% | 5 |
| 856 | uncharacterized protein C20orf85 . | 30425380 | 16 | 2 | 21% | 5 |
| 857 | NADH dehydrogenase [ubiquinone] 1 beta subcomplex subunit 8, mitochondrial precursor | 4826854 | 22 | 3 | 21% | 5 |
| 858 | coiled-coil domain-containing protein 164 | 217416374 | 87 | 3 | 4.70% | 5 |
| 859 | protein phosphatase 1 regulatory subunit 32 isoform 1 | 283046778 | 47 | 2 | 6.10% | 5 |
| 860 | outer dense fiber protein 3B . | 116292180 | 27 | 2 | 8.70% | 5 |
| 861 | 15 selenoprotein isoform 1 precursor | 42741648 | 18 | 2 | 18% | 5 |
| 862 | probable ergosterol biosynthetic protein 28 | 6005719 | 16 | 2 | 17% | 5 |
| 863 | SUN domain-containing protein 3 | 71834868 | 41 | 3 | 8.10% | 5 |
| 864 | uncharacterized protein C1orf194 . | 171916088 | 18 | 3 | 36% | 5 |
| 865 | protein NDRG3 isoform a | 14165266 | 41 | 3 | 14% | 5 |
| 866 | 60S ribosomal protein L28 isoform 2 | 13904866 | 16 | 3 | 22% | 5 |
| 867 | 60S ribosomal protein L14 | 78000181 | 23 | 2 | 11% | 5 |
| 868 | platelet-activating factor acetylhydrolase IB subunit alpha | 4557741 | 47 | 2 | 9.50% | 5 |
| 869 | up-regulated during skeletal muscle growth protein 5 | 14249376 | 6 | 2 | 43% | 5 |
| 870 | lysozyme-like protein 6 precursor | 9966905 | 17 | 2 | 24% | 5 |
| 871 | glycerol kinase isoform a | 42794763 | 58 | 2 | 6.00% | 5 |
| 872 | ATP synthase-coupling factor 6, mitochondrial isoform a precursor | 18644883 | 13 | 2 | 31% | 5 |
| 873 | mitochondrial fission 1 protein . | 151108473 | 17 | 3 | 26% | 5 |
| 874 | protein CutA isoform 1 | 62198241 | 21 | 2 | 21% | 5 |
| 875 | sperm-associated antigen 11B isoform A preproprotein | 7706551 | 11 | 2 | 19% | 5 |
| 876 | fibrinogen-like protein 1 precursor . | 42544189 | 36 | 3 | 9.90% | 5 |
| 877 | thioredoxin domain-containing protein 12 precursor | 7705696 | 19 | 2 | 14% | 5 |
| 878 | arrestin domain-containing protein 5 . | 122937478 | 38 | 2 | 8.20% | 5 |
| 879 | beta-defensin 105 precursor | 103485141 | 9 | 2 | 26% | 5 |
| 880 | protein transport protein Sec61 subunit beta | 5803165 | 10 | 2 | 27% | 5 |
| 881 | uncharacterized protein LOC145788 | 311771669 | 14 | 3 | 36% | 5 |
| 882 | cytoskeleton-associated protein 4 | 19920317 | 66 | 2 | 5.60% | 5 |
| 883 | programmed cell death 6-interacting protein isoform 1 | 22027538 | 96 | 2 | 3.30% | 5 |
| 884 | apolipoprotein A-I-binding protein precursor | 91984773 | 32 | 2 | 10% | 5 |
| 885 | glucosamine-6-phosphate isomerase 1 | 13027378 | 33 | 3 | 13% | 5 |
| 886 | isoleucyl-tRNA synthetase, cytoplasmic | 94721239 | 145 | 2 | 1.60% | 5 |
| 887 | histidine triad nucleotide-binding protein 3 | 189217863 | 20 | 2 | 13% | 5 |
| 888 | RIB43A-like with coiled-coils protein 1 isoform 1 | 72534784 | 44 | 3 | 16% | 5 |
| 889 | 40S ribosomal protein S7 | 4506741 | 22 | 2 | 18% | 5 |
| 890 | reactive oxygen species modulator 1 | 18152785 | 8 | 2 | 35% | 5 |
| 891 | beta-defensin 106 precursor | 22748611 | 7 | 2 | 23% | 5 |
| 892 | lipolysis-stimulated lipoprotein receptor isoform 1 | 45505163 | 70 | 2 | 4.60% | 5 |
| 893 | protein S100-A6 | 7657532 | 10 | 2 | 17% | 5 |
| 894 | synaptogyrin-1 isoform 1b | 22035698 | 21 | 3 | 23% | 5 |
| 895 | 40S ribosomal protein S28 . | 4506715 | 8 | 2 | 30% | 5 |
| 896 | torsin-1A-interacting protein 1 | 39753957 | 66 | 2 | 4.50% | 5 |
| 897 | deoxyguanosine kinase, mitochondrial isoform a precursor | 18426967 | 32 | 3 | 18% | 4 |
| 898 | adenylate kinase domain-containing protein 1 isoform 1 | 237858799 | 221 | 2 | 0.99% | 4 |
| 899 | glucosamine--fructose-6-phosphate aminotransferase [isomerizing] 1 isoform 1 | 347659028 | 79 | 4 | 8.00% | 4 |
| 900 | basigin isoform 2 precursor | 38372925 | 29 | 3 | 16% | 4 |
| 901 | isovaleryl-CoA dehydrogenase, mitochondrial isoform 1 precursor | 226958412 | 47 | 3 | 10% | 4 |
| 902 | coiled-coil domain-containing protein 151 | 117553613 | 69 | 3 | 5.70% | 4 |
| 903 | matrilin-2 isoform a precursor | 62548860 | 107 | 2 | 2.70% | 4 |
| 904 | profilin-2 isoform a | 16753215 | 15 | 2 | 19% | 4 |
| 905 | BAG family molecular chaperone regulator 5 isoform b | 6631077 | 51 | 2 | 6.00% | 4 |
| 906 | 40S ribosomal protein S9 | 14141193 | 23 | 2 | 8.80% | 4 |
| 907 | coiled-coil domain-containing protein 42A isoform 1 | 226494053 | 38 | 2 | 9.50% | 4 |
| 908 | 40S ribosomal protein S16 | 4506691 | 16 | 2 | 14% | 4 |
| 909 | acyl-coenzyme A thioesterase 13 isoform 2 | 231567183 | 12 | 2 | 26% | 4 |
| 910 | vitronectin precursor | 88853069 | 54 | 2 | 6.50% | 4 |
| 911 | thiosulfate sulfurtransferase/rhodanese-like domain-containing protein 1 isoform 1 | 163965377 | 13 | 2 | 20% | 4 |
| 912 | titin isoform N2-A | 291045225 | 3713 | 2 | 0.05% | 4 |
| 913 | 60S ribosomal protein L22 proprotein | 4506613 | 15 | 2 | 19% | 4 |
| 914 | 40S ribosomal protein S20 isoform 1 | 226246671 | 16 | 2 | 19% | 4 |
| 915 | protein sel-1 homolog 1 isoform 1 precursor | 19923669 | 89 | 2 | 4.90% | 4 |
| 916 | L-amino-acid oxidase isoform 2 precursor | 27477089 | 65 | 3 | 9.20% | 4 |
| 917 | chitinase domain-containing protein 1 isoform a | 218083142 | 45 | 2 | 9.70% | 4 |
| 918 | coiled-coil domain-containing protein 40 isoform 1 | 148664197 | 130 | 3 | 3.00% | 4 |
| 919 | platelet-activating factor acetylhydrolase precursor | 270133071 | 50 | 2 | 9.80% | 4 |
| 920 | glutathione S-transferase omega-2 isoform 1 | 38016131 | 28 | 2 | 9.50% | 4 |
| 921 | ethanolamine-phosphate cytidylyltransferase isoform 1 | 296841136 | 46 | 2 | 6.90% | 4 |
| 922 | mitochondrial 2-oxoglutarate/malate carrier protein isoform 1 | 21361114 | 34 | 2 | 7.00% | 4 |
| 923 | vesicle-associated membrane protein 8 | 14043026 | 11 | 2 | 23% | 4 |
| 924 | cytochrome c oxidase subunit 7C, mitochondrial precursor | 4502993 | 7 | 2 | 29% | 4 |
| 925 | eukaryotic translation initiation factor 3 subunit K | 10801345 | 25 | 2 | 11% | 4 |
| 926 | proactivator polypeptide isoform a preproprotein | 11386147 | 58 | 2 | 3.40% | 4 |
| 927 | transmembrane emp24 domain-containing protein 1 precursor | 5803040 | 25 | 2 | 12% | 4 |
| 928 | iron-sulfur cluster assembly enzyme ISCU, mitochondrial isoform ISCU2 precursor | 56699456 | 18 | 2 | 16% | 4 |
| 929 | COP9 signalosome complex subunit 7b | 12232385 | 30 | 2 | 10% | 4 |
| 930 | PITH domain-containing protein 1 | 21361837 | 24 | 2 | 18% | 4 |
| 931 | 40S ribosomal protein S27 | 4506711 | 9 | 2 | 25% | 4 |
| 932 | uncharacterized protein C6orf163 | 256600247 | 39 | 2 | 9.10% | 4 |
| 933 | beta-defensin 126 preproprotein . | 13624333 | 12 | 2 | 8.10% | 4 |
| 934 | uncharacterized protein C2orf77 | 146260271 | 66 | 3 | 6.70% | 4 |
| 935 | uncharacterized protein C2orf74 isoform 1 . | 221139892 | 22 | 2 | 18% | 4 |
| 936 | uncharacterized protein C7orf72 | 332634960 | 50 | 2 | 11% | 4 |
| 937 | suppressor of G2 allele of SKP1 homolog isoform SGT1B [Homo | 195963398 | 41 | 3 | 11% | 4 |
| 938 | 40S ribosomal protein S12 | 14277700 | 15 | 2 | 14% | 4 |
| 939 | acetyl-CoA acetyltransferase, cytosolic | 148539872 | 41 | 2 | 7.80% | 4 |
| 940 | exportin-2 | 29029559 | 110 | 2 | 2.40% | 4 |
| 941 | 60S acidic ribosomal protein P1 isoform 1 | 4506669 | 12 | 2 | 57% | 4 |
| 942 | biglycan preproprotein | 4502403 | 42 | 2 | 8.70% | 4 |
| 943 | uncharacterized protein C19orf18 precursor | 22748999 | 24 | 2 | 9.80% | 4 |
| 944 | retinoic acid receptor responder protein 1 isoform 2 precursor | 46255041 | 26 | 2 | 9.60% | 4 |
| 945 | uncharacterized protein C2orf61 isoform 1 . | 254675143 | 28 | 2 | 13% | 4 |
| 946 | dickkopf-like protein 1 isoform 2 precursor | 308818218 | 24 | 2 | 9.50% | 4 |
| 947 | actin-related protein 2/3 complex subunit 4 isoform c | 311771647 | 22 | 2 | 11% | 4 |
| 948 | glutaminyl-peptide cyclotransferase precursor | 6912618 | 41 | 2 | 6.90% | 4 |
| 949 | UPF0573 protein C2orf70 | 157502167 | 23 | 2 | 17% | 4 |
| 950 | ubiquitin-conjugating enzyme E2 variant 1 isoform d | 73765546 | 16 | 2 | 18% | 4 |
| 951 | 40S ribosomal protein S11 | 4506681 | 18 | 2 | 11% | 4 |
| 952 | 60S ribosomal protein L30 | 4506631 | 13 | 3 | 31% | 4 |
| 953 | transketolase | 205277463 | 68 | 3 | 9.50% | 4 |
| 954 | tropomyosin alpha-4 chain isoform 1 | 223555975 | 33 | 2 | 8.10% | 4 |
| 955 | disintegrin and metalloproteinase domain-containing protein 32 | 148664238 | 88 | 2 | 3.30% | 4 |
| 956 | 60S ribosomal protein L35a | 16117791 | 13 | 3 | 22% | 4 |
| 957 | inorganic pyrophosphatase | 11056044 | 33 | 3 | 18% | 4 |
| 958 | phosphatidylinositol-5-phosphate 4-kinase type-2 gamma isoform a | 226371739 | 47 | 3 | 11% | 4 |
| 959 | prostaglandin reductase 1 isoform 1 | 226059133 | 36 | 2 | 5.50% | 4 |
| 960 | 2-oxoisovalerate dehydrogenase subunit alpha, mitochondrial isoform | 11386135 | 50 | 2 | 8.50% | 4 |
| 961 | nucleoporin NDC1 isoform 1 | 271398350 | 76 | 2 | 4.70% | 4 |
| 962 | cytochrome b-c1 complex subunit 9 isoform a | 41281885 | 7 | 2 | 38% | 4 |
| 963 | NADH dehydrogenase [ubiquinone] 1 beta subcomplex subunit 11, mitochondrial isoform 1 | 20127561 | 18 | 2 | 11% | 4 |
| 964 | NADH dehydrogenase [ubiquinone] 1 beta subcomplex subunit 9 isoform | 6274550 | 22 | 2 | 16% | 4 |
| 965 | phosphatidylinositol transfer protein alpha isoform . | 5453908 | 32 | 2 | 13% | 4 |
| 966 | ran guanine nucleotide release factor isoform B . | 295317377 | 16 | 2 | 21% | 4 |
| 967 | dehydrogenase/reductase SDR family member 7B | 20149619 | 35 | 2 | 8.90% | 4 |
| 968 | isochorismatase domain-containing protein 1 . | 103471987 | 32 | 2 | 9.70% | 4 |
| 969 | ubiquitin-conjugating enzyme E2 N | 4507793 | 17 | 2 | 23% | 3 |
| 970 | serine/threonine-protein phosphatase with EF-hands 1 isoform 1b | 23312374 | 73 | 2 | 4.60% | 3 |
| 971 | thioredoxin reductase 2, mitochondrial precursor | 22035672 | 57 | 3 | 13% | 3 |
| 972 | non-specific lipid-transfer protein isoform 1 proprotein | 19923233 | 59 | 2 | 4.20% | 3 |
| 973 | DNA damage-binding protein 1 | 148529014 | 127 | 2 | 1.80% | 3 |
| 974 | 60S ribosomal protein L23 | 4506605 | 15 | 2 | 17% | 3 |
| 975 | protein FAM162A | 49355721 | 17 | 2 | 20% | 3 |
| 976 | prostate and testis expressed protein 2 precursor | 47086459 | 13 | 3 | 29% | 3 |
| 977 | poly(rC)-binding protein 2 isoform b | 14141166 | 38 | 2 | 9.70% | 3 |
| 978 | vacuolar protein sorting-associated protein 13A isoform A | 66346674 | 360 | 2 | 0.85% | 3 |
| 979 | NADH dehydrogenase [ubiquinone] 1 alpha subcomplex subunit 5 | 4826848 | 13 | 2 | 8.60% | 3 |
| 980 | clathrin light chain A isoform a . | 4502899 | 24 | 2 | 14% | 3 |
| 981 | protein BRICK1 | 27544939 | 9 | 2 | 28% | 3 |
| 982 | neuropilin-1 isoform a precursor | 182508169 | 103 | 2 | 3.40% | 3 |
| 983 | kinesin-1 heavy chain | 4758648 | 110 | 2 | 2.60% | 3 |
| 984 | dnaJ homolog subfamily C member 3 precursor | 5453980 | 58 | 2 | 10% | 3 |
| 985 | nucleosome assembly protein 1-like 1 | 4758756 | 45 | 2 | 6.10% | 3 |
| 986 | metaxin-2 | 5729937 | 30 | 2 | 11% | 3 |
| 987 | NADH dehydrogenase [ubiquinone] iron-sulfur protein 5 | 4758790 | 13 | 2 | 18% | 3 |
| 988 | myosin-VI | 92859701 | 149 | 2 | 1.90% | 3 |
| 989 | ribose-phosphate pyrophosphokinase 2 isoform 2 | 4506129 | 35 | 3 | 11% | 3 |
| 990 | cartilage acidic protein 1 isoform B precursor . | 330688397 | 70 | 2 | 6.70% | 3 |
| 991 | polyadenylate-binding protein 1 . | 46367787 | 71 | 3 | 5.80% | 3 |
| 992 | importin subunit alpha-4 . | 34485722 | 58 | 2 | 7.30% | 3 |
| 993 | peptidyl-prolyl cis-trans isomerase FKBP2 precursor . | 17149842 | 16 | 2 | 18% | 3 |
| 994 | secreted frizzled-related protein 1 precursor . | 56117838 | 35 | 2 | 8.90% | 3 |
| 995 | eukaryotic translation initiation factor 3 subunit M | 23397429 | 43 | 2 | 8.00% | 3 |
| 996 | heat shock protein beta-11 | 118343647 | 16 | 2 | 28% | 3 |
| 997 | nodal modulator 3 precursor . | 51944969 | 134 | 2 | 2.00% | 3 |
| 998 | actin-related protein 2 isoform b | 5031571 | 45 | 2 | 7.40% | 3 |
| 999 | peptidase M20 domain-containing protein 2 | 58082085 | 48 | 2 | 7.60% | 3 |
| 1000 | prostaglandin-H2 D-isomerase precursor | 32171249 | 21 | 2 | 12% | 3 |
| 1001 | annexin A13 isoform a | 51895795 | 35 | 2 | 8.20% | 3 |
| 1002 | low-density lipoprotein receptor-related protein 2 precursor | 126012573 | 522 | 2 | 0.49% | 3 |
| 1003 | 6-phosphofructokinase, liver type | 48762920 | 85 | 2 | 3.60% | 3 |
| 1004 | lanC-like protein 1 . | 212274337 | 45 | 2 | 6.30% | 3 |
| 1005 | amyloid beta A4 protein isoform d | 209862833 | 85 | 2 | 3.50% | 3 |
| 1006 | serine/threonine-protein phosphatase 2B catalytic subunit gamma isoform isoform 1 | 345842412 | 59 | 2 | 5.60% | 3 |
| 1007 | long-chain-fatty-acid--CoA ligase 3 | 42794752 | 80 | 2 | 4.20% | 3 |
| 1008 | transforming growth factor beta receptor type 3 isoform b precursor | 307574634 | 93 | 2 | 4.10% | 3 |
| 1009 | actin-related protein 2/3 complex subunit 3 | 5031597 | 21 | 2 | 13% | 3 |
| 1010 | beta-1,4-galactosyltransferase 1 | 13929462 | 44 | 2 | 6.80% | 3 |
| 1011 | 60S ribosomal protein L29 | 4506629 | 18 | 2 | 14% | 3 |
| 1012 | ubiquitin carboxyl-terminal hydrolase 7 | 150378533 | 128 | 2 | 2.70% | 2 |
| 1013 | ATP synthase subunit delta, mitochondrial precursor | 50345991 | 17 | 2 | 14% | 2 |
| 1014 | F-actin-capping protein subunit alpha-2 | 5453599 | 33 | 2 | 12% | 2 |
| 1015 | quinone oxidoreductase isoform a | 13236495 | 35 | 2 | 7.90% | 2 |
| 1016 | ras-related protein Rab-18 | 10880989 | 23 | 2 | 12% | 2 |
| 1017 | 4F2 cell-surface antigen heavy chain isoform c | 65506891 | 68 | 2 | 4.10% | 2 |
| 1018 | hsp90 co-chaperone Cdc37 | 5901922 | 44 | 2 | 11% | 2 |
| 1019 | mannosyl-oligosaccharide glucosidase isoform 1 | 149999606 | 92 | 2 | 3.60% | 2 |
| 1020 | BAG family molecular chaperone regulator 2 | 4757834 | 24 | 2 | 11% | 2 |
| 1021 | zona pellucida-binding protein 2 isoform 1 precursor | 84875535 | 36 | 2 | 7.30% | 2 |
| 1022 | proliferation-associated protein 2G4 | 124494254 | 44 | 2 | 8.60% | 2 |
| 1023 | prostate and testis expressed protein 4 | 221554530 | 11 | 2 | 23% | 2 |
| 1024 | Na(+)/H(+) exchange regulatory cofactor NHE-RF1 | 4759140 | 39 | 2 | 6.70% | 2 |
| 1025 | SPARC-related modular calcium-binding protein 1 isoform 2 precursor | 11545873 | 48 | 2 | 5.50% | 2 |
| 1026 | glutaredoxin-3 | 95113651 | 37 | 2 | 15% | 2 |
| 1027 | 60S ribosomal protein L13 isoform 1 | 15431295 | 24 | 2 | 9.00% | 2 |
| 1028 | NADPH--cytochrome P450 reductase | 127139033 | 77 | 2 | 4.90% | 2 |
| 1029 | aminoacyl tRNA synthase complex-interacting multifunctional protein 2 | 11125770 | 35 | 2 | 11% | 2 |
| 1030 | nesprin-1 isoform 1 | 330688408 | 1011 | 2 | 0.33% | 2 |
| 1031 | coiled-coil domain-containing protein 11 | 94721321 | 62 | 2 | 7.20% | 2 |
| 1032 | cadherin EGF LAG seven-pass G-type receptor 1 precursor | 7656967 | 329 | 2 | 1.00% | 2 |
| 1033 | AMY-1-associating protein expressed in testis 1 | 38569419 | 90 | 2 | 3.10% | 2 |
| 1034 | dnaJ homolog subfamily C member 5 | 45504382 | 22 | 2 | 8.60% | 2 |
| 1035 | endoribonuclease Dicer isoform 1 | 29294651 | 219 | 2 | 1.20% | 2 |
| 1036 | ATP-binding cassette sub-family B member 6, mitochondrial [Homo | 9955963 | 94 | 2 | 3.30% | 2 |
| 1037 | septin-10 isoform 1 . | 21945064 | 53 | 2 | 6.20% | 2 |
| 1038 | tetratricopeptide repeat protein 18 . | 229577238 | 126 | 2 | 2.10% | 2 |
| 1039 | peptidyl-prolyl cis-trans isomerase FKBP3 . | 4503727 | 25 | 2 | 12% | 2 |
| 1040 | prostate and testis expressed protein 3 precursor . | 222136622 | 12 | 2 | 16% | 2 |
| 1041 | calcineurin subunit B type 1 | 4506025 | 19 | 2 | 29% | 2 |
| 1042 | haloacid dehalogenase-like hydrolase domain-containing protein 2 | 14149777 | 29 | 2 | 14% | 2 |
