# Supplementary material for: Major protein alterations in spermatozoa from infertile men with unilateral varicocele
Source: Reprod Biol Endocrinol. 2015 Feb 22;13:8. doi: 10.1186/s12958-015-0007-2 (PMC4383193; doi:10.1186/s12958-015-0007-2)
Supplement: Additiona file 4: Table S4. — Global proteomic profiling of unilateral varicocele group in triplicate - first run - gel 1. [file 12958_2015_7_MOESM4_ESM.docx]

**Supplemental Table 4. Global proteomic profiling of unilateral varicocele group in triplicate - first run – gel 1.**

| **No.** | **Gel 1** | | | | | |
| --- | --- | --- | --- | --- | --- | --- |
|  | **Protein** | **Accession** | **Mass** | **Peptides** | **Sequence** | **Spectral** |
|  |  | **No.** | **kDa** | **No.** | **Cov (%)** | **Counts** |
| 1 | lactotransferrin isoform 1 precursor | 54607120 | 78 | 85 | 81% | 1593 |
| 2 | fibronectin isoform 3 preproprotein | 16933542 | 259 | 126 | 47% | 1299 |
| 3 | semenogelin-2 precursor | 4506885 | 65 | 23 | 47% | 917 |
| 4 | A-kinase anchor protein 4 isoform 2 | 21493039 | 93 | 72 | 61% | 821 |
| 5 | semenogelin-1 preproprotein | 4506883 | 52 | 41 | 62% | 771 |
| 6 | serum albumin preproprotein | 4502027 | 69 | 54 | 80% | 569 |
| 7 | keratin, type II cytoskeletal 1 | 119395750 | 66 | 36 | 55% | 481 |
| 8 | aminopeptidase N precursor | 157266300 | 110 | 48 | 35% | 409 |
| 9 | outer dense fiber protein 2 isoform 3 | 310750406 | 81 | 33 | 33% | 368 |
| 10 | keratin, type I cytoskeletal 9 | 55956899 | 62 | 32 | 58% | 358 |
| 11 | heat shock-related 70 protein 2 | 13676857 | 70 | 37 | 41% | 352 |
| 12 | alpha-enolase isoform 1 | 4503571 | 47 | 39 | 78% | 347 |
| 13 | keratin, type I cytoskeletal 10 | 195972866 | 59 | 31 | 46% | 337 |
| 14 | prostatic acid phosphatase isoform TM-PAP precursor | 197116348 | 48 | 18 | 30% | 297 |
| 15 | dipeptidyl peptidase 4 | 18765694 | 88 | 43 | 45% | 295 |
| 16 | malate dehydrogenase, mitochondrial precursor | 21735621 | 36 | 21 | 64% | 289 |
| 17 | keratin, type II cytoskeletal 2 epidermal | 47132620 | 65 | 28 | 54% | 287 |
| 18 | 78 glucose-regulated protein precursor | 16507237 | 72 | 28 | 34% | 271 |
| 19 | tubulin beta-4B chain | 5174735 | 50 | 32 | 63% | 270 |
| 20 | prolactin-inducible protein precursor | 4505821 | 17 | 15 | 77% | 265 |
| 21 | tubulin alpha-3C/D chain | 17921993 | 50 | 24 | 53% | 262 |
| 22 | A-kinase anchor protein 3 | 21493041 | 95 | 37 | 36% | 255 |
| 23 | myosin-9 | 12667788 | 227 | 54 | 18% | 255 |
| 24 | glyceraldehyde-3-phosphate dehydrogenase, testis-specific | 7657116 | 45 | 24 | 75% | 254 |
| 25 | clusterin preproprotein | 355594753 | 52 | 24 | 36% | 249 |
| 26 | triosephosphate isomerase isoform 2 | 226529917 | 31 | 25 | 86% | 246 |
| 27 | heat shock cognate 71 protein isoform 1 | 5729877 | 71 | 17 | 28% | 244 |
| 28 | dihydrolipoyl dehydrogenase, mitochondrial precursor | 91199540 | 54 | 26 | 60% | 241 |
| 29 | protein-glutamine gamma-glutamyltransferase 4 | 156627577 | 77 | 33 | 38% | 236 |
| 30 | actin, cytoplasmic 1 | 4501885 | 42 | 30 | 65% | 234 |
| 31 | angiotensin-converting enzyme isoform 1 precursor | 4503273 | 150 | 37 | 33% | 231 |
| 32 | cytosol aminopeptidase | 41393561 | 56 | 32 | 70% | 229 |
| 33 | heat shock 70 protein 1-like | 124256496 | 70 | 14 | 26% | 208 |
| 34 | pyruvate kinase isozymes M1/M2 isoform c | 332164775 | 66 | 27 | 39% | 203 |
| 35 | fatty acid synthase | 41872631 | 273 | 46 | 16% | 194 |
| 36 | aspartate aminotransferase, mitochondrial precursor | 73486658 | 48 | 32 | 69% | 190 |
| 37 | heat shock protein HSP 90-alpha isoform 1 | 153792590 | 98 | 35 | 28% | 188 |
| 38 | nuclear pore membrane glycoprotein 210-like isoform 1 precursor | 117414168 | 211 | 44 | 25% | 183 |
| 39 | filamin-B isoform 2 | 105990514 | 278 | 57 | 14% | 183 |
| 40 | heat shock 70 protein 1A/1B | 194248072 | 70 | 10 | 26% | 182 |
| 41 | ropporin-1A | 21359920 | 24 | 15 | 74% | 181 |
| 42 | peroxiredoxin-1 | 320461711 | 22 | 13 | 63% | 178 |
| 43 | endoplasmin precursor | 4507677 | 92 | 32 | 27% | 176 |
| 44 | heat shock protein HSP 90-beta | 20149594 | 83 | 18 | 29% | 176 |
| 45 | prostate-specific antigen isoform 1 preproprotein | 4502173 | 29 | 14 | 54% | 176 |
| 46 | maltase-glucoamylase, intestinal | 221316699 | 210 | 49 | 29% | 173 |
| 47 | calreticulin precursor | 4757900 | 48 | 21 | 51% | 169 |
| 48 | fructose-bisphosphate aldolase A isoform 2 | 342187211 | 45 | 21 | 49% | 161 |
| 49 | glutathione S-transferase Mu 3 | 23065552 | 27 | 15 | 55% | 152 |
| 50 | glutamate carboxypeptidase 2 isoform 1 | 4758398 | 84 | 35 | 46% | 152 |
| 51 | glyceraldehyde-3-phosphate dehydrogenase | 7669492 | 36 | 20 | 53% | 147 |
| 52 | olfactomedin-4 precursor | 32313593 | 57 | 20 | 42% | 146 |
| 53 | elongation factor 1-alpha 1 | 4503471 | 50 | 21 | 42% | 137 |
| 54 | zinc-alpha-2-glycoprotein precursor | 4502337 | 34 | 20 | 58% | 137 |
| 55 | cytosolic non-specific dipeptidase isoform 1 | 271398239 | 53 | 19 | 41% | 135 |
| 56 | glucose-6-phosphate isomerase isoform 2 | 18201905 | 63 | 26 | 54% | 133 |
| 57 | nitric oxide synthase, endothelial isoform 1 | 40254422 | 133 | 10 | 11% | 131 |
| 58 | keratin, type I cytoskeletal 14 | 15431310 | 52 | 11 | 41% | 126 |
| 59 | phosphoglycerate kinase 2 | 31543397 | 45 | 16 | 44% | 125 |
| 60 | tubulin alpha-1C chain | 14389309 | 50 | 2 | 42% | 122 |
| 61 | plastin-2 | 167614506 | 70 | 26 | 39% | 122 |
| 62 | laminin subunit alpha-5 precursor | 21264602 | 400 | 38 | 12% | 120 |
| 63 | peroxiredoxin-4 precursor | 5453549 | 31 | 15 | 66% | 120 |
| 64 | succinate dehydrogenase [ubiquinone] flavoprotein subunit, mitochondrial | 156416003 | 73 | 29 | 61% | 119 |
| 65 | serpin B6 | 41152086 | 43 | 23 | 72% | 118 |
| 66 | sorbitol dehydrogenase | 156627571 | 38 | 16 | 54% | 117 |
| 67 | keratin, type II cytoskeletal 5 | 119395754 | 62 | 11 | 20% | 114 |
| 68 | L-lactate dehydrogenase C chain | 4504973 | 36 | 11 | 30% | 114 |
| 69 | very long-chain specific acyl-CoA dehydrogenase, mitochondrial isoform 2 precursor | 76496475 | 68 | 36 | 66% | 110 |
| 70 | sperm protein associated with the nucleus on the X chromosome B/F | 22027492 | 12 | 7 | 81% | 109 |
| 71 | 4-trimethylaminobutyraldehyde dehydrogenase | 115387104 | 56 | 16 | 31% | 109 |
| 72 | sperm acrosome membrane-associated protein 1 precursor | 13569934 | 32 | 14 | 38% | 108 |
| 73 | dihydrolipoyllysine-residue acetyltransferase component of pyruvate dehydrogenase complex, mitochondrial precursor | 31711992 | 69 | 14 | 19% | 107 |
| 74 | ropporin-1B | 59891409 | 24 | 4 | 80% | 106 |
| 75 | elongation factor 2 | 4503483 | 95 | 19 | 22% | 105 |
| 76 | ATP synthase subunit alpha, mitochondrial precursor | 4757810 | 60 | 19 | 32% | 103 |
| 77 | histone H2B type 1-A | 24586679 | 14 | 6 | 41% | 103 |
| 78 | keratin, type I cytoskeletal 16 | 24430192 | 51 | 10 | 46% | 103 |
| 79 | phospholipid hydroperoxide glutathione peroxidase, mitochondrial isoform A precursor | 75709200 | 22 | 14 | 54% | 101 |
| 80 | clathrin heavy chain 1 | 4758012 | 192 | 28 | 12% | 101 |
| 81 | galectin-3-binding protein precursor | 5031863 | 65 | 16 | 28% | 101 |
| 82 | ferritin, mitochondrial precursor | 29126241 | 28 | 15 | 56% | 100 |
| 83 | medium-chain specific acyl-CoA dehydrogenase, mitochondrial isoform b precursor | 187960098 | 47 | 21 | 46% | 98 |
| 84 | histone H4 | 4504303 | 11 | 10 | 51% | 97 |
| 85 | ATP synthase subunit beta, mitochondrial precursor | 32189394 | 57 | 20 | 47% | 96 |
| 86 | calmodulin | 58218968 | 17 | 11 | 56% | 93 |
| 87 | heat shock protein beta-1 | 4504517 | 23 | 12 | 42% | 91 |
| 88 | serum amyloid P-component precursor | 4502133 | 25 | 10 | 34% | 91 |
| 89 | ubiquitin-40S ribosomal protein S27a precursor | 208022622 | 18 | 5 | 30% | 90 |
| 90 | glutathione reductase, mitochondrial isoform 1 precursor | 50301238 | 56 | 22 | 62% | 90 |
| 91 | T-complex protein 1 subunit gamma isoform a | 63162572 | 61 | 21 | 27% | 89 |
| 92 | leucine-rich repeat-containing protein 37B precursor | 53829385 | 106 | 16 | 15% | 85 |
| 93 | adipocyte plasma membrane-associated protein | 24308201 | 46 | 19 | 46% | 85 |
| 94 | aspartate aminotransferase, cytoplasmic | 4504067 | 46 | 22 | 65% | 85 |
| 95 | hexokinase-1 isoform HKI-ta/tb | 15991831 | 103 | 20 | 14% | 84 |
| 96 | fumarate hydratase, mitochondrial | 19743875 | 55 | 16 | 37% | 83 |
| 97 | T-complex protein 1 subunit beta isoform 1 | 5453603 | 57 | 18 | 34% | 83 |
| 98 | importin-5 | 24797086 | 126 | 22 | 16% | 83 |
| 99 | trifunctional enzyme subunit alpha, mitochondrial precursor | 20127408 | 83 | 21 | 22% | 82 |
| 100 | vesicular integral-membrane protein VIP36 precursor | 5803023 | 40 | 15 | 50% | 82 |
| 101 | transitional endoplasmic reticulum ATPase | 6005942 | 89 | 20 | 22% | 81 |
| 102 | T-complex protein 1 subunit epsilon | 24307939 | 60 | 17 | 29% | 81 |
| 103 | proteasome subunit alpha type-1 isoform 1 | 23110935 | 30 | 19 | 52% | 81 |
| 104 | creatine kinase B-type | 21536286 | 43 | 15 | 39% | 80 |
| 105 | L-asparaginase | 145275200 | 32 | 13 | 61% | 80 |
| 106 | agrin precursor | 54873613 | 215 | 24 | 13% | 80 |
| 107 | adenosylhomocysteinase isoform 1 | 9951915 | 48 | 19 | 44% | 79 |
| 108 | serotransferrin precursor | 4557871 | 77 | 22 | 35% | 78 |
| 109 | keratin, type II cytoskeletal 6A | 5031839 | 60 | 6 | 16% | 76 |
| 110 | tektin-3 | 13994250 | 57 | 14 | 30% | 76 |
| 111 | neprilysin | 116256329 | 86 | 19 | 20% | 76 |
| 112 | proteasome subunit alpha type-6 | 23110944 | 27 | 15 | 55% | 76 |
| 113 | 60 heat shock protein, mitochondrial | 31542947 | 61 | 15 | 22% | 75 |
| 114 | sperm protein associated with the nucleus on the X chromosome C | 13435137 | 11 | 9 | 57% | 75 |
| 115 | cysteine-rich secretory protein 1 isoform 1 precursor | 327315372 | 28 | 8 | 39% | 74 |
| 116 | phosphoglycerate kinase 1 | 4505763 | 45 | 14 | 38% | 72 |
| 117 | uromodulin precursor | 59850812 | 70 | 12 | 19% | 72 |
| 118 | proteasome subunit beta type-1 | 4506193 | 26 | 14 | 54% | 72 |
| 119 | ras-related protein Rab-2A isoform a | 4506365 | 24 | 14 | 50% | 71 |
| 120 | T-complex protein 1 subunit delta | 38455427 | 58 | 14 | 28% | 71 |
| 121 | tubulin alpha-1A chain | 17986283 | 50 | 2 | 37% | 70 |
| 122 | peroxiredoxin-2 isoform a | 32189392 | 22 | 10 | 42% | 70 |
| 123 | superoxide dismutase [Mn], mitochondrial isoform A precursor | 67782305 | 25 | 12 | 58% | 70 |
| 124 | annexin A5 | 4502107 | 36 | 16 | 51% | 69 |
| 125 | tubulin beta-6 chain | 14210536 | 50 | 3 | 12% | 69 |
| 126 | single-stranded DNA-binding protein, mitochondrial precursor | 4507231 | 17 | 11 | 71% | 69 |
| 127 | T-complex protein 1 subunit theta | 48762932 | 60 | 12 | 22% | 68 |
| 128 | laminin subunit beta-2 precursor | 119703755 | 196 | 24 | 12% | 68 |
| 129 | dipeptidase 3 isoform a precursor | 193211608 | 56 | 9 | 18% | 67 |
| 130 | 14-3-3 protein epsilon | 5803225 | 29 | 14 | 55% | 66 |
| 131 | WD repeat-containing protein 16 isoform b | 124028512 | 68 | 22 | 43% | 66 |
| 132 | protein disulfide-isomerase A3 precursor | 21361657 | 57 | 13 | 25% | 64 |
| 133 | long-chain-fatty-acid--CoA ligase 1 | 40807491 | 78 | 13 | 12% | 64 |
| 134 | 5-oxoprolinase | 48314820 | 137 | 19 | 14% | 64 |
| 135 | D-3-phosphoglycerate dehydrogenase | 23308577 | 57 | 13 | 30% | 63 |
| 136 | annexin A6 isoform 1 | 71773329 | 76 | 16 | 26% | 63 |
| 137 | carbonic anhydrase 4 precursor | 4502519 | 35 | 11 | 38% | 62 |
| 138 | peroxiredoxin-6 | 4758638 | 25 | 8 | 40% | 61 |
| 139 | proteasome subunit alpha type-7-like isoform 2 | 68303563 | 28 | 13 | 65% | 61 |
| 140 | proteasome subunit alpha type-2 | 4506181 | 26 | 13 | 57% | 61 |
| 141 | glucosidase 2 subunit beta isoform 1 precursor | 48255889 | 59 | 11 | 16% | 61 |
| 142 | T-complex protein 1 subunit alpha isoform a | 57863257 | 60 | 15 | 26% | 60 |
| 143 | proteasome subunit beta type-3 | 22538465 | 23 | 11 | 48% | 60 |
| 144 | proteasome subunit beta type-5 isoform 1 | 4506201 | 28 | 14 | 48% | 59 |
| 145 | proteasome subunit alpha type-3 isoform 2 | 23110939 | 28 | 12 | 46% | 59 |
| 146 | T-complex protein 1 subunit eta isoform a | 5453607 | 59 | 13 | 21% | 58 |
| 147 | protein disulfide-isomerase precursor | 20070125 | 57 | 16 | 20% | 57 |
| 148 | plasma serine protease inhibitor precursor | 194018472 | 46 | 17 | 45% | 57 |
| 149 | cytoplasmic dynein 1 heavy chain 1 | 33350932 | 532 | 30 | 4.60% | 57 |
| 150 | elongation factor 1-gamma | 4503481 | 50 | 10 | 16% | 56 |
| 151 | ectonucleotide pyrophosphatase/phosphodiesterase family member 3 | 111160296 | 100 | 21 | 34% | 56 |
| 152 | pyruvate kinase isozymes M1/M2 isoform a | 33286418 | 58 | 2 | 42% | 55 |
| 153 | annexin A1 | 4502101 | 39 | 12 | 39% | 54 |
| 154 | dynein light chain 2, cytoplasmic | 18087855 | 10 | 5 | 65% | 54 |
| 155 | myeloperoxidase precursor | 4557759 | 84 | 18 | 32% | 54 |
| 156 | cullin-associated NEDD8-dissociated protein 1 | 21361794 | 136 | 15 | 11% | 53 |
| 157 | ras GTPase-activating-like protein IQGAP1 | 4506787 | 189 | 13 | 8.20% | 53 |
| 158 | ruvB-like 1 | 4506753 | 50 | 12 | 22% | 52 |
| 159 | tektin-2 | 16507950 | 50 | 13 | 28% | 52 |
| 160 | mucin-6 precursor | 151301154 | 257 | 12 | 4.80% | 52 |
| 161 | G-protein coupled receptor 64 isoform 2 precursor | 119943116 | 110 | 12 | 14% | 52 |
| 162 | aspartyl aminopeptidase | 156416028 | 53 | 17 | 45% | 52 |
| 163 | electron transfer flavoprotein subunit alpha, mitochondrial isoform a | 4503607 | 35 | 12 | 41% | 51 |
| 164 | transketolase | 205277463 | 68 | 21 | 43% | 51 |
| 165 | thioredoxin reductase 2, mitochondrial precursor | 22035672 | 57 | 16 | 42% | 51 |
| 166 | catalase . | 4557014 | 60 | 19 | 44% | 51 |
| 167 | keratin, type I cytoskeletal 13 isoform a | 131412225 | 50 | 8 | 12% | 50 |
| 168 | izumo sperm-egg fusion protein 4 isoform 1 precursor | 89903025 | 24 | 4 | 29% | 50 |
| 169 | ras-related protein Rab-11B | 190358517 | 24 | 10 | 48% | 50 |
| 170 | brain acid soluble protein 1 | 30795231 | 23 | 12 | 81% | 50 |
| 171 | aconitate hydratase, mitochondrial precursor | 4501867 | 85 | 14 | 13% | 49 |
| 172 | tektin-4 | 21389613 | 51 | 12 | 28% | 49 |
| 173 | CD177 antigen precursor | 110735433 | 46 | 10 | 42% | 49 |
| 174 | leucine-rich repeat-containing protein 37A precursor | 289547512 | 188 | 9 | 4.60% | 48 |
| 175 | cytochrome c oxidase subunit 4 isoform 1, mitochondrial precursor | 4502981 | 20 | 10 | 47% | 48 |
| 176 | laminin subunit gamma-1 precursor | 145309326 | 178 | 16 | 13% | 48 |
| 177 | cathelicidin antimicrobial peptide preproprotein | 348041314 | 20 | 7 | 28% | 47 |
| 178 | isocitrate dehydrogenase [NADP] cytoplasmic | 28178825 | 47 | 13 | 21% | 47 |
| 179 | phosphatidylethanolamine-binding protein 1 preproprotein | 4505621 | 21 | 11 | 64% | 47 |
| 180 | 10 heat shock protein, mitochondrial | 4504523 | 11 | 10 | 74% | 47 |
| 181 | protein NipSnap homolog 3A | 22267436 | 28 | 12 | 60% | 46 |
| 182 | dynein light chain 1, cytoplasmic | 4505813 | 10 | 8 | 65% | 45 |
| 183 | 2,4-dienoyl-CoA reductase, mitochondrial precursor | 4503301 | 36 | 9 | 29% | 45 |
| 184 | acrosomal protein SP-10 isoform a precursor | 4501879 | 28 | 6 | 22% | 45 |
| 185 | dihydrolipoyllysine-residue succinyltransferase component of 2-oxoglutarate dehydrogenase complex, mitochondrial isoform 1 precursor | 19923748 | 49 | 9 | 29% | 45 |
| 186 | 14-3-3 protein zeta/delta | 4507953 | 28 | 7 | 35% | 44 |
| 187 | ADP/ATP translocase 4 | 13775208 | 35 | 11 | 30% | 44 |
| 188 | proteasome subunit alpha type-5 isoform 1 | 23110942 | 26 | 10 | 55% | 44 |
| 189 | mesencephalic astrocyte-derived neurotrophic factor precursor | 299523086 | 21 | 9 | 41% | 44 |
| 190 | tektin-5 | 21389569 | 56 | 10 | 19% | 44 |
| 191 | sperm equatorial segment protein 1 precursor | 21717832 | 39 | 7 | 15% | 43 |
| 192 | voltage-dependent anion-selective channel protein 3 isoform 1 | 25188179 | 31 | 5 | 19% | 43 |
| 193 | proteasome subunit alpha type-7 | 4506189 | 28 | 7 | 51% | 43 |
| 194 | lactoylglutathione lyase | 118402586 | 21 | 8 | 35% | 43 |
| 195 | proteasome subunit alpha type-4 isoform 1 | 4506185 | 29 | 13 | 56% | 43 |
| 196 | protein S100-A9 | 4506773 | 13 | 7 | 53% | 43 |
| 197 | acylamino-acid-releasing enzyme | 23510451 | 81 | 11 | 15% | 43 |
| 198 | hypoxanthine-guanine phosphoribosyltransferase | 4504483 | 25 | 11 | 57% | 42 |
| 199 | alpha-1-antitrypsin precursor | 50363217 | 47 | 15 | 39% | 42 |
| 200 | inositol monophosphatase 1 isoform 2 | 221625487 | 37 | 13 | 40% | 42 |
| 201 | ruvB-like 2 | 5730023 | 51 | 10 | 19% | 41 |
| 202 | ecto-ADP-ribosyltransferase 3 isoform a precursor | 194097380 | 44 | 8 | 21% | 41 |
| 203 | purine nucleoside phosphorylase | 157168362 | 32 | 11 | 45% | 41 |
| 204 | acid ceramidase isoform b | 189011546 | 47 | 13 | 18% | 41 |
| 205 | keratin, type II cytoskeletal 6B | 119703753 | 60 | 2 | 20% | 40 |
| 206 | glycerol kinase 2 | 41393575 | 61 | 10 | 15% | 40 |
| 207 | UPF0577 protein KIAA1324 precursor | 38569482 | 111 | 11 | 9.70% | 40 |
| 208 | platelet-activating factor acetylhydrolase precursor | 270133071 | 50 | 17 | 50% | 40 |
| 209 | zona pellucida-binding protein 1 isoform 1 precursor | 229577313 | 40 | 9 | 17% | 39 |
| 210 | protein NDRG1 | 37655183 | 43 | 5 | 21% | 39 |
| 211 | proteasome subunit beta type-6 | 23110925 | 25 | 6 | 31% | 39 |
| 212 | histone H2A type 1-A | 25092737 | 14 | 6 | 35% | 39 |
| 213 | calpain-1 catalytic subunit | 311893363 | 82 | 15 | 29% | 39 |
| 214 | dolichyl-diphosphooligosaccharide--protein glycosyltransferase subunit 1 precursor | 4506675 | 69 | 11 | 12% | 38 |
| 215 | importin subunit beta-1 | 19923142 | 97 | 11 | 11% | 38 |
| 216 | epididymal secretory protein E1 precursor | 5453678 | 17 | 10 | 46% | 38 |
| 217 | superoxide dismutase [Cu-Zn] | 4507149 | 16 | 6 | 57% | 38 |
| 218 | glutathione synthetase | 4504169 | 52 | 15 | 37% | 38 |
| 219 | epididymal sperm-binding protein 1 precursor | 301601648 | 26 | 7 | 29% | 37 |
| 220 | lysosome-associated membrane glycoprotein 1 precursor | 112380628 | 45 | 8 | 13% | 37 |
| 221 | protein S100-A8 | 21614544 | 11 | 8 | 53% | 37 |
| 222 | proteasome subunit beta type-2 isoform 1 | 4506195 | 23 | 11 | 38% | 37 |
| 223 | complement component 1 Q subcomponent-binding protein, mitochondrial precursor | 4502491 | 31 | 6 | 29% | 37 |
| 224 | poly(rC)-binding protein 1 | 222352151 | 37 | 7 | 23% | 37 |
| 225 | homogentisate 1,2-dioxygenase | 115527117 | 50 | 12 | 41% | 37 |
| 226 | carnitine O-palmitoyltransferase 2, mitochondrial precursor | 4503023 | 74 | 11 | 19% | 36 |
| 227 | electron transfer flavoprotein subunit beta isoform 1 | 4503609 | 28 | 10 | 38% | 36 |
| 228 | nicastrin precursor | 24638433 | 78 | 9 | 15% | 36 |
| 229 | stress-70 protein, mitochondrial precursor | 24234688 | 74 | 9 | 11% | 35 |
| 230 | cytochrome c oxidase subunit 5A, mitochondrial precursor | 190885499 | 17 | 6 | 31% | 35 |
| 231 | peptidyl-prolyl cis-trans isomerase B precursor | 4758950 | 24 | 7 | 37% | 35 |
| 232 | proteasome subunit beta type-7 proprotein | 4506203 | 30 | 8 | 21% | 35 |
| 233 | annexin A2 isoform 1 | 50845388 | 40 | 10 | 22% | 34 |
| 234 | succinyl-CoA:3-ketoacid-coenzyme A transferase 1, mitochondrial precursor | 4557817 | 56 | 9 | 24% | 34 |
| 235 | protein DJ-1 | 31543380 | 20 | 7 | 44% | 34 |
| 236 | 26S proteasome non-ATPase regulatory subunit 11 | 28872725 | 47 | 6 | 18% | 34 |
| 237 | 6-phosphogluconate dehydrogenase, decarboxylating | 40068518 | 53 | 10 | 21% | 34 |
| 238 | glucosamine--fructose-6-phosphate aminotransferase [isomerizing] 1 isoform 1 | 347659028 | 79 | 11 | 12% | 34 |
| 239 | calcium-binding tyrosine phosphorylation-regulated protein isoform a | 24797108 | 53 | 4 | 22% | 33 |
| 240 | cytochrome c oxidase subunit II | 251831110 | 26 | 6 | 26% | 33 |
| 241 | 14-3-3 protein theta | 5803227 | 28 | 8 | 29% | 33 |
| 242 | hyaluronidase PH-20 isoform 2 | 291290981 | 58 | 7 | 9.20% | 33 |
| 243 | nucleobindin-2 precursor | 4826870 | 50 | 10 | 14% | 33 |
| 244 | ATP-citrate synthase isoform 1 | 38569421 | 121 | 11 | 10% | 33 |
| 245 | cytochrome b-c1 complex subunit 2, mitochondrial precursor | 50592988 | 48 | 7 | 20% | 32 |
| 246 | voltage-dependent anion-selective channel protein 2 isoform 2 | 296317339 | 32 | 7 | 28% | 32 |
| 247 | sperm acrosome membrane-associated protein 3 | 27777653 | 23 | 8 | 33% | 32 |
| 248 | ribonuclease inhibitor | 42822872 | 50 | 10 | 22% | 32 |
| 249 | CDGSH iron-sulfur domain-containing protein 1 | 8923930 | 12 | 6 | 49% | 32 |
| 250 | selenium-binding protein 1 | 16306550 | 52 | 12 | 29% | 32 |
| 251 | xaa-Pro dipeptidase isoform 1 | 149589008 | 55 | 12 | 31% | 32 |
| 252 | ATP synthase subunit d, mitochondrial isoform a | 5453559 | 18 | 6 | 39% | 31 |
| 253 | glypican-4 precursor | 21614525 | 62 | 7 | 20% | 31 |
| 254 | testis-expressed protein 101 isoform 1 | 194018544 | 29 | 4 | 18% | 31 |
| 255 | 3-ketoacyl-CoA thiolase, mitochondrial | 167614485 | 42 | 13 | 47% | 31 |
| 256 | fructose-1,6-bisphosphatase 1 | 189083692 | 37 | 10 | 45% | 31 |
| 257 | deoxyguanosine kinase, mitochondrial isoform a precursor | 18426967 | 32 | 6 | 31% | 31 |
| 258 | uncharacterized protein C1orf56 precursor | 20149646 | 37 | 5 | 21% | 30 |
| 259 | erlin-2 isoform 1 | 6005721 | 38 | 9 | 21% | 30 |
| 260 | peptidyl-prolyl cis-trans isomerase A | 10863927 | 18 | 8 | 47% | 30 |
| 261 | chloride intracellular channel protein 1 | 14251209 | 27 | 6 | 32% | 30 |
| 262 | prostasin preproprotein | 4506153 | 36 | 5 | 22% | 30 |
| 263 | glycerol-3-phosphate dehydrogenase 1-like protein | 24307999 | 38 | 9 | 25% | 30 |
| 264 | alpha-aminoadipic semialdehyde dehydrogenase isoform 2 | 319655561 | 55 | 11 | 37% | 30 |
| 265 | hypoxia up-regulated protein 1 precursor | 5453832 | 111 | 7 | 5.30% | 29 |
| 266 | histone H3.1 | 4504281 | 15 | 5 | 38% | 29 |
| 267 | L-xylulose reductase isoform 1 | 7705925 | 26 | 5 | 23% | 29 |
| 268 | proteasome subunit beta type-4 | 22538467 | 29 | 7 | 39% | 29 |
| 269 | beta-lactamase-like protein 2 | 7705793 | 33 | 11 | 53% | 29 |
| 270 | calcium-binding tyrosine phosphorylation-regulated protein isoform c | 24797112 | 41 | 7 | 14% | 28 |
| 271 | saccharopine dehydrogenase-like oxidoreductase | 55770836 | 47 | 7 | 19% | 28 |
| 272 | sperm protein associated with the nucleus on the X chromosome E | 22027496 | 11 | 2 | 56% | 28 |
| 273 | calnexin precursor | 10716563 | 68 | 10 | 14% | 28 |
| 274 | ferritin heavy chain | 56682959 | 21 | 10 | 58% | 28 |
| 275 | serine/threonine-protein phosphatase 2A catalytic subunit alpha isoform | 4506017 | 36 | 8 | 38% | 28 |
| 276 | leukocyte surface antigen CD47 isoform 1 precursor | 4502673 | 35 | 3 | 8.70% | 28 |
| 277 | proactivator polypeptide isoform a preproprotein | 11386147 | 58 | 8 | 12% | 28 |
| 278 | peptidase M20 domain-containing protein 2 | 58082085 | 48 | 9 | 27% | 28 |
| 279 | beta-microseminoprotein isoform a precursor | 4557036 | 13 | 3 | 18% | 28 |
| 280 | fatty acid-binding protein, epidermal | 4557581 | 15 | 9 | 64% | 28 |
| 281 | N(G),N(G)-dimethylarginine dimethylaminohydrolase 1 isoform 1 | 6912328 | 31 | 8 | 44% | 28 |
| 282 | delta-aminolevulinic acid dehydratase | 189083849 | 36 | 9 | 35% | 28 |
| 283 | acetyl-CoA acetyltransferase, mitochondrial precursor | 4557237 | 45 | 8 | 24% | 27 |
| 284 | carnitine O-acetyltransferase precursor | 21618331 | 71 | 10 | 21% | 27 |
| 285 | peptidyl-prolyl cis-trans isomerase FKBP4 | 4503729 | 52 | 10 | 19% | 27 |
| 286 | PITH domain-containing protein 1 | 21361837 | 24 | 6 | 32% | 27 |
| 287 | serine/threonine-protein phosphatase 5 isoform 1 | 5453958 | 57 | 10 | 25% | 27 |
| 288 | cofilin-1 | 5031635 | 19 | 9 | 55% | 26 |
| 289 | hydroxyacylglutathione hydrolase, mitochondrial isoform 1 precursor | 94538322 | 34 | 10 | 39% | 26 |
| 290 | zymogen granule protein 16 homolog B precursor | 94536866 | 23 | 3 | 22% | 26 |
| 291 | CD59 glycoprotein preproprotein | 187828910 | 14 | 5 | 26% | 26 |
| 292 | EGF-like repeat and discoidin I-like domain-containing protein 3 precursor | 31317224 | 54 | 10 | 24% | 26 |
| 293 | UPF0556 protein C19orf10 precursor | 33457348 | 19 | 4 | 27% | 26 |
| 294 | glucosamine-6-phosphate isomerase 1 | 13027378 | 33 | 10 | 44% | 26 |
| 295 | tropomyosin alpha-4 chain isoform 1 | 223555975 | 33 | 11 | 41% | 26 |
| 296 | 60S ribosomal protein L5 | 14591909 | 34 | 8 | 34% | 26 |
| 297 | glycerol-3-phosphate dehydrogenase, mitochondrial precursor | 285002233 | 81 | 10 | 22% | 26 |
| 298 | pyruvate dehydrogenase E1 component subunit beta, mitochondrial isoform 1 precursor | 156564403 | 39 | 7 | 19% | 25 |
| 299 | myosin light polypeptide 6 isoform 2 | 88999583 | 17 | 4 | 31% | 25 |
| 300 | mitochondrial carrier homolog 2 | 7657347 | 33 | 7 | 30% | 25 |
| 301 | adenylyl cyclase-associated protein 1 | 5453595 | 52 | 10 | 19% | 25 |
| 302 | proteasome activator complex subunit 1 isoform 1 | 5453990 | 29 | 6 | 17% | 25 |
| 303 | copper homeostasis protein cutC homolog | 148596990 | 29 | 8 | 41% | 25 |
| 304 | ADP/ATP translocase 2 | 156071459 | 33 | 4 | 16% | 25 |
| 305 | complement decay-accelerating factor isoform 2 precursor | 168693643 | 49 | 8 | 26% | 25 |
| 306 | basement membrane-specific heparan sulfate proteoglycan core protein precursor | 126012571 | 469 | 14 | 4.10% | 25 |
| 307 | cytochrome b-c1 complex subunit 1, mitochondrial precursor | 46593007 | 53 | 6 | 18% | 24 |
| 308 | NADH-cytochrome b5 reductase 2 | 47778923 | 31 | 10 | 47% | 24 |
| 309 | dynein heavy chain 8, axonemal | 332688227 | 539 | 8 | 1.50% | 24 |
| 310 | integrin beta-2 precursor | 89191865 | 85 | 9 | 11% | 24 |
| 311 | alpha-1-acid glycoprotein 1 precursor | 167857790 | 24 | 7 | 34% | 24 |
| 312 | ester hydrolase C11orf54 | 21361495 | 29 | 7 | 38% | 24 |
| 313 | nuclear pore membrane glycoprotein 210 precursor | 27477134 | 205 | 9 | 5.10% | 23 |
| 314 | L-lactate dehydrogenase A-like 6B | 15082234 | 42 | 5 | 15% | 23 |
| 315 | alcohol dehydrogenase [NADP+] | 24497577 | 37 | 8 | 17% | 23 |
| 316 | sodium/potassium-transporting ATPase subunit beta-3 | 4502281 | 32 | 6 | 19% | 23 |
| 317 | 60S acidic ribosomal protein P2 | 4506671 | 12 | 4 | 70% | 23 |
| 318 | general vesicular transport factor p115 | 4505541 | 108 | 7 | 4.30% | 23 |
| 319 | serine/threonine-protein phosphatase with EF-hands 1 isoform 1b | 23312374 | 73 | 10 | 11% | 23 |
| 320 | filaggrin | 60097902 | 435 | 9 | 2.60% | 23 |
| 321 | aldehyde oxidase | 71773480 | 148 | 13 | 10% | 23 |
| 322 | fumarylacetoacetase . | 4557587 | 46 | 10 | 33% | 23 |
| 323 | cAMP-dependent protein kinase type II-alpha regulatory subunit | 4758958 | 46 | 8 | 19% | 22 |
| 324 | 6-phosphofructokinase type C isoform 1 | 11321601 | 86 | 9 | 7.70% | 22 |
| 325 | protein disulfide-isomerase A6 precursor | 5031973 | 48 | 6 | 9.30% | 22 |
| 326 | hsc70-interacting protein | 19923193 | 41 | 5 | 14% | 22 |
| 327 | profilin-1 | 4826898 | 15 | 6 | 36% | 22 |
| 328 | ubiquitin-like modifier-activating enzyme 1 | 23510340 | 118 | 7 | 6.80% | 22 |
| 329 | enoyl-CoA delta isomerase 1, mitochondrial isoform 2 precursor | 295842266 | 31 | 6 | 14% | 22 |
| 330 | alpha-mannosidase 2C1 | 46852164 | 116 | 10 | 12% | 22 |
| 331 | alcohol dehydrogenase class-3 | 71565154 | 40 | 7 | 18% | 22 |
| 332 | membrane cofactor protein isoform 4 precursor | 24432108 | 43 | 5 | 8.30% | 22 |
| 333 | 3'(2'),5'-bisphosphate nucleotidase 1 . | 116812595 | 33 | 5 | 19% | 22 |
| 334 | acrosin-binding protein precursor | 17999524 | 61 | 6 | 13% | 21 |
| 335 | cAMP-dependent protein kinase type I-alpha regulatory subunit | 4506063 | 43 | 7 | 25% | 21 |
| 336 | valyl-tRNA synthetase | 5454158 | 140 | 8 | 5.50% | 21 |
| 337 | cytochrome c oxidase subunit 5B, mitochondrial precursor | 17017988 | 14 | 8 | 43% | 21 |
| 338 | alpha-actinin-4 | 12025678 | 105 | 6 | 7.00% | 21 |
| 339 | glutamine synthetase | 19923206 | 42 | 7 | 21% | 21 |
| 340 | ras-related protein Rab-27B | 5729997 | 25 | 5 | 20% | 21 |
| 341 | nitrilase homolog 1 isoform 3 | 297632348 | 34 | 10 | 33% | 21 |
| 342 | ferritin light chain | 20149498 | 20 | 6 | 43% | 21 |
| 343 | copine-3 . | 4503015 | 60 | 4 | 7.80% | 21 |
| 344 | ezrin | 21614499 | 69 | 7 | 6.00% | 20 |
| 345 | transmembrane emp24 domain-containing protein 10 precursor | 98986464 | 25 | 4 | 17% | 20 |
| 346 | protein dpy-19 homolog 2 | 93277105 | 87 | 5 | 4.40% | 20 |
| 347 | barrier-to-autointegration factor | 4502389 | 10 | 6 | 64% | 20 |
| 348 | prenylcysteine oxidase 1 precursor | 166795301 | 57 | 7 | 17% | 20 |
| 349 | nucleophosmin isoform 1 | 10835063 | 33 | 5 | 19% | 20 |
| 350 | glucosamine-6-phosphate isomerase 2 | 19923881 | 31 | 2 | 32% | 20 |
| 351 | peroxiredoxin-5, mitochondrial isoform a precursor | 6912238 | 22 | 5 | 26% | 19 |
| 352 | gastricsin isoform 1 preproprotein | 4505757 | 42 | 3 | 7.50% | 19 |
| 353 | ras-related protein Rab-2B isoform 1 | 21361884 | 24 | 2 | 36% | 19 |
| 354 | malectin precursor | 7661948 | 32 | 8 | 32% | 19 |
| 355 | carboxypeptidase E preproprotein | 4503009 | 53 | 5 | 9.90% | 19 |
| 356 | transmembrane emp24 domain-containing protein 7 precursor | 32996709 | 25 | 5 | 22% | 19 |
| 357 | dynein heavy chain 17, axonemal | 256542310 | 509 | 10 | 2.40% | 19 |
| 358 | calmegin precursor | 4758004 | 70 | 8 | 22% | 19 |
| 359 | programmed cell death 6-interacting protein isoform 1 | 22027538 | 96 | 6 | 6.20% | 19 |
| 360 | lysosome-associated membrane glycoprotein 2 isoform C precursor | 169790833 | 45 | 4 | 9.20% | 19 |
| 361 | D-dopachrome decarboxylase | 4503291 | 13 | 6 | 49% | 19 |
| 362 | fibrous sheath-interacting protein 2 | 297206791 | 790 | 9 | 0.99% | 19 |
| 363 | prostate stem cell antigen preproprotein | 289547757 | 12 | 3 | 25% | 19 |
| 364 | receptor-type tyrosine-protein phosphatase eta isoform 1 precursor | 148728162 | 146 | 7 | 8.20% | 19 |
| 365 | branched-chain-amino-acid aminotransferase, mitochondrial isoform a | 50658084 | 44 | 8 | 32% | 19 |
| 366 | axonemal dynein light intermediate polypeptide 1 | 37595560 | 32 | 4 | 15% | 18 |
| 367 | 26S protease regulatory subunit 8 isoform 1 | 24497435 | 46 | 5 | 15% | 18 |
| 368 | mitochondria-eating protein | 21687119 | 61 | 5 | 8.00% | 18 |
| 369 | 26S protease regulatory subunit 10B | 195539395 | 46 | 5 | 13% | 18 |
| 370 | kunitz-type protease inhibitor 3 precursor | 189571689 | 10 | 2 | 29% | 18 |
| 371 | apolipoprotein D precursor | 4502163 | 21 | 7 | 35% | 18 |
| 372 | succinyl-CoA ligase [ADP-forming] subunit beta, mitochondrial precursor | 11321583 | 50 | 5 | 13% | 18 |
| 373 | annexin A3 | 4826643 | 36 | 6 | 17% | 17 |
| 374 | vesicle-associated membrane protein-associated protein A isoform 2 | 94721252 | 28 | 4 | 24% | 17 |
| 375 | ras-related protein Rab-14 | 19923483 | 24 | 6 | 29% | 17 |
| 376 | actin-related protein T2 | 29893808 | 42 | 5 | 14% | 17 |
| 377 | tektin-1 | 16753231 | 48 | 6 | 16% | 17 |
| 378 | FUN14 domain-containing protein 2 | 24371248 | 21 | 3 | 12% | 17 |
| 379 | aldose reductase | 4502049 | 36 | 7 | 13% | 17 |
| 380 | tomoregulin-2 precursor | 12383051 | 41 | 6 | 14% | 17 |
| 381 | GLIPR1-like protein 1 precursor | 22749527 | 26 | 5 | 16% | 17 |
| 382 | ras-related protein Ral-A precursor | 33946329 | 24 | 5 | 23% | 17 |
| 383 | fumarylacetoacetate hydrolase domain-containing protein 2B | 40786394 | 35 | 6 | 34% | 17 |
| 384 | ras-related protein Rab-7a | 34147513 | 23 | 6 | 35% | 17 |
| 385 | dipeptidase 1 precursor | 4758190 | 46 | 7 | 25% | 17 |
| 386 | serine/threonine-protein phosphatase 2B catalytic subunit alpha isoform isoform 2 | 194688147 | 58 | 6 | 13% | 17 |
| 387 | beta-glucuronidase isoform 1 precursor . | 268834192 | 75 | 4 | 8.00% | 17 |
| 388 | thioredoxin reductase 1, cytoplasmic isoform 3 | 148277071 | 71 | 7 | 16% | 17 |
| 389 | citrate synthase, mitochondrial precursor | 38327625 | 52 | 8 | 26% | 16 |
| 390 | beta-2-microglobulin precursor | 4757826 | 14 | 3 | 19% | 16 |
| 391 | cytochrome c1, heme protein, mitochondrial | 21359867 | 35 | 4 | 14% | 16 |
| 392 | alanyl-tRNA editing protein Aarsd1 isoform 1 | 217416402 | 66 | 5 | 10% | 16 |
| 393 | kelch-like protein 10 | 148664209 | 69 | 4 | 7.90% | 16 |
| 394 | cathepsin D preproprotein | 4503143 | 45 | 7 | 23% | 16 |
| 395 | gamma-glutamyltranspeptidase 1 precursor | 73915090 | 61 | 8 | 16% | 16 |
| 396 | serine protease 58 precursor | 48255915 | 27 | 5 | 25% | 16 |
| 397 | azurocidin preproprotein | 11342670 | 27 | 5 | 29% | 16 |
| 398 | apolipoprotein A-I-binding protein precursor | 91984773 | 32 | 6 | 31% | 16 |
| 399 | S-adenosylmethionine synthase isoform type-2 | 5174529 | 44 | 6 | 19% | 16 |
| 400 | alpha-1-antichymotrypsin precursor | 50659080 | 48 | 6 | 19% | 16 |
| 401 | DDB1- and CUL4-associated factor 7 . | 108936958 | 39 | 7 | 23% | 16 |
| 402 | arylsulfatase A isoform a precursor . | 313569791 | 54 | 7 | 20% | 16 |
| 403 | testis-specific serine kinase substrate | 11119430 | 65 | 5 | 8.40% | 16 |
| 404 | threonyl-tRNA synthetase, cytoplasmic | 38202255 | 83 | 7 | 10% | 16 |
| 405 | probable Xaa-Pro aminopeptidase 3 isoform 1 | 11559925 | 57 | 6 | 21% | 16 |
| 406 | adenylate kinase isoenzyme 1 | 4502011 | 22 | 4 | 19% | 15 |
| 407 | delta(3,5)-Delta(2,4)-dienoyl-CoA isomerase, mitochondrial precursor | 70995211 | 36 | 4 | 11% | 15 |
| 408 | uncharacterized protein C20orf107 precursor | 71043642 | 19 | 5 | 24% | 15 |
| 409 | carboxypeptidase Z isoform 2 precursor | 62388875 | 73 | 6 | 5.60% | 15 |
| 410 | NME1-NME2 protein | 66392203 | 30 | 5 | 21% | 15 |
| 411 | ras-related protein Rab-1B | 13569962 | 22 | 4 | 21% | 15 |
| 412 | prostate and testis expressed protein 1 precursor | 19923082 | 14 | 3 | 28% | 15 |
| 413 | malate dehydrogenase, cytoplasmic isoform 1 | 312283701 | 39 | 5 | 16% | 15 |
| 414 | 26S proteasome non-ATPase regulatory subunit 6 | 7661914 | 46 | 6 | 11% | 15 |
| 415 | cystatin-S precursor | 4503109 | 16 | 5 | 42% | 15 |
| 416 | basigin isoform 2 precursor | 38372925 | 29 | 5 | 15% | 15 |
| 417 | CD63 antigen isoform A | 4502679 | 26 | 2 | 5.00% | 15 |
| 418 | acid sphingomyelinase-like phosphodiesterase 3b isoform 1 precursor | 57242798 | 51 | 5 | 17% | 15 |
| 419 | acrosin precursor | 148613878 | 46 | 9 | 24% | 14 |
| 420 | ATP synthase subunit b, mitochondrial precursor | 21361565 | 29 | 4 | 22% | 14 |
| 421 | T-complex protein 1 subunit zeta isoform a | 4502643 | 58 | 6 | 8.30% | 14 |
| 422 | dolichyl-diphosphooligosaccharide--protein glycosyltransferase subunit 2 isoform 2 precursor | 209413738 | 68 | 5 | 15% | 14 |
| 423 | fructose-bisphosphate aldolase C | 4885063 | 39 | 2 | 17% | 14 |
| 424 | importin subunit alpha-2 | 4504897 | 58 | 4 | 11% | 14 |
| 425 | nuclear pore complex protein Nup93 isoform 1 | 208609990 | 93 | 5 | 4.80% | 14 |
| 426 | SYNJ2BP-COX16 protein isoform 1 | 321400118 | 21 | 4 | 25% | 14 |
| 427 | eukaryotic translation initiation factor 5A-1 isoform B | 219555712 | 17 | 4 | 23% | 14 |
| 428 | transmembrane protein 190 precursor | 21040263 | 19 | 3 | 27% | 14 |
| 429 | translin | 4759270 | 26 | 3 | 20% | 14 |
| 430 | fragile X mental retardation 1 neighbor protein | 22749199 | 29 | 4 | 20% | 14 |
| 431 | annexin A11 | 22165431 | 54 | 4 | 8.30% | 14 |
| 432 | ubiquitin carboxyl-terminal hydrolase 14 isoform b | 82880645 | 52 | 3 | 12% | 14 |
| 433 | acyl-CoA-binding protein isoform 5 | 295842514 | 14 | 5 | 44% | 14 |
| 434 | kinesin-1 heavy chain | 4758648 | 110 | 6 | 4.50% | 14 |
| 435 | ribose-phosphate pyrophosphokinase 2 isoform 2 | 4506129 | 35 | 5 | 19% | 14 |
| 436 | transaldolase | 5803187 | 38 | 6 | 19% | 14 |
| 437 | calcium/calmodulin-dependent protein kinase type II subunit delta isoform 2 | 26667189 | 54 | 5 | 9.60% | 14 |
| 438 | inosine-5'-monophosphate dehydrogenase 2 | 66933016 | 56 | 4 | 9.90% | 14 |
| 439 | plasma glutamate carboxypeptidase precursor | 7706387 | 52 | 6 | 18% | 14 |
| 440 | proteasome subunit beta type-8 isoform E2 proprotein [Homo | 73747875 | 30 | 7 | 25% | 14 |
| 441 | cytoplasmic dynein 1 intermediate chain 2 | 24307879 | 71 | 7 | 16% | 14 |
| 442 | ras-related protein Ral-B | 4506405 | 23 | 3 | 31% | 14 |
| 443 | inositol-3-phosphate synthase 1 isoform 2 | 283135176 | 55 | 6 | 17% | 14 |
| 444 | trifunctional enzyme subunit beta, mitochondrial precursor | 4504327 | 51 | 3 | 4.20% | 13 |
| 445 | protein FAM71B | 222418633 | 65 | 4 | 9.10% | 13 |
| 446 | synaptophysin-like protein 1 isoform a | 5803185 | 29 | 3 | 13% | 13 |
| 447 | L-lactate dehydrogenase A chain isoform 3 | 260099723 | 40 | 4 | 15% | 13 |
| 448 | synaptic vesicle membrane protein VAT-1 homolog | 18379349 | 42 | 5 | 19% | 13 |
| 449 | vesicle-fusing ATPase | 156564401 | 83 | 4 | 5.80% | 13 |
| 450 | 40S ribosomal protein S3 | 15718687 | 27 | 6 | 17% | 13 |
| 451 | receptor expression-enhancing protein 5 | 115430112 | 21 | 4 | 15% | 13 |
| 452 | midkine precursor | 4505135 | 16 | 3 | 23% | 13 |
| 453 | interferon-inducible GTPase 5 | 10257429 | 50 | 4 | 14% | 13 |
| 454 | serine/threonine-protein phosphatase PP1-beta catalytic subunit isoform 1 | 4506005 | 37 | 3 | 11% | 13 |
| 455 | 40S ribosomal protein S12 | 14277700 | 15 | 4 | 42% | 13 |
| 456 | poly(rC)-binding protein 2 isoform b | 14141166 | 38 | 4 | 23% | 13 |
| 457 | histone H2B type 1-M | 4504263 | 14 | 2 | 36% | 13 |
| 458 | ubiquitin carboxyl-terminal hydrolase 5 isoform 2 | 148727247 | 93 | 4 | 4.00% | 13 |
| 459 | dynein intermediate chain 2, axonemal isoform 1 . | 217416452 | 69 | 7 | 17% | 13 |
| 460 | talin-1 | 223029410 | 270 | 7 | 4.00% | 13 |
| 461 | bleomycin hydrolase . | 4557367 | 53 | 5 | 15% | 13 |
| 462 | protein S100-A7 . | 115298657 | 11 | 4 | 25% | 13 |
| 463 | heat shock 70 protein 4L | 31541941 | 95 | 4 | 6.00% | 12 |
| 464 | 60S acidic ribosomal protein P0 | 16933546 | 34 | 4 | 9.50% | 12 |
| 465 | alpha-crystallin B chain | 4503057 | 20 | 4 | 18% | 12 |
| 466 | thioredoxin-related transmembrane protein 4 precursor | 40254947 | 39 | 2 | 7.20% | 12 |
| 467 | sodium/potassium-transporting ATPase subunit alpha-4 isoform 1 | 153946397 | 114 | 5 | 4.40% | 12 |
| 468 | carbonyl reductase [NADPH] 1 | 4502599 | 30 | 5 | 15% | 12 |
| 469 | flavin reductase (NADPH) | 4502419 | 22 | 3 | 16% | 12 |
| 470 | sperm acrosome membrane-associated protein 4 precursor | 19424138 | 13 | 2 | 22% | 12 |
| 471 | cytochrome c oxidase subunit 6B1 | 4502985 | 10 | 4 | 44% | 12 |
| 472 | histone H1t . | 20544168 | 22 | 4 | 18% | 12 |
| 473 | V-type proton ATPase catalytic subunit A | 19913424 | 68 | 3 | 6.60% | 12 |
| 474 | 3-oxoacyl-[acyl-carrier-protein] synthase, mitochondrial isoform 1 | 8923559 | 49 | 7 | 33% | 12 |
| 475 | importin-4 | 62460637 | 119 | 6 | 4.00% | 12 |
| 476 | glycerophosphodiester phosphodiesterase 1 | 7706617 | 38 | 4 | 10.00% | 12 |
| 477 | endoplasmic reticulum-Golgi intermediate compartment protein 3 isoform a | 38327615 | 44 | 4 | 11% | 12 |
| 478 | cysteine-rich secretory protein 2 precursor | 215490018 | 27 | 4 | 16% | 12 |
| 479 | histone H2A.V isoform 1 | 6912616 | 14 | 2 | 31% | 12 |
| 480 | transferrin receptor protein 1 | 189458817 | 85 | 8 | 12% | 12 |
| 481 | probable aminopeptidase NPEPL1 isoform 3 | 325652118 | 51 | 6 | 16% | 12 |
| 482 | neutral alpha-glucosidase AB isoform 2 precursor | 38202257 | 107 | 3 | 3.50% | 11 |
| 483 | radial spoke head protein 9 homolog isoform 1 | 32964825 | 31 | 4 | 14% | 11 |
| 484 | radial spoke head 1 homolog | 18254456 | 35 | 3 | 13% | 11 |
| 485 | plastin-3 isoform 1 | 209862851 | 71 | 2 | 11% | 11 |
| 486 | dolichyl-diphosphooligosaccharide--protein glycosyltransferase 48 subunit precursor | 20070197 | 51 | 6 | 11% | 11 |
| 487 | nuclear pore complex protein Nup155 isoform 1 | 24430149 | 155 | 5 | 2.80% | 11 |
| 488 | adenylate kinase 7 | 148727333 | 83 | 4 | 8.00% | 11 |
| 489 | endophilin-B1 isoform 2 | 331284170 | 44 | 5 | 11% | 11 |
| 490 | lactadherin isoform a preproprotein | 167830475 | 43 | 4 | 14% | 11 |
| 491 | 26S protease regulatory subunit 7 isoform 1 | 4506209 | 49 | 4 | 9.50% | 11 |
| 492 | L-lactate dehydrogenase B chain | 4557032 | 37 | 2 | 12% | 11 |
| 493 | glycogen phosphorylase, brain form | 21361370 | 97 | 7 | 7.80% | 11 |
| 494 | tropomyosin alpha-3 chain isoform 2 | 24119203 | 29 | 5 | 33% | 11 |
| 495 | neutrophil defensin 3 preproprotein | 4885179 | 10 | 4 | 27% | 11 |
| 496 | NADH dehydrogenase [ubiquinone] iron-sulfur protein 6, mitochondrial precursor | 4758792 | 14 | 4 | 43% | 11 |
| 497 | neutrophil gelatinase-associated lipocalin precursor | 38455402 | 23 | 4 | 28% | 11 |
| 498 | multifunctional protein ADE2 isoform 2 | 5453539 | 47 | 4 | 9.20% | 11 |
| 499 | sulfhydryl oxidase 1 isoform a precursor | 13325075 | 83 | 6 | 11% | 11 |
| 500 | arginase-2, mitochondrial precursor . | 4502215 | 39 | 7 | 28% | 11 |
| 501 | lipoprotein lipase precursor | 4557727 | 53 | 4 | 7.40% | 10 |
| 502 | 14-3-3 protein sigma | 5454052 | 28 | 2 | 18% | 10 |
| 503 | T-complex protein 1 subunit zeta-2 isoform 1 | 58331173 | 58 | 3 | 8.30% | 10 |
| 504 | uncharacterized protein C20orf106 precursor | 71043622 | 20 | 2 | 35% | 10 |
| 505 | isocitrate dehydrogenase [NAD] subunit alpha, mitochondrial precursor | 5031777 | 40 | 5 | 14% | 10 |
| 506 | 14-3-3 protein beta/alpha | 21328448 | 28 | 2 | 23% | 10 |
| 507 | beta-defensin 129 precursor | 18250304 | 20 | 3 | 11% | 10 |
| 508 | lipid phosphate phosphohydrolase 1 isoform 1 | 29171736 | 32 | 3 | 10% | 10 |
| 509 | protein S100-A11 | 5032057 | 12 | 4 | 25% | 10 |
| 510 | LETM1 and EF-hand domain-containing protein 1, mitochondrial precursor | 6912482 | 83 | 4 | 4.70% | 10 |
| 511 | NADH dehydrogenase [ubiquinone] flavoprotein 1, mitochondrial isoform 1 precursor | 20149568 | 51 | 3 | 8.00% | 10 |
| 512 | phosphate carrier protein, mitochondrial isoform b precursor | 47132595 | 40 | 4 | 10% | 10 |
| 513 | 40S ribosomal protein S18 | 11968182 | 18 | 2 | 13% | 10 |
| 514 | calicin | 169636428 | 67 | 2 | 4.10% | 10 |
| 515 | arginyl-tRNA synthetase, cytoplasmic | 15149476 | 75 | 5 | 4.80% | 10 |
| 516 | 40S ribosomal protein S25 | 4506707 | 14 | 2 | 15% | 10 |
| 517 | hornerin | 57864582 | 282 | 2 | 1.50% | 10 |
| 518 | histone H2A-Bbd type 2/3 | 63029935 | 13 | 2 | 27% | 10 |
| 519 | glycerol kinase isoform a | 42794763 | 58 | 2 | 7.70% | 10 |
| 520 | glutathione S-transferase omega-2 isoform 1 | 38016131 | 28 | 5 | 24% | 10 |
| 521 | acetyl-CoA acetyltransferase, cytosolic | 148539872 | 41 | 3 | 16% | 10 |
| 522 | prostaglandin-H2 D-isomerase precursor | 32171249 | 21 | 5 | 33% | 10 |
| 523 | integrin alpha-M isoform 1 precursor | 224831239 | 127 | 6 | 7.50% | 10 |
| 524 | CD9 antigen | 4502693 | 25 | 4 | 14% | 10 |
| 525 | macrophage migration inhibitory factor | 4505185 | 12 | 3 | 36% | 10 |
| 526 | ubiquitin fusion degradation protein 1 homolog isoform B | 241982785 | 30 | 3 | 14% | 10 |
| 527 | gamma-glutamylcyclotransferase isoform 1 . | 13129018 | 21 | 4 | 27% | 10 |
| 528 | alpha-1-acid glycoprotein 2 precursor | 4505529 | 24 | 2 | 22% | 10 |
| 529 | annexin A4 | 4502105 | 36 | 3 | 7.50% | 9 |
| 530 | sperm-associated antigen 6 isoform 1 | 6912678 | 55 | 4 | 11% | 9 |
| 531 | succinate dehydrogenase [ubiquinone] iron-sulfur subunit, mitochondrial precursor | 115387094 | 32 | 4 | 10.00% | 9 |
| 532 | transmembrane protease serine 2 isoform 2 | 205360943 | 54 | 3 | 6.30% | 9 |
| 533 | pyruvate dehydrogenase E1 component subunit alpha, testis-specific form, mitochondrial precursor | 4885543 | 43 | 3 | 7.50% | 9 |
| 534 | sperm acrosome-associated protein 5 precursor | 120952755 | 18 | 3 | 21% | 9 |
| 535 | casein kinase II subunit alpha isoform a | 29570791 | 45 | 4 | 8.40% | 9 |
| 536 | carboxypeptidase D isoform 1 precursor | 22202611 | 153 | 3 | 4.10% | 9 |
| 537 | ATPase inhibitor, mitochondrial isoform 1 precursor | 7705927 | 12 | 2 | 7.50% | 9 |
| 538 | ATP synthase subunit g, mitochondrial | 51479156 | 11 | 3 | 32% | 9 |
| 539 | transmembrane protein 89 precursor | 56847630 | 18 | 3 | 17% | 9 |
| 540 | exportin-7 | 154448892 | 124 | 5 | 6.10% | 9 |
| 541 | serine/threonine-protein phosphatase 2A 65 regulatory subunit A alpha isoform | 21361399 | 65 | 4 | 10% | 9 |
| 542 | EF-hand domain-containing family member C2 | 31542743 | 87 | 3 | 3.20% | 9 |
| 543 | transketolase-like protein 1 isoform b | 225637461 | 65 | 3 | 7.80% | 9 |
| 544 | cytochrome b-c1 complex subunit 6, mitochondrial | 83627705 | 11 | 4 | 53% | 9 |
| 545 | exportin-2 | 29029559 | 110 | 5 | 4.20% | 9 |
| 546 | disintegrin and metalloproteinase domain-containing protein 32 | 148664238 | 88 | 4 | 3.60% | 9 |
| 547 | actin-related protein 2 isoform b | 5031571 | 45 | 2 | 4.80% | 9 |
| 548 | galectin-7 | 109948279 | 15 | 4 | 40% | 9 |
| 549 | sperm-associated antigen 16 protein isoform 1 | 70909324 | 71 | 4 | 8.60% | 9 |
| 550 | growth hormone-inducible transmembrane protein | 118200356 | 37 | 3 | 9.30% | 9 |
| 551 | alanyl-tRNA synthetase, cytoplasmic | 109148542 | 107 | 4 | 4.40% | 9 |
| 552 | spermine synthase | 21264341 | 41 | 4 | 13% | 9 |
| 553 | cation-dependent mannose-6-phosphate receptor isoform 1 precursor | 4505061 | 31 | 4 | 18% | 9 |
| 554 | nucleoporin Nup43 . | 38605733 | 42 | 3 | 12% | 9 |
| 555 | sialate O-acetylesterase isoform 1 precursor . | 24850115 | 58 | 3 | 5.00% | 9 |
| 556 | attractin isoform 1 preproprotein | 21450861 | 159 | 5 | 3.80% | 9 |
| 557 | neuroplastin isoform b precursor | 6912646 | 44 | 4 | 13% | 9 |
| 558 | neuroserpin precursor . | 170295807 | 46 | 4 | 11% | 9 |
| 559 | elongation factor 1-delta isoform 1 | 304555581 | 71 | 2 | 5.60% | 8 |
| 560 | transmembrane and coiled-coil domain-containing protein 2 | 56847610 | 20 | 2 | 8.80% | 8 |
| 561 | adenine phosphoribosyltransferase isoform a | 4502171 | 20 | 3 | 26% | 8 |
| 562 | dnaJ homolog subfamily B member 11 precursor | 7706495 | 41 | 4 | 13% | 8 |
| 563 | mRNA export factor | 62739173 | 41 | 4 | 13% | 8 |
| 564 | dnaJ homolog subfamily A member 2 | 5031741 | 46 | 2 | 3.90% | 8 |
| 565 | ras-related protein Rab-5C isoform a | 41393614 | 23 | 3 | 12% | 8 |
| 566 | apoptosis-inducing factor 1, mitochondrial isoform 2 precursor | 22202629 | 66 | 3 | 5.10% | 8 |
| 567 | coiled-coil domain-containing protein 105 | 226492892 | 57 | 4 | 4.60% | 8 |
| 568 | histidine triad nucleotide-binding protein 2, mitochondrial precursor | 14211923 | 17 | 3 | 25% | 8 |
| 569 | heat shock 70 protein 4 | 38327039 | 94 | 3 | 4.50% | 8 |
| 570 | epoxide hydrolase 2 | 27597073 | 63 | 3 | 5.20% | 8 |
| 571 | transforming protein RhoA precursor | 10835049 | 22 | 3 | 10% | 8 |
| 572 | platelet-activating factor acetylhydrolase IB subunit alpha | 4557741 | 47 | 4 | 15% | 8 |
| 573 | 60S acidic ribosomal protein P1 isoform 1 | 4506669 | 12 | 2 | 57% | 8 |
| 574 | UDP-glucose:glycoprotein glucosyltransferase 1 precursor | 9910280 | 177 | 4 | 2.40% | 8 |
| 575 | prostaglandin E synthase 3 | 23308579 | 19 | 3 | 25% | 8 |
| 576 | asparaginyl-tRNA synthetase, cytoplasmic | 4758762 | 63 | 4 | 7.50% | 8 |
| 577 | coatomer subunit beta | 7705369 | 107 | 2 | 2.20% | 8 |
| 578 | ADP-sugar pyrophosphatase . | 37594464 | 24 | 5 | 25% | 8 |
| 579 | isocitrate dehydrogenase [NADP], mitochondrial precursor | 28178832 | 51 | 2 | 8.40% | 8 |
| 580 | NADH-ubiquinone oxidoreductase 75 subunit, mitochondrial isoform 1 | 33519475 | 79 | 4 | 8.30% | 7 |
| 581 | 3-hydroxyisobutyrate dehydrogenase, mitochondrial precursor | 23308751 | 35 | 2 | 8.00% | 7 |
| 582 | matrix-remodeling-associated protein 5 precursor | 139948432 | 312 | 3 | 1.20% | 7 |
| 583 | prohibitin | 4505773 | 30 | 3 | 12% | 7 |
| 584 | protein-L-isoaspartate(D-aspartate) O-methyltransferase isoform 2 | 354983493 | 30 | 3 | 15% | 7 |
| 585 | transmembrane emp24 domain-containing protein 9 precursor | 39725636 | 27 | 2 | 8.10% | 7 |
| 586 | 26S proteasome non-ATPase regulatory subunit 13 isoform 1 | 157502193 | 43 | 5 | 9.60% | 7 |
| 587 | alpha-soluble NSF attachment protein | 47933379 | 33 | 3 | 13% | 7 |
| 588 | glypican-1 precursor | 167001141 | 62 | 4 | 10% | 7 |
| 589 | cadherin-1 preproprotein | 4757960 | 97 | 3 | 3.20% | 7 |
| 590 | stromal cell-derived factor 2-like protein 1 precursor | 56243533 | 24 | 3 | 19% | 7 |
| 591 | ubiquitin thioesterase OTUB1 | 109148508 | 31 | 2 | 7.00% | 7 |
| 592 | testis-specific H1 histone | 32401437 | 28 | 2 | 8.20% | 7 |
| 593 | 26S proteasome non-ATPase regulatory subunit 1 isoform 1 | 25777600 | 106 | 4 | 6.90% | 7 |
| 594 | keratin, type I cytoskeletal 18 | 4557888 | 48 | 2 | 5.60% | 7 |
| 595 | aspartyl-tRNA synthetase, cytoplasmic | 45439306 | 57 | 5 | 6.00% | 7 |
| 596 | beta-defensin 126 preproprotein . | 13624333 | 12 | 2 | 8.10% | 7 |
| 597 | disintegrin and metalloproteinase domain-containing protein 29 preproprotein | 73765552 | 93 | 4 | 6.20% | 7 |
| 598 | disintegrin and metalloproteinase domain-containing protein 30 preproprotein | 31881770 | 89 | 3 | 4.30% | 7 |
| 599 | tissue alpha-L-fucosidase precursor . | 119360348 | 54 | 3 | 8.40% | 7 |
| 600 | coatomer subunit beta' | 4758032 | 102 | 4 | 3.10% | 7 |
| 601 | oligoribonuclease, mitochondrial precursor . | 224496106 | 27 | 3 | 14% | 7 |
| 602 | endonuclease domain-containing 1 protein precursor | 148225659 | 55 | 3 | 7.00% | 7 |
| 603 | ribose-5-phosphate isomerase . | 94536842 | 33 | 5 | 24% | 7 |
| 604 | UPF0160 protein MYG1, mitochondrial precursor . | 145275185 | 42 | 4 | 12% | 7 |
| 605 | N-acetylgalactosamine-6-sulfatase precursor . | 4503899 | 58 | 2 | 8.00% | 7 |
| 606 | NADH dehydrogenase [ubiquinone] iron-sulfur protein 3, mitochondrial precursor | 4758788 | 30 | 2 | 11% | 6 |
| 607 | receptor expression-enhancing protein 6 | 19923919 | 21 | 2 | 9.80% | 6 |
| 608 | plasma membrane calcium-transporting ATPase 4 isoform 4b | 48255957 | 134 | 2 | 2.30% | 6 |
| 609 | membrane-associated progesterone receptor component 2 | 291621647 | 26 | 2 | 8.10% | 6 |
| 610 | tripeptidyl-peptidase 2 | 186972143 | 138 | 3 | 2.00% | 6 |
| 611 | 26S proteasome non-ATPase regulatory subunit 2 | 25777602 | 100 | 2 | 3.30% | 6 |
| 612 | dynactin subunit 2 | 5453629 | 45 | 2 | 5.90% | 6 |
| 613 | signal peptidase complex subunit 3 | 11345462 | 20 | 3 | 18% | 6 |
| 614 | long-chain-fatty-acid--CoA ligase 6 isoform e | 327412327 | 79 | 2 | 4.70% | 6 |
| 615 | thioredoxin isoform 1 | 50592994 | 12 | 2 | 24% | 6 |
| 616 | cystatin-B | 4503117 | 11 | 4 | 55% | 6 |
| 617 | translocon-associated protein subunit alpha precursor | 169404009 | 32 | 2 | 6.60% | 6 |
| 618 | metalloproteinase inhibitor 1 precursor | 4507509 | 23 | 3 | 23% | 6 |
| 619 | cytochrome c oxidase subunit 7A2, mitochondrial precursor | 262118227 | 13 | 2 | 20% | 6 |
| 620 | lysosome membrane protein 2 isoform 1 precursor | 5031631 | 54 | 3 | 7.70% | 6 |
| 621 | 40S ribosomal protein S8 | 4506743 | 24 | 2 | 13% | 6 |
| 622 | transketolase-like protein 2 . | 133778974 | 68 | 2 | 3.70% | 6 |
| 623 | ras-related protein Rap-1b isoform 1 precursor | 7661678 | 21 | 3 | 20% | 6 |
| 624 | tripeptidyl-peptidase 1 preproprotein | 5729770 | 61 | 3 | 6.20% | 6 |
| 625 | 26S proteasome non-ATPase regulatory subunit 12 isoform 1 | 4506221 | 53 | 2 | 4.80% | 6 |
| 626 | BAG family molecular chaperone regulator 5 isoform b | 6631077 | 51 | 3 | 9.80% | 6 |
| 627 | vitronectin precursor | 88853069 | 54 | 2 | 5.20% | 6 |
| 628 | mitochondrial 2-oxoglutarate/malate carrier protein isoform 1 | 21361114 | 34 | 2 | 7.00% | 6 |
| 629 | 2-oxoisovalerate dehydrogenase subunit alpha, mitochondrial isoform | 11386135 | 50 | 3 | 11% | 6 |
| 630 | clathrin light chain A isoform a . | 4502899 | 24 | 3 | 9.20% | 6 |
| 631 | 6-phosphofructokinase, liver type | 48762920 | 85 | 2 | 3.60% | 6 |
| 632 | C-Myc-binding protein | 57242777 | 12 | 2 | 36% | 6 |
| 633 | thioredoxin domain-containing protein 2 isoform 2 | 148727319 | 60 | 2 | 7.10% | 6 |
| 634 | heterogeneous nuclear ribonucleoprotein K isoform b | 14165435 | 51 | 2 | 5.80% | 6 |
| 635 | antileukoproteinase precursor | 4507065 | 14 | 2 | 15% | 6 |
| 636 | dehydrogenase/reductase SDR family member 7 precursor | 7706318 | 38 | 3 | 9.10% | 6 |
| 637 | matrix metalloproteinase-9 preproprotein | 74272287 | 78 | 4 | 6.20% | 6 |
| 638 | neurofilament heavy polypeptide | 32483416 | 112 | 2 | 3.20% | 6 |
| 639 | polyamine-modulated factor 1-binding protein 1 isoform a | 237858619 | 117 | 2 | 1.60% | 6 |
| 640 | pancreatic secretory granule membrane major glycoprotein GP2 isoform 1 precursor | 119220569 | 59 | 3 | 7.30% | 6 |
| 641 | complement C1q tumor necrosis factor-related protein 1 isoform 1 | 13569944 | 32 | 2 | 6.40% | 6 |
| 642 | alpha-N-acetylglucosaminidase precursor | 66346698 | 82 | 4 | 6.90% | 6 |
| 643 | 40S ribosomal protein S5 . | 13904870 | 23 | 2 | 14% | 6 |
| 644 | alpha-N-acetylgalactosaminidase precursor | 4557781 | 47 | 4 | 13% | 6 |
| 645 | N(4)-(beta-N-acetylglucosaminyl)-L-asparaginase isoform 1 | 285002251 | 37 | 2 | 12% | 6 |
| 646 | immunoglobulin lambda-like polypeptide 5 isoform 1 . | 295986608 | 23 | 2 | 14% | 6 |
| 647 | Golgi apparatus protein 1 isoform 2 precursor . | 224586815 | 136 | 4 | 3.90% | 6 |
| 648 | N-sulphoglucosamine sulphohydrolase precursor | 4506919 | 57 | 3 | 7.40% | 6 |
| 649 | growth arrest-specific protein 1 precursor . | 167466169 | 36 | 3 | 12% | 6 |
| 650 | HLA class II histocompatibility antigen, DR alpha chain precursor | 52426774 | 29 | 2 | 11% | 6 |
| 651 | IgGFc-binding protein precursor | 154146262 | 572 | 3 | 0.67% | 6 |
| 652 | keratin, type II cytoskeletal 4 | 331999954 | 56 | 2 | 5.60% | 5 |
| 653 | endoplasmic reticulum resident protein 44 precursor | 52487191 | 47 | 3 | 9.90% | 5 |
| 654 | ribonuclease-like protein 13 precursor | 59276062 | 18 | 2 | 11% | 5 |
| 655 | translationally-controlled tumor protein | 4507669 | 20 | 2 | 16% | 5 |
| 656 | 40S ribosomal protein S3a | 4506723 | 30 | 2 | 6.10% | 5 |
| 657 | stress-induced-phosphoprotein 1 | 5803181 | 63 | 3 | 6.40% | 5 |
| 658 | heme-binding protein 2 | 7657603 | 23 | 2 | 9.80% | 5 |
| 659 | 26S proteasome non-ATPase regulatory subunit 3 | 25777612 | 61 | 4 | 10% | 5 |
| 660 | F-actin-capping protein subunit beta isoform 1 | 4826659 | 31 | 2 | 8.80% | 5 |
| 661 | acyl-protein thioesterase 1 | 5453722 | 25 | 2 | 12% | 5 |
| 662 | dnaJ homolog subfamily A member 4 isoform 2 | 194328760 | 45 | 2 | 4.80% | 5 |
| 663 | glutathione S-transferase Mu 1 isoform 1 | 23065544 | 26 | 2 | 14% | 5 |
| 664 | atlastin-3 | 45827806 | 61 | 3 | 5.40% | 5 |
| 665 | uncharacterized protein C6orf81 | 31542280 | 41 | 2 | 8.20% | 5 |
| 666 | carnitine O-palmitoyltransferase 1, muscle isoform isoform a | 223468678 | 88 | 2 | 3.00% | 5 |
| 667 | trafficking protein particle complex subunit 3 | 7656926 | 20 | 2 | 13% | 5 |
| 668 | sarcoplasmic/endoplasmic reticulum calcium ATPase 2 isoform b | 24638454 | 115 | 3 | 4.00% | 5 |
| 669 | 40S ribosomal protein S16 | 4506691 | 16 | 2 | 12% | 5 |
| 670 | dickkopf-like protein 1 isoform 2 precursor | 308818218 | 24 | 2 | 9.00% | 5 |
| 671 | lanC-like protein 1 . | 212274337 | 45 | 2 | 6.30% | 5 |
| 672 | spermatid-associated protein | 22749425 | 52 | 2 | 5.10% | 5 |
| 673 | protein AHNAK2 | 156766050 | 617 | 2 | 0.78% | 5 |
| 674 | inorganic pyrophosphatase 2, mitochondrial isoform 1 precursor | 29171702 | 38 | 3 | 11% | 5 |
| 675 | semaphorin-3C precursor | 5454048 | 85 | 2 | 5.10% | 5 |
| 676 | 26S proteasome non-ATPase regulatory subunit 5 | 4826952 | 56 | 3 | 8.10% | 5 |
| 677 | nuclear pore complex protein Nup214 | 33946327 | 214 | 2 | 1.10% | 5 |
| 678 | phospholipase A2, membrane associated precursor | 239915991 | 16 | 2 | 18% | 5 |
| 679 | carcinoembryonic antigen-related cell adhesion molecule 6 precursor | 40255013 | 37 | 2 | 9.90% | 5 |
| 680 | carcinoembryonic antigen-related cell adhesion molecule 8 precursor | 21314600 | 38 | 3 | 11% | 5 |
| 681 | EF-hand domain-containing protein KIAA0494 | 7662160 | 55 | 2 | 7.70% | 5 |
| 682 | coronin-1B | 14149734 | 54 | 2 | 5.30% | 5 |
| 683 | amyotrophic lateral sclerosis 2 chromosomal region candidate gene 12 protein isoform a | 189011586 | 52 | 2 | 4.30% | 5 |
| 684 | baculoviral IAP repeat-containing protein 6 | 153792694 | 530 | 2 | 0.86% | 5 |
| 685 | HLA class I histocompatibility antigen, A-1 alpha chain precursor | 24797067 | 41 | 2 | 10% | 5 |
| 686 | methionine adenosyltransferase 2 subunit beta isoform 1 | 11034825 | 38 | 2 | 7.50% | 5 |
| 687 | kunitz-type protease inhibitor 1 isoform 1 precursor [Homo | 32313599 | 58 | 3 | 5.90% | 5 |
| 688 | mammalian ependymin-related protein 1 isoform 1 precursor [Homo | 345110632 | 25 | 2 | 8.50% | 5 |
| 689 | cysteine-rich with EGF-like domain protein 2 isoform a precursor | 205360956 | 44 | 2 | 6.00% | 5 |
| 690 | lysosomal alpha-mannosidase isoform 2 precursor | 291045220 | 114 | 4 | 6.20% | 5 |
| 691 | transmembrane 9 superfamily member 3 precursor . | 190194386 | 68 | 2 | 4.80% | 5 |
| 692 | carboxypeptidase O precursor . | 27436871 | 43 | 2 | 7.80% | 5 |
| 693 | tetraspanin-1 . | 21264578 | 26 | 2 | 7.50% | 5 |
| 694 | thioredoxin reductase 3 isoform 1 . | 291045266 | 71 | 2 | 9.20% | 5 |
| 695 | mitochondrial inner membrane protein isoform 3 | 154354966 | 83 | 2 | 3.20% | 4 |
| 696 | sperm surface protein Sp17 | 8394343 | 17 | 2 | 9.90% | 4 |
| 697 | uncharacterized protein KIAA1683 isoform a | 224451032 | 147 | 2 | 2.60% | 4 |
| 698 | ATP synthase subunit O, mitochondrial precursor | 4502303 | 23 | 2 | 14% | 4 |
| 699 | ADP-ribosylation factor 1 | 4502201 | 21 | 2 | 15% | 4 |
| 700 | epididymal secretory protein E3-beta precursor | 11641279 | 18 | 2 | 16% | 4 |
| 701 | collagen alpha-1(XVIII) chain isoform 1 precursor | 110611235 | 154 | 2 | 2.40% | 4 |
| 702 | parkin coregulated gene protein isoform 2 | 122939202 | 29 | 2 | 8.90% | 4 |
| 703 | EF-hand calcium-binding domain-containing protein 6 isoform a | 38570107 | 173 | 2 | 1.30% | 4 |
| 704 | lysyl-tRNA synthetase isoform 1 | 194272210 | 71 | 2 | 2.70% | 4 |
| 705 | cAMP-dependent protein kinase catalytic subunit alpha isoform 2 | 46909584 | 40 | 2 | 6.10% | 4 |
| 706 | ras-related protein Rab-3B | 19923750 | 25 | 2 | 12% | 4 |
| 707 | izumo sperm-egg fusion protein 2 precursor | 63999117 | 25 | 2 | 7.70% | 4 |
| 708 | acyl carrier protein, mitochondrial precursor | 4826852 | 17 | 2 | 12% | 4 |
| 709 | thioredoxin-dependent peroxide reductase, mitochondrial isoform b | 32483377 | 26 | 2 | 11% | 4 |
| 710 | dnaJ homolog subfamily B member 8 | 23503241 | 26 | 2 | 9.90% | 4 |
| 711 | mitochondrial dicarboxylate carrier | 20149598 | 31 | 2 | 8.40% | 4 |
| 712 | gamma-glutamyl hydrolase precursor | 4503987 | 36 | 2 | 8.80% | 4 |
| 713 | chloride intracellular channel protein 4 | 7330335 | 29 | 2 | 9.50% | 4 |
| 714 | proteasome activator complex subunit 4 | 163644283 | 211 | 3 | 2.90% | 4 |
| 715 | 60S ribosomal protein L7a | 4506661 | 30 | 2 | 5.30% | 4 |
| 716 | ras-related C3 botulinum toxin substrate 1 isoform Rac1b | 9845509 | 23 | 2 | 11% | 4 |
| 717 | nucleoside diphosphate kinase 7 isoform a | 7019465 | 42 | 3 | 5.90% | 4 |
| 718 | protein ERGIC-53 precursor | 5031873 | 58 | 2 | 3.70% | 4 |
| 719 | nuclear pore complex protein Nup205 | 57634534 | 228 | 2 | 0.99% | 4 |
| 720 | isoleucyl-tRNA synthetase, cytoplasmic | 94721239 | 145 | 2 | 1.40% | 4 |
| 721 | nascent polypeptide-associated complex subunit alpha isoform a | 333033787 | 95 | 2 | 3.10% | 4 |
| 722 | transducin beta-like protein 2 precursor | 7549793 | 50 | 2 | 4.50% | 4 |
| 723 | serine/threonine-protein kinase SMG1 | 62243658 | 410 | 2 | 0.41% | 4 |
| 724 | espin | 110431370 | 92 | 2 | 3.00% | 4 |
| 725 | UDP-glucose 6-dehydrogenase isoform 1 | 4507813 | 55 | 2 | 6.70% | 4 |
| 726 | WD repeat-containing protein 1 isoform 1 | 9257257 | 66 | 2 | 5.00% | 4 |
| 727 | sialic acid synthase | 12056473 | 40 | 2 | 6.70% | 4 |
| 728 | nuclear transport factor 2 | 5031985 | 14 | 2 | 17% | 4 |
| 729 | vacuolar protein sorting-associated protein 35 | 17999541 | 92 | 3 | 5.00% | 4 |
| 730 | glutathione S-transferase omega-1 isoform 1 | 4758484 | 28 | 2 | 7.50% | 4 |
| 731 | 60S ribosomal protein L15 isoform 1 | 15431293 | 24 | 2 | 10% | 4 |
| 732 | glycine cleavage system H protein, mitochondrial precursor [Homo | 49574537 | 19 | 2 | 17% | 4 |
| 733 | lambda-crystallin homolog | 115430219 | 35 | 2 | 6.90% | 4 |
| 734 | polymeric immunoglobulin receptor precursor | 31377806 | 83 | 3 | 4.60% | 4 |
| 735 | NIF3-like protein 1 isoform 1 | 209862879 | 42 | 3 | 14% | 4 |
| 736 | phospholipase A-2-activating protein | 72534670 | 87 | 2 | 4.00% | 4 |
| 737 | calcineurin-like phosphoesterase domain-containing protein 1 isoform a | 153251270 | 36 | 3 | 15% | 4 |
| 738 | ribosyldihydronicotinamide dehydrogenase [quinone] . | 156564357 | 26 | 2 | 15% | 4 |
| 739 | copper chaperone for superoxide dismutase | 4826665 | 29 | 2 | 7.70% | 4 |
| 740 | tetraspanin-6 isoform a . | 4507541 | 28 | 2 | 8.60% | 4 |
| 741 | lysosomal acid phosphatase isoform 1 precursor | 4557010 | 48 | 2 | 5.20% | 4 |
| 742 | nucleoporin Nup37 . | 34222121 | 37 | 2 | 10% | 4 |
| 743 | ATP-dependent (S)-NAD(P)H-hydrate dehydratase isoform b [Homo | 338968897 | 37 | 2 | 6.90% | 4 |
| 744 | hydroxyacyl-coenzyme A dehydrogenase, mitochondrial isoform 1 precursor | 296179427 | 36 | 2 | 6.30% | 3 |
| 745 | solute carrier family 2, facilitated glucose transporter member 14 | 23592238 | 56 | 2 | 3.50% | 3 |
| 746 | eukaryotic initiation factor 4A-I isoform 1 | 4503529 | 46 | 3 | 12% | 3 |
| 747 | phosphoglycerate mutase 2 | 50593010 | 29 | 3 | 14% | 3 |
| 748 | methionyl-tRNA synthetase, cytoplasmic | 14043022 | 101 | 2 | 3.60% | 3 |
| 749 | large proline-rich protein BAG6 isoform a | 149158692 | 119 | 2 | 2.00% | 3 |
| 750 | cullin-3 | 4503165 | 89 | 2 | 2.90% | 3 |
| 751 | nuclear migration protein nudC | 5729953 | 38 | 2 | 5.40% | 3 |
| 752 | destrin isoform a | 5802966 | 19 | 2 | 11% | 3 |
| 753 | protein ALEX XLas | 117938759 | 111 | 2 | 2.20% | 3 |
| 754 | transmembrane emp24 domain-containing protein 1 precursor | 5803040 | 25 | 2 | 9.70% | 3 |
| 755 | cartilage acidic protein 1 isoform B precursor . | 330688397 | 70 | 2 | 4.80% | 3 |
| 756 | rab GDP dissociation inhibitor alpha | 4503971 | 51 | 2 | 5.60% | 3 |
| 757 | protein arginine N-methyltransferase 5 isoform a | 20070220 | 73 | 2 | 5.30% | 3 |
| 758 | beta-hexosaminidase subunit beta preproprotein | 4504373 | 63 | 2 | 5.40% | 3 |
| 759 | cytosolic 5'-nucleotidase 1B isoform 4 | 312283642 | 71 | 2 | 3.50% | 3 |
| 760 | myeloblastin precursor | 71361688 | 28 | 3 | 17% | 3 |
| 761 | AP-1 complex subunit beta-1 isoform a | 260436862 | 105 | 2 | 2.70% | 3 |
| 762 | E3 ubiquitin-protein ligase UBR4 | 82659109 | 574 | 2 | 0.48% | 3 |
| 763 | protein Niban . | 16757970 | 103 | 2 | 1.80% | 3 |
| 764 | mucin-2 precursor . | 116284392 | 540 | 2 | 0.39% | 3 |
| 765 | growth/differentiation factor 15 precursor . | 153792495 | 34 | 2 | 10% | 3 |
| 766 | leukocyte elastase inhibitor | 13489087 | 43 | 2 | 13% | 3 |
| 767 | protein canopy homolog 3 precursor | 33942072 | 31 | 2 | 9.40% | 3 |
| 768 | phosphotriesterase-related protein isoform 1 . | 20070186 | 39 | 2 | 5.20% | 3 |
| 769 | hydroxymethylglutaryl-CoA lyase, mitochondrial isoform 1 precursor | 62198232 | 34 | 3 | 11% | 3 |
| 770 | ectonucleoside triphosphate diphosphohydrolase 3 . | 166197702 | 59 | 2 | 2.80% | 3 |
| 771 | enkurin | 21450721 | 29 | 2 | 12% | 2 |
| 772 | cytochrome b-c1 complex subunit Rieske, mitochondrial | 163644321 | 30 | 2 | 11% | 2 |
| 773 | epididymal secretory protein E3-alpha precursor | 11386189 | 18 | 2 | 10% | 2 |
| 774 | casein kinase II subunit beta | 23503295 | 25 | 2 | 13% | 2 |
| 775 | 3-hydroxyacyl-CoA dehydrogenase type-2 isoform 1 . | 4758504 | 27 | 2 | 12% | 2 |
| 776 | ATP synthase subunit epsilon, mitochondrial | 5901896 | 6 | 2 | 31% | 2 |
| 777 | disintegrin and metalloproteinase domain-containing protein 7 preproprotein | 114326453 | 86 | 2 | 2.50% | 2 |
| 778 | F-actin-capping protein subunit alpha-3 | 15277417 | 35 | 2 | 5.40% | 2 |
| 779 | ATP5J2-PTCD1 fusion protein | 311893396 | 84 | 2 | 3.20% | 2 |
| 780 | titin isoform N2-A | 291045225 | 3713 | 2 | 0.06% | 2 |
| 781 | secreted frizzled-related protein 1 precursor . | 56117838 | 35 | 2 | 6.10% | 2 |
| 782 | eukaryotic translation initiation factor 3 subunit M | 23397429 | 43 | 2 | 5.90% | 2 |
| 783 | long-chain-fatty-acid--CoA ligase 3 | 42794752 | 80 | 2 | 3.80% | 2 |
| 784 | zona pellucida-binding protein 2 isoform 1 precursor | 84875535 | 36 | 2 | 4.70% | 2 |
| 785 | bovine seminal plasma protein homolog 1 precursor | 190358548 | 16 | 2 | 17% | 2 |
| 786 | lysosomal Pro-X carboxypeptidase isoform 1 preproprotein | 4826940 | 56 | 2 | 5.80% | 2 |
| 787 | shootin-1 isoform a | 187761324 | 72 | 2 | 3.20% | 2 |
| 788 | presequence protease, mitochondrial isoform 1 precursor | 334085248 | 118 | 2 | 2.00% | 2 |
| 789 | major histocompatibility complex, class II, DR beta 5 precursor | 18641375 | 30 | 2 | 9.80% | 2 |
| 790 | armadillo repeat-containing protein 4 | 31657114 | 116 | 2 | 3.00% | 2 |
| 791 | insulin-degrading enzyme isoform 1 | 155969707 | 118 | 2 | 2.40% | 2 |
| 792 | peroxisomal membrane protein 11B isoform 2 . | 296317239 | 27 | 2 | 9.00% | 2 |
| 793 | translation initiation factor eIF-2B subunit alpha | 4503503 | 34 | 2 | 8.90% | 2 |
| 794 | protein CIP2A | 190194355 | 102 | 2 | 2.10% | 2 |
| 795 | angio-associated migratory cell protein . | 55743075 | 47 | 2 | 5.50% | 2 |
